# Computational fluid dynamic analysis of physical forces playing a role in brain organoid cultures in two different multiplex platforms

**DOI:** 10.1186/s12861-019-0183-y

**Published:** 2019-03-07

**Authors:** Livia Goto-Silva, Nadia M. E. Ayad, Iasmin L. Herzog, Nilton P. Silva, Bernard Lamien, Helcio R. B. Orlande, Annie da Costa Souza, Sidarta Ribeiro, Michele Martins, Gilberto B. Domont, Magno Junqueira, Fernanda Tovar-Moll, Stevens K. Rehen

**Affiliations:** 1 D’Or Institute for Research and Education (IDOR), Rua Diniz Cordeiro, 30 - Botafogo, Rio de Janeiro, RJ 22281-100 Brazil; 2 Department of Mechanical Engineering, Politecnica/COPPE - Federal University of Rio de Janeiro, UFRJ, Av. Horácio Macedo, 2030, Cidade Universitária, Rio de Janeiro, RJ 21941-914 Brazil; 3 Brain Institute, Federal University of Rio Grande do Norte, Av. Nascimento de Castro, 2155, Natal, RN 59056-450 Brazil; 4 Proteomics Unit, Institute of Chemistry, Federal University of Rio de Janeiro, UFRJ, Av. Athos da Silveira Ramos 149, Rio de Janeiro, 21941-909 Brazil; 5 Institute of Biomedical Sciences, Federal University of Rio de Janeiro, UFRJ, Av. Carlos Chagas Filho 373, Bloco K, Cidade Universitária, Rio de Janeiro, RJ 21941-902 Brazil

## Abstract

**Background:**

Organoid cultivation in suspension culture requires agitation at low shear stress to allow for nutrient diffusion, which preserves tissue structure. Multiplex systems for organoid cultivation have been proposed, but whether they meet similar shear stress parameters as the regularly used spinner flask and its correlation with the successful generation of brain organoids has not been determined.

**Results:**

Here we used computational fluid dynamics (CFD) to simulate two multiplex culture conditions: steering plates on an orbital shaker and the use of a previously described bioreactor. The bioreactor had low speed and high shear stress regions that may affect cell aggregate growth, depending on volume, whereas the computed variables of the steering plates were closer to those of the spinning flask.

**Conclusion:**

Our protocol improves the initial steps of the standard brain organoid formation, and the produced organoids displayed regionalized brain structures, including retinal pigmented cells. Overall, we conclude that suspension culture on orbital steering plates is a cost-effective practical alternative to previously described platforms for the cultivation of brain organoids for research and multiplex testing.

**Electronic supplementary material:**

The online version of this article (10.1186/s12861-019-0183-y) contains supplementary material, which is available to authorized users.

## Background

Three-dimensional (3D) cerebral organoids generated from human pluripotent stem cells (hPSCs) are complex structures that partly reproduce fetal brain development in vitro, making them powerful tools for the study of human development and disease [1]. The self-organization that occurs during hPSC differentiation in cerebral organoids allows for the appearance of complex structures, including those recapitulating regions of the cerebral cortex, ventral forebrain, midbrain, hindbrain, hippocampus, and retina [2–4]. Several research groups have used this model to study the development of diseases such as microcephaly, lissencephaly [5, 6], and Zika infection [7], as well as for drug testing [8].

Organoids are grown in 3D suspension culture, which enables efficient nutrient delivery to 3D organized tissue. Historically, cerebral organoids have been cultured in spinner flasks [9]. These flasks have the advantage of providing a low-shear environment [10], which is important because hPSCs have been shown to be sensitive to shear stress [10, 11]. However, spinner flasks have the disadvantage of requiring a high volume of cell culture media for cultivation, increasing the costs of experiments. Thus, they are limited to drug testing and other multiplex experiments including comparison of multiple patients and controls. Recently, Qian et al. (2016) [12] proposed the use of a 3D-printed scalable mini-bioreactor, the SpinΩ, which would be cost effective and provide a feasible, reproducible platform for chemical compound testing. However, cultivation in the SpinΩ still requires, the availability of 3D-printing equipment and other materials, which might make it infeasible for most laboratories.

The use of orbital shaker plates described originally [9] is a multiplex alternative to the often cost-prohibitive use of spinner flasks. However, whether the SpinΩ and orbital shaker plates provide the particle floating and nutrient mixing in a low-shear environment required to support organoid growth has not been addressed.

Here, we applied computational fluid dynamics (CFD) simulations to compute shear stress and fluid flow fields in orbital shaker plates and the SpinΩ. Additionally, we developed an improved protocol to support the initial steps of organoid development (static phase), including embryoid body (EB) formation and compared suspension cultures in the SpinΩ bioreactor and orbital shaker plates [9], in the initial 30-day cultivation period.

## Results

### Improved embryoid body (EB) formation and analysis of organoid growth

**Fig. 1:**
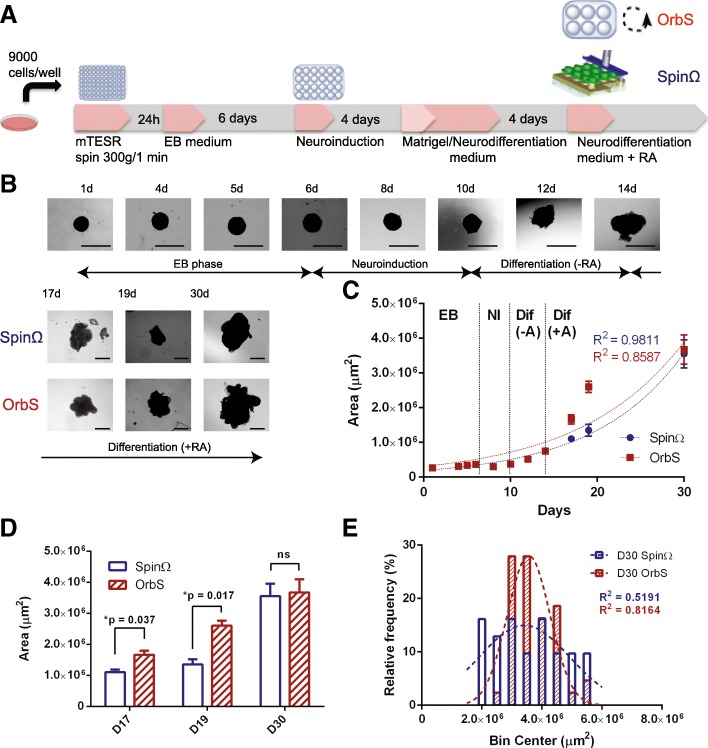
Growth curves, size distribution, and morphology obtained with the orbital shaker and SpinΩ bioreactor. **a** Workflow for brain organoid preparation. **b** Organoid morphology on selected days of the 30-day culture period, with arrows indicating the length of exposure to each medium condition. On day 14, organoids were divided into growth in the orbital shaker (red) and in the SpinΩ (blue). Scale bar = 1000 μm. **c**. Area growth curves (μm^2^) from day 1 to day 30, from *n* = 2 independent tests. Each test replicate contained at least 12 individual brain organoids. Lines represent exponential curve fit for the SpinΩ (blue) and orbital shaker (red), with correlation coefficients for each curve displayed on the graph. **d**. Comparison of area growth between the SpinΩ and shaker groups *p < 0.05. **e.** Histographic analysis of organoids grown in the SpinΩ and orbital shaker on day 30 (area in μm^2^) showing relative frequencies in terms of the percentage of automatically binned size. Data were pooled from two independent tests, *n* = 31 for the SpinΩ and *n* = 43 for the orbital shaker

To improve the first step of EB formation, we introduced some variations to the protocol published by Lancaster and Knoblich (2014) [9]. The experimental flow is depicted in Fig. 1a. Changes in the protocol include the addition of Rho-associated protein kinase inhibitor (ROCKi) for cell survival at cell dissociation [13] and post-plating centrifugation [14]. iPSCs cultivated in mTeSR1 medium derived from manual passages showed better EB formation compared with Ethylenediaminetetraacetic acid (EDTA)-passaged iPSCs (data not shown). Therefore, the cells were passaged manually before the EB formation step. Immediately after treatment for cell dissociation and before centrifugation, 10 μM ROCKi was added to the trituration solution. This step improved cell morphology after dissociation (Additional file 1: Figure S1a and b) [13]. The centrifugation step significantly improved the circularity of organoids on day 1 of growth (Additional file 1: Figure S1e), which was correlated with a significant increase in the observed areas of organoids in the two conditions (Additional file 1: Figure S1f). However, after 10 days, no significant difference was seen between specimens treated with and without centrifugation (Additional file 1: Figure S1 f), suggesting that this potentially negative effect was temporary. During the EB stage, no significant growth was observed (Fig.1a, b) and the morphology of aggregates did not change (Fig. 1b). Growth during the neuroinduction stage also was not significant (Fig. 1a, b). In the neuroinduction stage, protrusions of developing organoids started to expand; these continued to grow over time (Fig. 1b) and formed neuroepithelium-like tissue (see also Fig. 3b). The pattern of organoid growth resembled an exponential curve (Fig. 1c), with R square values of 0.9811 for the SpinΩ and 0.8587 for the orbital shaker. Growth in the orbital shaker was initially more rapid than that in the SpinΩ (Fig. 1d), but no significant difference was observed at the 30-day time point. A histogram analysis of organoids grown in the SpinΩ and the orbital shaker at 30 days showed that the size distribution of organoids grown on the shaker more closely resembled a Gaussian fit (Fig. 1e), suggesting more homogeneity in the shaker.

### The orbital shaker delivered higher velocity fields, but less shear stress, than the SpinΩ

**Fig. 2:**
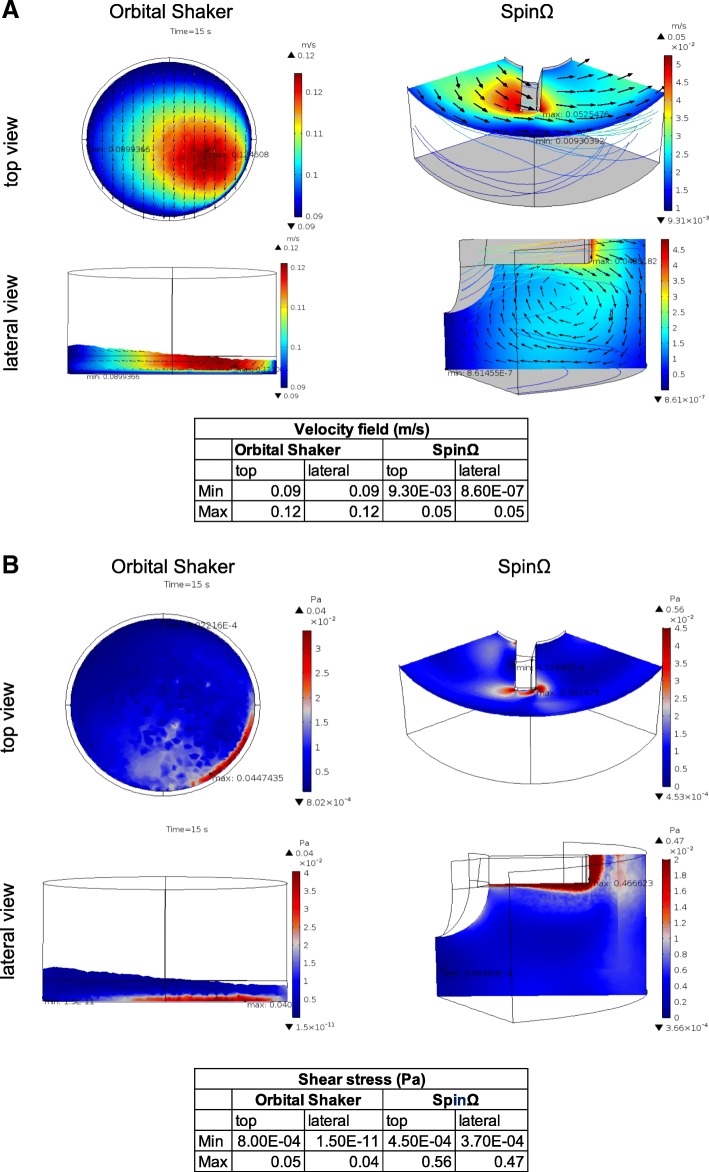
Fluid dynamic models in suspension culture. Computational fluid dynamic analysis of fluid velocity and shear stress for the SpinΩ and orbital shaker. **a** Velocity fields and **b** shear stress, top and lateral views for the SpinΩ and orbital shaker at 15 s after the start of movement. Minimum and maximum values are presented in the tables

Fluid dynamics in cultivation vessels has been shown to influence cell stemness, differentiation, and growth (for a review, see [15]). Here, we describe the fluid dynamic conditions to which organoids were subjected. In the plates on the orbital shaker, although some regions of the well were depleted of fluid at 0.5 s, just after the start of the plate movement, the fluid eventually covered the whole bottom surface of the well as the flow developed for the quasi-steady-state regime. The velocities were symmetrical at 14 and 15 s, due to the periodic flow created by the circular movement of the shaker (Additional file 2: Figure S2). Figure 2a presents the absolute velocity fields for the well on the orbital shaker at 15 s after the initiation of plate movement. The maximum absolute velocity reached with the stirrer plate during the quasi-steady-state regime was about 0.12 m/s. The shear stress field at 15 s is shown in Fig. 2b. The maximum stress was about 0.045 Pa in the regions of maximum-velocity gradients near the walls (see also Fig. 2a). The magnitude of the shear stress was about 10^− 2^ Pa in a large region of the fluid (Fig. 2)a.

The SpinΩ analysis is presented for different plane cuts. The velocity and shear stress fields are presented in Fig. 2a and b, respectively. The highest velocities occurred at the edge of the impeller, with values around 0.05 m/s (Fig. 2a). The velocities decreased with distance from the impeller and rotating shaft, being null at the well walls due to the no-slip conditions. In particular, lower velocities at the bottom of the well did not favor the mixture required for the enhanced growth of organoids. Our attempts to form aggregates from single cells in the SpinΩ created bodies with disparate sizes. Single cells accumulated in the low-speed area of the bioreactor and formed a large aggregate, while adjacent cells formed smaller bodies (Additional file 3: Figure S3). Large differences in the velocity fields could imply differences in nutrient mixing, which could in part explain the delayed growth in the SpinΩ on days 17–19 (Fig. 1d) and the wide area distribution shown on the histogram at day 30 for SpinΩ (Fig. 1e).

The maximum shear stress was 0.56 Pa at the edge of the impeller due to the large velocity gradients in this region (Fig. 2b). Shear stress in the bulk fluid was smaller, with magnitudes on the order of 10^− 3^ to 10^− 2^ Pa.

Velocity gradients and shear stress are correlated parameters. In this study, however, the absolute velocity magnitudes were larger for the orbital shaker than for the SpinΩ, whereas shear stress values had the opposite pattern. These results can be explained by the large differences in velocity values at the region around the SpinΩ impeller and the rotating shaft increasing the shear of fluid in this area.

The gold standard for organoid protocols, a spinning bioreactor, has been reported to sustain organoid growth in culture for more than 8 months [2, 9]. Comparison with previous literature on the CFD of a spinning bioreactor [10] showed that shear stresses of the steering plates found by us are of the same order of magnitude as those reported for the spinner bioreactor [10]. Maximum shear stress values for the spinner were 0.028 Pa at 40 rpm and 0.047 Pa at 75 rpm [10] (Additional file 4: Table S2), and a maximum value of 0.045 Pa at 90 rpm was predicted for the orbital shaker (Additional file 4: Table S2). The maximum shear stress of the SpinΩ (0.56 Pa) was one order of magnitude greater.

The velocity fields of the steering plates (maximum, 0.12 m/s) were of the same order of magnitude as those of the spinner bioreactor (maximum, 0.277 m/s), whereas those of the SpinΩ were lower (0.05 m/s) (Additional file 4: Table S2).

Computational simulations suggest that the use of the stirrer plate was more suitable for the growth of organoids than was the use of the bioreactor. First, the SpinΩ has regions of low velocity at the bottom of the well, where fluid mixing is poor and particles deposition is likely to happen. Second, shear stresses are smaller in the orbital shaker, which could be better for the preservation of organoid structures in long-term culture.

### The SpinΩ reactor and orbital shaker derived structured organoids

**Fig. 3:**
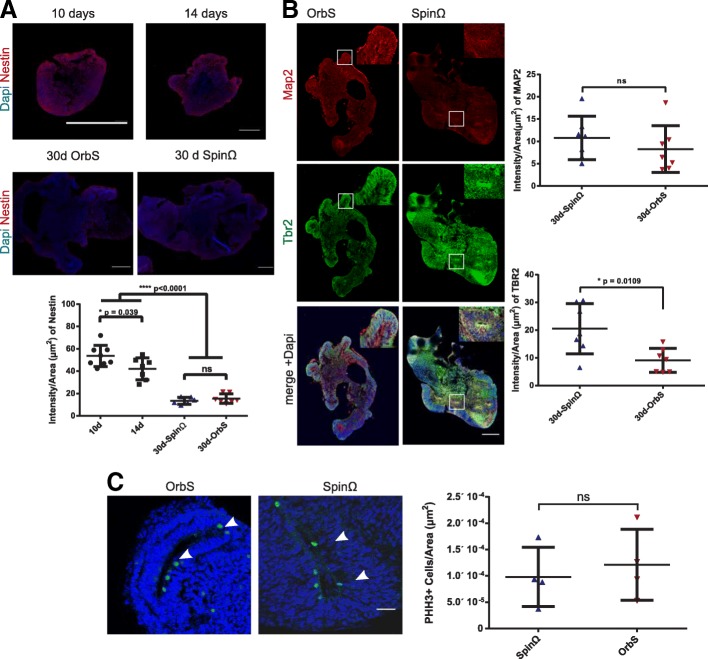
Neurogenesis in the early stages of organoid development. **a** Top, representative nestin immunostaining of organoids after 10, 14, and 30 days in culture. Bars = 500 μm. Bottom, quantification of intensity of nestin staining normalized with area of organoid for 10, 14 and 30 days in culture for the different conditions. *N* = 8, 7, 7 and 6, respectively. **b** Left, representative MAP2 and TBR2 staining of 30-day-old organoids. Bars = 500 μm. Right, quantification of intensity of MAP2 and TBR2 staining. *N* = 7 organoids for each condition. **c** Left, representative phospho-histone H3 staining of ventricular-like regions of 30-day-old organoids. Bars = 100 μm. and right, corresponding quantification. *N* = 4 for each condition. For all staining, organoids were collected from 2 independent experiments. Mean + SD for all quantifications

We examined the maturation of organoids with a focus on the transition from predominantly neuroprogenitor stem cells to the development of neuroepithelial regions. Nestin staining of neural stem cells, performed at 10, 14, and 30 days of culture, showed similar decreases over time for the orbital shaker and SpinΩ treatments (Fig. 3a). These results are consistent with the start of differentiation of progenitor cells into neurons. At 30 days, the organoids had developed ventricular-like regions and neuroepithelium-like structures that were positive for MAP2 and TBR2 (Fig. 3b). MAP2 staining levels were similar in organoids cultivated in the orbital shaker and SpinΩ, but TBR2 staining levels were significantly stronger than those cultivated in the SpinΩ. As TBR2 labeled neuron progenitors in sub-ventricular zones, we examined whether cell proliferation was increased under our culture conditions through phospho-histone-3 staining on day 30. However, no difference in the number of proliferating cells was detected between the two conditions (Fig. 3c).

### Organoids generated in suspension cultures presented markers for distinct brain regions

**Fig. 4:**
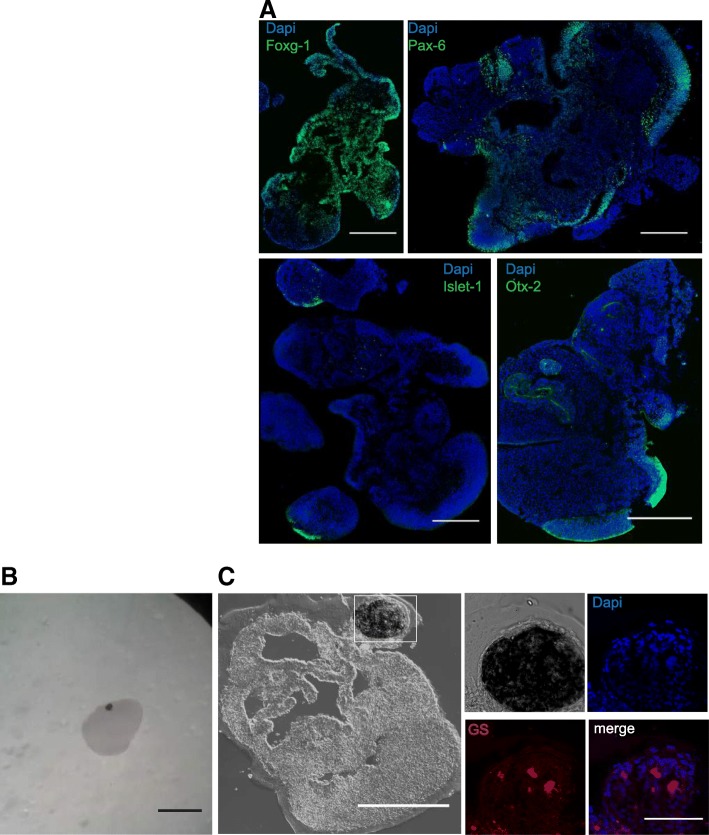
Cell types and brain regions represented in early brain organoid development. **a** Immunostaining of 30-day-old organoids grown in the orbital shaker for FOXG-1, PAX-6, islet-1, and OTX-2. Bars = 500 μm; **b** Stereoscopic image of an organoid with pigmented regions. Bar = 1 mm. **c** Pigmented regions (box) of organoids after 45 days in culture. Bar = 1 mm. The pigmented regions (box) were positive for glycogen synthetase. Bar = 500 μm

Organoids grown in the SpinΩ and orbital shaker displayed very similar morphology and developmental profile. We decided to focus on organoids grown on the orbital plates to provide further characterization of the organoid generation pipeline, because the organoids grown in SpinΩ have been described elsewhere [12]. We observed that 30-day organoids from orbital shaker cultures were positive for FOXG-1 (forebrain), PAX-6 (dorsal telencephalon), OTX-2 (retinal cells and midbrain), and Islet-1 (hindbrain; Fig. 4a) showing diversification and development consistent with previous reports [2]. We observed that, at 45 days, the organoids had pigmented regions (Fig. 4b, c), which were previously described to reproduce the formation of retinal pigmented epithelium [2]. The pigmented regions were positive for the retinal cell marker glycogen synthetase (GS) (Fig. 4c).

Proteomic analysis of organoids grown for 30 days led to the identification of 4099 proteins (Additional file 5: dataset). The 100 most abundant proteins (ordered by peptide spectral match) were analyzed by gene enrichment; 86 matched DAVID IDs [16, 17]. Gene enrichment analysis revealed that the majority (76.6%) of the identified proteins matched brain entries with the high *p*-value. Proteins identified in our analysis are markers of the forebrain (BCL11B, DBI, CLU, SPARC), midbrain (OTX-2), and hindbrain (HOXA1). Retinal cell proteins were also identified: a general marker (GS) and those for the Muller glia (DKK3), photoreceptors (RCVRN), and retinal ganglion cells (NEFL) (Additional file 5: dataset). The appearance of these proteins at 30 days preceded the formation of the pigmented regions which were observed later, at 45 days (Fig. 4c). Additionally, the synaptic proteins synaptotagmin, synaptobrevin, synaptojanin, GABA receptor and a set of voltage-dependent channels were detected in the proteomic analysis (Additional file 5: dataset), revealing the potential of these organoids to form synapses in cultures conditions.

## Discussion

Brain organoids present cytoarchitecture that recapitulates brain tissue organization, offering a complex in vitro model for the study of brain normal and pathological development [5, 18, 19]. Although brain organoid cultivation presents challenges related to the lack of reproducibility and scalability, we achieved high reproducibility of early-stage organoid size and growth by adding steps to a standard protocol [9]. This included the use of higher concentrations of ROCKi and plate centrifugation in the EB formation step, which have been previously demonstrated to improve EB formation but have never been applied to grow organoids [13, 14].

Scalability was achieved by applying two multiplex platforms: steering plates on an orbital shaker and the SpinΩ bioreactor [12]. The SpinΩ was 3D printed according to the blueprints provided by Qian et al. [12]. We encountered the following issues with SpinΩ use: 1) manual handling, as medium changes involved disassembly of a combination of pieces; 2) the need for sterilization for consecutive use; and 3) the maintenance of sterile conditions, as the equipment has 12 gears that could not be cleaned properly during the course of the experiment. These issues make the SpinΩ dependent on user skills, rendering it more prone to error and susceptible to contamination over the long timeframe of brain organoid cultivation (up to 8 months) [2, 9], when compared with the use of steering plates on an orbital shaker.

We suggest that the lower velocities of the SpinΩ, may affect nutrient mixing, which could explain the decreased organoid growth seen on days 17 and 19, and the wide size distribution of organoids observed on day 30. Overall, our CFD analysis indicated that the fluid dynamic variables of the steering plate on the orbital shaker are closer to those of the spinner bioreactor. Therefore, this method should be preferentially selected as a multiplex alternative to the use of a spinner bioreactor.

The appearance of diverse brain regions and pigmented regions labeled with the retinal epithelium marker GS has been previously described [2] and related to a regional differentiation in organoids. Organoids produced using our protocol presented pigmented regions positive for GS suggesting that the technique described here may be appropriate for studies involving the complexity of early brain development.

In addition, proteomic analysis confirmed the organoids (produced in this study) show a protein profile that is compatible with several differentiated brain regions. Altogether, those results corroborate that the new proposed protocol opens a new window, allowing the exploration, with multiple analyses, of important biomarkers of the morphological, genetic and molecular complexity of the human brain development under normal and abnormal conditions.

## Conclusion

We conclude that the use of an orbital shaker with an improved organoid preparation protocol successfully generates brain organoids with significant reliability across different iPSC lines. Use of CFD analysis indicates that use of an orbital shaker offers advantages to the SpinΩ multiplex platform and produces morphological and molecular complex human brain organoids.

### Experimental procedures

#### Pluripotent stem cell culture

The human induced pluripotent stem cells (iPSCs) used in this work are described in Additional file 4: Table S1. GM23279 cell line from the NIGMS Human Genetic Cell Repository was obtained and certified by the Coriell cell repository; the remaining cell lines were generated in-house. The iPSCs were maintained in six-well plates coated with Geltrex in mTeSR® medium (StemCell Technologies, Canada). Cells were either passaged manually or with 0.15 mM EDTA through passage 48.

#### Culture of brain organoids

The method used to produce cerebral organoids was based on a previously published protocol [9]. Briefly, iPSC colonies grown in six-well plates were dissociated with 1 ml Accutase Cell Detachment Solution (MPBio, USA) for 4 min at 37 °C, and 1 ml phosphate-buffered saline (PBS; LGC Biotechnology, USA) was then added. The resulting solution was transferred to a 15-ml conical tube, and 20 μl 10 mM Rho-kinase inhibitor (ROCKi, Y27632; Merck Millipore, USA) was added before centrifugation [13], to obtain a final concentration of 10 μM. Cells were counted in a hemocytometer and centrifuged at 300×*g* for 4 min. Cells were plated in hESC medium containing 50 μM ROCKi and 4 ng/ml b-FGF. hESC medium contained 20% knockout serum replacement (Life Technologies), 3% ESC-quality fetal bovine serum (Thermo Fisher Scientific, USA), 1% GlutaMAX (Life Technologies, Canada), 1% minimum essential medium non-essential amino acids (MEM-NEAAs; Life Technologies), 0.7% 2-mercapto-ethanol, and 1% penicillin-streptomycin (P/S; Life Technologies), as described in Lancaster and Knoblich (2014) [9]. We used 9000 cells/well of a 96-well plate, which has been demonstrated to lead to efficient organoid formation under the cultivation conditions described [20]. The 96-well plates were centrifuged for 1 min at 300×*g* to improve initial EB aggregation [14] (Additional file 1: Figure S1b).

After day 1, embryoid bodies (EBs) were cultured as described by Lancaster and Knoblich (2014) [9]. The medium was changed every 48 h after plating for 6 days. On day 6, EBs were transferred to 24-well ultralow-attachment culture plates (one/well) containing 0.5 ml neuroinduction medium [1% N_2_ supplement (Gibco), 1% GlutaMAX (Life Technologies), 1% MEM-NEAAs, 1% P/S, and 1 μg/ml heparin in DMEM/F12 (Life Technologies). After 4 days (day 10), organoids were coated with Matrigel similarly as described by Sartore et al. (2017) [21] in a 60-mm non-adherent tissue culture plate; six organoids were placed in 3 ml diluted Matrigel and incubated for 1 h at 37 °C under 5% CO_2_. The coated organoids were then returned to the 24-well ultra-low-attachment plates with 0.5 ml neurodifferentiation medium with no vitamin A (50% neurobasal medium, 0.5% N_2_, 1% B_27_ supplement without vitamin A, 1:100 2-mercapto-ethanol, 0.5% MEM-NEAA, 1% GlutaMAX, and 1:100 P/S in DMEM/F12) and left for 4 days in static culture. Subsequently, cerebral organoids were grown in suspension using two different platforms: 1) steering plates on a standard orbital shaker (six-well culture plates), agitated at 90 rpm [as proposed by Lancaster and Knoblich (2014) [9]]; and 2) SpinΩ system developed by Qian et al. [12], which was 3D printed by the company DelthaThinkers using the blueprints provided in the manuscript and coupled to 12-well culture plates, agitated at 60 rpm. In both cases, 10 organoids were placed in 3 ml neurodifferentiation medium with vitamin A (day 14). The medium was changed weekly until day 60 of culture. They were imaged with an EVOS cell imaging system (Thermo Fisher Scientific) in brightfield. The area, diameter, and circularity of individual cerebral organoids were quantified using a custom macro in ImageJ.

#### Computational fluid dynamics simulation

CFD simulations were performed for the flows imposed by the SpinΩ impeller and the orbital shaker using the finite element commercial code COMSOL Multiphysics® and using the geometry and finite element mesh as described on Additional file 6: Figure S4. The methods used in the analysis are described in the supplementary information section and in [22].

#### Histology and immunofluorescence

Cerebral organoids were fixed in 4% paraformaldehyde, incubated sequentially in sucrose solutions (10, 20, and 30%) prepared in PBS, embedded in optimal cutting temperature compound, and frozen in liquid nitrogen. The organoids were sectioned (20-μm thickness) with a cryostat (Leica Biosystems, Germany). Immunofluorescence was performed using the following primary antibodies: rabbit anti-nestin (RA22125, 1:500; Neuromics, USA), rabbit anti-PAX6 (42–6600, 1:100; Thermofisher Scientific), rabbit anti-TBR2 (AB2283,1:200; Millipore), mouse anti-MAP2 (M1406, 1:300; Sigma-Aldrich, USA), rabbit anti-FOXG1 (ab18259, 1:1000; Abcam, UK), rabbit anti-islet-1 (ab20670; 1:1000; Abcam), rabbit anti–OTX-2 (ab21990, 1:200; Abcam), mouse anti-glycogen synthetase (610,518, 1:500; BD), and rabbit anti-PH3 (06–570, 1:500; Millipore). The following secondary antibodies were used: Alexa Fluor 488 goat anti-mouse (A11001, 1:500; Invitrogen, Canada) and Alexa Fluor 546 goat anti-rabbit (A11010, 1:500; Invitrogen). 4′,6-Diamidino-2-phenylindole (1 mg/ml) was used for nucleus staining. Images were acquired using a Leica TCS SP8 confocal microscope.

The specificity of the immunofluorescence staining was controlled with a negative secondary antibody control, which consisted in incubating the slices with secondary antibody in the absence of primary antibody.

### Proteomic analysis

Two independent pools of four organoids each were used in the experiments. Protein digestion, peptide fractionation, mass spectrometric analysis, and raw data processing was performed as described by Murillo et al. (2017) [23]. Gene enrichment analysis was performed using the DAVID Bioinformatics Database (https://david.ncifcrf.gov/summary.jsp).

### Statistical analysis

Statistical testing was performed using two-tailed t-test with GraphPad Prism 6 software. Statistical significance was defined as *p* < 0.05 unless otherwise stated in figure legends. Correlation analysis was done comparing the R square of a non-linear fit (Exponential fit in Fig. 1c and a Gaussian fit in Fig. 1e) for the two conditions, SpinΩ and orbital shaker.

## Additional files

Additional file 1: **Figure S1.** Effect of ROCKi treatment and centrifugation at EB formation step**.** A. Changes in cell morphology were observed during cell counting. 10 μM iROCK treatment at dissociation step preserves cell membrane smoothness and prevents blebbing. Arrowheads: cell membranes. B. Effect of centrifugation on cell aggregation. Bars: 2000 μm. C. Measured circularity based on organoid morphology with and without centrifugation step; *n* = 8 for one independent test for the condition without centrifugation and n > =12 for two independent tests for the condition with centrifugation. D. Comparison of area of organoids with and without centrifugation at day 1 and day 10. (PDF 10252 kb)

Additional file 2: **Figure S2.** CFD analysis of plates in an orbital shaker. Transient states were simulated until a quasi-steady-state regime was reached, when the flow became periodic. Liquid flow was analyzed at 0.5, 14 and 15 s after the start of the movement. (PDF 1067 kb)

Additional file 3: **Figure S3.** Incubation of single cells in the SpinΩ demonstrate the cell aggregation at low-speed areas. Dissociated neural stem cells from GM23279A line were incubated in the SpinΩ. Large aggregates were observed after 3 days in culture. Bars: 1000 μm. (PDF 1481 kb)

Additional file 4: **Table S1.** Cell lines used to generate brain organoids. **Table S2**: comparison of CFD analysis from SpinΩ, Orbital Shaker and Spinner. Supplementary methods: description of computational Fluid Dynamics Simulation. (PDF 383 kb)

Additional file 5: Dataset proteomic analysis of a 45 day organoid. List of identified proteins with the respective accession number, description, number of peptides identified per protein, number of peptides spectral matches (PSM), unique peptides identified and protein characteristics: number of amino acids (# AAs), molecular weight (MW [kDa]) and calculated isoelectric point (calc. pI). (PDF 629 kb)

Additional file 6: **Figure S4.** Geometries and finite element meshes used in the CFD simulations. A. Geometry for the bioreactor. B. Mesh used for the bioreactor containing 390,000 finite elements. C. Geometry for the well on the stirrer plate. D. Mesh for the well on the stirrer plate containing 125,000 finite elements. (PDF 422 kb)

## Abbreviations

3D: Three-dimensional
BCL11B: B cell leukemia/lymphoma 11B
CFD: Computational fluid dynamics
CLU: Clusterin
DBI: Diazepam binding inhibitors
DKK3: Dickkopf-related protein 3
EB: Embryoid body
EDTA: Ethylenediaminetetraacetic acid
FOXG1: Forkhead box G1
GABA: Gamma-Aminobutyric Acid
GS: Glycogen synthetase
HOXA1: Homeobox A1
hPSC: Human pluripotent stem cells
MAP 2: Microtubule associated protein 2
NEFL: Neurofilament light
OTX-2: Orthodenticle homeobox 2
Pa: Pascal
PAX-6: Paired box 6
RCVRN: Recoverin
ROCKi: Rho-Associated protein kinase inhibitor
SPARC: Secreted acidic cysteine rich glycoprotein
TBR2: T-box brain gene 2
