# Supplementary figures and images for: Computational fluid dynamic analysis of physical forces playing a role in brain organoid cultures in two different multiplex platforms
Source: BMC Dev Biol. 2019 Mar 7;19:3. doi: 10.1186/s12861-019-0183-y (PMC6404276; doi:10.1186/s12861-019-0183-y)

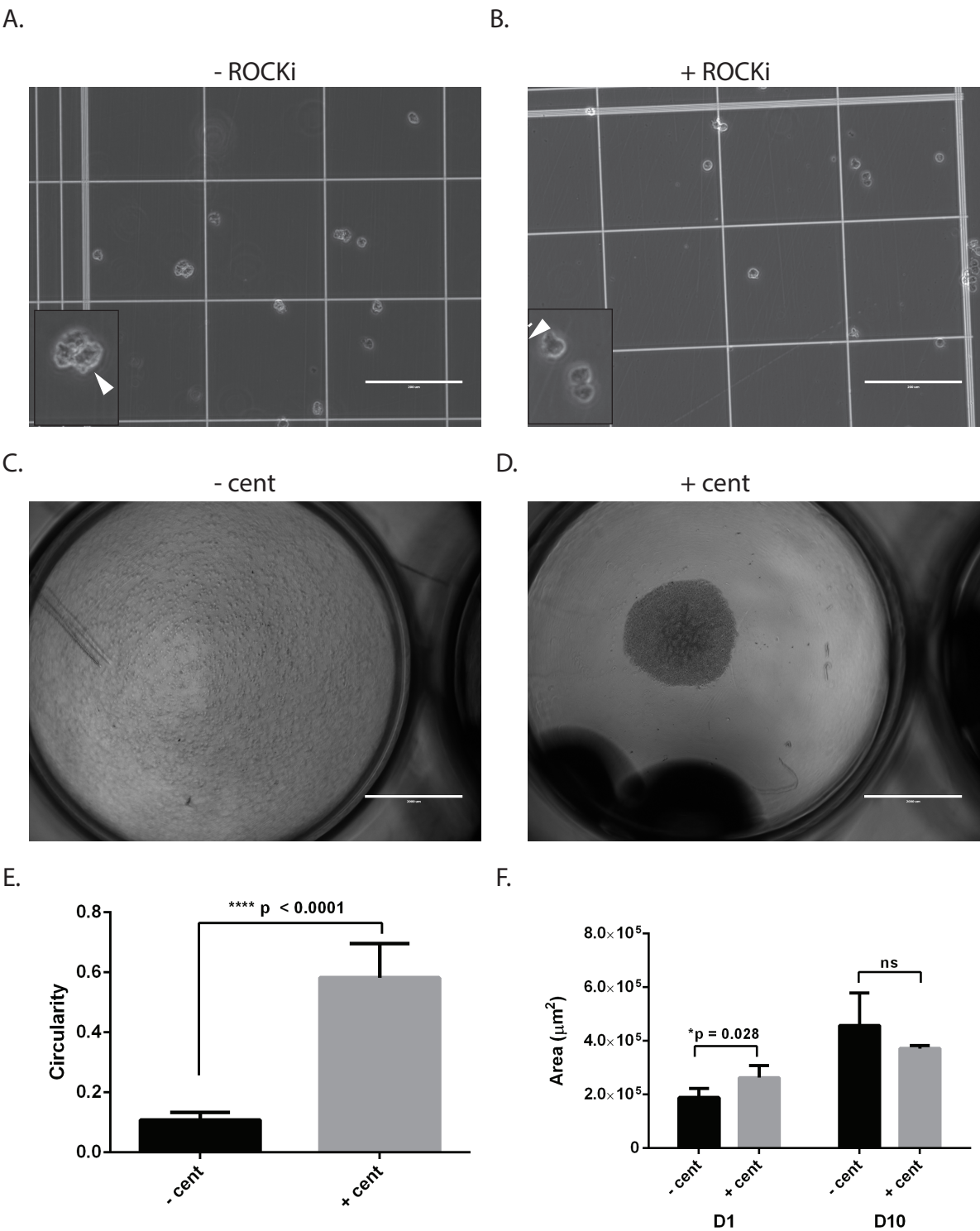

Supplement: Supplementary file 1 — Figure S1. Effect of ROCKi treatment and centrifugation at EB formation step. A. Changes in cell morphology were observed during cell counting. 10 μM iROCK treatment at dissociation step preserves cell membrane smoothness and prevents blebbing. Arrowheads: cell membranes. B. Effect of centrifugation on cell aggregation. Bars: 2000 μm. C. Measured circularity based on organoid morphology with and without centrifugation step; n = 8 for one independent test for the condition without centrifugation and n > =12 for two independent tests for the condition with centrifugation. D. Comparison of area of organoids with and without centrifugation at day 1 and day 10. (PDF 10252 kb) [file 12861_2019_183_MOESM1_ESM.pdf]

Orbital shaker

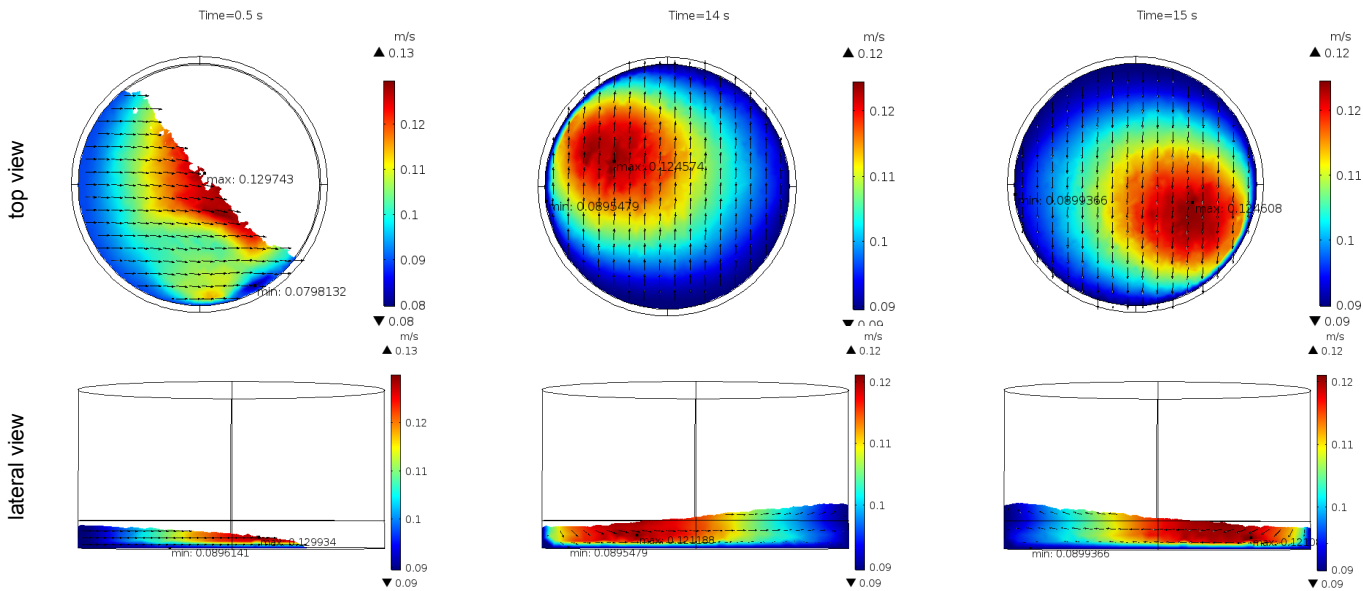

Supplement: Supplementary file 2 — Figure S2. CFD analysis of plates in an orbital shaker. Transient states were simulated until a quasi-steady-state regime was reached, when the flow became periodic. Liquid flow was analyzed at 0.5, 14 and 15 s after the start of the movement. (PDF 1067 kb) [file 12861_2019_183_MOESM2_ESM.pdf]

Supplementary Figure 3

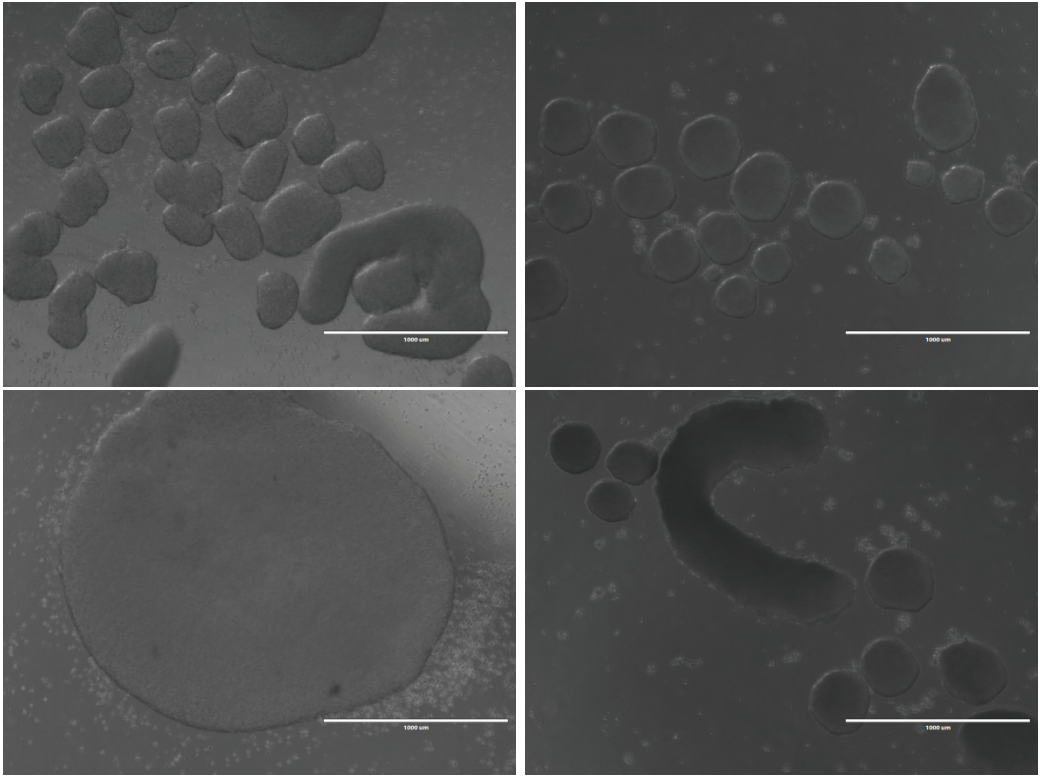

Supplement: Supplementary file 3 — Figure S3. Incubation of single cells in the SpinΩ demonstrate the cell aggregation at low-speed areas. Dissociated neural stem cells from GM23279A line were incubated in the SpinΩ. Large aggregates were observed after 3 days in culture. Bars: 1000 μm. (PDF 1481 kb) [file 12861_2019_183_MOESM3_ESM.pdf]

Supplementary Figure 4

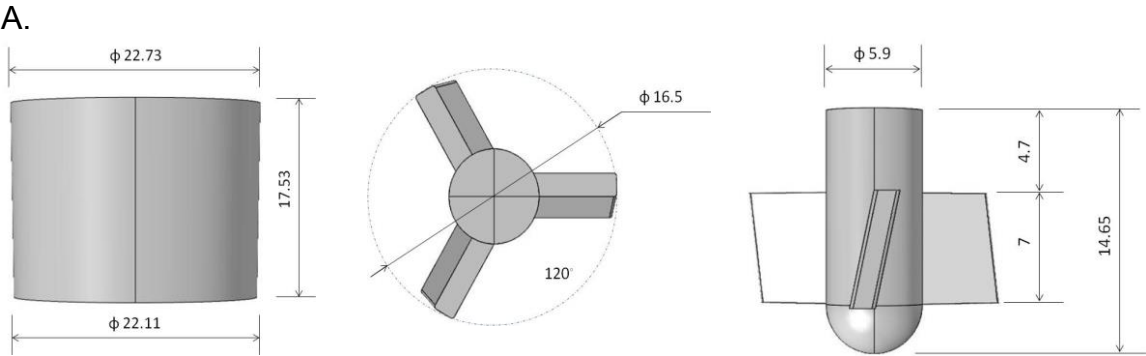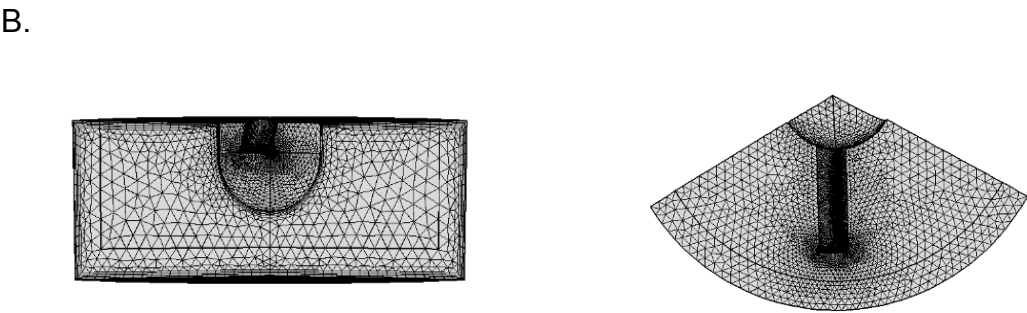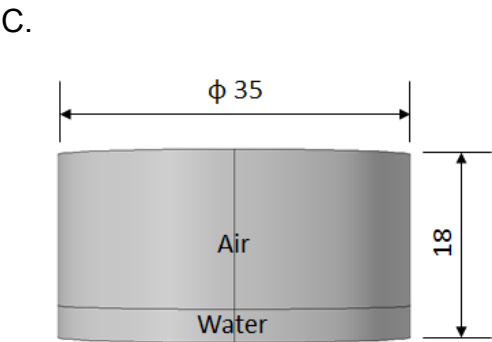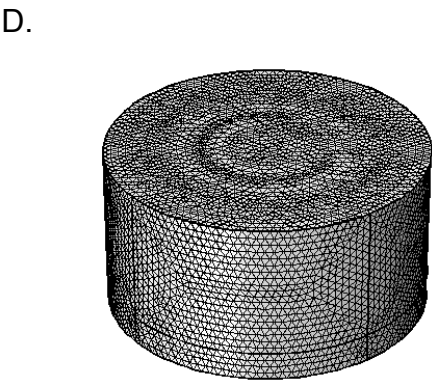

Supplement: Supplementary file 6 — Figure S4. Geometries and finite element meshes used in the CFD simulations. A. Geometry for the bioreactor. B. Mesh used for the bioreactor containing 390,000 finite elements. C. Geometry for the well on the stirrer plate. D. Mesh for the well on the stirrer plate containing 125,000 finite elements. (PDF 422 kb) [file 12861_2019_183_MOESM6_ESM.pdf]
