# Supplementary material for: Computational fluid dynamic analysis of physical forces playing a role in brain organoid cultures in two different multiplex platforms
Source: BMC Dev Biol. 2019 Mar 7;19:3. doi: 10.1186/s12861-019-0183-y (PMC6404276; doi:10.1186/s12861-019-0183-y)
Supplement: Supplementary file 4 — Table S1. Cell lines used to generate brain organoids. Table S2: comparison of CFD analysis from SpinΩ, Orbital Shaker and Spinner. Supplementary methods: description of computational Fluid Dynamics Simulation. (PDF 383 kb) [file 12861_2019_183_MOESM4_ESM.pdf]

Supp. Table 1: Cell lines used to generate brain organoids

	Cell line name	Disease	Passage n.	Reference
1	GM23279A	No	39-44	NIGMS human genetic cell repository
2	ADHD4	1	16-18	Sochacki et al. 2016
3	ADHD24	1	16-18	Sochacki et al. 2016
4	DCC1ctl	No	10-21	unpublished
5	DCC4ctl	No	17	unpublished
6	DCC2	2	20-30	unpublished
7	DCC5	2	5-13	unpublished

1- Attention Deficit Hyperactivity Disorder
2- Corpus callosum agenesis

Supp. Table 2

	Velocity field (max) [m/s]	Shear stress (max) [Pa]	Reference
**SpinΩ**	0.050	0.560	-
**Orbital Shaker**	0.120	0.045	-
**Spinner**	0.178	0.150	(Wang et al. *Stem Cell Res*. (2013)

Supplementary Information - Methods

Computational Fluid Dynamics Simulation

Computational Fluid Dynamics (CFD) simulations were performed for the flows either imposed by the impeller in the Spin Ω or by the orbital shaker, by using the finite element commercial code COMSOL Multiphysics®. For the SpinΩ the rotating speed used was 60 rpm and for the orbital shaker, the rotating speed was 90 rpm, with a radius of 9.5 mm. Geometry and the finite element mesh used in the simulations involved 3 ml of the suspension culture media for both mixing techniques (Supp. Fig 4). The finite element meshes used for the bioreactor and stirrer plate flow simulations contained 390,000 and 125,000 elements, respectively. The numbers of finite elements and time steps used in the simulations were selected after grid refinement analyses; the differences between the velocity components computed with the meshes used in this work and with less refined meshes were smaller than 3%, at selected points in the fluid domains. The mesh used for the well on the stirrer plate was practically uniform, however, regions of large gradients may occur at different positions during the periodic movement that is externally imposed by the plate (see figure 2D). For the bioreactor, regions of largest gradients occur at the edge of the impeller and on the surface of the rotating shaft. In these areas, the mesh was refined (see figure 2B). The simulations were performed with the κ-ω turbulence model for the SpinΩ. A laminar two-phase model was used for the steering plates on the orbital shaker, which was validated with the experimental results of Salek et al. (Salek et al., 2012).
