# Supplementary material for: Computational fluid dynamic analysis of physical forces playing a role in brain organoid cultures in two different multiplex platforms
Source: BMC Dev Biol. 2019 Mar 7;19:3. doi: 10.1186/s12861-019-0183-y (PMC6404276; doi:10.1186/s12861-019-0183-y)
Supplement: Supplementary file 5 — Dataset proteomic analysis of a 45 day organoid. List of identified proteins with the respective accession number, description, number of peptides identified per protein, number of peptides spectral matches (PSM), unique peptides identified and protein characteristics: number of amino acids (# AAs), molecular weight (MW [kDa]) and calculated isoelectric point (calc. pI). (PDF 629 kb) [file 12861_2019_183_MOESM5_ESM.pdf]

Accession	supp. Dataset<br>Description	# Peptides	# PSMs	# Unique Peptides	# AAs	MW [kDa]	calc. pI
Q71U36	Tubulin alpha-1A chain , GN=TUBA1A	19	937	2	451	50.104	5.06
Q9BVA1	Tubulin beta-2B chain , GN=TUBB2B	22	861	2	445	49.921	4.89
P07437	Tubulin beta chain , GN=TUBB	21	855	4	444	49.639	4.89
Q13885	Tubulin beta-2A chain , GN=TUBB2A	21	840	1	445	49.875	4.89
P68363	Tubulin alpha-1B chain , GN=TUBA1B	19	850	2	451	50.12	5.06
P68371	Tubulin beta-4B chain , GN=TUBB4B	21	791	1	445	49.799	4.89
P04350	Tubulin beta-4A chain , GN=TUBB4A	20	590	3	444	49.554	4.88
Q13509	Tubulin beta-3 chain , GN=TUBB3	20	620	6	450	50.4	4.93
P06733	Alpha-enolase , GN=ENO1	21	544	19	434	47.139	7.39
P68104	Elongation factor 1-alpha 1 , GN=EEF1A1	12	420	8	462	50.109	9.01
P14618	Pyruvate kinase PKM , GN=PKM	28	380	4	531	57.9	7.84
P46821	Microtubule-associated protein 1B ,	53	364	52	2468	270.468	4.81
P08670	Vimentin , GN=VIM	31	374	26	466	53.619	5.12
P68032	Actin, alpha cardiac muscle 1 , GN=ACTC1	7	390	2	377	41.992	5.39
P22314	Ubiquitin-like modifier-activating enzyme 1 , GN=UBA1	29	327	29	1058	117.774	5.76
P07900-2	Isoform 2 of Heat shock protein HSP 90-alpha , GN=HSP90AA1	35	357	20	854	98.099	5.16
P14618-2	Isoform M1 of Pyruvate kinase PKM ,	25	322	1	531	58.025	7.71
P06576	ATP synthase subunit beta, mitochondrial , GN=ATP5B	19	331	19	529	56.525	5.4
Q14204	Cytoplasmic dynein 1 heavy chain 1 , GN=DYNC1H1	85	314	85	4646	532.072	6.4
P52272	Heterogeneous nuclear ribonucleoprotein M , GN=HNRNPM	23	350	23	730	77.464	8.7
P11142	Heat shock cognate 71 kDa protein ,	24	335	21	646	70.854	5.52
P11021	78 kDa glucose-regulated protein , GN=HSPA5	31	330	29	654	72.288	5.16
P04406	Glyceraldehyde-3-phosphate dehydrogenase , GN=GAPDH	16	292	16	335	36.03	8.46
P12277	Creatine kinase B-type , GN=CKB	13	288	13	381	42.617	5.59
P35580-4	Isoform 4 of Myosin-10 , GN=MYH10	59	258	55	2007	232.385	5.55
P09104	Gamma-enolase , GN=ENO2	13	266	12	434	47.239	5.03
Q562R1	Beta-actin-like protein 2 , GN=ACTBL2	6	292	4	376	41.976	5.59
P08238	Heat shock protein HSP 90-beta ,	31	296	14	724	83.212	5.03
Q00610	Clathrin heavy chain 1 , GN=CLTC	38	238	38	1675	191.493	5.69
Q9BUF5	Tubulin beta-6 chain , GN=TUBB6	16	253	6	446	49.825	4.88
P49327	Fatty acid synthase , GN=FASN	42	217	42	2511	273.254	6.44
P48681	Nestin , GN=NES	39	225	39	1621	177.332	4.36
P60028	Rab GDP dissociation inhibitor alpha OS=Pan troglodytes GN=GDI1 PE=2	17	193	13	447	50.55	5.14
P10809	60 kDa heat shock protein, mitochondrial , GN=HSPD1	24	207	24	573	61.016	5.87
P14625	Endoplasmin , GN=HSP90B1	29	203	27	803	92.411	4.84
Q13813-2	Isoform 2 of Spectrin alpha chain, non-erythrocytic 1 , GN=SPTAN1	68	201	68	2477	284.919	5.35
Q5QNW6-2	Isoform 2 of Histone H2B type 2-F , GN=HIST2H2BF	2	195	1	134	14.832	10.35
P06899	Histone H2B type 1-J , GN=HIST1H2BJ	2	193	1	126	13.896	10.32
P06748	Nucleophosmin , GN=NPM1	6	174	6	294	32.555	4.78
P07910-2	Isoform C1 of Heterogeneous nuclear ribonucleoproteins C1/C2 , GN=HNRNPC	9	223	5	293	32.318	5.08
P55072	Transitional endoplasmic reticulum ATPase , GN=VCP	24	199	24	806	89.266	5.26
P62258	14-3-3 protein epsilon , GN=YWHAE	15	197	14	255	29.155	4.74
P0DMV9	Heat shock 70 kDa protein 1B , GN=HSPA1B	19	198	17	641	70.009	5.66
P50395	Rab GDP dissociation inhibitor beta ,	17	171	13	445	50.631	6.47
P35579	Myosin-9 , GN=MYH9	44	173	39	1960	226.392	5.6
P21333	Filamin-A , GN=FLNA	38	176	36	2647	280.564	6.06
Q05639	Elongation factor 1-alpha 2 , GN=EEF1A2	6	173	2	463	50.438	9.03
P13639	Elongation factor 2 , GN=EEF2	27	155	27	858	95.277	6.83
P13929	Beta-enolase , GN=ENO3	6	159	4	434	46.957	7.71
P25705	ATP synthase subunit alpha, mitochondrial , GN=ATP5A1	17	164	17	553	59.714	9.13
P63104	14-3-3 protein zeta/delta , GN=YWHAZ	14	158	11	245	27.728	4.79
Q9Y4L1	Hypoxia up-regulated protein 1 , GN=HYOU1	20	147	20	999	111.266	5.22

P09651	Heterogeneous nuclear ribonucleoprotein A1 , GN=HNRNPA1	13	156	11	372	38.723	9.13
P20700	Lamin-B1 , GN=LMNB1	22	149	22	586	66.368	5.16
P61978-2	Isoform 2 of Heterogeneous nuclear ribonucleoprotein K , GN=HNRNPK	16	155	1	464	50.996	5.33
P61978-3	Isoform 3 of Heterogeneous nuclear ribonucleoprotein K , GN=HNRNPK	16	154	1	440	48.532	5.54
P31943	Heterogeneous nuclear ribonucleoprotein H , GN=HNRNPH1	10	143	6	449	49.198	6.3
P09211	Glutathione S-transferase P , GN=GSTP1	8	128	8	210	23.341	5.64
P19338	Nucleolin , GN=NCL	15	138	15	710	76.568	4.7
Q16555	Dihydropyrimidinase-related protein 2 , GN=DPYSL2	18	137	16	572	62.255	6.38
Q15084-2	Isoform 2 of Protein disulfide-isomerase A6 , GN=PDIA6	16	144	16	492	53.867	5.33
P00558	Phosphoglycerate kinase 1 , GN=PGK1	17	136	17	417	44.586	8.1
Q01082	Spectrin beta chain, non-erythrocytic 1 , GN=SPTBN1	46	134	4	2364	274.439	5.57
P43243	Matrin-3 , GN=MATR3	18	133	18	847	94.565	6.25
Q14195-2	Isoform LCRMP-4 of Dihydropyrimidinase-related protein 3 , GN=DPYSL3	22	135	20	684	73.865	6.35
P30101	Protein disulfide-isomerase A3 , GN=PDIA3	23	136	23	505	56.747	6.35
P13010	X-ray repair cross-complementing protein 5 , GN=XRCC5	13	124	13	732	82.652	5.81
P23246	Splicing factor, proline- and glutamine-rich , GN=SFPQ	16	134	16	707	76.102	9.44
P26599-3	Isoform 3 of Polypyrimidine tract-binding protein 1 , GN=PTBP1	10	125	10	557	59.596	9.16
Q09666	Neuroblast differentiation-associated protein AHNAK , GN=AHNAK	73	148	73	5890	628.699	6.15
O75874	Isocitrate dehydrogenase [NADP] cytoplasmic , GN=IDH1	14	145	14	414	46.63	7.01
P50454	Serpin H1 , GN=SERPINH1	18	136	18	418	46.411	8.69
P78527	DNA-dependent protein kinase catalytic subunit , GN=PRKDC	45	138	45	4128	468.788	7.12
P08133	Annexin A6 , GN=ANXA6	23	130	23	673	75.826	5.6
Q14980	Nuclear mitotic apparatus protein 1 , GN=NUMA1	41	123	41	2115	238.115	5.78
Q01082-3	Isoform 2 of Spectrin beta chain, non-erythrocytic 1 , GN=SPTBN1	43	118	1	2155	251.241	5.53
P22626	Heterogeneous nuclear ribonucleoproteins A2/B1 , GN=HNRNPA2B1	19	123	17	353	37.407	8.95
P34932	Heat shock 70 kDa protein 4 , GN=HSPA4	18	121	17	840	94.271	5.19
B2RXH8	Heterogeneous nuclear ribonucleoprotein C-like 2 , GN=HNRNPCL2	5	153	1	293	32.053	5.44
Q9Y490	Talin-1 , GN=TLN1	33	112	31	2541	269.599	6.07
P60174	Triosephosphate isomerase , GN=TPI1	11	120	11	286	30.772	5.92
P0DP23	Calmodulin-1 , GN=CALM1	8	115	8	149	16.827	4.22
P07195	L-lactate dehydrogenase B chain , GN=LDHB	14	117	13	334	36.615	6.05
Q14697-2	Isoform 2 of Neutral alpha-glucosidase AB , GN=GANAB	21	106	21	966	109.369	6.24
P31942	Heterogeneous nuclear ribonucleoprotein H3 , GN=HNRNPH3	8	112	8	346	36.903	6.87
Q99798	Aconitate hydratase, mitochondrial ,	15	99	15	780	85.372	7.61
P08758	Annexin A5 , GN=ANXA5	12	118	12	320	35.914	5.05
P13667	Protein disulfide-isomerase A4 , GN=PDIA4	27	113	27	645	72.887	5.07
Q08211	ATP-dependent RNA helicase A , GN=DHX9	17	112	17	1270	140.869	6.84
P29401-2	Isoform 2 of Transketolase , GN=TKT	14	99	14	631	68.77	7.52
P78371	T-complex protein 1 subunit beta , GN=CCT2	19	103	19	535	57.452	6.46
Q15233	Non-POU domain-containing octamer-binding protein , GN=NONO	12	114	12	471	54.197	8.95
P42704	Leucine-rich PPR motif-containing protein, mitochondrial , GN=LRPPRC	31	104	31	1394	157.805	6.13
P52209	6-phosphogluconate dehydrogenase, decarboxylating , GN=PGD	13	104	13	483	53.106	7.23
P27797	Calreticulin , GN=CALR	11	99	11	417	48.112	4.44
O43707	Alpha-actinin-4 , GN=ACTN4	25	105	19	911	104.788	5.44

P38646	Stress-70 protein, mitochondrial , GN=HSPA9	17	115	17	679	73.635	6.16
P12956	X-ray repair cross-complementing protein 6 , GN=XRCC6	23	102	23	609	69.799	6.64
P07737	Profilin-1 , GN=PFN1	4	107	4	140	15.045	8.27
P04075-2	Isoform 2 of Fructose-bisphosphate aldolase A , GN=ALDOA	9	97	9	418	45.232	8.25
P61163	Alpha-centractin , GN=ACTR1A	9	98	4	376	42.587	6.64
P26038	Moesin , GN=MSN	17	101	12	577	67.778	6.4
Q14974	Importin subunit beta-1 , GN=KPNB1	16	105	16	876	97.108	4.78
P63010-2	Isoform 2 of AP-2 complex subunit beta , GN=AP2B1	18	92	11	951	105.625	5.34
P40926	Malate dehydrogenase, mitochondrial , GN=MDH2	7	85	7	338	35.481	8.68
P23528	Cofilin-1 , GN=CFL1	10	87	10	166	18.491	8.09
O75369	Filamin-B , GN=FLNB	33	95	31	2602	277.99	5.73
O75643	U5 small nuclear ribonucleoprotein 200 kDa helicase , GN=SNRNP200	31	88	31	2136	244.353	6.06
O43390	Heterogeneous nuclear ribonucleoprotein R , GN=HNRNPR	13	97	12	633	70.899	8.13
P27348	14-3-3 protein theta , GN=YWHAQ	11	90	8	245	27.747	4.78
P45974	Ubiquitin carboxyl-terminal hydrolase 5 , GN=USP5	19	89	19	858	95.725	5.03
P07237	Protein disulfide-isomerase , GN=P4HB	15	90	15	508	57.081	4.87
P12814-3	Isoform 3 of Alpha-actinin-1 , GN=ACTN1	19	93	13	914	105.502	5.41
P22392-2	Isoform 3 of Nucleoside diphosphate kinase B , GN=NME2	7	88	3	267	30.118	8.92
P05455	Lupus La protein , GN=SSB	16	94	16	408	46.808	7.12
P12270	Nucleoprotein TPR , GN=TPR	33	88	33	2363	267.131	5.02
P02766	Transthyretin , GN=TTR	10	75	10	147	15.877	5.76
O43175	D-3-phosphoglycerate dehydrogenase , GN=PHGDH	10	83	10	533	56.614	6.71
P15311	Ezrin , GN=EZR	13	86	9	586	69.37	6.27
P60842	Eukaryotic initiation factor 4A-I , GN=EIF4A1	13	86	10	406	46.125	5.48
P18206	Vinculin , GN=VCL	16	100	16	1134	123.722	5.66
O43347	RNA-binding protein Musashi homolog 1 , GN=MSI1	8	89	8	362	39.099	7.85
P13797	Plastin-3 , GN=PLS3	18	87	18	630	70.766	5.6
P62937	Peptidyl-prolyl cis-trans isomerase A ,	9	91	9	165	18.001	7.81
P40939	Trifunctional enzyme subunit alpha, mitochondrial , GN=HADHA	14	88	14	763	82.947	9.04
P04792	Heat shock protein beta-1 , GN=HSPB1	6	76	6	205	22.768	6.4
Q14194-2	Isoform LCRMP-1 of Dihydropyrimidinase-related protein 1 , GN=CRMP1	14	82	13	686	74.216	6.86
P08865	40S ribosomal protein SA , GN=RPSA	9	83	9	295	32.833	4.87
P09972	Fructose-bisphosphate aldolase C ,	8	78	8	364	39.431	6.87
P14866	Heterogeneous nuclear ribonucleoprotein L , GN=HNRNPL	6	74	6	589	64.092	8.22
Q14240-2	Isoform 2 of Eukaryotic initiation factor 4A-II , GN=EIF4A2	12	81	9	408	46.46	5.48
P48735	Isocitrate dehydrogenase [NADP], mitochondrial , GN=IDH2	9	85	9	452	50.877	8.69
O60506	Heterogeneous nuclear ribonucleoprotein Q , GN=SYNCRIP	16	88	15	623	69.56	8.59
P38606	V-type proton ATPase catalytic subunit A , GN=ATP6V1A	16	77	16	617	68.26	5.52
P17980	26S proteasome regulatory subunit 6A , GN=PSMC3	12	79	12	439	49.172	5.24
P40227	T-complex protein 1 subunit zeta , GN=CCT6A	11	73	11	531	57.988	6.68
Q6P2Q9	Pre-mRNA-processing-splicing factor 8 , GN=PRPF8	26	75	26	2335	273.427	8.84
Q13185	Chromobox protein homolog 3 , GN=CBX3	5	69	4	183	20.798	5.33
P11137	Microtubule-associated protein 2 , GN=MAP2	25	74	25	1827	199.404	4.91
P49321-3	Isoform 3 of Nuclear autoantigenic sperm protein , GN=NASP	10	67	10	790	86.216	4.35
Q99497	Protein/nucleic acid deglycase DJ-1 ,	8	74	8	189	19.878	6.79
Q00839	Heterogeneous nuclear ribonucleoprotein U , GN=HNRNPU SV=6	11	77	11	825	90.528	6

Q14103-2	Isoform 2 of Heterogeneous nuclear ribonucleoprotein D0 , GN=HNRNPD	7	75	1	336	36.249	8.1	
Q14103	Heterogeneous nuclear ribonucleoprotein D0 , GN=HNRNPD	7	75	1	355	38.41	7.81	
Q13263	Transcription intermediary factor 1-beta , GN=TRIM28	13	75	13	835	88.493	5.77	
P83916	Chromobox protein homolog 1 , GN=CBX1	4	66	3	185	21.405	4.93	
Q86VP6	Cullin-associated NEDD8-dissociated protein 1 , GN=CAND1	16	76	15	1230	136.289	5.78	
P48643	T-complex protein 1 subunit epsilon ,	12	67	12	541	59.633	5.66	
P35241-5	Isoform 5 of Radixin , GN=RDX	13	74	8	604	71.005	6.71	
Q96KP4	Cytosolic non-specific dipeptidase ,	14	72	14	475	52.845	5.97	
P50991	T-complex protein 1 subunit delta , GN=CCT4	13	70	13	539	57.888	7.83	
P49588-2	Isoform 2 of Alanine--tRNA ligase, cytoplasmic , GN=AARS	19	72	19	992	109.249	5.57	
Q01105	Protein SET , GN=SET	9	67	6	290	33.469	4.32	
Q12905	Interleukin enhancer-binding factor 2 ,	12	72	12	390	43.035	5.26	
P27816	Microtubule-associated protein 4 , GN=MAP4	24	69	24	1152	120.93	5.43	
Q14257-2	Isoform 2 of Reticulocalbin-2 , GN=RCN2	6	60	6	335	39.115	4.45	
P54819	Adenylate kinase 2, mitochondrial , GN=AK2	8	66	8	239	26.461	7.81	
P11177	Pyruvate dehydrogenase E1 component subunit beta, mitochondrial , GN=PDHB	8	71	8	359	39.208	6.65	
P50990	T-complex protein 1 subunit theta , GN=CCT8	16	78	16	548	59.583	5.6	
P55786	Puromycin-sensitive aminopeptidase , GN=NPEPPS	19	74	19	919	103.211	5.72	
Q14203-4	Isoform 4 of Dynactin subunit 1 , GN=DCTN1	21	72	21	1253	138.664	5.54	
P35221	Catenin alpha-1 , GN=CTNNA1	20	67	18	906	100.009	6.29	
Q9UKA9-2	Isoform 2 of Polypyrimidine tract-binding protein 2 , GN=PTBP2	12	68	1	532	57.611	8.81	
O60664	Perilipin-3 , GN=PLIN3	11	70	11	434	47.046	5.44	
Q92945	Far upstream element-binding protein 2 , GN=KHSRP	11	73	11	711	73.07	7.3	
P51991	Heterogeneous nuclear ribonucleoprotein A3 , GN=HNRNPA3	13	73	1	378	39.571	9.01	
P21399	Cytoplasmic aconitate hydratase , GN=ACO1	13	69	13	889	98.337	6.68	
Q01518	Adenylyl cyclase-associated protein 1 , GN=CAP1	8	67	1	475	51.869	8.06	
Q07866-3	Isoform G of Kinesin light chain 1 , GN=KLC1	16	66	12	564	64.279	6.3	
Q15637-5	Isoform 5 of Splicing factor 1 , GN=SF1	9	64	9	673	71.706	9.58	
P09874	Poly [ADP-ribose] polymerase 1 , GN=PARP1	20	72	20	1014	113.012	8.88	
P62195	26S proteasome regulatory subunit 8 , GN=PSMC5	12	68	12	406	45.597	7.55	
P51991-2	Isoform 2 of Heterogeneous nuclear ribonucleoprotein A3 , GN=HNRNPA3	13	72	1	356	37.006	8.31	
Q92841	Probable ATP-dependent RNA helicase DDX17 , GN=DDX17	13	78	10	729	80.222	8.27	
O43602-2	Isoform 2 of Neuronal migration protein doublecortin , GN=DCX	11	68	9	360	40.019	9.39	
Q15029	116 kDa U5 small nuclear ribonucleoprotein component , GN=EFTUD2	20	72	20	972	109.366		5
Q15417	Calponin-3 , GN=CNN3	8	64	8	329	36.391	6.05	
Q92598	Heat shock protein 105 kDa , GN=HSPH1	13	70	12	858	96.804	5.39	
Q9NQC3	Reticulon-4 , GN=RTN4	12	66	12	1192	129.851	4.5	
Q15149	Plectin , GN=PLEC	38	69	37	4684	531.466	5.96	
P07355-2	Isoform 2 of Annexin A2 , GN=ANXA2	15	66	15	357	40.386	8.37	
Q9NYU2	UDP-glucose:glycoprotein glucosyltransferase 1 , GN=UGGT1	21	63	21	1555	177.078	5.63	
P61204	ADP-ribosylation factor 3 , GN=ARF3	5	63	3	181	20.588	7.43	
Q14315	Filamin-C , GN=FLNC	27	70	24	2725	290.841	5.97	
Q9UHX1	Poly(U)-binding-splicing factor PUF60 , GN=PUF60	9	65	9	559	59.838	5.29	
P19367	Hexokinase-1 , GN=HK1	11	69	9	917	102.42	6.8	
P32119	Peroxiredoxin-2 , GN=PRDX2	3	63	3	198	21.878	5.97	
Q12931	Heat shock protein 75 kDa, mitochondrial , GN=TRAP1	11	63	10	704	80.06	8.21	
Q9UKA9-3	Isoform 3 of Polypyrimidine tract-binding protein 2 , GN=PTBP2	12	59	1	537	58.048	8.81	

P09936	Ubiquitin carboxyl-terminal hydrolase isozyme L1 , GN=UCHL1	8	56	8	223	24.808	5.48
P43686	26S proteasome regulatory subunit 6B , GN=PSMC4	9	60	9	418	47.337	5.21
P23526	Adenosylhomocysteinase , GN=AHCY	10	66	10	432	47.685	6.34
P31939	Bifunctional purine biosynthesis protein PURH , GN=ATIC	14	60	14	592	64.575	6.71
Q10567	AP-1 complex subunit beta-1 , GN=AP1B1	17	62	10	949	104.57	5.06
Q12906-7	Isoform 7 of Interleukin enhancer-binding factor 3 , GN=ILF3	14	59	12	898	95.748	8.81
O00410-3	Isoform 3 of Importin-5 , GN=IPO5	14	66	12	1115	125.464	4.92
P30153	Serine/threonine-protein phosphatase 2A 65 kDa regulatory subunit A alpha isoform , GN=PPP2R1A	16	65	14	589	65.267	5.11
Q03252	Lamin-B2 , GN=LMNB2	20	67	20	620	69.906	5.59
P55795	Heterogeneous nuclear ribonucleoprotein H2 , GN=HNRNPH2	9	57	6	449	49.232	6.3
P20618	Proteasome subunit beta type-1 , GN=PSMB1	7	55	7	241	26.472	8.13
P49368	T-complex protein 1 subunit gamma ,	16	62	16	545	60.495	6.49
P61981	14-3-3 protein gamma , GN=YWHAG	9	64	7	247	28.285	4.89
P02461	Collagen alpha-1(III) chain , GN=COL3A1	18	58	12	1466	138.479	6.61
O00429-6	Isoform 6 of Dynamin-1-like protein ,	13	58	13	749	83.347	7.08
Q9NZI8	Insulin-like growth factor 2 mRNA-binding protein 1 , GN=IGF2BP1	9	63	7	577	63.441	9.2
P17987	T-complex protein 1 subunit alpha , GN=TCP1	13	58	13	556	60.306	6.11
P42025	Beta-centractin , GN=ACTR1B	8	60	3	376	42.267	6.4
P52597	Heterogeneous nuclear ribonucleoprotein F , GN=HNRNPF	7	57	5	415	45.643	5.58
Q52LJ0-2	Isoform 2 of Protein FAM98B , GN=FAM98B	9	47	9	433	45.519	8.69
Q7KZF4	Staphylococcal nuclease domain-containing protein 1 , GN=SND1	20	60	20	910	101.934	7.17
P62316	Small nuclear ribonucleoprotein Sm D2 , GN=SNRPD2	3	47	3	118	13.518	9.91
Q16891	MICOS complex subunit MIC60 , GN=IMMT	15	61	15	758	83.626	6.48
Q92820	Gamma-glutamyl hydrolase , GN=GGH	9	53	9	318	35.941	7.11
P62191	26S proteasome regulatory subunit 4 , GN=PSMC1	9	51	9	440	49.154	6.21
Q99714	3-hydroxyacyl-CoA dehydrogenase type-2 , GN=HSD17B10	9	55	9	261	26.906	7.78
P05388	60S acidic ribosomal protein P0 , GN=RPLP0	10	59	10	317	34.252	5.97
O00425	Insulin-like growth factor 2 mRNA-binding protein 3 , GN=IGF2BP3	11	61	8	579	63.666	8.87
Q01581	Hydroxymethylglutaryl-CoA synthase, cytoplasmic , GN=HMGCS1	10	53	10	520	57.257	5.41
O95757	Heat shock 70 kDa protein 4L , GN=HSPA4L	8	55	6	839	94.453	5.88
O00571	ATP-dependent RNA helicase DDX3X , GN=DDX3X	9	61	8	662	73.198	7.18
P31948	Stress-induced-phosphoprotein 1 , GN=STIP1	15	59	15	543	62.599	6.8
P35222	Catenin beta-1 , GN=CTNNB1	9	56	9	781	85.442	5.86
P62081	40S ribosomal protein S7 , GN=RPS7	6	52	6	194	22.113	10.1
Q15293	Reticulocalbin-1 , GN=RCN1	11	54	11	331	38.866	5
P78347	General transcription factor II-I , GN=GTF2I	17	57	17	998	112.346	6.39
O43399-7	Isoform 7 of Tumor protein D54 ,	6	44	6	229	24.839	6.42
P30041	Peroxiredoxin-6 , GN=PRDX6	9	52	9	224	25.019	6.38
P31946-2	Isoform Short of 14-3-3 protein beta/alpha , GN=YWHAB	10	59	6	244	27.833	4.83
Q16836-2	Isoform 2 of Hydroxyacyl-coenzyme A dehydrogenase, mitochondrial , GN=HADH	7	51	7	390	42.113	9.26
P06753-2	Isoform 2 of Tropomyosin alpha-3 chain , GN=TPM3	13	59	1	248	29.015	4.78
Q8WUM4-2	Isoform 2 of Programmed cell death 6-interacting protein , GN=PDCD6IP	11	50	11	873	96.711	6.52
Q15019-2	Isoform 2 of Septin-2 , GN=SEPT2	10	49	10	396	45.432	6.89
Q01518-2	Isoform 2 of Adenylyl cyclase-associated protein 1 , GN=CAP1	8	55	1	474	51.798	8.06
P11586	C-1-tetrahydrofolate synthase, cytoplasmic , GN=MTHFD1	12	56	12	935	101.495	7.3

Q16181	Septin-7 , GN=SEPT7	11	55	11	437		50.648	8.63
Q99733-2	Isoform 2 of Nucleosome assembly protein 1-like 4 , GN=NAP1L4	9	53	7	386		44.051	4.7
Q99832	T-complex protein 1 subunit eta , GN=CCT7	11	49	11	543		59.329	7.65
Q9Y265	RuvB-like 1 , GN=RUVBL1	11	54	11	456		50.196	6.42
P13804	Electron transfer flavoprotein subunit alpha, mitochondrial , GN=ETFA	8	52	8	333		35.058	8.38
O95865	N(G),N(G)-dimethylarginine dimethylaminohydrolase 2 , GN=DDAH2	7	46	7	285		29.625	6.01
Q96AE4-2	Isoform 2 of Far upstream element-binding protein 1 , GN=FUBP1	13	50	13	653		68.562	7.31
O95336	6-phosphogluconolactonase , GN=PGLS	5	47	5	258	27.53		6.05
Q92769	Histone deacetylase 2 , GN=HDAC2	8	48	5	488		55.329	5.91
P49915	GMP synthase [glutamine-hydrolyzing] , GN=GMPS	11	51	11	693		76.667	6.87
Q9UQ35	Serine/arginine repetitive matrix protein 2 , GN=SRRM2	16	52	16	2752		299.438	12.06
Q9UMX0	Ubiquilin-1 , GN=UBQLN1	8	59	5	589		62.479	5.11
P07954	Fumarate hydratase, mitochondrial , GN=FH	13	45	13	510		54.602	8.76
P33176	Kinesin-1 heavy chain , GN=KIF5B	14	51	9	963		109.617	6.51
P18669	Phosphoglycerate mutase 1 , GN=PGAM1	12	48	12	254		28.786	7.18
Q92499	ATP-dependent RNA helicase DDX1 ,	8	46	8	740	82.38		7.23
P63241-2	Isoform 2 of Eukaryotic translation initiation factor 5A-1 , GN=EIF5A	8	40	8	184		20.157	7.01
P30085	UMP-CMP kinase , GN=CMPK1	9	49	9	196		22.208	5.57
Q9NY33	Dipeptidyl peptidase 3 , GN=DPP3	11	43	11	737		82.538	5.1
Q9Y6M1	Insulin-like growth factor 2 mRNA-binding protein 2 , GN=IGF2BP2	12	54	10	599		66.081	8.46
P55060	Exportin-2 , GN=CSE1L	10	45	10	971		110.346	5.77
P21281	V-type proton ATPase subunit B, brain isoform , GN=ATP6V1B2	11	47	11	511		56.465	5.81
P09960	Leukotriene A-4 hydrolase , GN=LTA4H	14	49	14	611		69.241	6.18
O14531	Dihydropyrimidinase-related protein 4 , GN=DPYSL4	11	47	11	572		61.838	7.09
P53396	ATP-citrate synthase , GN=ACLY	21	54	21	1101		120.762	7.33
P09429	High mobility group protein B1 , GN=HMGB1	9	47	8	215		24.878	5.74
Q8N8S7	Protein enabled homolog , GN=ENAH	9	50	9	591	66.47		6.93
P23396	40S ribosomal protein S3 , GN=RPS3	9	56	9	243		26.671	9.66
Q9C005	Protein dpy-30 homolog , GN=DPY30	4	47	4	99		11.243	4.88
Q7Z6Z7	E3 ubiquitin-protein ligase HUWE1 ,	17	53	17	4374		481.589	5.22
Q13200	26S proteasome non-ATPase regulatory subunit 2 , GN=PSMD2	13	51	13	908		100.136	5.2
Q16658	Fascin , GN=FSCN1	9	43	9	493		54.496	7.24
P29762	Cellular retinoic acid-binding protein 1 , GN=CRABP1	6	52	6	137		15.556	5.38
P00338-3	Isoform 3 of L-lactate dehydrogenase A chain , GN=LDHA	12	47	11	361		39.812	8.43
O43852-3	Isoform 3 of Calumenin , GN=CALU	10	42	2	323		38.027	4.63
Q6FI13	Histone H2A type 2-A , GN=HIST2H2AA3	4	48	3	130		14.087	10.9
O75506	Heat shock factor-binding protein 1 ,	5	40	5	76		8.538	4.36
O75533	Splicing factor 3B subunit 1 , GN=SF3B1	18	48	18	1304		145.738	7.09
P06753-6	Isoform 6 of Tropomyosin alpha-3 chain , GN=TPM3	14	51	1	248		28.905	4.75
P07339	Cathepsin D , GN=CTSD	5	49	5	412		44.524	6.54
P05091	Aldehyde dehydrogenase, mitochondrial , GN=ALDH2	10	48	10	517		56.346	7.05
P49411	Elongation factor Tu, mitochondrial ,	9	48	9	452	49.51		7.61
P15531-2	Isoform 2 of Nucleoside diphosphate kinase A , GN=NME1	5	46	1	177		19.641	5.58
Q15393	Splicing factor 3B subunit 3 , GN=SF3B3	13	46	13	1217		135.492	5.26
Q16881	Thioredoxin reductase 1, cytoplasmic , GN=TXNRD1	10	44	10	649		70.862	7.39
Q9UPN3-2	Isoform 2 of Microtubule-actin cross-linking factor 1, isoforms 1/2/3/5 , GN=MACF1	24	46	23	5430		620.034	5.38
Q04837	Single-stranded DNA-binding protein, mitochondrial , GN=SSBP1	3	41	3	148		17.249	9.6
Q9Y230	RuvB-like 2 , GN=RUVBL2	10	47	10	463		51.125	5.64

Q02790	Peptidyl-prolyl cis-trans isomerase FKBP4 , GN=FKBP4	10	42	10	459		51.772	5.43	
P51659	Peroxisomal multifunctional enzyme type 2 , GN=HSD17B4	15	47	15	736		79.636	8.84	
O75947	ATP synthase subunit d, mitochondrial , GN=ATP5H	7	48	7	161		18.479	5.3	
Q14839-2	Isoform 2 of Chromodomain-helicase-DNA-binding protein 4 , GN=CHD4	18	44	18	1940		220.709	5.97	
Q9BWD1	Acetyl-CoA acetyltransferase, cytosolic , GN=ACAT2	6	39	6	397		41.324	6.92	
P30044	Peroxiredoxin-5, mitochondrial , GN=PRDX5	7	45	7	214		22.073	8.7	
P06744	Glucose-6-phosphate isomerase , GN=GPI	9	44	9	558		63.107	8.32	
Q13011	Delta(3,5)-Delta(2,4)-dienoyl-CoA isomerase, mitochondrial , GN=ECH1	5	45	5	328		35.793		8
P49748-3	Isoform 3 of Very long-chain specific acyl-CoA dehydrogenase, mitochondrial , GN=ACADVL	15	48	15	678		72.881	8.75	
Q00341	Vigilin , GN=HDLBP	16	40	16	1268		141.368	6.87	
Q15717-2	Isoform 2 of ELAV-like protein 1 , GN=ELAVL1	9	43	9	353		38.972	9.45	
Q07666	KH domain-containing, RNA-binding, signal transduction-associated protein 1 , GN=KHDRBS1	5	37	5	443		48.197	8.66	
O43852-4	Isoform 4 of Calumenin , GN=CALU	9	38	1	323		38.055	4.58	
P27824-2	Isoform 2 of Calnexin , GN=CANX	7	45	7	627		71.458	4.7	
Q9P2J5	Leucine--tRNA ligase, cytoplasmic , GN=LARS	13	42	13	1176		134.379	7.3	
P26368	Splicing factor U2AF 65 kDa subunit ,	8	40	8	475		53.467	9.09	
P10253	Lysosomal alpha-glucosidase , GN=GAA	9	44	9	952		105.257		6
P13674	Prolyl 4-hydroxylase subunit alpha-1 , GN=P4HA1	10	40	10	534		61.011	6.01	
P67936	Tropomyosin alpha-4 chain , GN=TPM4	12	45	6	248		28.504	4.69	
Q13148	TAR DNA-binding protein 43 , GN=TARDBP	4	42	4	414		44.711	6.19	
Q15365	Poly(rC)-binding protein 1 , GN=PCBP1	5	41	3	356		37.474	7.09	
O95433	Activator of 90 kDa heat shock protein ATPase homolog 1 , GN=AHSA1	6	38	6	338	38.25		5.53	
P17174	Aspartate aminotransferase, cytoplasmic , GN=GOT1	7	40	7	413		46.219	7.01	
Q99460	26S proteasome non-ATPase regulatory subunit 1 , GN=PSMD1	13	44	13	953		105.769	5.39	
P51610	Host cell factor 1 , GN=HCFC1	14	44	14	2035		208.602	7.46	
P62140	Serine/threonine-protein phosphatase PP1-beta catalytic subunit , GN=PPP1CB	3	36	2	327		37.163	6.19	
Q9Y3I0	tRNA-splicing ligase RtcB homolog , GN=RTCB	14	43	14	505		55.175	7.23	
Q8WXF1	Paraspeckle component 1 , GN=PSPC1	10	42	10	523		58.706	6.67	
P14314	Glucosidase 2 subunit beta , GN=PRKCSH	11	48	11	528		59.388	4.41	
P05387	60S acidic ribosomal protein P2 , GN=RPLP2	5	39	5	115		11.658	4.54	
P11940	Polyadenylate-binding protein 1 ,	14	43	8	636		70.626	9.5	
Q04917	14-3-3 protein eta , GN=YWHAH	8	48	7	246		28.201	4.84	
P61758	Prefoldin subunit 3 , GN=VBP1	4	36	4	197		22.643	7.11	
Q9HB71	Calcyclin-binding protein , GN=CACYBP	9	38	9	228		26.194	8.25	
P51665	26S proteasome non-ATPase regulatory subunit 7 , GN=PSMD7	9	38	9	324		37.002	6.77	
Q16643-3	Isoform 3 of Drebrin , GN=DBN1	9	38	9	695		76.253	4.51	
P50570-4	Isoform 4 of Dynamin-2 , GN=DNM2	12	42	11	870		97.905	7.46	
Q92616	eIF-2-alpha kinase activator GCN1 , GN=GCN1 SV=6	17	38	17	2671		292.572	7.47	
Q02952	A-kinase anchor protein 12 , GN=AKAP12	12	39	12	1782		191.367	4.41	
P30040	Endoplasmic reticulum resident protein 29 , GN=ERP29	9	43	9	261		28.975	7.31	
Q9P2K5	Myelin expression factor 2 , GN=MYEF2	11	43	2	600		64.081	8.75	
P17844	Probable ATP-dependent RNA helicase DDX5 , GN=DDX5	10	47	7	614		69.105	8.92	
Q9UBB4	Ataxin-10 , GN=ATXN10	10	45	10	475		53.455	5.25	
Q9H9B4	Sideroflexin-1 , GN=SFXN1	4	45	4	322		35.596	9.07	
P62805	Histone H4 , GN=HIST1H4A	8	49	8	103	11.36		11.36	
P60660	Myosin light polypeptide 6 , GN=MYL6	6	40	4	151		16.919	4.65	
Q9UJU6-3	Isoform 3 of Drebrin-like protein , GN=DBNL	7	40	7	439		49.012	5.05	
P61158	Actin-related protein 3 , GN=ACTR3	8	41	8	418		47.341	5.88	
Q9H0B6	Kinesin light chain 2 , GN=KLC2	9	36	4	622		68.892	7.15	

P16104	Histone H2AX , GN=H2AFX	3	34	2	143		15.135	10.74	
Q9NRR5	Ubiquilin-4 , GN=UBQLN4	8	48	6	601		63.812	5.22	
P55209	Nucleosome assembly protein 1-like 1 , GN=NAP1L1	8	41	6	391		45.346	4.46	
Q9UQE7	Structural maintenance of chromosomes protein 3 , GN=SMC3	15	41	15	1217		141.454	7.18	
Q13435	Splicing factor 3B subunit 2 , GN=SF3B2	10	36	10	895		100.165	5.67	
P00918	Carbonic anhydrase 2 , GN=CA2	8	38	8	260		29.228	7.4	
P25787	Proteasome subunit alpha type-2 ,	6	34	6	234		25.882	7.43	
P31323	cAMP-dependent protein kinase type II-beta regulatory subunit , GN=PRKAR2B	4	38	3	418		46.273	4.92	
P62829	60S ribosomal protein L23 , GN=RPL23	2	39	2	140		14.856	10.51	
P18583-5	Isoform D of Protein SON , GN=SON	13	41	13	2459		266.866	5.73	
P16949	Stathmin , GN=STMN1	7	42	7	149		17.292	5.97	
P38159	RNA-binding motif protein, X chromosome , GN=RBMX	8	40	8	391		42.306	10.05	
Q14152	Eukaryotic translation initiation factor 3 subunit A , GN=EIF3A	15	39	15	1382		166.468	6.79	
E9PAV3	subunit alpha, muscle-specific form , GN=NACA	6	39	5	2078		205.295	9.58	
P43246	DNA mismatch repair protein Msh2 ,	6	34	6	934		104.677	5.77	
P47756-2	Isoform 2 of F-actin-capping protein subunit beta , GN=CAPZB	10	41	10	272		30.609		6
P26232-5	Isoform 5 of Catenin alpha-2 , GN=CTNNA2	13	36	11	939		104.208	6.27	
Q86UP2	Kinectin , GN=KTN1	13	34	13	1357		156.179	5.64	
P13861	cAMP-dependent protein kinase type II-alpha regulatory subunit , GN=PRKAR2A	6	35	5	404	45.49		5.07	
Q06323	Proteasome activator complex subunit 1 , GN=PSME1	7	33	7	249		28.705	6.02	
Q9BPU6	Dihydropyrimidinase-related protein 5 , GN=DPYSL5	10	37	10	564		61.382	7.2	
O60701	UDP-glucose 6-dehydrogenase , GN=UGDH	11	32	11	494		54.989	7.12	
P04632	Calpain small subunit 1 , GN=CAPNS1	5	34	5	268		28.298	5.2	
P09493-4	Isoform 4 of Tropomyosin alpha-1 chain , GN=TPM1	14	40	6	284		32.828	4.77	
Q9NPH2	Inositol-3-phosphate synthase 1 , GN=ISYNA1	5	36	5	558		61.029	5.76	
Q12888-2	Isoform 2 of TP53-binding protein 1 , GN=TP53BP1	16	37	16	1977		213.985	4.7	
Q15459	Splicing factor 3A subunit 1 , GN=SF3A1	11	33	11	793		88.831	5.22	
Q16352	Alpha-internexin , GN=INA	10	36	9	499		55.357	5.4	
P40925-3	Isoform 3 of Malate dehydrogenase, cytoplasmic , GN=MDH1	9	40	9	352		38.603	7.71	
P61088	Ubiquitin-conjugating enzyme E2 N ,	3	40	3	152		17.127	6.57	
P62136-2	Isoform 2 of Serine/threonine-protein phosphatase PP1-alpha catalytic subunit , GN=PPP1CA	3	30	2	341		38.606	6.62	
P23588	Eukaryotic translation initiation factor 4B , GN=EIF4B	7	33	7	611	69.11		5.73	
O14818	Proteasome subunit alpha type-7 ,	6	33	6	248	27.87		8.46	
P35232	Prohibitin , GN=PHB	7	35	7	272		29.786	5.76	
P02787	Serotransferrin , GN=TF	13	38	13	698		77.014	7.12	
Q9P2K5-2	Isoform 2 of Myelin expression factor 2 , GN=MYEF2	10	36	1	576		61.889	8.62	
Q99623	Prohibitin-2 , GN=PHB2	7	37	7	299		33.276	9.83	
Q02252	dehydrogenase [acylating], mitochondrial , GN=ALDH6A1	11	33	11	535		57.803	8.5	
O60282	Kinesin heavy chain isoform 5C , GN=KIF5C	16	39	10	957		109.427	6.19	
Q9UNZ2-5	Isoform 3 of NSFL1 cofactor p47 , GN=NSFL1C	9	34	9	372		40.791	5.15	
O75531	Barrier-to-autointegration factor , GN=BANF1	3	37	3	89		10.052	6.09	
P80404	4-aminobutyrate aminotransferase, mitochondrial , GN=ABAT	10	36	10	500		56.403	7.96	
P00491	Purine nucleoside phosphorylase , GN=PNP	8	33	8	289		32.097	6.95	
Q99729-2	Isoform 2 of Heterogeneous nuclear ribonucleoprotein A/B , GN=HNRNPAB	5	36	5	332		35.945	6.95	
Q9UHD8	Septin-9 , GN=SEPT9	7	33	7	586		65.361	8.97	
Q00688	Peptidyl-prolyl cis-trans isomerase FKBP3 , GN=FKBP3	7	36	7	224		25.161	9.28	

O14773	Tripeptidyl-peptidase 1 , GN=TPP1	3	29	3	563	61.21		6.48
P41250	Glycine--tRNA ligase , GN=GARS	11	30	11	739		83.113	7.03
Q14914	Prostaglandin reductase 1 , GN=PTGR1	7	35	7	329		35.847	8.29
P54136	Arginine--tRNA ligase, cytoplasmic , GN=RARS	9	38	9	660		75.331	6.68
P22061-2	Isoform 2 of Protein-L-isoaspartate(D-aspartate) O-methyltransferase , GN=PCMT1	7	32	7	228		24.664	6.52
P50502	Hsc70-interacting protein , GN=ST13	4	34	4	369		41.305	5.27
P36776	Lon protease homolog, mitochondrial , GN=LONP1	10	34	10	959		106.422	6.39
P62333	26S proteasome regulatory subunit 10B , GN=PSMC6	9	36	9	389		44.145	7.49
P54886	Delta-1-pyrroline-5-carboxylate synthase , GN=ALDH18A1	12	33	12	795		87.248	7.12
Q9UHD9	Ubiquilin-2 , GN=UBQLN2	6	41	4	624		65.655	5.22
O60763-2	Isoform 2 of General vesicular transport factor p115 , GN=USO1	12	36	12	973		109.127	4.89
Q13310-3	Isoform 3 of Polyadenylate-binding protein 4 , GN=PABPC4	8	33	2	660		72.345	9.35
Q13409-2	Isoform 2B of Cytoplasmic dynein 1 intermediate chain 2 , GN=DYNC1I2	6	33	6	632		70.601	5.24
Q9P2E9	Ribosome-binding protein 1 , GN=RRBP1	13	33	13	1410		152.381	8.6
Q9Y696	Chloride intracellular channel protein 4 , GN=CLIC4	6	30	6	253		28.754	5.59
O94905	Erlin-2 , GN=ERLIN2	9	32	7	339		37.815	5.62
P30086	Phosphatidylethanolamine-binding protein 1 , GN=PEBP1	6	32	6	187		21.044	7.53
Q9HC38	Glyoxalase domain-containing protein 4 , GN=GLOD4	5	30	5	313		34.771	5.6
P17812	CTP synthase 1 , GN=CTPS1	7	33	5	591		66.648	6.46
P51858	Hepatoma-derived growth factor , GN=HDGF	7	29	7	240		26.772	4.73
P26640	Valine--tRNA ligase , GN=VARS	14	33	14	1264		140.387	7.59
A6NHL2	Tubulin alpha chain-like 3 , GN=TUBAL3	3	34	1	446		49.877	6.05
P37802-2	Isoform 2 of Transgelin-2 , GN=TAGLN2	6	37	5	220		24.438	8.25
Q9UJZ1	Stomatin-like protein 2, mitochondrial , GN=STOML2	10	34	10	356	38.51		7.39
P07814	Bifunctional glutamate/proline--tRNA ligase , GN=EPRS	13	33	13	1512		170.483	7.33
P28482	Mitogen-activated protein kinase 1 ,	7	27	6	360		41.363	6.98
P51149	Ras-related protein Rab-7a , GN=RAB7A	8	32	8	207		23.475	6.7
P78559-2	Isoform 2 of Microtubule-associated protein 1A , GN=MAP1A	13	35	12	2805		305.484	4.92
P33993	DNA replication licensing factor MCM7 , GN=MCM7	4	30	4	719		81.257	6.46
O60888-2	Isoform A of Protein CutA , GN=CUTA	3	29	3	198		20.912	5.48
P0DME0	Protein SETSIP , GN=SETSIP	4	33	1	302		34.861	4.31
P35606	Coatomer subunit beta' , GN=COPB2	11	34	11	906		102.422	5.27
P15586	N-acetylglucosamine-6-sulfatase , GN=GNS	5	32	5	552		62.042	8.31
P55010	Eukaryotic translation initiation factor 5 , GN=EIF5	1	31	1	431		49.192	5.58
P46109	Crk-like protein , GN=CRKL	6	30	6	303		33.756	6.74
O75390	Citrate synthase, mitochondrial , GN=CS	5	28	5	466	51.68		8.32
Q9UJS0-2	Isoform 2 of Calcium-binding mitochondrial carrier protein Aralar2 , GN=SLC25A13	9	31	9	676		74.257	8.62
Q9NUQ9	Protein FAM49B , GN=FAM49B	6	33	5	324		36.725	6.06
P62826	GTP-binding nuclear protein Ran , GN=RAN	6	29	6	216		24.408	7.49
P49756	RNA-binding protein 25 , GN=RBM25	5	30	5	843		100.124	6.32
P23368	NAD-dependent malic enzyme, mitochondrial , GN=ME2	6	29	6	584		65.402	7.61
P02452	Collagen alpha-1(I) chain , GN=COL1A1	12	31	12	1464		138.857	5.8
Q9GZS3	WD repeat-containing protein 61 ,	5	27	5	305	33.56		5.47
P29692-2	Isoform 2 of Elongation factor 1-delta , GN=EEF1D	8	33	8	647		71.364	6.42
P30084	Enoyl-CoA hydratase, mitochondrial ,	4	34	4	290		31.367	8.07
P28074	Proteasome subunit beta type-5 , GN=PSMB5	9	34	9	263		28.462	6.92
P42166	Lamina-associated polypeptide 2, isoform alpha , GN=TMPO	10	29	4	694		75.446	7.66

O75368	SH3 domain-binding glutamic acid-rich-like protein , GN=SH3BGRL	7	31	7	114		12.766	5.25	
Q9Y6G9	Cytoplasmic dynein 1 light intermediate chain 1 , GN=DYNC1LI1	7	27	7	523		56.544	6.42	
P49792	E3 SUMO-protein ligase RanBP2 ,	14	30	14	3224		357.974	6.2	
O43488	Aflatoxin B1 aldehyde reductase member 2 , GN=AKR7A2	6	35	6	359		39.564	7.17	
P05937	Calbindin , GN=CALB1	9	29	9	261		30.006	4.83	
O14950	Myosin regulatory light chain 12B ,	9	32	9	172		19.767	4.84	
Q13838-2	Isoform 2 of Spliceosome RNA helicase DDX39B , GN=DDX39B	8	30	3	443		50.647	5.88	
P51784	Ubiquitin carboxyl-terminal hydrolase 11 , GN=USP11	11	30	11	963		109.747	5.45	
Q9Y3F4-2	Isoform 2 of Serine-threonine kinase receptor-associated protein , GN=STRAP	5	28	5	363		39.753	5.31	
Q15436	Protein transport protein Sec23A ,	8	30	7	765		86.105	7.08	
P24752	Acetyl-CoA acetyltransferase, mitochondrial , GN=ACAT1	5	30	5	427		45.171	8.85	
O14980	Exportin-1 , GN=XPO1	12	31	12	1071		123.306	6.06	
Q96FW1	Ubiquitin thioesterase OTUB1 , GN=OTUB1	7	28	7	271		31.264	4.94	
P08708	40S ribosomal protein S17 , GN=RPS17	7	28	7	135	15.54		9.85	
Q13151	Heterogeneous nuclear ribonucleoprotein A0 , GN=HNRNPA0	5	28	5	305		30.822	9.29	
P49903	Selenide, water dikinase 1 , GN=SEPHS1	8	28	8	392		42.883	5.97	
Q9NTK5	Obg-like ATPase 1 , GN=OLA1	8	30	8	396		44.715	7.81	
O60784-2	Isoform 2 of Target of Myb protein 1 ,	11	30	11	493		53.841	4.74	
Q08209	Serine/threonine-protein phosphatase 2B catalytic subunit alpha isoform , GN=PPP3CA	4	28	1	521	58.65		5.86	
Q02880	DNA topoisomerase 2-beta , GN=TOP2B	16	29	13	1626		183.152		8
P52907	F-actin-capping protein subunit alpha-1 , GN=CAPZA1	6	31	6	286		32.902	5.69	
P49419-2	Isoform 2 of Alpha-aminoadipic semialdehyde dehydrogenase , GN=ALDH7A1	9	30	9	511		55.331	6.86	
P25325-2	Isoform 2 of 3-mercaptopyruvate sulfurtransferase , GN=MPST	4	24	4	317		35.228	6.61	
P21796	Voltage-dependent anion-selective channel protein 1 , GN=VDAC1	7	30	7	283		30.754	8.54	
P52565	Rho GDP-dissociation inhibitor 1 ,	3	28	3	204		23.193	5.11	
Q99536	Synaptic vesicle membrane protein VAT-1 homolog , GN=VAT1	7	31	7	393		41.893	6.29	
O95373	Importin-7 , GN=IPO7	10	30	10	1038	119.44		4.82	
Q8N1F7	Nuclear pore complex protein Nup93 , GN=NUP93	9	31	9	819	93.43		5.72	
P28070	Proteasome subunit beta type-4 , GN=PSMB4	4	26	4	264		29.185	5.97	
O00487	26S proteasome non-ATPase regulatory subunit 14 , GN=PSMD14	3	25	3	310		34.555	6.52	
Q9H0U4	Ras-related protein Rab-1B , GN=RAB1B	6	26	4	201		22.157	5.73	
P56537	Eukaryotic translation initiation factor 6 , GN=EIF6	4	27	4	245		26.582	4.68	
P53618	Coatomer subunit beta , GN=COPB1	7	25	7	953		107.074	6.05	
Q8N568-3	Isoform 3 of Serine/threonine-protein kinase DCLK2 , GN=DCLK2	4	26	2	783	85.33		8.53	
P49591	Serine--tRNA ligase, cytoplasmic , GN=SARS	6	23	6	514	58.74		6.43	
P62979	Ubiquitin-40S ribosomal protein S27a , GN=RPS27A	2	29	2	156		17.953	9.64	
P28072	Proteasome subunit beta type-6 , GN=PSMB6	4	22	4	239		25.341	4.92	
P14550	Alcohol dehydrogenase [NADP(+)] ,	6	29	6	325	36.55		6.79	
P33991	DNA replication licensing factor MCM4 , GN=MCM4	13	26	13	863		96.498	6.74	
P16298-4	Isoform 4 of Serine/threonine-protein phosphatase 2B catalytic subunit beta isoform , GN=PPP3CB	4	26	1	525		59.086	5.91	
P22695	Cytochrome b-c1 complex subunit 2, mitochondrial , GN=UQCRC2	7	27	7	453		48.413	8.63	
Q13153	Serine/threonine-protein kinase PAK 1 , GN=PAK1	8	27	6	545		60.609	5.76	
Q6DKJ4	Nucleoredoxin , GN=NXN	5	27	5	435		48.362	4.97	

P10909-2	Isoform 2 of Clusterin , GN=CLU	6	28	6	501	57.796	6.68
P42167	Lamina-associated polypeptide 2, isoforms beta/gamma , GN=TMPO	7	25	1	454	50.639	9.38
Q09028	Histone-binding protein RBBP4 , GN=RBBP4	5	25	4	425	47.626	4.89
O14497	AT-rich interactive domain-containing protein 1A , GN=ARID1A	9	27	9	2285	241.892	6.7
Q92734	Protein TFG , GN=TFG	5	24	5	400	43.421	5.1
P12111	Collagen alpha-3(VI) chain , GN=COL6A3	11	27	11	3177	343.457	6.68
P07858	Cathepsin B , GN=CTSB	4	24	4	339	37.797	6.3
P08123	Collagen alpha-2(I) chain , GN=COL1A2 SV=7	11	28	11	1366	129.235	8.95
O43809	Cleavage and polyadenylation specificity factor subunit 5 , GN=NUDT21	5	28	5	227	26.211	8.82
P50453	Serpin B9 , GN=SERPINB9	7	31	7	376	42.376	5.86
P98175	RNA-binding protein 10 , GN=RBM10	7	25	7	930	103.469	5.97
Q15366-2	Isoform 2 of Poly(rC)-binding protein 2 , GN=PCBP2	4	25	2	366	38.627	6.79
Q12874	Splicing factor 3A subunit 3 , GN=SF3A3	8	26	8	501	58.812	5.38
P48147	Prolyl endopeptidase , GN=PREP	11	28	11	710	80.648	5.86
O60716-3	Isoform 1AC of Catenin delta-1 , GN=CTNND1	13	27	13	939	104.912	6.87
P52701	DNA mismatch repair protein Msh6 ,	9	27	9	1360	152.689	6.9
P35611-3	Isoform 3 of Alpha-adducin , GN=ADD1	10	26	10	768	84.25	5.96
Q14498	RNA-binding protein 39 , GN=RBM39	8	26	8	530	59.343	10.1
Q8IY67-2	Isoform 2 of Ribonucleoprotein PTB-binding 1 , GN=RAVER1	9	25	9	739	77.811	8.84
Q96JE9	Microtubule-associated protein 6 , GN=MAP6	8	26	8	813	86.452	9.16
P46926	Glucosamine-6-phosphate isomerase 1 , GN=GNPDA1	5	29	5	289	32.648	6.92
Q16576-2	Isoform 2 of Histone-binding protein RBBP7 , GN=RBBP7	4	24	3	469	52.282	5.27
Q9UBE0	SUMO-activating enzyme subunit 1 ,	8	26	8	346	38.426	5.3
Q14247	Src substrate cortactin , GN=CTTN	7	27	7	550	61.549	5.4
Q9UMS4	Pre-mRNA-processing factor 19 , GN=PRPF19	5	22	5	504	55.146	6.61
Q99436	Proteasome subunit beta type-7 , GN=PSMB7	3	28	3	277	29.946	7.68
P14678-3	Isoform SM-B1 of Small nuclear ribonucleoprotein-associated proteins B and B' , GN=SNRPB	2	26	2	289	30.012	10.18
Q7L014	Probable ATP-dependent RNA helicase DDX46 , GN=DDX46	6	25	6	1031	117.29	9.29
O75955	Flotillin-1 , GN=FLOT1	8	23	8	427	47.326	7.49
P28161	Glutathione S-transferase Mu 2 , GN=GSTM2	5	26	5	218	25.728	6.37
Q9UK76-2	Isoform 2 of Jupiter microtubule associated homolog 1 , GN=JPT1	4	21	4	181	19.904	8.32
P25205-2	Isoform 2 of DNA replication licensing factor MCM3 , GN=MCM3	9	25	9	853	95.848	5.77
P22234	Multifunctional protein ADE2 , GN=PAICS	8	25	8	425	47.049	7.23
P41219-2	Isoform 2 of Peripherin , GN=PRPH	7	30	3	471	53.746	5.47
P23381	Tryptophan--tRNA ligase, cytoplasmic , GN=WARS	8	25	8	471	53.132	6.23
O14974	Protein phosphatase 1 regulatory subunit 12A , GN=PPP1R12A	10	24	10	1030	115.211	5.4
P26641-2	Isoform 2 of Elongation factor 1-gamma , GN=EEF1G	7	26	7	487	56.114	7.72
O75663	TIP41-like protein , GN=TIPRL	5	24	5	272	31.424	5.91
Q07065	Cytoskeleton-associated protein 4 ,	9	24	9	602	65.983	5.92
P67809	Nuclease-sensitive element-binding protein 1 , GN=YBX1	4	24	1	324	35.903	9.88
Q8N163	Cell cycle and apoptosis regulator protein 2 , GN=CCAR2	7	27	7	923	102.838	5.22
P06865	Beta-hexosaminidase subunit alpha ,	4	20	4	529	60.664	5.16
Q1KMD3	Heterogeneous nuclear ribonucleoprotein U-like protein 2 , GN=HNRNPUL2	9	24	9	747	85.052	4.91
O75347	Tubulin-specific chaperone A , GN=TBCA	7	22	7	108	12.847	5.29
Q9ULV4-3	Isoform 3 of Coronin-1C , GN=CORO1C	4	20	4	527	58.91	7.75
P80303	Nucleobindin-2 , GN=NUCB2	8	24	8	420	50.164	5.12
P06396	Gelsolin , GN=GSN	8	22	8	782	85.644	6.28
P49721	Proteasome subunit beta type-2 , GN=PSMB2	5	22	5	201	22.822	7.02

Q13283	Ras GTPase-activating protein-binding protein 1 , GN=G3BP1	7	22	6	466	52.132	5.52
P30048	Thioredoxin-dependent peroxide reductase, mitochondrial , GN=PRDX3	3	23	3	256	27.675	7.78
P09417	Dihydropteridine reductase , GN=QDPR	4	22	4	244	25.773	7.37
Q9BXW7	Haloacid dehalogenase-like hydrolase domain-containing 5 , GN=HDHD5	6	23	6	423	46.292	8.13
P09493-2	Isoform 2 of Tropomyosin alpha-1 chain , GN=TPM1	10	28	2	228	26.664	4.82
P54920	Alpha-soluble NSF attachment protein , GN=NAPA	7	24	7	295	33.211	5.36
O43396	Thioredoxin-like protein 1 , GN=TXNL1	4	27	4	289	32.231	4.96
Q9BZK3	Putative nascent polypeptide-associated complex subunit alpha-like protein , GN=NACAP1 PE=5	2	24	1	213	23.292	4.59
P35998	26S proteasome regulatory subunit 7 , GN=PSMC2	10	26	10	433	48.603	5.95
P54578	Ubiquitin carboxyl-terminal hydrolase 14 , GN=USP14	8	25	8	494	56.033	5.3
Q99426	Tubulin-folding cofactor B , GN=TBCB	6	26	6	244	27.308	5.15
P08237-3	phosphofructokinase, muscle type , GN=PFKM	10	25	10	851	93.194	7.97
P46060	Ran GTPase-activating protein 1 ,	10	24	10	587	63.502	4.68
Q9UI15	Transgelin-3 , GN=TAGLN3	3	27	2	199	22.458	7.33
Q07955-2	Isoform ASF-2 of Serine/arginine-rich splicing factor 1 , GN=SRSF1	7	30	7	292	31.979	5.91
O15027	Protein transport protein Sec16A ,	10	24	10	2179	233.373	5.63
P31689	DnaJ homolog subfamily A member 1 , GN=DNAJA1	5	22	5	397	44.839	7.08
Q01130	Serine/arginine-rich splicing factor 2 ,	1	21	1	221	25.461	11.85
O15020	Spectrin beta chain, non-erythrocytic 2 , GN=SPTBN2	9	26	8	2390	271.157	6.11
Q16851	UTP--glucose-1-phosphate uridylyltransferase , GN=UGP2	8	23	8	508	56.905	8.15
P31937	3-hydroxyisobutyrate dehydrogenase, mitochondrial , GN=HIBADH	7	25	7	336	35.306	8.13
Q7L099-2	Isoform 2 of Protein RUFY3 , GN=RUFY3	7	24	7	506	55.839	5.57
O43143	Pre-mRNA-splicing factor ATP-dependent RNA helicase DHX15 , GN=DHX15	11	27	11	795	90.875	7.46
Q9UNM6-2	Isoform 2 of 26S proteasome non-ATPase regulatory subunit 13 , GN=PSMD13	4	23	4	378	42.915	6.54
Q9NRX4	14 kDa phosphohistidine phosphatase , GN=PHPT1	4	24	4	125	13.824	6.07
Q9Y224	UPF0568 protein C14orf166 , GN=C14orf166	7	25	7	244	28.051	6.65
P00367	Glutamate dehydrogenase 1, mitochondrial , GN=GLUD1	12	25	12	558	61.359	7.8
Q9UBF2	Coatomer subunit gamma-2 , GN=COPG2	6	23	5	871	97.56	5.81
P09455-3	Isoform 3 of Retinol-binding protein 1 ,	5	25	5	157	17.512	6.19
P46379-3	Isoform 3 of Large proline-rich protein BAG6 , GN=BAG6	11	26	11	1162	122.265	5.69
Q07021	Complement component 1 Q subcomponent-binding protein, mitochondrial , GN=C1QBP	5	22	5	282	31.343	4.84
P61011	Signal recognition particle 54 kDa protein , GN=SRP54	10	21	10	504	55.668	8.75
O00154	Cytosolic acyl coenzyme A thioester hydrolase , GN=ACOT7	5	26	5	380	41.769	8.54
Q9HCJ6	Synaptic vesicle membrane protein VAT-1 homolog-like , GN=VAT1L	8	23	8	419	45.87	5.06
Q9NYL9	Tropomodulin-3 , GN=TMOD3	7	24	7	352	39.57	5.19
P60891	Ribose-phosphate pyrophosphokinase 1 , GN=PRPS1	5	24	5	318	34.812	6.98
P09493-5	Isoform 5 of Tropomyosin alpha-1 chain , GN=TPM1	9	27	1	245	28.368	4.77
P10768	S-formylglutathione hydrolase , GN=ESD	4	22	4	282	31.442	7.02
P61923	Coatomer subunit zeta-1 , GN=COPZ1	4	23	4	177	20.185	4.81
Q9UQ80	Proliferation-associated protein 2G4 , GN=PA2G4	8	27	8	394	43.759	6.55

Q04760	Lactoylglutathione lyase , GN=GLO1	3	22	3	184	20.764	5.31
P28331-2	oxidoreductase 75 kDa subunit, mitochondrial , GN=NDUFS1	10	25	10	741	80.945	6.64
Q8N1G4	Leucine-rich repeat-containing protein 47 , GN=LRRC47	7	25	7	583	63.434	8.28
O75915	PRA1 family protein 3 , GN=ARL6IP5	2	18	2	188	21.6	9.77
Q9C0C2	182 kDa tankyrase-1-binding protein , GN=TNKS1BP1	10	22	10	1729	181.685	4.86
P46940	Ras GTPase-activating-like protein IQGAP1 , GN=IQGAP1	16	23	16	1657	189.134	6.48
Q15056	Eukaryotic translation initiation factor 4H , GN=EIF4H	4	22	4	248	27.368	7.23
Q96P70	Importin-9 , GN=IPO9	9	23	9	1041	115.889	4.81
Q96PU8	Protein quaking , GN=QKI	6	25	6	341	37.647	8.56
P02545	Prelamin-A/C , GN=LMNA	10	25	10	664	74.095	7.02
Q9UBT2	SUMO-activating enzyme subunit 2 ,	8	19	8	640	71.179	5.29
Q13561-2	Isoform 2 of Dynactin subunit 2 , GN=DCTN2	6	20	6	406	44.792	5.17
P30419	Glycylpeptide N-tetradecanoyltransferase 1 , GN=NMT1	6	19	6	496	56.77	7.8
Q9UNW9	RNA-binding protein Nova-2 , GN=NOVA2	6	22	5	492	48.979	8.16
O14770	Homeobox protein Meis2 , GN=MEIS2	3	19	2	477	51.757	6.4
Q5T4S7-2	Isoform 2 of E3 ubiquitin-protein ligase UBR4 , GN=UBR4	9	24	9	5204	575.592	6.01
P05023	Sodium/potassium-transporting ATPase subunit alpha-1 , GN=ATP1A1	7	24	5	1023	112.824	5.49
O00231-2	Isoform 2 of 26S proteasome non-ATPase regulatory subunit 11 , GN=PSMD11	11	25	11	423	47.505	6.48
Q641Q2	WASH complex subunit 2A , GN=WASHC2A	9	22	9	1341	147.095	4.81
Q9Y4G6	Talin-2 , GN=TLN2	9	21	7	2542	271.443	5.57
P49773	Histidine triad nucleotide-binding protein 1 , GN=HINT1	4	18	4	126	13.793	6.95
O43237	Cytoplasmic dynein 1 light intermediate chain 2 , GN=DYNC1LI2	5	19	5	492	54.066	6.38
P61160	Actin-related protein 2 , GN=ACTR2	7	23	2	394	44.732	6.74
Q15750	TGF-beta-activated kinase 1 and MAP3K7-binding protein 1 , GN=TAB1	6	19	6	504	54.61	5.52
Q5JWF2	Guanine nucleotide-binding protein G(s) subunit alpha isoforms XLas , GN=GNAS	4	20	4	1037	110.956	5.03
P29966	Myristoylated alanine-rich C-kinase substrate , GN=MARCKS	4	22	4	332	31.536	4.45
Q96T23	Remodeling and spacing factor 1 , GN=RSF1	6	20	6	1441	163.72	5.01
Q15369	Elongin-C , GN=ELOC	3	20	3	112	12.465	4.78
Q96K76-4	Isoform 4 of Ubiquitin carboxyl-terminal hydrolase 47 , GN=USP47	8	21	8	1355	154.6	5.06
O76003	Glutaredoxin-3 , GN=GLRX3	6	22	6	335	37.408	5.39
P62263	40S ribosomal protein S14 , GN=RPS14	4	23	4	151	16.263	10.05
P78344	Eukaryotic translation initiation factor 4 gamma 2 , GN=EIF4G2	7	21	7	907	102.297	7.14
P16152	Carbonyl reductase [NADPH] 1 , GN=CBR1	5	20	5	277	30.356	8.32
A5YKK6	CCR4-NOT transcription complex subunit 1 , GN=CNOT1	12	25	12	2376	266.768	7.11
O94979-8	Isoform 8 of Protein transport protein Sec31A , GN=SEC31A	8	23	8	1233	134.415	6.73
O14737-2	Isoform 2 of Programmed cell death protein 5 , GN=PDCD5	3	26	3	129	14.988	6.93
Q9NSK0-3	Isoform 3 of Kinesin light chain 4 , GN=KLC4	8	21	7	637	70.509	6.34
Q13573	SNW domain-containing protein 1 ,	7	22	7	536	61.456	9.52
O43776	Asparagine--tRNA ligase, cytoplasmic ,	6	21	6	548	62.903	6.25
Q9BXP5	Serrate RNA effector molecule homolog , GN=SRRT	7	23	7	876	100.604	5.96
P27695	DNA-(apurinic or apyrimidinic site) lyase , GN=APEX1	3	19	3	318	35.532	8.12
O15355	Protein phosphatase 1G , GN=PPM1G	5	21	5	546	59.235	4.36
P56192	Methionine--tRNA ligase, cytoplasmic , GN=MARS	8	20	8	900	101.052	6.16
Q02809-2	Isoform 2 of Procollagen-lysine,2-oxoglutarate 5-dioxygenase 1 , GN=PLOD1	8	25	8	774	88.217	7.11

Q16630-2	Isoform 2 of Cleavage and polyadenylation specificity factor subunit 6 , GN=CPSF6	3	18	3	588	63.432	7.71
Q9P013	Spliceosome-associated protein CWC15 homolog , GN=CWC15	2	17	2	229	26.608	5.71
O75937	DnaJ homolog subfamily C member 8 , GN=DNAJC8	4	18	4	253	29.823	9.06
P35637	RNA-binding protein FUS , GN=FUS	6	22	5	526	53.394	9.36
P07197	Neurofilament medium polypeptide ,	8	20	7	916	102.411	4.91
P28066	Proteasome subunit alpha type-5 ,	6	24	6	241	26.394	4.79
Q9Y5Z4	Heme-binding protein 2 , GN=HEBP2	3	23	3	205	22.861	4.63
O75436	Vacuolar protein sorting-associated protein 26A , GN=VPS26A	7	23	7	327	38.146	6.57
Q16531	DNA damage-binding protein 1 , GN=DDB1	7	18	7	1140	126.887	5.26
P10586	Receptor-type tyrosine-protein phosphatase F , GN=PTPRF	10	21	10	1907	212.744	6.3
Q9UKV3	Apoptotic chromatin condensation inducer in the nucleus , GN=ACIN1	5	22	5	1341	151.771	6.43
P42765	3-ketoacyl-CoA thiolase, mitochondrial , GN=ACAA2	4	20	4	397	41.898	8.09
O60341-2	Isoform 2 of Lysine-specific histone demethylase 1A , GN=KDM1A	5	20	5	876	95.096	6.25
P12268	Inosine-5'-monophosphate dehydrogenase 2 , GN=IMPDH2	9	20	9	514	55.77	6.9
P33316	Deoxyuridine 5'-triphosphate nucleotidohydrolase, mitochondrial , GN=DUT	5	21	5	252	26.547	9.36
P31153	S-adenosylmethionine synthase isoform type-2 , GN=MAT2A	3	25	3	395	43.633	6.48
P31930	Cytochrome b-c1 complex subunit 1, mitochondrial , GN=UQCRC1	9	20	9	480	52.612	6.37
P36871-2	Isoform 2 of Phosphoglucomutase-1 ,	9	21	9	580	63.75	5.83
P13693	Translationally-controlled tumor protein , GN=TPT1	4	23	4	172	19.583	4.93
Q14157-5	Isoform 5 of Ubiquitin-associated protein 2-like , GN=UBAP2L	7	23	7	1104	116.569	7.02
Q9NQX3-2	Isoform 2 of Gephyrin , GN=GPHN	6	20	6	769	83.395	5.58
O14979	Heterogeneous nuclear ribonucleoprotein D-like , GN=HNRNPDL	6	21	6	420	46.409	9.57
P55145	Mesencephalic astrocyte-derived neurotrophic factor , GN=MANF	3	19	3	182	20.687	8.69
P49790	Nuclear pore complex protein Nup153 , GN=NUP153	6	20	6	1475	153.843	8.73
O75122-3	Isoform 3 of CLIP-associating protein 2 , GN=CLASP2	8	22	8	1515	165.821	8.37
Q6P2E9	Enhancer of mRNA-decapping protein 4 , GN=EDC4	7	19	7	1401	151.567	5.86
P35080	Profilin-2 , GN=PFN2	4	21	4	140	15.036	6.99
P04181	Ornithine aminotransferase, mitochondrial , GN=OAT	8	22	8	439	48.504	7.03
Q93009	Ubiquitin carboxyl-terminal hydrolase 7 , GN=USP7	7	21	7	1102	128.22	5.55
Q01658	Protein Dr1 , GN=DR1	3	18	3	176	19.432	4.75
Q9Y5B9	FACT complex subunit SPT16 , GN=SUPT16H	10	20	10	1047	119.838	5.66
O00764	Pyridoxal kinase , GN=PDXK	6	22	6	312	35.08	6.13
O60502	Protein O-GlcNAcase , GN=MGEA5	10	21	10	916	102.849	4.91
P48637	Glutathione synthetase , GN=GSS	5	19	5	474	52.352	5.92
Q9BZZ5-2	Isoform 2 of Apoptosis inhibitor 5 , GN=API5	6	19	6	504	56.735	5.92
P51812	Ribosomal protein S6 kinase alpha-3 , GN=RPS6KA3	6	21	5	740	83.683	6.89
Q13126	S-methyl-5'-thioadenosine phosphorylase , GN=MTAP	4	21	4	283	31.216	7.18
Q15008-4	Isoform 4 of 26S proteasome non-ATPase regulatory subunit 6 , GN=PSMD6	8	24	8	442	51.889	6.74
P35237	Serpin B6 , GN=SERPINB6	9	20	9	376	42.594	5.27
P30837	Aldehyde dehydrogenase X, mitochondrial , GN=ALDH1B1	4	22	4	517	57.17	6.8
P52789	Hexokinase-2 , GN=HK2	5	24	3	917	102.313	6.05

Q07812-2	Isoform Beta of Apoptosis regulator BAX , GN=BAX	6	22	6	218	24.204	7.9
Q12840	Kinesin heavy chain isoform 5A , GN=KIF5A	7	23	1	1032	117.306	5.9
P55036	26S proteasome non-ATPase regulatory subunit 4 , GN=PSMD4	5	23	3	377	40.711	4.79
Q8WWY3	U4/U6 small nuclear ribonucleoprotein Prp31 , GN=PRPF31	7	19	7	499	55.421	5.78
Q9UL46	Proteasome activator complex subunit 2 , GN=PSME2	5	18	5	239	27.384	5.73
Q9BUJ2	Heterogeneous nuclear ribonucleoprotein U-like protein 1 , GN=HNRNPUL1	6	19	6	856	95.679	6.92
O43242	26S proteasome non-ATPase regulatory subunit 3 , GN=PSMD3	6	21	6	534	60.939	8.44
P27708	CAD protein , GN=CAD	8	19	8	2225	242.829	6.46
O00299	Chloride intracellular channel protein 1 , GN=CLIC1	5	21	5	241	26.906	5.17
P53992	Protein transport protein Sec24C ,	8	21	8	1094	118.249	7.06
Q9H0R4	Haloacid dehalogenase-like hydrolase domain-containing protein 2 , GN=HDHD2	4	17	4	259	28.518	6.24
Q9Y5Y2	Cytosolic Fe-S cluster assembly factor NUBP2 , GN=NUBP2	2	16	2	271	28.807	5.83
P58546	Myotrophin , GN=MTPN	5	17	5	118	12.887	5.52
Q8TAQ2	SWI/SNF complex subunit SMARCC2 , GN=SMARCC2	10	22	8	1214	132.797	5.69
O95831	Apoptosis-inducing factor 1, mitochondrial , GN=AIFM1	7	22	7	613	66.859	8.95
P31040	flavoprotein subunit, mitochondrial , GN=SDHA	7	20	7	664	72.645	7.39
P07942	Laminin subunit beta-1 , GN=LAMB1	7	19	7	1786	197.909	4.94
Q08257	Quinone oxidoreductase , GN=CRYZ	3	19	3	329	35.185	8.44
P17858	ATP-dependent 6-phosphofructokinase, liver type , GN=PFKL SV=6	4	17	3	780	84.964	7.5
P23921	Ribonucleoside-diphosphate reductase large subunit , GN=RRM1	7	20	7	792	90.013	7.15
O95232	Luc7-like protein 3 , GN=LUC7L3	3	19	3	432	51.435	9.79
Q13428-4	Isoform 4 of Treacle protein , GN=TCOF1	11	21	11	1524	155.836	8.95
P41252	Isoleucine--tRNA ligase, cytoplasmic ,	9	20	9	1262	144.406	6.15
P43034	Platelet-activating factor acetylhydrolase IB subunit alpha , GN=PAFAH1B1	6	20	6	410	46.608	7.37
P51532	Transcription activator BRG1 , GN=SMARCA4	11	20	11	1647	184.53	7.88
P16989	Y-box-binding protein 3 , GN=YBX3	4	19	1	372	40.066	9.77
P22059	Oxysterol-binding protein 1 , GN=OSBP	4	16	4	807	89.365	7.3
P14324	Farnesyl pyrophosphate synthase , GN=FDPS	6	19	6	419	48.245	6.15
P52292	Importin subunit alpha-1 , GN=KPNA2	4	20	4	529	57.826	5.4
O95202	Mitochondrial proton/calcium exchanger protein , GN=LETM1	5	20	5	739	83.302	6.7
Q13162	Peroxiredoxin-4 , GN=PRDX4	5	20	5	271	30.521	6.29
P38919	Eukaryotic initiation factor 4A-III , GN=EIF4A3	6	20	5	411	46.841	6.73
Q9NX40	OCIA domain-containing protein 1 ,	1	18	1	245	27.609	7.49
Q9ULA0	Aspartyl aminopeptidase , GN=DNPEP	3	16	3	475	52.395	7.42
P26196	Probable ATP-dependent RNA helicase DDX6 , GN=DDX6	10	18	10	483	54.382	8.66
Q9UBQ7	Glyoxylate reductase/hydroxypyruvate reductase , GN=GRHPR	4	19	4	328	35.646	7.39
Q99598	Translin-associated protein X , GN=TSNAX	2	18	2	290	33.092	6.55
Q92973-2	Isoform 2 of Transportin-1 , GN=TNPO1	9	22	7	890	101.244	4.96
Q16718	NADH dehydrogenase [ubiquinone] 1 alpha subcomplex subunit 5 , GN=NDUFA5	3	14	3	116	13.45	5.99
P12004	Proliferating cell nuclear antigen , GN=PCNA	6	19	6	261	28.75	4.69
Q08380	Galectin-3-binding protein , GN=LGALS3BP	6	20	6	585	65.289	5.27
Q96JB5-4	Isoform 4 of CDK5 regulatory subunit-associated protein 3 , GN=CDK5RAP3	3	20	3	531	59.551	4.92
P84085	ADP-ribosylation factor 5 , GN=ARF5	4	20	2	180	20.517	6.79
Q02818	Nucleobindin-1 , GN=NUCB1	9	20	9	461	53.846	5.25
O60313-2	Isoform 2 of Dynamin-like 120 kDa protein, mitochondrial , GN=OPA1	7	19	7	997	115.812	7.87

P42126	Enoyl-CoA delta isomerase 1, mitochondrial , GN=ECI1	3	22	3	302	32.795	8.54	
P68036-3	Isoform 3 of Ubiquitin-conjugating enzyme E2 L3 , GN=UBE2L3	4	19	4	212	23.989	8.75	
P49750-4	Isoform 4 of YLP motif-containing protein 1 , GN=YLPM1	14	23	14	2146	241.495	6.55	
Q9BPW8	Protein NipSnap homolog 1 , GN=NIPSNAP1	6	20	6	284	33.289	9.31	
P10155	60 kDa SS-A/Ro ribonucleoprotein ,	8	21	8	538	60.631	8.03	
Q02218	2-oxoglutarate dehydrogenase, mitochondrial , GN=OGDH	7	20	7	1023	115.861	6.86	
Q9NTJ3	Structural maintenance of chromosomes protein 4 , GN=SMC4	8	18	8	1288	147.091	6.79	
P23434	Glycine cleavage system H protein, mitochondrial , GN=GCSH	1	17	1	173	18.873	4.88	
Q9Y678	Coatomer subunit gamma-1 , GN=COPG1	6	19	5	874	97.655	5.47	
O00232	26S proteasome non-ATPase regulatory subunit 12 , GN=PSMD12	7	20	7	456	52.871	7.65	
P09661	U2 small nuclear ribonucleoprotein A' , GN=SNRPA1	6	19	6	255	28.398	8.62	
O00303	Eukaryotic translation initiation factor 3 subunit F , GN=EIF3F	5	16	5	357	37.54	5.45	
P30050	60S ribosomal protein L12 , GN=RPL12	2	16	2	165	17.808	9.42	
P41208	Centrin-2 , GN=CETN2	4	21	4	172	19.726		5
P60900	Proteasome subunit alpha type-6 ,	5	19	5	246	27.382	6.76	
P13798	Acylamino-acid-releasing enzyme , GN=APEH	4	22	4	732	81.173	5.48	
P20674	Cytochrome c oxidase subunit 5A, mitochondrial , GN=COX5A	3	18	3	150	16.752	6.79	
P11766	Alcohol dehydrogenase class-3 , GN=ADH5	3	16	3	374	39.698	7.49	
P55809	Succinyl-CoA:3-ketoacid coenzyme A transferase 1, mitochondrial , GN=OXCT1	5	17	5	520	56.122	7.46	
Q9NR45	Sialic acid synthase , GN=NANS	5	20	5	359	40.281	6.74	
Q8WWM7-3	Isoform 3 of Ataxin-2-like protein ,	7	18	7	1097	115.511	8.66	
Q7KZ85	Transcription elongation factor SPT6 , GN=SUPT6H	8	18	8	1726	198.949	4.91	
Q3LXA3	Triokinase/FMN cyclase , GN=TKFC	7	19	7	575	58.91	7.49	
Q9BX68	Histidine triad nucleotide-binding protein 2, mitochondrial , GN=HINT2	4	18	4	163	17.151	9.16	
Q9Y266	Nuclear migration protein nudC , GN=NUDC	8	21	8	331	38.219	5.38	
Q15691	Microtubule-associated protein RP/EB family member 1 , GN=MAPRE1	5	18	5	268	29.98	5.14	
O75367	Core histone macro-H2A.1 , GN=H2AFY	7	17	6	372	39.592	9.79	
Q9ULC4-3	Isoform 3 of Malignant T-cell-amplified sequence 1 , GN=MCTS1	2	18	2	182	20.537	8.82	
P61604	10 kDa heat shock protein, mitochondrial , GN=HSPE1	4	21	4	102	10.925	8.92	
P23258	Tubulin gamma-1 chain , GN=TUBG1	6	16	6	451	51.138	6.14	
P02771	Alpha-fetoprotein , GN=AFP	6	18	5	609	68.633	5.68	
Q6UN15	Pre-mRNA 3'-end-processing factor FIP1 , GN=FIP1L1	6	18	6	594	66.487	5.59	
P30042	ES1 protein homolog, mitochondrial , GN=C21orf33	4	16	4	268	28.153	8.27	
Q13177	Serine/threonine-protein kinase PAK 2 , GN=PAK2	7	18	5	524	58.006	5.96	
O75323	Protein NipSnap homolog 2 , GN=NIPSNAP2	3	18	3	286	33.721	9.36	
Q15121-2	Isoform 2 of Astrocytic phosphoprotein PEA-15 , GN=PEA15	4	19	4	151	17.296	4.89	
Q9Y6I3-1	Isoform 2 of Epsin-1 , GN=EPN1	5	16	5	662	68.996	5.05	
P09622	Dihydrolipoyl dehydrogenase, mitochondrial , GN=DLD	5	19	5	509	54.143	7.85	
Q9NQG5	Regulation of nuclear pre-mRNA domain-containing protein 1B , GN=RPRD1B	6	18	5	326	36.877	5.97	
A6NIZ1	Ras-related protein Rap-1b-like protein , PE=2	3	18	3	184	20.912	5.48	
Q04637-9	Isoform 9 of Eukaryotic translation initiation factor 4 gamma 1 , GN=EIF4G1	7	19	6	1606	176.128	5.34	
Q9UNF1	Melanoma-associated antigen D2 ,	6	19	6	606	64.914	9.32	
Q9UNS2	COP9 signalosome complex subunit 3 , GN=COPS3	3	16	3	423	47.842	6.65	

P36405	ADP-ribosylation factor-like protein 3 ,	5	17	5	182	20.443	7.24
P55884-2	Isoform 2 of Eukaryotic translation initiation factor 3 subunit B , GN=EIF3B	9	19	9	873	98.967	5.08
Q14683	Structural maintenance of chromosomes protein 1A , GN=SMC1A	7	19	7	1233	143.144	7.64
Q9BV38	WD repeat-containing protein 18 ,	5	16	5	432	47.375	6.7
Q96I24	Far upstream element-binding protein 3 , GN=FUBP3	4	16	4	572	61.602	8.38
Q9HCM4	Band 4.1-like protein 5 , GN=EPB41L5	6	18	6	733	81.805	6.58
P57737-3	Isoform 3 of Coronin-7 , GN=CORO7	6	18	6	1048	114.095	6.33
P55265-4	Isoform 4 of Double-stranded RNA-specific adenosine deaminase , GN=ADAR	7	18	7	1269	140.749	8.56
P27694	Replication protein A 70 kDa DNA-binding subunit , GN=RPA1	8	17	8	616	68.095	7.21
Q96AY3	Peptidyl-prolyl cis-trans isomerase FKBP10 , GN=FKBP10	6	17	6	582	64.204	5.62
P19174-2	Isoform 2 of 1-phosphatidylinositol 4,5-bisphosphate phosphodiesterase gamma-1 , GN=PLCG1	5	19	5	1291	148.566	6.05
P53004	Biliverdin reductase A , GN=BLVRA	6	18	6	296	33.407	6.44
P05198	Eukaryotic translation initiation factor 2 subunit 1 , GN=EIF2S1	5	17	5	315	36.089	5.08
Q14444	Caprin-1 , GN=CAPRIN1	5	18	5	709	78.318	5.25
Q9NQ48	Leucine zipper transcription factor-like protein 1 , GN=LZTFL1	8	18	8	299	34.571	5.36
O15067	Phosphoribosylformylglycinamidine synthase , GN=PFAS	6	15	6	1338	144.643	5.76
P07196	Neurofilament light polypeptide , GN=NEFL	6	18	6	543	61.479	4.65
Q96DH6	RNA-binding protein Musashi homolog 2 , GN=MSI2	5	18	5	328	35.174	8.48
Q9BUT1	3-hydroxybutyrate dehydrogenase type 2 , GN=BDH2	4	16	4	245	26.707	7.65
P14868	Aspartate--tRNA ligase, cytoplasmic ,	6	17	6	501	57.1	6.55
Q86XP3	ATP-dependent RNA helicase DDX42 , GN=DDX42	7	18	7	938	102.912	7.02
Q6P587	Acylpyruvase FAHD1, mitochondrial ,	3	15	3	224	24.827	7.39
P07384	Calpain-1 catalytic subunit , GN=CAPN1	7	16	7	714	81.838	5.67
P43490	Nicotinamide phosphoribosyltransferase , GN=NAMPT	6	14	6	491	55.487	7.15
P16435	NADPH--cytochrome P450 reductase ,	4	15	4	677	76.641	5.58
P12694	2-oxoisovalerate dehydrogenase subunit alpha, mitochondrial , GN=BCKDHA	4	14	4	445	50.439	8.27
Q99471	Prefoldin subunit 5 , GN=PFDN5	4	15	4	154	17.317	6.33
Q9NRF8	CTP synthase 2 , GN=CTPS2	8	17	6	586	65.636	6.9
Q92859	Neogenin , GN=NEO1	5	16	5	1461	159.917	6.54
P39060	Collagen alpha-1(XVIII) chain , GN=COL18A1	4	16	4	1754	178.077	6.01
Q92783	Signal transducing adapter molecule 1 , GN=STAM	7	18	7	540	59.142	4.82
P00441	Superoxide dismutase [Cu-Zn] , GN=SOD1	2	19	2	154	15.926	6.13
Q13618	Cullin-3 , GN=CUL3	5	14	5	768	88.873	8.48
Q9Y2J2-2	Isoform 2 of Band 4.1-like protein 3 , GN=EPB41L3	7	18	6	865	96.454	5.43
Q6NUK1	Calcium-binding mitochondrial carrier protein SCaMC-1 , GN=SLC25A24	6	16	6	477	53.32	6.33
Q9P2R3-4	Isoform 4 of Rabankyrin-5 , GN=ANKFY1	4	14	4	1211	132.737	6.49
Q13363	C-terminal-binding protein 1 , GN=CTBP1	4	16	4	440	47.505	6.77
Q15287	RNA-binding protein with serine-rich domain 1 , GN=RNPS1	3	15	3	305	34.188	11.84
P32322-3	Isoform 3 of Pyrroline-5-carboxylate reductase 1, mitochondrial , GN=PYCR1	5	16	5	346	35.958	7.61
Q96CX2	BTB/POZ domain-containing protein KCTD12 , GN=KCTD12	5	18	5	325	35.679	5.64
Q15181	Inorganic pyrophosphatase , GN=PPA1	7	17	7	289	32.639	5.86
Q92905	COP9 signalosome complex subunit 5 , GN=COPS5	5	18	5	334	37.555	6.54
Q92922	SWI/SNF complex subunit SMARCC1 , GN=SMARCC1	7	18	5	1105	122.79	5.76

Q969G3	SWI/SNF-related matrix-associated actin-dependent regulator of chromatin subfamily E member 1 , GN=SMARCE1	6	16	6	411	46.621	4.88
P22102	Trifunctional purine biosynthetic protein adenosine-3 , GN=GART	7	16	7	1010	107.699	6.7
P25788	Proteasome subunit alpha type-3 ,	5	20	5	255	28.415	5.33
P19022	Cadherin-2 , GN=CDH2	5	15	5	906	99.747	4.81
Q15435	Protein phosphatase 1 regulatory subunit 7 , GN=PPP1R7	5	15	5	360	41.539	4.91
Q13242	Serine/arginine-rich splicing factor 9 ,	5	20	5	221	25.526	8.65
P63151-2	Isoform 2 of Serine/threonine-protein phosphatase 2A 55 kDa regulatory subunit B alpha isoform , GN=PPP2R2A	6	16	6	457	52.966	6.49
O95486	Protein transport protein Sec24A ,	6	17	5	1093	119.674	7.66
P35659	Protein DEK , GN=DEK	5	18	5	375	42.648	8.56
P07602-3	Isoform Sap-mu-9 of Prosaposin , GN=PSAP	1	18	1	527	58.445	5.14
Q9HDC9	Adipocyte plasma membrane-associated protein , GN=APMAP	4	15	4	416	46.451	6.16
Q99961	Endophilin-A2 , GN=SH3GL1	5	14	5	368	41.464	5.43
Q15075	Early endosome antigen 1 , GN=EEA1	8	15	8	1411	162.367	5.68
Q9H0Q0	Protein FAM49A , GN=FAM49A PE=2	2	18	1	323	37.289	6.01
O15347	High mobility group protein B3 , GN=HMGB3	7	16	7	200	22.965	8.37
P61160-2	Isoform 2 of Actin-related protein 2 ,	6	16	1	399	45.348	7.2
Q8TAT6-2	Isoform 2 of Nuclear protein localization protein 4 homolog , GN=NPLOC4	4	15	4	617	69.417	6.68
Q99459	Cell division cycle 5-like protein , GN=CDC5L	10	16	10	802	92.194	8.18
P47985	Cytochrome b-c1 complex subunit Rieske, mitochondrial , GN=UQCRFS1	3	14	3	274	29.649	8.32
Q9UKX7	Nuclear pore complex protein Nup50 , GN=NUP50	4	13	4	468	50.113	7.06
Q13796	Protein Shroom2 , GN=SHROOM2	9	16	9	1616	176.303	7.09
O75306	NADH dehydrogenase [ubiquinone] iron-sulfur protein 2, mitochondrial , GN=NDUFS2	5	17	5	463	52.512	7.55
Q14566	DNA replication licensing factor MCM6 , GN=MCM6	9	17	9	821	92.831	5.41
Q3YEC7-2	Isoform 2 of Rab-like protein 6 , GN=RABL6	3	17	3	730	79.587	5.22
Q9BT78	COP9 signalosome complex subunit 4 , GN=COPS4	6	15	6	406	46.24	5.83
P34897	Serine hydroxymethyltransferase, mitochondrial , GN=SHMT2	9	17	8	504	55.958	8.53
O00148	ATP-dependent RNA helicase DDX39A , GN=DDX39A	6	17	1	427	49.098	5.68
P20962	Parathymosin , GN=PTMS	2	20	2	102	11.523	4.16
P35658-3	Isoform 3 of Nuclear pore complex protein Nup214 , GN=NUP214	5	16	5	2091	213.559	7.47
P00390	Glutathione reductase, mitochondrial ,	4	14	4	522	56.221	8.5
Q12904-2	Isoform 2 of Aminoacyl tRNA synthase complex-interacting multifunctional protein 1 , GN=AIMP1	5	17	5	336	37.016	8.65
Q8NCW5	NAD(P)H-hydrate epimerase , GN=NAXE	3	16	3	288	31.654	7.66
Q9H910-3	Isoform 3 of Jupiter microtubule associated homolog 2 , GN=JPT2	3	17	3	218	23.012	9.57
P24666	Low molecular weight phosphotyrosine protein phosphatase , GN=ACP1	4	15	4	158	18.031	6.74
Q14011	Cold-inducible RNA-binding protein ,	5	16	5	172	18.637	9.51
Q9UN86	Ras GTPase-activating protein-binding protein 2 , GN=G3BP2	6	15	5	482	54.088	5.55
Q12996	Cleavage stimulation factor subunit 3 , GN=CSTF3	7	15	7	717	82.869	8.12
P24534	Elongation factor 1-beta , GN=EEF1B2	3	14	3	225	24.748	4.67
Q13564-4	Isoform 4 of NEDD8-activating enzyme E1 regulatory subunit , GN=NAE1	6	15	6	537	60.479	5.4
Q9H4A4	Aminopeptidase B , GN=RNPEP	6	15	6	650	72.549	5.74
Q92878-2	Isoform 2 of DNA repair protein RAD50 , GN=RAD50	6	15	6	1318	154.491	6.89
Q9UEY8	Gamma-adducin , GN=ADD3	6	16	6	706	79.105	6.32
Q14112	Nidogen-2 , GN=NID2	5	13	5	1375	151.158	5.29

Q96QK1	Vacuolar protein sorting-associated protein 35 , GN=VPS35	5	17	5	796		91.649	5.49	
P17050	Alpha-N-acetylgalactosaminidase , GN=NAGA	4	15	4	411		46.534	5.19	
P36543	V-type proton ATPase subunit E 1 ,	2	16	2	226		26.129		8
O14579	Coatomer subunit epsilon , GN=COPE	6	15	6	308	34.46		5.12	
Q9NQW7-3	Isoform 3 of Xaa-Pro aminopeptidase 1 , GN=XPNPEP1	7	16	7	666		74.751	5.88	
Q96CN7	Isochorismatase domain-containing protein 1 , GN=ISOC1	3	15	3	298		32.216	7.39	
Q9Y285	Phenylalanine--tRNA ligase alpha subunit , GN=FARSA	5	15	5	508		57.528	7.8	
Q15063	Periostin , GN=POSTN	7	15	7	836		93.255	7.53	
P42785-2	Isoform 2 of Lysosomal Pro-X carboxypeptidase , GN=PRCP	3	15	3	517		58.062	7.4	
P46459	Vesicle-fusing ATPase , GN=NSF	7	16	7	744		82.542	6.95	
Q13547	Histone deacetylase 1 , GN=HDAC1	5	16	2	482		55.068	5.48	
P23786	Carnitine O-palmitoyltransferase 2, mitochondrial , GN=CPT2	7	15	7	658	73.73		8.18	
P49736	DNA replication licensing factor MCM2 , GN=MCM2	8	15	8	904		101.832	5.52	
P47897	Glutamine--tRNA ligase , GN=QARS	8	16	8	775		87.743	7.15	
Q13641	Trophoblast glycoprotein , GN=TPBG	2	16	2	420		46.003	6.83	
Q16543	Hsp90 co-chaperone Cdc37 , GN=CDC37	4	15	4	378	44.44		5.25	
P61106	Ras-related protein Rab-14 , GN=RAB14	6	17	5	215		23.882	6.21	
Q6STE5	SWI/SNF-related matrix-associated actin-dependent regulator of chromatin subfamily D member 3 , GN=SMARCD3	4	15	3	483		54.981	9.35	
Q12765-2	Isoform 2 of Secernin-1 , GN=SCRN1	5	16	1	434		48.683	4.78	
Q12765-3	Isoform 3 of Secernin-1 , GN=SCRN1	5	16	1	346		38.839	4.7	
Q13510-2	Isoform 2 of Acid ceramidase , GN=ASAH1	4	13	4	411		46.474	7.85	
Q02878	60S ribosomal protein L6 , GN=RPL6	2	18	2	288		32.708	10.58	
Q01813	ATP-dependent 6-phosphofructokinase, platelet type , GN=PFKP	4	16	3	784		85.542	7.55	
P68402	Platelet-activating factor acetylhydrolase IB subunit beta , GN=PAFAH1B2	2	13	2	229		25.553	5.92	
P00568	Adenylate kinase isoenzyme 1 , GN=AK1	3	18	3	194		21.621	8.63	
Q9Y2W2	WW domain-binding protein 11 , GN=WBP11	6	14	6	641		69.954	8.38	
P10515	Dihydrolipoyllysine-residue acetyltransferase component of pyruvate dehydrogenase complex, mitochondrial , GN=DLAT	5	14	5	647		68.953	7.84	
Q9HCC0	Methylcrotonoyl-CoA carboxylase beta chain, mitochondrial , GN=MCCC2	4	14	4	563		61.294	7.68	
Q8NC51	Plasminogen activator inhibitor 1 RNA-binding protein , GN=SERBP1	5	14	5	408		44.938	8.65	
P35249	Replication factor C subunit 4 , GN=RFC4	4	12	4	363		39.657	8.02	
P63279	SUMO-conjugating enzyme UBC9 , GN=UBE2I	5	16	5	158		17.995	8.66	
P13591	Neural cell adhesion molecule 1 , GN=NCAM1	5	17	5	858		94.515	4.87	
Q9NTZ6	RNA-binding protein 12 , GN=RBM12	5	17	5	932		97.333	8.63	
P49189	4-trimethylaminobutyraldehyde dehydrogenase , GN=ALDH9A1	4	18	4	494		53.767	5.87	
P25789	Proteasome subunit alpha type-4 ,	2	17	2	261		29.465	7.72	
O00267	Transcription elongation factor SPT5 , GN=SUPT5H	8	15	8	1087		120.925	5.06	
O60216	Double-strand-break repair protein rad21 homolog , GN=RAD21	5	14	5	631		71.645	4.65	
Q8WVM8	Sec1 family domain-containing protein 1 , GN=SCFD1	7	14	7	642		72.334	6.27	
P80723	Brain acid soluble protein 1 , GN=BASP1	6	16	6	227	22.68		4.63	
P26583	High mobility group protein B2 , GN=HMGB2	6	17	5	209		24.019	7.81	
Q9NVA2-2	Isoform 2 of Septin-11 , GN=SEPT11	7	16	3	439		50.791	7.01	
P14859-6	Isoform 6 of POU domain, class 2, transcription factor 1 , GN=POU2F1	4	13	4	766		78.487	6.11	
Q08623-4	Isoform 4 of Pseudouridine-5'-phosphatase , GN=PUDP	9	15	9	251		27.555	5.47	
Q15257	Serine/threonine-protein phosphatase 2A activator , GN=PTPA	4	14	4	358		40.641	5.94	

P23634	Plasma membrane calcium-transporting ATPase 4 , GN=ATP2B4	6	14	4	1241		137.833	6.6
Q15631	Translin , GN=TSN	6	15	6	228		26.167	6.44
Q9UN36-6	Isoform 6 of Protein NDRG2 , GN=NDRG2	4	11	4	367		40.271	5.29
Q92797	Symplekin , GN=SYMPK	6	14	6	1274		141.059	6.13
Q15334	Lethal(2) giant larvae protein homolog 1 , GN=LLGL1	10	16	10	1064		115.346	6.29
P12236	ADP/ATP translocase 3 , GN=SLC25A6	4	17	1	298		32.845	9.74
P63220	40S ribosomal protein S21 , GN=RPS21	5	14	5	83		9.106	8.5
Q9NVE7	Pantothenate kinase 4 , GN=PANK4	8	15	8	773		85.937	6.28
P62306	Small nuclear ribonucleoprotein F ,	1	15	1	86		9.719	4.67
P30566	Adenylosuccinate lyase , GN=ADSL	4	13	4	484		54.854	7.11
O75179	Ankyrin repeat domain-containing protein 17 , GN=ANKRD17	5	13	2	2603		274.088	6.52
Q92621	Nuclear pore complex protein Nup205 , GN=NUP205	6	13	6	2012		227.776	6.19
Q93008	Probable ubiquitin carboxyl-terminal hydrolase FAF-X , GN=USP9X	10	16	10	2570		292.094	5.8
Q7LBC6	Lysine-specific demethylase 3B , GN=KDM3B	7	16	7	1761		191.461	7.18
P49006	MARCKS-related protein , GN=MARCKSL1	2	15	2	195		19.517	4.67
P30038	dehydrogenase, mitochondrial , GN=ALDH4A1	4	13	4	563		61.681	8.07
Q9Y263	Phospholipase A-2-activating protein ,	6	15	6	795		87.101	6.37
O60256	Phosphoribosyl pyrophosphate synthase-associated protein 2 , GN=PRPSAP2	6	16	5	369		40.899	7.44
Q9BR76	Coronin-1B , GN=CORO1B	3	13	3	489	54.2		5.88
Q96AB3-2	Isoform 2 of Isochorismatase domain-containing protein 2 , GN=ISOC2	3	12	3	221		24.082	7.77
P36957	succinyltransferase component of 2-oxoglutarate dehydrogenase complex, mitochondrial , GN=DLST	3	12	3	453		48.724	8.95
Q14847-2	Isoform 2 of LIM and SH3 domain protein 1 , GN=LASP1	6	17	6	323		35.991	8.62
P11216	Glycogen phosphorylase, brain form ,	8	15	8	843		96.635	6.86
Q8NBX0	Saccharopine dehydrogenase-like oxidoreductase , GN=SCCPDH	6	14	6	429		47.121	9.14
P63244	Receptor of activated protein C kinase 1 , GN=RACK1	6	14	6	317		35.055	7.69
P05386	60S acidic ribosomal protein P1 , GN=RPLP1	1	17	1	114		11.507	4.32
Q9BSD7	Cancer-related nucleoside-triphosphatase , GN=NTPCR	4	15	4	190	20.7		9.54
P46783	40S ribosomal protein S10 , GN=RPS10	3	13	3	165		18.886	10.15
P35613	Basigin , GN=BSG	4	15	4	385		42.174	5.66
Q13907-2	Isoform 2 of Isopentenyl-diphosphate Delta-isomerase 1 , GN=IDI1	4	15	4	284		32.465	7.88
Q10713	Mitochondrial-processing peptidase subunit alpha , GN=PMPCA	5	16	5	525		58.216	6.92
Q5VYK3	Proteasome-associated protein ECM29 homolog , GN=ECM29	9	15	9	1845	204.16		7.12
P25786-2	Isoform Long of Proteasome subunit alpha type-1 , GN=PSMA1	6	17	6	269	30.22		6.99
P11532	Dystrophin , GN=DMD	8	15	4	3685		426.484	5.88
Q16629	Serine/arginine-rich splicing factor 7 ,	4	16	3	238	27.35		11.82
P39687	Acidic leucine-rich nuclear phosphoprotein 32 family member A , GN=ANP32A	3	16	1	249		28.568	4.09
P30043	Flavin reductase (NADPH) , GN=BLVRB	3	15	3	206		22.105	7.65
P62328	Thymosin beta-4 , GN=TMSB4X	3	16	3	44	5.05		5.06
Q15942	Zyxin , GN=ZYX	4	14	4	572		61.238	6.67
O75489	NADH dehydrogenase [ubiquinone] iron-sulfur protein 3, mitochondrial , GN=NDUFS3	6	16	6	264		30.223	7.5
Q9UBS4	DnaJ homolog subfamily B member 11 , GN=DNAJB11	4	14	4	358		40.489	6.18
P46937-9	Isoform 9 of Transcriptional coactivator YAP1 , GN=YAP1	4	13	4	508		54.908	5.24
P32969	60S ribosomal protein L9 , GN=RPL9	4	14	4	192	21.85		9.95
P53990	IST1 homolog , GN=IST1	4	14	4	364		39.725	5.35
Q9UHG3	Prenylcysteine oxidase 1 , GN=PCYOX1	6	17	6	505		56.604	6.18

Q9UBP9	PTB domain-containing engulfment adapter protein 1 , GN=GULP1	4	15	4	304		34.468	7.9
P33240	Cleavage stimulation factor subunit 2 , GN=CSTF2	6	14	3	577	60.92		6.83
Q14008-3	Isoform 3 of Cytoskeleton-associated protein 5 , GN=CKAP5	11	15	11	2039		226.114	7.77
O00192	Armadillo repeat protein deleted in velo-cardio-facial syndrome , GN=ARVCF	5	15	5	962		104.577	6.81
O75083	WD repeat-containing protein 1 , GN=WDR1	3	13	3	606		66.152	6.65
Q9NWU2	Glucose-induced degradation protein 8 homolog , GN=GID8	5	14	5	228		26.732	4.97
Q9BY77	Polymerase delta-interacting protein 3 , GN=POLDIP3	4	14	4	421	46.06		9.99
Q8WXH0-2	Isoform 2 of Nesprin-2 , GN=SYNE2	10	14	10	6907		798.362	5.36
P63208	S-phase kinase-associated protein 1 ,	7	14	7	163		18.646	4.54
Q9H2U2-2	Isoform 2 of Inorganic pyrophosphatase 2, mitochondrial , GN=PPA2	6	15	6	349		39.613	6.92
Q9UHY1	Nuclear receptor-binding protein , GN=NRBP1	5	14	5	535		59.807	5.08
P12081	Histidine--tRNA ligase, cytoplasmic ,	4	13	4	509		57.374	5.88
O60884	DnaJ homolog subfamily A member 2 , GN=DNAJA2	5	13	5	412		45.717	6.48
Q9Y383	Putative RNA-binding protein Luc7-like 2 , GN=LUC7L2	4	14	3	392		46.486	10.01
Q14558-2	Isoform 2 of Phosphoribosyl pyrophosphate synthase-associated protein 1 , GN=PRPSAP1	4	16	3	385	42.44		8.47
P16930	Fumarylacetoacetase , GN=FAH	4	14	4	419		46.344	6.95
P46777	60S ribosomal protein L5 , GN=RPL5	5	16	5	297		34.341	9.72
Q01844-5	Isoform 5 of RNA-binding protein EWS , GN=EWSR1	5	15	5	661		68.923	9.29
Q14126	Desmoglein-2 , GN=DSG2	4	16	4	1118		122.218	5.24
O94874	E3 UFM1-protein ligase 1 , GN=UFL1	8	15	8	794	89.54		6.79
Q7L576	Cytoplasmic FMR1-interacting protein 1 , GN=CYFIP1	9	17	5	1253		145.089	6.9
P05141	ADP/ATP translocase 2 , GN=SLC25A5 SV=7	4	16	1	298		32.831	9.69
Q9UM54-6	Isoform 6 of Unconventional myosin-VI , GN=MYO6	5	12	5	1285		148.565	8.56
P60228	Eukaryotic translation initiation factor 3 subunit E , GN=EIF3E	3	12	3	445		52.187	6.04
O94973-2	Isoform 2 of AP-2 complex subunit alpha-2 , GN=AP2A2	8	13	7	940		104.024	6.86
Q8WZA0-2	Isoform 2 of Protein LZIC , GN=LZIC	3	12	3	211	23.91		5.11
Q99873	Protein arginine N-methyltransferase 1 , GN=PRMT1	4	14	4	361		41.489	5.43
O94903	Pyridoxal phosphate homeostasis protein , GN=PROSC	2	15	2	275		30.325	7.5
Q13308-6	Isoform 6 of Inactive tyrosine-protein kinase 7 , GN=PTK7	3	14	3	1078		119.122	6.96
Q13451	Peptidyl-prolyl cis-trans isomerase FKBP5 , GN=FKBP5	5	15	5	457	51.18		5.9
Q9BWF3	RNA-binding protein 4 , GN=RBM4	5	12	2	364		40.289	7.08
Q92558	Wiskott-Aldrich syndrome protein family member 1 , GN=WASF1	4	14	4	559		61.614	6.46
Q9P289	Serine/threonine-protein kinase 26 ,	3	13	2	416	46.5		5.29
P21266	Glutathione S-transferase Mu 3 , GN=GSTM3	3	12	3	225		26.542	5.54
Q13228-4	Isoform 4 of Selenium-binding protein 1 , GN=SELENBP1	7	15	7	514	56.83		6.48
P55196-1	Isoform 2 of Afadin , GN=AFDN	8	14	8	1816		205.478	6.77
P48047	ATP synthase subunit O, mitochondrial , GN=ATP5O	5	16	5	213		23.263	9.96
Q9NZZ3	Charged multivesicular body protein 5 , GN=CHMP5	5	13	5	219		24.555	4.83
P11182	branched-chain alpha-keto acid dehydrogenase complex, mitochondrial , GN=DBT	2	13	2	482		53.453	8.51
Q96C90	Protein phosphatase 1 regulatory subunit 14B , GN=PPP1R14B	3	13	3	147		15.901	4.86
Q9Y3B4	Splicing factor 3B subunit 6 , GN=SF3B6	2	15	2	125		14.576	9.38

Q5TZA2	Rootletin , GN=CROCC	8	13	8	2017	228.388	5.5
Q32P28-3	Isoform 3 of Prolyl 3-hydroxylase 1 ,	4	14	4	804	90.559	5.71
Q8N8N7	Prostaglandin reductase 2 , GN=PTGR2	6	14	6	351	38.474	5.41
Q9BXS5-2	Isoform 2 of AP-1 complex subunit mu-1 , GN=AP1M1	7	14	7	435	49.809	7.61
Q05682	Caldesmon , GN=CALD1	7	14	7	793	93.175	5.66
Q99996-6	Isoform 6 of A-kinase anchor protein 9 , GN=AKAP9	7	14	7	3929	455.142	4.98
P22307	Non-specific lipid-transfer protein , GN=SCP2	5	13	5	547	58.956	6.89
P98179	RNA-binding protein 3 , GN=RBM3	3	11	3	157	17.16	8.91
P02751-15	Isoform 15 of Fibronectin , GN=FN1	6	13	6	2477	272.15	5.5
P61764-2	Isoform 2 of Syntaxin-binding protein 1 , GN=STXBP1	7	12	7	603	68.692	6.77
O15144	Actin-related protein 2/3 complex subunit 2 , GN=ARPC2	5	12	5	300	34.311	7.36
Q8IV08	Phospholipase D3 , GN=PLD3	3	10	3	490	54.671	6.47
Q96GM5	SWI/SNF-related matrix-associated actin-dependent regulator of chromatin subfamily D member 1 , GN=SMARCD1	4	14	3	515	58.196	9.25
Q9BTT0	Acidic leucine-rich nuclear phosphoprotein 32 family member E , GN=ANP32E	3	11	3	268	30.674	3.85
Q16204	Coiled-coil domain-containing protein 6 , GN=CCDC6	6	13	6	474	53.258	7.34
Q5SW79	Centrosomal protein of 170 kDa , GN=CEP170	7	14	7	1584	175.187	7.11
Q05086	Ubiquitin-protein ligase E3A , GN=UBE3A	7	13	7	875	100.623	5.22
P18124	60S ribosomal protein L7 , GN=RPL7	3	16	3	248	29.207	10.65
P04066	Tissue alpha-L-fucosidase , GN=FUCA1	3	14	3	466	53.655	6.84
Q16698	2,4-dienoyl-CoA reductase, mitochondrial , GN=DECR1	5	14	5	335	36.045	9.28
Q14738	Serine/threonine-protein phosphatase 2A 56 kDa regulatory subunit delta isoform , GN=PPP2R5D	6	13	6	602	69.947	8.13
P43146	Netrin receptor DCC , GN=DCC	6	13	6	1447	158.357	6.77
Q7L2H7	Eukaryotic translation initiation factor 3 subunit M , GN=EIF3M	3	13	3	374	42.476	5.63
Q5EB52	Mesoderm-specific transcript homolog protein , GN=MEST PE=2	3	11	3	335	38.806	9.74
Q2TAY7	WD40 repeat-containing protein SMU1 , GN=SMU1	5	12	5	513	57.507	7.18
Q9UHV9	Prefoldin subunit 2 , GN=PFDN2	5	15	5	154	16.638	6.58
P46108	Adapter molecule crk , GN=CRK	2	12	2	304	33.81	5.55
Q9HAU0-6	Isoform 6 of Pleckstrin homology domain-containing family A member 5 , GN=PLEKHA5	9	12	9	1282	146.805	7.65
P53041	Serine/threonine-protein phosphatase 5 , GN=PPP5C	6	14	6	499	56.842	6.28
Q9HCD5	Nuclear receptor coactivator 5 , GN=NCOA5	3	12	3	579	65.496	9.6
P04899-4	Isoform sGi2 of Guanine nucleotide-binding protein G(i) subunit alpha-2 , GN=GNAI2	5	14	5	366	41.522	5.99
Q9Y4P1	Cysteine protease ATG4B , GN=ATG4B	4	13	4	393	44.266	5.07
P62714	Serine/threonine-protein phosphatase 2A catalytic subunit beta isoform , GN=PPP2CB	2	14	2	309	35.552	5.43
Q9NUU7	ATP-dependent RNA helicase DDX19A , GN=DDX19A	6	15	2	478	53.941	6.58
O00401	Neural Wiskott-Aldrich syndrome protein , GN=WASL	4	12	4	505	54.793	7.93
Q5TDH0-3	Isoform 3 of Protein DDI1 homolog 2 ,	5	13	5	419	46.542	5.1
P07686	Beta-hexosaminidase subunit beta , GN=HEXB	5	13	5	556	63.071	6.76
Q8IWZ3-6	Isoform 6 of Ankyrin repeat and KH domain-containing protein 1 , GN=ANKHD1	6	12	3	2617	277.004	5.86
P28838	Cytosol aminopeptidase , GN=LAP3	6	14	6	519	56.131	7.93
Q96A49	Synapse-associated protein 1 , GN=SYAP1	3	12	3	352	39.909	4.53
P05165	Propionyl-CoA carboxylase alpha chain, mitochondrial , GN=PCCA	7	13	7	728	80.008	7.52
P25685	DnaJ homolog subfamily B member 1 , GN=DNAJB1	3	15	3	340	38.02	8.63
Q96N67	Dedicator of cytokinesis protein 7 ,	7	14	7	2140	242.407	6.8

Q9BXJ9	N-alpha-acetyltransferase 15, NatA auxiliary subunit , GN=NAA15	5	15	5	866		101.208	7.42
P37837	Transaldolase , GN=TALDO1	5	15	5	337		37.516	6.81
P37108	Signal recognition particle 14 kDa protein , GN=SRP14	6	14	6	136		14.561	10.04
Q13596	Sorting nexin-1 , GN=SNX1	7	14	6	522		59.033	5.15
Q7Z2Z2	Elongation factor-like GTPase 1 , GN=EFL1	3	12	3	1120	125.35		5.91
Q13423	NAD(P) transhydrogenase, mitochondrial , GN=NNT	6	12	6	1086		113.823	8.09
P08579	U2 small nuclear ribonucleoprotein B'' , GN=SNRPB2	4	13	4	225	25.47		9.72
P04080	Cystatin-B , GN=CSTB	4	13	4	98		11.133	7.56
P29373	Cellular retinoic acid-binding protein 2 , GN=CRABP2	6	13	6	138		15.683	5.4
Q9H2P0	Activity-dependent neuroprotector homeobox protein , GN=ADNP	5	13	5	1102		123.485	7.34
P20908	Collagen alpha-1(V) chain , GN=COL5A1	6	13	6	1838		183.447	5.06
O75487	Glypican-4 , GN=GPC4	5	12	4	556		62.372	6.68
P49593	Protein phosphatase 1F , GN=PPM1F	4	12	4	454	49.8		5.1
P50148	Guanine nucleotide-binding protein G(q) subunit alpha , GN=GNAQ	4	13	3	359		42.115	5.68
P53999	Activated RNA polymerase II transcriptional coactivator p15 , GN=SUB1	2	12	2	127		14.386	9.6
Q9BS26	Endoplasmic reticulum resident protein 44 , GN=ERP44	3	11	3	406		46.941	5.26
Q9GZL7	Ribosome biogenesis protein WDR12 , GN=WDR12	3	12	3	423		47.678	5.9
O43252	Bifunctional 3'-phosphoadenosine 5'-phosphosulfate synthase 1 , GN=PAPSS1	3	14	3	624		70.788	6.86
Q15067	Peroxisomal acyl-coenzyme A oxidase 1 , GN=ACOX1	5	11	5	660		74.376	8.16
P14649	Myosin light chain 6B , GN=MYL6B	6	15	4	208	22.75		5.73
P36542	ATP synthase subunit gamma, mitochondrial , GN=ATP5C1	5	14	5	298		32.975	9.22
P02786	Transferrin receptor protein 1 , GN=TFRC	6	15	6	760		84.818	6.61
O43251-8	Isoform 8 of RNA binding protein fox-1 homolog 2 , GN=RBFOX2	3	12	2	451		47.314	6.62
Q9P2B4	CTTNBP2 N-terminal-like protein , GN=CTTNBP2NL	5	13	5	639		70.115	8.06
P00387-3	Isoform 3 of NADH-cytochrome b5 reductase 3 , GN=CYB5R3	5	12	5	334		38.202	6.9
P04843	Dolichyl-diphosphooligosaccharide--protein glycosyltransferase subunit 1 , GN=RPN1	5	12	5	607		68.527	6.38
O00264	Membrane-associated progesterone receptor component 1 , GN=PGRMC1	3	13	3	195		21.658	4.7
P63172	Dynein light chain Tctex-type 1 , GN=DYNLT1	2	11	2	113		12.444	5.08
O60271	C-Jun-amino-terminal kinase-interacting protein 4 , GN=SPAG9	9	13	9	1321		146.115	5.15
Q92900	Regulator of nonsense transcripts 1 ,	6	12	6	1129		124.267	6.61
Q9Y2B0	Protein canopy homolog 2 , GN=CNPY2	3	14	3	182		20.639	4.92
Q12792	Twinfilin-1 , GN=TWF1	3	11	3	350		40.258	6.96
O00470-2	Isoform 2 of Homeobox protein Meis1 , GN=MEIS1	2	10	1	463		50.543	6.55
Q05193	Dynamin-1 , GN=DNM1	8	13	7	864		97.347	7.17
P53634	Dipeptidyl peptidase 1 , GN=CTSC	2	10	2	463	51.82		6.99
Q9HAT2	Sialate O-acetylesterase , GN=SIAE	3	14	3	523		58.277	7.33
P52564	Dual specificity mitogen-activated protein kinase kinase 6 , GN=MAP2K6	5	13	5	334		37.468	7.39
P05166-2	Isoform 2 of Propionyl-CoA carboxylase beta chain, mitochondrial , GN=PCCB	6	13	6	559		60.483	7.24
Q15819	Ubiquitin-conjugating enzyme E2 variant 2 , GN=UBE2V2	4	14	4	145		16.352	8.09
P22570-3	Isoform 3 of NADPH:adrenodoxin oxidoreductase, mitochondrial , GN=FDXR	4	10	4	534		58.267	7.87
Q86YP4-3	Isoform 3 of Transcriptional repressor p66-alpha , GN=GATAD2A	6	13	5	634		68.092	9.94

Q14160-3	Isoform 3 of Protein scribble homolog , GN=SCRIB	8	12	8	1655	177.586	5.11
Q9NZL9	Methionine adenosyltransferase 2 subunit beta , GN=MAT2B	4	13	4	334	37.528	7.36
O43681	ATPase ASNA1 , GN=ASNA1	5	12	5	348	38.767	4.91
Q9H6T3	RNA polymerase II-associated protein 3 , GN=RPAP3	5	13	5	665	75.672	6.84
P45973	Chromobox protein homolog 5 , GN=CBX5	6	14	6	191	22.211	5.86
Q9NUJ1	Mycophenolic acid acyl-glucuronide esterase, mitochondrial , GN=ABHD10	5	11	5	306	33.911	8.57
O95747	Serine/threonine-protein kinase OSR1 , GN=OXSR1	5	11	5	527	57.986	6.43
Q9Y6E2	Basic leucine zipper and W2 domain-containing protein 2 , GN=BZW2	3	12	3	419	48.132	6.68
P18085	ADP-ribosylation factor 4 , GN=ARF4	4	14	2	180	20.498	7.14
Q8IZ21-2	Isoform 2 of Phosphatase and actin regulator 4 , GN=PHACTR4	4	12	4	712	79.081	6.73
Q6IN85	Serine/threonine-protein phosphatase 4 regulatory subunit 3A , GN=PPP4R3A	4	12	4	833	95.308	4.94
P24928	DNA-directed RNA polymerase II subunit RPB1 , GN=POLR2A	6	12	6	1970	217.039	7.37
P17655	Calpain-2 catalytic subunit , GN=CAPN2 SV=6	6	11	6	700	79.945	4.98
P04818	Thymidylate synthase , GN=TYMS	5	12	5	313	35.693	7.01
O15075	Serine/threonine-protein kinase DCLK1 , GN=DCLK1	5	13	5	740	82.173	8.66
O95299-2	Isoform 2 of NADH dehydrogenase [ubiquinone] 1 alpha subcomplex subunit 10, mitochondrial , GN=NDUFA10	4	11	4	429	48.532	7.21
O60518	Ran-binding protein 6 , GN=RANBP6	3	15	1	1105	124.633	5.01
Q6IA86-6	Isoform 6 of Elongator complex protein 2 , GN=ELP2	1	9	1	891	99.924	6.42
Q16775	Hydroxyacylglutathione hydrolase, mitochondrial , GN=HAGH	4	11	4	308	33.784	8.12
Q7Z4H8	KDEL motif-containing protein 2 , GN=KDELC2	4	12	4	507	58.535	8.24
P40123	Adenylyl cyclase-associated protein 2 , GN=CAP2	7	14	7	477	52.791	6.37
O95793	Double-stranded RNA-binding protein Staufen homolog 1 , GN=STAU1	4	13	4	577	63.143	9.44
O15173-2	Isoform 2 of Membrane-associated progesterone receptor component 2 , GN=PGRMC2	3	13	3	247	26.154	5.31
P49755	Transmembrane emp24 domain-containing protein 10 , GN=TMED10	3	11	3	219	24.96	7.44
Q9H0W9	Ester hydrolase C11orf54 , GN=C11orf54	3	12	3	315	35.095	6.7
P62314	Small nuclear ribonucleoprotein Sm D1 , GN=SNRPD1	1	13	1	119	13.273	11.56
Q15427	Splicing factor 3B subunit 4 , GN=SF3B4	3	12	3	424	44.357	8.56
P11047	Laminin subunit gamma-1 , GN=LAMC1	5	12	5	1609	177.489	5.12
Q8WZA9	Immunity-related GTPase family Q protein , GN=IRGQ	5	13	5	623	62.678	4.88
Q9Y2X3	Nucleolar protein 58 , GN=NOP58	4	10	4	529	59.541	8.92
P49257	Protein ERGIC-53 , GN=LMAN1	4	10	4	510	57.513	6.77
Q5EBL8-2	Isoform 2 of PDZ domain-containing protein 11 , GN=PDZD11	2	9	2	171	19.48	7.65
Q14019	Coactosin-like protein , GN=COTL1	2	11	2	142	15.935	5.67
O15042	U2 snRNP-associated SURP motif-containing protein , GN=U2SURP	6	13	6	1029	118.219	8.47
Q8NFH5	Nucleoporin NUP53 , GN=NUP35	4	12	4	326	34.751	9.09
Q8TEX9-2	Isoform 2 of Importin-4 , GN=IPO4	5	11	5	1083	118.826	4.94
P62820	Ras-related protein Rab-1A , GN=RAB1A	4	12	2	205	22.663	6.21
Q9C0C9	(E3-independent) E2 ubiquitin-conjugating enzyme , GN=UBE2O	5	12	5	1292	141.205	5.12
Q96M27-2	Isoform 2 of Protein PRRC1 , GN=PRRC1	3	11	3	464	48.495	5.39
Q13158	FAS-associated death domain protein , GN=FADD	2	9	2	208	23.265	5.69
Q8WW12	PEST proteolytic signal-containing nuclear protein , GN=PCNP	3	12	3	178	18.913	7.49

P15289	Arylsulfatase A , GN=ARSA	3	13	3	507		53.554	6.07
Q99829	Copine-1 , GN=CPNE1	4	11	4	537		59.022	5.83
Q07157	Tight junction protein ZO-1 , GN=TJP1	8	11	8	1748	195.34		6.7
Q8N3U4-2	Isoform 2 of Cohesin subunit SA-2 ,	3	10	3	1268		145.658	5.52
P35573	Glycogen debranching enzyme , GN=AGL	5	11	5	1532		174.652	6.76
P49354	Protein farnesyltransferase/geranylgeranyltransferase type-1 subunit alpha , GN=FNTA	2	10	2	379		44.381	5.08
P53621-2	Isoform 2 of Coatomer subunit alpha ,	9	13	9	1233		139.235	7.59
Q9Y2A7-2	Isoform 2 of Nck-associated protein 1 , GN=NCKAP1	5	13	5	1134		129.433	6.68
P47755	F-actin-capping protein subunit alpha-2 , GN=CAPZA2	3	12	3	286		32.929	5.85
P13489	Ribonuclease inhibitor , GN=RNH1	3	11	3	461		49.941	4.82
P09012	U1 small nuclear ribonucleoprotein A , GN=SNRPA	3	10	3	282		31.259	9.83
Q14166	Tubulin--tyrosine ligase-like protein 12 , GN=TTLL12	6	12	6	644		74.356	5.53
Q9H1B7	Interferon regulatory factor 2-binding protein-like , GN=IRF2BPL	4	11	4	796		82.607	8.24
Q96FJ2	Dynein light chain 2, cytoplasmic ,	3	12	2	89		10.343	7.37
O75934	Pre-mRNA-splicing factor SPF27 , GN=BCAS2	4	13	4	225		26.115	5.66
Q02246	Contactin-2 , GN=CNTN2	3	11	3	1040		113.322	7.96
Q9BRJ6	Uncharacterized protein C7orf50 ,	2	10	2	194	22.07		9.64
P60866-2	Isoform 2 of 40S ribosomal protein S20 , GN=RPS20	3	12	3	142		15.995	9.32
Q86YR5	G-protein-signaling modulator 1 , GN=GPSM1	4	11	4	675		74.464	6.54
P48556	26S proteasome non-ATPase regulatory subunit 8 , GN=PSMD8	3	12	3	350		39.587	9.7
Q6IBS0	Twinfilin-2 , GN=TWF2	3	10	3	349		39.523	6.84
O95487-3	Isoform 3 of Protein transport protein Sec24B , GN=SEC24B	5	11	4	1298		140.333	6.57
B5ME19	Eukaryotic translation initiation factor 3 subunit C-like protein , GN=EIF3CL PE=3	5	11	5	914		105.407	5.64
P14927	Cytochrome b-c1 complex subunit 7 ,	2	12	2	111		13.522	8.78
Q9Y2Z0	Protein SGT1 homolog , GN=SUGT1	7	12	7	365		40.998	5.16
Q9P035	Very-long-chain (3R)-3-hydroxyacyl-CoA dehydratase 3 , GN=HACD3	4	11	4	362		43.132	8.94
P99999	Cytochrome c , GN=CYCS	5	11	5	105		11.741	9.57
Q9Y2W1	Thyroid hormone receptor-associated protein 3 , GN=THRAP3	5	12	5	955		108.601	10.15
Q9UKK9	ADP-sugar pyrophosphatase , GN=NUDT5	5	11	5	219		24.312	4.94
O96019	Actin-like protein 6A , GN=ACTL6A	5	13	5	429	47.43		5.6
P15374	Ubiquitin carboxyl-terminal hydrolase isozyme L3 , GN=UCHL3	2	13	2	230		26.166	4.92
O00505	Importin subunit alpha-4 , GN=KPNA3	5	9	5	521		57.775	4.94
Q13617-2	Isoform 2 of Cullin-2 , GN=CUL2	4	13	4	764		89.434	7.11
Q8IXT5	RNA-binding protein 12B , GN=RBM12B	6	13	6	1001	118.03		6.81
P23193	Transcription elongation factor A protein 1 , GN=TCEA1	5	12	5	301		33.948	8.38
P51114	Fragile X mental retardation syndrome-related protein 1 , GN=FXR1	5	11	5	621		69.678	6.15
P60981	Destrin , GN=DSTN	2	12	2	165		18.493	7.85
P61960-2	Isoform 2 of Ubiquitin-fold modifier 1 ,	2	9	2	103		10.953	8.59
P23284	Peptidyl-prolyl cis-trans isomerase B ,	4	12	4	216		23.728	9.41
P09496-2	Isoform Non-brain of Clathrin light chain A , GN=CLTA	2	11	2	218		23.647	4.53
P16402	Histone H1.3 , GN=HIST1H1D	4	12	4	221		22.336	11.02
P52294	Importin subunit alpha-5 , GN=KPNA1	2	12	2	538		60.184	5.01
A8MWD9	Putative small nuclear ribonucleoprotein G-like protein 15 , GN=SNRPGP15 PE=5	2	14	2	76		8.538	8.84
Q06124	Tyrosine-protein phosphatase non-receptor type 11 , GN=PTPN11	5	10	5	597		68.393	7.3
P50851-2	Isoform 2 of Lipopolysaccharide-responsive and beige-like anchor protein , GN=LRBA	4	11	2	2851		317.501	5.57
Q9UDT6	CAP-Gly domain-containing linker protein 2 , GN=CLIP2	4	11	4	1046		115.767	6.73

Q9UL25	Ras-related protein Rab-21 , GN=RAB21	2	11	2	225		24.332	7.94	
Q96AC1-3	Isoform 3 of Fermitin family homolog 2 , GN=FERMT2	5	10	5	687		78.625	6.7	
P54727	UV excision repair protein RAD23 homolog B , GN=RAD23B	3	13	3	409		43.145	4.84	
Q9P1F3	Costars family protein ABRACL , GN=ABRACL	1	8	1	81		9.051	6.29	
P62847-4	Isoform 4 of 40S ribosomal protein S24 , GN=RPS24	2	11	2	289	32.41		10.15	
Q8NBF2	NHL repeat-containing protein 2 ,	7	11	7	726		79.393	5.55	
P50897	Palmitoyl-protein thioesterase 1 , GN=PPT1	3	12	3	306		34.171	6.52	
Q9UBC2-2	Isoform 2 of Epidermal growth factor receptor substrate 15-like 1 , GN=EPS15L1	6	10	6	910		99.545	5.08	
O75400	Pre-mRNA-processing factor 40 homolog A , GN=PRPF40A	4	11	4	957		108.737	7.56	
O15145	Actin-related protein 2/3 complex subunit 3 , GN=ARPC3	2	10	2	178		20.533	8.59	
Q9NRV9	Heme-binding protein 1 , GN=HEBP1	4	10	4	189		21.084	5.8	
Q96A65	Exocyst complex component 4 , GN=EXOC4	5	11	5	974		110.429	6.49	
Q01085-2	Isoform 2 of Nucleolysin TIAR , GN=TIAL1	5	11	4	392		43.421	7.99	
P40763	Signal transducer and activator of transcription 3 , GN=STAT3	4	10	4	770		88.011	6.3	
Q05397-5	Isoform 5 of Focal adhesion kinase 1 ,	5	13	5	1065		120.823	6.74	
P98172	Ephrin-B1 , GN=EFNB1	3	11	3	346		37.982	8.94	
Q6YN16	Hydroxysteroid dehydrogenase-like protein 2 , GN=HSDL2	5	11	5	418		45.366	7.99	
P37198	Nuclear pore glycoprotein p62 , GN=NUP62	5	11	5	522		53.222	5.31	
O43615	Mitochondrial import inner membrane translocase subunit TIM44 , GN=TIMM44	6	12	6	452		51.323	8.32	
O15540	Fatty acid-binding protein, brain , GN=FABP7	2	11	2	132		14.879	5.59	
Q13616	Cullin-1 , GN=CUL1	3	9	3	776		89.622		8
P55263	Adenosine kinase , GN=ADK	2	10	2	362	40.52		6.7	
Q9NPI1-2	Isoform 2 of Bromodomain-containing protein 7 , GN=BRD7	4	10	4	652	74.22		6.39	
P00505	Aspartate aminotransferase, mitochondrial , GN=GOT2	7	12	7	430		47.487	9.01	
P15924	Desmoplakin , GN=DSP	8	12	8	2871		331.569	6.81	
Q16799	Reticulon-1 , GN=RTN1	4	11	4	776		83.566	4.69	
Q9P0K7-2	Isoform 2 of Ankycorbin , GN=RAI14	6	10	6	983		110.354	6.11	
P11532-6	Isoform 6 of Dystrophin , GN=DMD	5	11	1	635		72.145	6.15	
Q9H7Z7	Prostaglandin E synthase 2 , GN=PTGES2	5	12	5	377		41.917	9.16	
Q8IWU6	Extracellular sulfatase Sulf-1 , GN=SULF1	1	8	1	871		100.962	9.09	
Q8IX01	SURP and G-patch domain-containing protein 2 , GN=SUGP2	4	10	4	1082		120.132	7.28	
P29992	Guanine nucleotide-binding protein subunit alpha-11 , GN=GNA11	3	11	2	359		42.097	5.69	
O15372	Eukaryotic translation initiation factor 3 subunit H , GN=EIF3H	4	11	4	352		39.905	6.54	
O95721	Synaptosomal-associated protein 29 , GN=SNAP29	4	12	4	258		28.953	5.81	
O76070	Gamma-synuclein , GN=SNCG	3	9	3	127		13.323	4.86	
P36578	60S ribosomal protein L4 , GN=RPL4	5	10	5	427		47.667	11.06	
Q9H8Y8	Golgi reassembly-stacking protein 2 , GN=GORASP2	4	10	4	452		47.116	4.82	
P58107	Epiplakin , GN=EPPK1	5	10	4	5090		555.279	5.6	
P35244	Replication protein A 14 kDa subunit ,	3	9	3	121	13.56		5.08	
P48444	Coatomer subunit delta , GN=ARCN1	7	11	7	511		57.174	6.21	
Q96F07	Cytoplasmic FMR1-interacting protein 2 , GN=CYFIP2	6	13	2	1278		148.302	7.31	
P48634	Protein PRRC2A , GN=PRRC2A	4	9	4	2157		228.724	9.45	
Q15813-2	Isoform 2 of Tubulin-specific chaperone E , GN=TBCE	4	11	4	578		64.811	7.27	
Q7Z4H3	HD domain-containing protein 2 , GN=HDDC2	2	10	2	204		23.376	5.49	
O75821	Eukaryotic translation initiation factor 3 subunit G , GN=EIF3G	3	11	3	320		35.589	6.13	
Q07954	Prolow-density lipoprotein receptor-related protein 1 , GN=LRP1	5	11	5	4544		504.276	5.39	
Q96L92	Sorting nexin-27 , GN=SNX27	3	12	3	541		61.226	6.49	

Q9UPY3	Endoribonuclease Dicer , GN=DICER1	7	10	7	1922	218.542	5.68
P60983	Glia maturation factor beta , GN=GMFB	4	11	4	142	16.702	5.29
P26639-2	Isoform 2 of Threonine--tRNA ligase, cytoplasmic , GN=TARS	5	12	5	756	86.806	6.77
Q9BRX8	Redox-regulatory protein FAM213A , GN=FAM213A	2	11	2	229	25.747	8.84
P04745	Alpha-amylase 1 , GN=AMY1A	5	9	5	511	57.731	6.93
P49023-3	Isoform Gamma of Paxillin , GN=PXN	4	10	4	605	66.17	6.37
Q9BQ04	RNA-binding protein 4B , GN=RBM4B	5	10	2	359	40.124	6.74
O75351	Vacuolar protein sorting-associated protein 4B , GN=VPS4B	2	11	2	444	49.271	7.23
Q9Y295	Developmentally-regulated GTP-binding protein 1 , GN=DRG1	4	12	4	367	40.517	8.9
Q9Y6D9	Mitotic spindle assembly checkpoint protein MAD1 , GN=MAD1L1	6	10	6	718	83.016	5.92
Q07960	Rho GTPase-activating protein 1 ,	6	11	6	439	50.404	6.29
Q13867	Bleomycin hydrolase , GN=BLMH	7	11	7	455	52.528	6.27
Q5SQI0	Alpha-tubulin N-acetyltransferase 1 ,	4	11	4	421	46.781	9.95
Q5SRE5	Nucleoporin NUP188 homolog , GN=NUP188	2	9	2	1749	195.917	6.73
Q9Y617	Phosphoserine aminotransferase , GN=PSAT1	6	12	6	370	40.397	7.66
P12110	Collagen alpha-2(VI) chain , GN=COL6A2	6	10	6	1019	108.512	6.21
P49585	Choline-phosphate cytidylyltransferase A , GN=PCYT1A	3	9	3	367	41.705	7.25
P35269	General transcription factor IIF subunit 1 , GN=GTF2F1	3	10	3	517	58.205	7.49
P56545-2	Isoform 2 of C-terminal-binding protein 2 , GN=CTBP2	1	9	1	985	106.121	8.02
Q13347	Eukaryotic translation initiation factor 3 subunit I , GN=EIF3I	4	11	4	325	36.479	5.64
P82650	28S ribosomal protein S22, mitochondrial , GN=MRPS22	5	10	5	360	41.254	7.9
P82979	SAP domain-containing ribonucleoprotein , GN=SARNP	4	10	4	210	23.656	6.42
Q7L775	EPM2A-interacting protein 1 , GN=EPM2AIP1	5	10	5	607	70.325	6.11
Q14108	Lysosome membrane protein 2 , GN=SCARB2	1	7	1	478	54.255	5.14
O14744	Protein arginine N-methyltransferase 5 , GN=PRMT5	4	9	4	637	72.638	6.29
P30876	DNA-directed RNA polymerase II subunit RPB2 , GN=POLR2B	6	10	6	1174	133.811	6.87
Q9UBK7-3	Isoform 3 of Rab-like protein 2A , GN=RABL2A	2	9	2	238	27.183	4.91
Q9HD45	Transmembrane 9 superfamily member 3 , GN=TM9SF3	1	7	1	589	67.843	7.21
Q6ZU35	Uncharacterized protein KIAA1211 , GN=KIAA1211	9	11	9	1233	136.678	5.6
Q7Z739	YTH domain-containing family protein 3 , GN=YTHDF3	5	10	4	585	63.822	9.04
Q96QR8	Transcriptional activator protein Pur-beta , GN=PURB	3	8	3	312	33.22	5.43
P29218-3	Isoform 3 of Inositol monophosphatase 1 , GN=IMPA1	5	11	5	336	36.671	7.91
P51570-2	Isoform 2 of Galactokinase , GN=GALK1	4	9	4	422	45.329	6.68
Q3KQU3	MAP7 domain-containing protein 1 , GN=MAP7D1	2	11	2	841	92.764	10.11
Q8WXX5	DnaJ homolog subfamily C member 9 , GN=DNAJC9	4	9	4	260	29.891	5.73
P04844	Dolichyl-diphosphooligosaccharide--protein glycosyltransferase subunit 2 , GN=RPN2	4	10	4	631	69.241	5.69
Q8NFP9	Neurobeachin , GN=NBEA	3	10	1	2946	327.614	6.18
Q6Y7W6	GRB10-interacting GYF protein 2 , GN=GIGYF2	7	10	7	1299	149.978	5.54
Q15276	Rab GTPase-binding effector protein 1 , GN=RABEP1	7	10	7	862	99.229	5.01
O95782	AP-2 complex subunit alpha-1 , GN=AP2A1	4	10	3	977	107.478	7.03
P78310	Coxsackievirus and adenovirus receptor , GN=CXADR	4	10	4	365	40.004	7.56
Q96ST3	Paired amphipathic helix protein Sin3a , GN=SIN3A	5	11	5	1273	145.085	7.25

Q8N6H7	ADP-ribosylation factor GTPase-activating protein 2 , GN=ARFGAP2	4	10	4	521	56.685	7.99
Q99707	Methionine synthase , GN=MTR	7	10	7	1265	140.437	5.58
O14745	Na(+)/H(+) exchange regulatory cofactor NHE-RF1 , GN=SLC9A3R1	4	10	4	358	38.845	5.77
Q9NSD9	Phenylalanine--tRNA ligase beta subunit , GN=FARSB	4	10	4	589	66.074	6.84
Q96NY7	Chloride intracellular channel protein 6 , GN=CLIC6 PE=2	3	9	3	704	72.968	4.37
Q9UMR2	ATP-dependent RNA helicase DDX19B , GN=DDX19B	5	11	1	479	53.893	6.3
P68400	Casein kinase II subunit alpha , GN=CSNK2A1	5	10	5	391	45.115	7.74
Q92626	Peroxidasin homolog , GN=PXDN	5	10	5	1479	165.17	7.17
P31949	Protein S100-A11 , GN=S100A11	1	8	1	105	11.733	7.12
Q15811	Intersectin-1 , GN=ITSN1	4	9	4	1721	195.3	7.77
Q53QV2	Protein LBH , GN=LBH	3	9	3	105	12.209	4.41
P61289-2	Isoform 2 of Proteasome activator complex subunit 3 , GN=PSME3	2	9	2	267	30.867	6.11
O75494	Serine/arginine-rich splicing factor 10 , GN=SRSF10	3	10	2	262	31.282	11.27
P14923	Junction plakoglobin , GN=JUP	3	11	3	745	81.693	6.14
P06132	Uroporphyrinogen decarboxylase , GN=UROD	3	9	3	367	40.761	6.14
P0C0S5	Histone H2A.Z , GN=H2AFZ	4	11	3	128	13.545	10.58
Q9Y6Y8	SEC23-interacting protein , GN=SEC23IP	5	9	5	1000	111.007	5.54
O95372	Acyl-protein thioesterase 2 , GN=LYPLA2	2	10	2	231	24.721	7.23
Q7L4I2	Arginine/serine-rich coiled-coil protein 2 , GN=RSRC2	2	9	2	434	50.529	11.33
O75832	26S proteasome non-ATPase regulatory subunit 10 , GN=PSMD10	2	9	2	226	24.412	6.1
P16220	Cyclic AMP-responsive element-binding protein 1 , GN=CREB1	3	8	1	341	36.666	5.57
P51148-2	Isoform 2 of Ras-related protein Rab-5C , GN=RAB5C	1	10	1	249	27.019	8.66
Q9NUL3	Double-stranded RNA-binding protein Staufen homolog 2 , GN=STAU2	4	10	4	570	62.601	9.61
P60953	Cell division control protein 42 homolog , GN=CDC42	1	11	1	191	21.245	6.55
A0AVT1	Ubiquitin-like modifier-activating enzyme 6 , GN=UBA6	4	10	4	1052	117.895	6.14
Q96PK6	RNA-binding protein 14 , GN=RBM14	5	11	5	669	69.449	9.67
Q8WXI9	Transcriptional repressor p66-beta , GN=GATAD2B	6	10	5	593	65.22	9.7
Q96HY6	DDRGK domain-containing protein 1 , GN=DDRGK1	2	10	2	314	35.589	5.12
Q9Y2Z4	Tyrosine--tRNA ligase, mitochondrial ,	4	10	4	477	53.166	8.98
Q9BYD6	39S ribosomal protein L1, mitochondrial , GN=MRPL1	2	9	2	325	36.885	8.78
Q68EM7	Rho GTPase-activating protein 17 , GN=ARHGAP17	3	9	3	881	95.377	7.62
Q14576	ELAV-like protein 3 , GN=ELAVL3 PE=2	3	10	2	367	39.522	9.28
Q99719	Septin-5 , GN=SEPT5	2	9	2	369	42.75	6.67
P63098	Calcineurin subunit B type 1 , GN=PPP3R1	3	8	3	170	19.288	4.81
Q9HAV4	Exportin-5 , GN=XPO5	7	10	7	1204	136.222	5.8
P12955	Xaa-Pro dipeptidase , GN=PEPD	2	8	2	493	54.513	6
P20042	Eukaryotic translation initiation factor 2 subunit 2 , GN=EIF2S2	4	9	4	333	38.364	5.8
Q9NX63	MICOS complex subunit MIC19 , GN=CHCHD3	3	10	3	227	26.136	8.28
Q05209	Tyrosine-protein phosphatase non-receptor type 12 , GN=PTPN12	4	8	4	780	88.051	5.62
P23229	Integrin alpha-6 , GN=ITGA6	5	10	5	1130	126.526	6.61
Q9P287-2	Isoform 2 of BRCA2 and CDKN1A-interacting protein , GN=BCCIP	3	9	3	322	36.192	4.69
Q9H9A6	Leucine-rich repeat-containing protein 40 , GN=LRRC40	4	9	4	602	68.207	6.43
A1L020	RNA-binding protein MEX3A , GN=MEX3A	5	8	5	520	54.139	7.27
P41240	Tyrosine-protein kinase CSK , GN=CSK	3	9	3	450	50.672	7.06
Q5JSH3	WD repeat-containing protein 44 ,	4	10	4	913	101.304	5.45

P62495	Eukaryotic peptide chain release factor subunit 1 , GN=ETF1	4	10	4	437		49	5.71
P35250	Replication factor C subunit 2 , GN=RFC2	3	10	3	354		39.132	6.44
P10599	Thioredoxin , GN=TXN	3	11	3	105	11.73		4.92
Q10570	Cleavage and polyadenylation specificity factor subunit 1 , GN=CPSF1	3	9	3	1443		160.782	6.4
P62701	40S ribosomal protein S4, X isoform ,	4	10	4	263		29.579	10.15
Q6ZTA4	Tripartite motif-containing protein 67 , GN=TRIM67 PE=2	3	8	3	783		83.769	7.33
Q15428	Splicing factor 3A subunit 2 , GN=SF3A2	2	9	2	464		49.224	9.64
Q8ND24	RING finger protein 214 , GN=RNF214	4	9	4	703		77.619	6.95
P31483	Nucleolysin TIA-1 isoform p40 , GN=TIA1	3	11	2	386		42.936	7.74
Q08945	FACT complex subunit SSRP1 , GN=SSRP1	5	10	5	709		81.024	6.87
P48449	Lanosterol synthase , GN=LSS	2	10	2	732		83.255	6.61
Q9H9Q2	COP9 signalosome complex subunit 7b , GN=COPS7B	4	9	4	264		29.603	6.15
Q12926	ELAV-like protein 2 , GN=ELAVL2	4	10	1	359		39.479	9.13
Q9UKM9	RNA-binding protein Raly , GN=RALY	5	11	5	306		32.444	9.17
Q9NR28	Diablo homolog, mitochondrial , GN=DIABLO	3	10	3	239		27.114	5.9
Q8WYA6	Beta-catenin-like protein 1 , GN=CTNNBL1	3	11	3	563		65.132	5.05
P11310-2	Isoform 2 of Medium-chain specific acyl-CoA dehydrogenase, mitochondrial , GN=ACADM	4	9	4	425	46.99		8.31
Q9H6Z4	Ran-binding protein 3 , GN=RANBP3	4	9	4	567		60.173	4.78
Q9P0M6	Core histone macro-H2A.2 , GN=H2AFY2	4	9	3	372		40.033	9.69
Q13017	Rho GTPase-activating protein 5 ,	6	9	6	1502		172.352	6.62
O43592	Exportin-T , GN=XPOT	4	10	4	962		109.893	5.39
Q9H3P2	Negative elongation factor A , GN=NELFA	4	10	4	528		57.241	9.03
P13637-3	Isoform 3 of Sodium/potassium-transporting ATPase subunit alpha-3 , GN=ATP1A3	3	9	1	1026		112.987	5.38
Q9NV70	Exocyst complex component 1 , GN=EXOC1	3	8	3	894		101.917	6.61
O75477	Erlin-1 , GN=ERLIN1	4	10	2	346		38.901	7.87
O43148-2	Isoform 2 of mRNA cap guanine-N7 methyltransferase , GN=RNMT	2	9	2	504		57.688	7.69
Q13620	Cullin-4B , GN=CUL4B	6	10	5	913		103.916	7.37
Q96A26	Protein FAM162A , GN=FAM162A	3	9	3	154		17.331	9.77
Q9UHB9	Signal recognition particle subunit SRP68 , GN=SRP68	3	9	3	627		70.686	8.56
Q9Y262	Eukaryotic translation initiation factor 3 subunit L , GN=EIF3L	3	9	3	564		66.684	6.34
Q96T76-8	Isoform 5 of MMS19 nucleotide excision repair protein homolog , GN=MMS19	4	10	4	1051		115.631	6.52
Q96G03	Phosphoglucomutase-2 , GN=PGM2	3	8	3	612	68.24		6.73
P61586	Transforming protein RhoA , GN=RHOA	2	9	1	193		21.754	6.1
Q6DN90	IQ motif and SEC7 domain-containing protein 1 , GN=IQSEC1	1	8	1	963		108.247	6.93
Q8NG27	E3 ubiquitin-protein ligase Praja-1 , GN=PJA1	2	8	2	643		70.959	5.17
P40425	Pre-B-cell leukemia transcription factor 2 , GN=PBX2	4	9	3	430		45.853	7.56
Q15555	Microtubule-associated protein RP/EB family member 2 , GN=MAPRE2	4	9	3	327		37.008	5.57
Q8TD19	Serine/threonine-protein kinase Nek9 , GN=NEK9	3	10	3	979	107.1		5.74
P40222	Alpha-taxilin , GN=TXLNA	5	8	5	546		61.853	6.52
Q9Y5S2	Serine/threonine-protein kinase MRCK beta , GN=CDC42BPB	5	8	5	1711		194.193	6.37
P50895	Basal cell adhesion molecule , GN=BCAM	3	9	3	628		67.363	5.81
Q9BTV5	Fibronectin type III and SPRY domain-containing protein 1 , GN=FSD1	3	8	3	496		55.784	6.99
Q7Z460-3	Isoform 3 of CLIP-associating protein 1 , GN=CLASP1	7	9	7	1494		164.465	8.47
Q9NZ08-2	Isoform 2 of Endoplasmic reticulum aminopeptidase 1 , GN=ERAP1	4	10	4	948		107.772	6.28
Q8IVD9	NudC domain-containing protein 3 , GN=NUDCD3	3	9	3	361		40.797	5.25
P51452	Dual specificity protein phosphatase 3 , GN=DUSP3	4	11	4	185		20.465	7.8

Q9C0B1	Alpha-ketoglutarate-dependent dioxygenase FTO , GN=FTO	3	9	3	505		58.245	5.22
O95319-3	Isoform 3 of CUGBP Elav-like family member 2 , GN=CELF2	3	8	3	521		55.719	9.04
Q02978	Mitochondrial 2-oxoglutarate/malate carrier protein , GN=SLC25A11	3	8	3	314	34.04		9.91
Q13576	Ras GTPase-activating-like protein IQGAP2 , GN=IQGAP2	5	8	5	1575		180.465	5.64
Q13868	Exosome complex component RRP4 , GN=EXOSC2	4	8	4	293		32.768	7.5
P45880-1	Isoform 1 of Voltage-dependent anion-selective channel protein 2 , GN=VDAC2	3	10	3	309		33.351	7.59
P05997	Collagen alpha-2(V) chain , GN=COL5A2	4	8	4	1499		144.821	6.46
P24539	ATP synthase F(0) complex subunit B1, mitochondrial , GN=ATP5F1	4	9	4	256	28.89		9.36
Q9H9Z2	Protein lin-28 homolog A , GN=LIN28A	2	10	2	209		22.728	8.07
O00291	Huntingtin-interacting protein 1 , GN=HIP1	4	7	4	1037		116.148	5.3
O75396	Vesicle-trafficking protein SEC22b ,	3	9	3	215		24.578	6.92
Q9BTE6-3	Isoform 3 of Alanyl-tRNA editing protein Aarsd1 , GN=AARSD1	4	8	4	586	65.66		6.51
P53602	Diphosphomevalonate decarboxylase , GN=MVD	3	7	3	400		43.377	7.23
Q96SB3	Neurabin-2 , GN=PPP1R9B	2	7	2	815		89.138	4.97
Q8IXM3	39S ribosomal protein L41, mitochondrial , GN=MRPL41	2	9	2	137		15.373	9.57
P12109	Collagen alpha-1(VI) chain , GN=COL6A1	7	10	7	1028		108.462	5.43
A0MZ66-3	Isoform 3 of Shootin-1 , GN=SHTN1	3	8	3	649		73.564	5.45
P17612	cAMP-dependent protein kinase catalytic subunit alpha , GN=PRKACA	3	9	2	351		40.564	8.79
P62841	40S ribosomal protein S15 , GN=RPS15	3	7	3	145		17.029	10.39
P28340	DNA polymerase delta catalytic subunit , GN=POLD1	5	9	5	1107		123.553	7.03
Q93052	Lipoma-preferred partner , GN=LPP	3	8	3	612		65.704	7.37
P52788	Spermine synthase , GN=SMS	4	9	4	366		41.242	5.02
Q9BTW9-4	Isoform 4 of Tubulin-specific chaperone D , GN=TBCD	5	8	5	1248	138.58		6.37
O75376	Nuclear receptor corepressor 1 , GN=NCOR1	6	8	6	2440		270.044	7.11
P62995	Transformer-2 protein homolog beta , GN=TRA2B	2	9	2	288		33.646	11.25
Q29RF7	Sister chromatid cohesion protein PDS5 homolog A , GN=PDS5A	3	8	3	1337		150.734	7.91
Q8IVL6	Prolyl 3-hydroxylase 3 , GN=P3H3	2	9	2	736		81.786	6.32
O43684	Mitotic checkpoint protein BUB3 , GN=BUB3	3	9	3	328		37.131	6.84
Q06210	Glutamine--fructose-6-phosphate aminotransferase [isomerizing] 1 , GN=GFPT1	4	9	4	699		78.756	7.11
Q15046-2	Isoform Mitochondrial of Lysine--tRNA ligase , GN=KARS	5	9	5	625		71.451	6.81
Q9NYF8	Bcl-2-associated transcription factor 1 , GN=BCLAF1	4	9	4	920		106.059	9.98
Q8IYK4	Procollagen galactosyltransferase 2 , GN=COLGALT2	3	9	3	626		72.878	6.2
Q8IYB3	Serine/arginine repetitive matrix protein 1 , GN=SRRM1	2	7	2	904		102.274	11.84
Q99439	Calponin-2 , GN=CNN2	4	8	4	309		33.675	7.33
O00499	Myc box-dependent-interacting protein 1 , GN=BIN1	4	9	4	593		64.659	5.06
Q13123	Protein Red , GN=IK	4	9	4	557		65.562	6.64
Q86U42	Polyadenylate-binding protein 2 ,	4	10	4	306		32.729	5.06
P00492	Hypoxanthine-guanine phosphoribosyltransferase , GN=HPRT1	4	10	4	218		24.564	6.68
Q6NVY1	3-hydroxyisobutyryl-CoA hydrolase, mitochondrial , GN=HIBCH	5	10	5	386		43.454	8.19
Q9H4M9	EH domain-containing protein 1 , GN=EHD1	6	8	6	534		60.589	6.83
P46939-2	Isoform 2 of Utrophin , GN=UTRN	4	7	4	3438		394.723	5.38
Q9UPN6-2	Isoform 2 of Protein SCAF8 , GN=SCAF8	4	9	3	1337		147.254	8.38
Q8IX12	Cell division cycle and apoptosis regulator protein 1 , GN=CCAR1	4	8	4	1150		132.739	5.76

Q14671-3	Isoform 3 of Pumilio homolog 1 , GN=PUM1	4	9	3	1188	126.607	6.84
P08240	Signal recognition particle receptor subunit alpha , GN=SRPRA	3	9	3	638	69.767	8.95
Q14676	Mediator of DNA damage checkpoint protein 1 , GN=MDC1	3	7	3	2089	226.529	5.47
P13716-2	Isoform 2 of Delta-aminolevulinic acid dehydratase , GN=ALAD	2	10	2	359	39.009	7.65
P42345	Serine/threonine-protein kinase mTOR , GN=MTOR	7	9	7	2549	288.707	7.17
Q96HC4	PDZ and LIM domain protein 5 , GN=PDLIM5	4	10	4	596	63.904	8.21
Q96I99	Succinate--CoA ligase [GDP-forming] subunit beta, mitochondrial , GN=SUCLG2	5	9	5	432	46.481	6.39
O95671	N-acetylserotonin O-methyltransferase-like protein , GN=ASMTL	3	9	3	621	68.813	6.07
Q92974	Rho guanine nucleotide exchange factor 2 , GN=ARHGEF2	6	9	6	986	111.473	7.27
P61201-2	Isoform 2 of COP9 signalosome complex subunit 2 , GN=COPS2	4	10	4	450	52.371	5.47
Q15907	Ras-related protein Rab-11B , GN=RAB11B	2	10	2	218	24.473	5.94
P20340	Ras-related protein Rab-6A , GN=RAB6A	3	10	2	208	23.578	5.54
P54105	Methylosome subunit pICln , GN=CLNS1A	3	8	3	237	26.199	4.11
Q16774-3	Isoform 3 of Guanylate kinase , GN=GUK1	2	8	2	241	26.58	9.1
P52943-2	Isoform 2 of Cysteine-rich protein 2 ,	2	6	2	282	30.202	8.22
Q7Z3K3	Pogo transposable element with ZNF domain , GN=POGZ	4	9	4	1410	155.245	7.4
Q15370-2	Isoform 2 of Elongin-B , GN=ELOB	3	9	3	161	17.9	6.1
P16278	Beta-galactosidase , GN=GLB1	4	8	4	677	76.027	6.57
Q5TFE4	5'-nucleotidase domain-containing protein 1 , GN=NT5DC1	3	9	3	455	51.812	6.35
Q9H0L4	Cleavage stimulation factor subunit 2 tau variant , GN=CSTF2T	4	8	1	616	64.396	7.25
P15121	Aldose reductase , GN=AKR1B1	3	8	3	316	35.83	6.98
Q9NQ86	E3 ubiquitin-protein ligase TRIM36 ,	2	8	2	728	82.96	6.11
P51513	RNA-binding protein Nova-1 , GN=NOVA1	4	8	3	510	52.024	8.88
P42285	Superkiller viralicidic activity 2-like 2 , GN=SKIV2L2	4	8	4	1042	117.729	6.52
Q9NZ45	CDGSH iron-sulfur domain-containing protein 1 , GN=CISD1	2	8	2	108	12.191	9.09
Q9NP81-2	Isoform 2 of Serine--tRNA ligase, mitochondrial , GN=SARS2	3	8	3	520	57.994	7.36
Q9UHD1	Cysteine and histidine-rich domain-containing protein 1 , GN=CHORDC1	3	9	3	332	37.466	7.87
Q8IXM2-2	Isoform 2 of Chromatin complexes subunit BAP18 , GN=BAP18	2	8	1	138	13.949	7.24
O43765	Small glutamine-rich tetratricopeptide repeat-containing protein alpha , GN=SGTA	5	9	5	313	34.042	4.87
Q8TEQ6	Gem-associated protein 5 , GN=GEMIN5	4	8	4	1508	168.483	6.62
Q9UNN5	FAS-associated factor 1 , GN=FAF1	4	8	4	650	73.908	4.88
Q16539	Mitogen-activated protein kinase 14 , GN=MAPK14	4	8	4	360	41.267	5.78
O00442-2	Isoform 2 of RNA 3'-terminal phosphate cyclase , GN=RTCA	2	7	2	379	40.683	8.34
Q9NXF1	Testis-expressed protein 10 , GN=TEX10	4	9	4	929	105.608	9.36
Q4G0N4	NAD kinase 2, mitochondrial , GN=NADK2	3	8	3	442	49.402	8.18
P49959-3	Isoform 3 of Double-strand break repair protein MRE11 , GN=MRE11	6	8	6	711	81.02	6.15
P20338	Ras-related protein Rab-4A , GN=RAB4A	3	9	2	218	24.374	6.07
Q86X55	Histone-arginine methyltransferase CARM1 , GN=CARM1	5	7	5	608	65.811	6.73
O75223	Gamma-glutamylcyclotransferase , GN=GGCT	3	8	3	188	20.994	5.14
Q8IXM2-3	Isoform 3 of Chromatin complexes subunit BAP18 , GN=BAP18	2	8	1	192	19.948	5.66
P52758	2-iminobutanoate/2-iminopropanoate deaminase , GN=RIDA	2	8	2	137	14.485	8.68
P23142-4	Isoform C of Fibulin-1 , GN=FBLN1	3	9	1	683	74.384	5.24
Q92597	Protein NDRG1 , GN=NDRG1	2	9	2	394	42.808	5.82
P27635	60S ribosomal protein L10 , GN=RPL10	2	9	2	214	24.588	10.08

Q8WVJ2	NudC domain-containing protein 2 , GN=NUDCD2	3	8	3	157	17.665	5.07
P34913	Bifunctional epoxide hydrolase 2 , GN=EPHX2	4	8	4	555	62.575	6.28
Q9UHJ6	Sedoheptulokinase , GN=SHPK	2	7	2	478	51.458	6.83
P15170-3	factor GTP-binding subunit ERF3A , GN=GSPT1	4	9	2	637	68.656	5.33
Q9UMZ2	Synergin gamma , GN=SYNRG	3	8	3	1314	140.566	5.03
Q712K3	Ubiquitin-conjugating enzyme E2 R2 , GN=UBE2R2	1	10	1	238	27.149	4.42
P49902	Cytosolic purine 5'-nucleotidase , GN=NT5C2	2	9	2	561	64.928	6.14
Q9Y6B6	GTP-binding protein SAR1b , GN=SAR1B	2	7	1	198	22.396	6.11
O14602	Eukaryotic translation initiation factor 1A, Y-chromosomal , GN=EIF1AY	2	9	2	144	16.432	5.24
P30520	Adenylosuccinate synthetase isozyme 2 , GN=ADSS	4	9	4	456	50.066	6.55
Q92665	28S ribosomal protein S31, mitochondrial , GN=MRPS31	4	8	4	395	45.29	9.29
Q96Q11	CCA tRNA nucleotidyltransferase 1, mitochondrial , GN=TRNT1	3	8	3	434	50.096	8.1
Q96DI7-2	ribonucleoprotein 40 kDa protein , GN=SNRNP40	2	6	2	409	44.488	7.9
P11413-2	Isoform Long of Glucose-6-phosphate 1-dehydrogenase , GN=G6PD	3	8	3	561	63.786	6.89
Q9H3K6	BolA-like protein 2 , GN=BOLA2	2	7	2	86	10.11	6.52
Q12769	Nuclear pore complex protein Nup160 , GN=NUP160	5	8	5	1436	162.017	5.5
O95881	Thioredoxin domain-containing protein 12 , GN=TXNDC12	2	8	2	172	19.194	5.4
Q9H6K5	Proline-rich protein 36 , GN=PRR36	4	8	4	1346	132.667	10.48
Q9NPI6	mRNA-decapping enzyme 1A , GN=DCP1A	4	6	4	582	63.27	6.25
P78406	mRNA export factor , GN=RAE1	3	9	3	368	40.942	7.83
P49418	Amphiphysin , GN=AMPH	5	8	5	695	76.21	4.64
Q9NX55	Huntingtin-interacting protein K , GN=HYPK	3	8	3	129	14.656	4.93
Q13459	Unconventional myosin-IXb , GN=MYO9B	2	6	2	2157	243.249	8.75
Q04323	UBX domain-containing protein 1 ,	4	7	4	297	33.305	5.25
P13807	Glycogen [starch] synthase, muscle ,	3	8	3	737	83.732	6.18
Q92947	Glutaryl-CoA dehydrogenase, mitochondrial , GN=GCDH	6	8	6	438	48.096	8.06
Q14669-3	Isoform 3 of E3 ubiquitin-protein ligase TRIP12 , GN=TRIP12	4	8	4	2040	225.379	8.69
P36507	Dual specificity mitogen-activated protein kinase kinase 2 , GN=MAP2K2	5	8	4	400	44.396	6.55
Q9NVZ3-2	Isoform 2 of Adaptin ear-binding coat-associated protein 2 , GN=NECAP2	1	7	1	273	29.446	8.38
Q8N7H5	RNA polymerase II-associated factor 1 homolog , GN=PAF1	4	9	4	531	59.939	4.63
P54803	Galactocerebrosidase , GN=GALC	3	7	3	685	77.015	6.64
O43719	HIV Tat-specific factor 1 , GN=HTATSF1	3	7	3	755	85.801	4.4
O75694	Nuclear pore complex protein Nup155 , GN=NUP155	4	7	4	1391	155.1	6.16
Q14789-2	Isoform 2 of Golgin subfamily B member 1 , GN=GOLGB1	5	7	5	3269	376.907	5
O75116	Rho-associated protein kinase 2 , GN=ROCK2	3	8	3	1388	160.799	6.02
P50402	Emerin , GN=EMD	3	8	3	254	28.976	5.5
P12429	Annexin A3 , GN=ANXA3	3	8	3	323	36.353	5.92
Q9P258	Protein RCC2 , GN=RCC2	6	9	6	522	56.049	8.78
P33992	DNA replication licensing factor MCM5 , GN=MCM5	2	8	2	734	82.233	8.37
Q08379	Golgin subfamily A member 2 , GN=GOLGA2	4	8	3	1002	113.017	5.02
Q6SZW1	Sterile alpha and TIR motif-containing protein 1 , GN=SARM1	4	9	4	724	79.338	6.55
Q12979	Active breakpoint cluster region-related protein , GN=ABR PE=2	3	7	3	859	97.536	6.55
P26378-3	Isoform 3 of ELAV-like protein 4 , GN=ELAVL4	4	9	1	383	42.411	9.39
Q96DE0	U8 snoRNA-decapping enzyme , GN=NUDT16	4	7	4	195	21.26	6.89
Q2TAL8	Glutamine-rich protein 1 , GN=QRICH1	3	6	3	776	86.382	5.87
P61601	Neurocalcin-delta , GN=NCALD	2	9	1	193	22.231	5.35

Q9NVI7-2	Isoform 2 of ATPase family AAA domain-containing protein 3A , GN=ATAD3A	4	8	4	586	66.177	9.25
Q6IAA8	Ragulator complex protein LAMTOR1 , GN=LAMTOR1	5	8	5	161	17.734	5.15
Q53H12	Acylglycerol kinase, mitochondrial , GN=AGK	2	7	2	422	47.107	8.09
P54577	Tyrosine--tRNA ligase, cytoplasmic , GN=YARS	4	8	4	528	59.106	7.05
Q9UK41-2	Isoform 2 of Vacuolar protein sorting-associated protein 28 homolog , GN=VPS28	3	8	3	233	26.445	7.72
Q9H857-2	Isoform 2 of 5'-nucleotidase domain-containing protein 2 , GN=NT5DC2	4	9	4	557	64.103	7.74
Q13098-7	Isoform 2 of COP9 signalosome complex subunit 1 , GN=GPS1	3	7	3	527	59.013	6.32
Q4G0J3-3	Isoform 3 of La-related protein 7 , GN=LARP7	3	9	3	589	67.612	9.54
P08263	Glutathione S-transferase A1 , GN=GSTA1	2	8	2	222	25.615	8.88
O60832	H/ACA ribonucleoprotein complex subunit 4 , GN=DKC1	3	8	3	514	57.638	9.42
Q9UIJ7	GTP:AMP phosphotransferase AK3, mitochondrial , GN=AK3	3	8	3	227	25.55	9.16
O95777	U6 snRNA-associated Sm-like protein LSm8 , GN=LSM8	2	6	2	96	10.396	4.48
P15880	40S ribosomal protein S2 , GN=RPS2	3	9	3	293	31.305	10.24
Q8NBJ5	Procollagen galactosyltransferase 1 , GN=COLGALT1	3	7	3	622	71.59	7.31
P52756	RNA-binding protein 5 , GN=RBM5	2	6	2	815	92.097	6.28
P16870	Carboxypeptidase E , GN=CPE	4	8	4	476	53.117	5.14
Q9BRQ0	Pygopus homolog 2 , GN=PYGO2	2	9	2	406	41.218	7.28
Q9NVT9	Armadillo repeat-containing protein 1 , GN=ARMC1	2	5	2	282	31.261	5.74
P62269	40S ribosomal protein S18 , GN=RPS18	2	8	2	152	17.708	10.99
O43172	U4/U6 small nuclear ribonucleoprotein Prp4 , GN=PRPF4	3	7	3	522	58.412	7.42
Q92688	Acidic leucine-rich nuclear phosphoprotein 32 family member B , GN=ANP32B	4	8	2	251	28.77	4.06
A6NE02	BTB/POZ domain-containing protein 17 , GN=BTBD17 PE=3	3	7	3	478	52.438	8.78
Q12955	Ankyrin-3 , GN=ANK3	3	7	3	4377	480.113	6.49
Q9NS69	Mitochondrial import receptor subunit TOM22 homolog , GN=TOMM22	2	9	2	142	15.512	4.34
O00139-4	Isoform 4 of Kinesin-like protein KIF2A , GN=KIF2A	4	7	4	744	84.037	6.37
Q07020	60S ribosomal protein L18 , GN=RPL18	2	9	2	188	21.621	11.72
O43290	U4/U6.U5 tri-snRNP-associated protein 1 , GN=SART1	3	7	3	800	90.2	6.13
Q9Y3E1	Hepatoma-derived growth factor-related protein 3 , GN=HDGFL3	3	7	2	203	22.606	7.99
O15160	DNA-directed RNA polymerases I and III subunit RPAC1 , GN=POLR1C	2	9	2	346	39.225	5.5
Q92575	UBX domain-containing protein 4 ,	2	7	2	508	56.743	6.38
Q9H3Q1	Cdc42 effector protein 4 , GN=CDC42EP4	4	7	4	356	37.957	5.19
Q8N129	Protein canopy homolog 4 , GN=CNPY4 PE=2	4	8	4	248	28.292	4.64
Q9GZZ9	Ubiquitin-like modifier-activating enzyme 5 , GN=UBA5	4	8	4	404	44.834	4.84
P31327-3	Isoform 3 of Carbamoyl-phosphate synthase [ammonia], mitochondrial , GN=CPS1	5	8	5	1506	165.545	6.79
Q7Z7K6	Centromere protein V , GN=CENPV	2	6	1	275	29.927	9.73
P61221	ATP-binding cassette sub-family E member 1 , GN=ABCE1	4	7	4	599	67.271	8.34
Q32MZ4	Leucine-rich repeat flightless-interacting protein 1 , GN=LRRFIP1	4	8	3	808	89.199	4.65
Q9UI30	Multifunctional methyltransferase subunit TRM112-like protein , GN=TRMT112	4	8	4	125	14.19	5.26
P40424	Pre-B-cell leukemia transcription factor 1 , GN=PBX1	3	7	2	430	46.597	7.03
Q16537	Serine/threonine-protein phosphatase 2A 56 kDa regulatory subunit epsilon isoform , GN=PPP2R5E	4	8	4	467	54.664	6.95
Q15477	Helicase SKI2W , GN=SKIV2L	6	8	6	1246	137.668	6.06

P62942	Peptidyl-prolyl cis-trans isomerase FKBP1A , GN=FKBP1A	3	7	3	108		11.943	8.16
P13611	Versican core protein , GN=VCAN	4	8	4	3396	372.59		4.51
Q5UIP0	Telomere-associated protein RIF1 , GN=RIF1	6	8	6	2472		274.294	5.52
O00422	Histone deacetylase complex subunit SAP18 , GN=SAP18	2	6	2	153	17.55		9.35
O15357	Phosphatidylinositol 3,4,5-trisphosphate 5-phosphatase 2 , GN=INPPL1	4	8	4	1258		138.513	6.54
P62899-2	Isoform 2 of 60S ribosomal protein L31 , GN=RPL31	2	8	2	128		14.623	10.8
Q9H4A6	Golgi phosphoprotein 3 , GN=GOLPH3	2	7	2	298	33.79		6.44
P09543	2',3'-cyclic-nucleotide 3'-phosphodiesterase , GN=CNP	3	7	3	421		47.549	9.07
P37268	Squalene synthase , GN=FDFT1	3	7	3	417		48.084	6.54
P50552	Vasodilator-stimulated phosphoprotein , GN=VASP	3	7	3	380		39.805	8.94
Q16795	NADH dehydrogenase [ubiquinone] 1 alpha subcomplex subunit 9, mitochondrial , GN=NDUFA9	2	8	2	377		42.483	9.8
O43598	2'-deoxynucleoside 5'-phosphate N-hydrolase 1 , GN=DNPH1	2	7	2	174		19.097	5.05
Q8NE71	ATP-binding cassette sub-family F member 1 , GN=ABCF1	5	8	5	845		95.866	6.8
Q9UPY8	Microtubule-associated protein RP/EB family member 3 , GN=MAPRE3	2	8	1	281		31.962	5.54
P29144	Tripeptidyl-peptidase 2 , GN=TPP2	2	7	2	1249		138.263	6.32
Q9Y3A5	Ribosome maturation protein SBDS ,	2	6	2	250		28.745	8.75
Q5BKZ1	DBIRD complex subunit ZNF326 , GN=ZNF326	3	8	3	582		65.613	5.15
P39019	40S ribosomal protein S19 , GN=RPS19	3	8	3	145		16.051	10.32
P55084	Trifunctional enzyme subunit beta, mitochondrial , GN=HADHB	6	8	6	474		51.262	9.41
Q9H2G2	STE20-like serine/threonine-protein kinase , GN=SLK	4	8	4	1235		142.607	5.15
P12931-2	Isoform 2 of Proto-oncogene tyrosine-protein kinase Src , GN=SRC	3	8	2	542	60.55		7.84
Q7Z417	Nuclear fragile X mental retardation-interacting protein 2 , GN=NUFIP2	5	7	5	695		76.075	8.7
Q9NP97	Dynein light chain roadblock-type 1 , GN=DYNLRB1	4	8	4	96		10.915	7.25
O14641	Segment polarity protein dishevelled homolog DVL-2 , GN=DVL2	4	8	4	736		78.899	6.02
Q01081	Splicing factor U2AF 35 kDa subunit ,	2	7	2	240		27.854	8.81
Q9H008	Phospholysine phosphohistidine inorganic pyrophosphate phosphatase , GN=LHPP	2	7	2	270		29.147	6.15
Q8NHP8	Putative phospholipase B-like 2 , GN=PLBD2	1	8	1	589	65.43		6.8
Q9UBV8	Peflin , GN=PEF1	4	8	4	284		30.361	6.54
Q9Y570-4	Isoform 4 of Protein phosphatase methylesterase 1 , GN=PPME1	2	8	2	400		43.842	5.85
Q9NRL3-3	Isoform 3 of Striatin-4 , GN=STRN4	3	8	3	760		81.215	5.4
Q7LG56-6	Isoform 6 of Ribonucleoside-diphosphate reductase subunit M2 B , GN=RRM2B	3	7	2	423		48.756	7.99
Q9NXH9	tRNA (guanine(26)-N(2))-dimethyltransferase , GN=TRMT1	5	8	5	659		72.188	7.64
P63027	Vesicle-associated membrane protein 2 , GN=VAMP2	1	5	1	116		12.655	8.13
Q8N6N7	Acyl-CoA-binding domain-containing protein 7 , GN=ACBD7	2	7	2	88		9.784	6.71
P20810-6	Isoform 6 of Calpastatin , GN=CAST	3	6	3	791		84.891	5.36
O00399	Dynactin subunit 6 , GN=DCTN6	2	7	2	190		20.734	6.32
O60264	SWI/SNF-related matrix-associated actin-dependent regulator of chromatin subfamily A member 5 , GN=SMARCA5	4	8	3	1052		121.828	8.09
O75822	Eukaryotic translation initiation factor 3 subunit J , GN=EIF3J	2	8	2	258		29.045	4.83
P11172	Uridine 5'-monophosphate synthase ,	3	8	3	480		52.189	7.24
P42224	Signal transducer and activator of transcription 1-alpha/beta , GN=STAT1	3	7	3	750	87.28		6.05

Q96AX1	Vacuolar protein sorting-associated protein 33A , GN=VPS33A	3	7	3	596	67.568	6.96
Q5VT52	Regulation of nuclear pre-mRNA domain-containing protein 2 , GN=RPRD2	5	7	5	1461	155.924	7.42
O14639	Actin-binding LIM protein 1 , GN=ABLIM1	3	8	3	778	87.631	8.59
Q13724	Mannosyl-oligosaccharide glucosidase , GN=MOGS	4	7	4	837	91.861	8.9
Q9UPT8	Zinc finger CCCH domain-containing protein 4 , GN=ZC3H4	3	8	3	1303	140.169	6.27
Q9Y547	Intraflagellar transport protein 25 homolog , GN=HSPB11	3	7	3	144	16.287	5.03
Q7Z3T8	Zinc finger FYVE domain-containing protein 16 , GN=ZFYVE16	6	8	6	1539	168.797	4.82
O43795	Unconventional myosin-Ib , GN=MYO1B	2	6	2	1136	131.902	9.38
P23142	Fibulin-1 , GN=FBLN1	3	8	1	703	77.162	5.22
Q9Y6K1	DNA (cytosine-5)-methyltransferase 3A , GN=DNMT3A	3	7	3	912	101.793	6.57
Q8TDZ2-4	Isoform 4 of [F-actin]-monooxygenase MICAL1 , GN=MICAL1	3	6	3	1086	119.746	6.38
Q92917	G-patch domain and KOW motifs-containing protein , GN=GPKOW	3	6	3	476	52.197	6.15
P09471	Guanine nucleotide-binding protein G(o) subunit alpha , GN=GNAO1	3	7	3	354	40.025	5.53
O60869	Endothelial differentiation-related factor 1 , GN=EDF1	1	7	1	148	16.359	9.95
Q99653	Calcineurin B homologous protein 1 ,	2	7	2	195	22.442	5.1
Q9UQB8	Brain-specific angiogenesis inhibitor 1-associated protein 2 , GN=BAIAP2	3	7	3	552	60.83	8.9
P02792	Ferritin light chain , GN=FTL	3	7	3	175	20.007	5.78
P28370	Probable global transcription activator SNF2L1 , GN=SMARCA1	5	8	4	1054	122.527	8.09
P41091	Eukaryotic translation initiation factor 2 subunit 3 , GN=EIF2S3	2	7	2	472	51.077	8.4
O14964	Hepatocyte growth factor-regulated tyrosine kinase substrate , GN=HGS	3	6	3	777	86.138	6.16
Q53GS9	U4/U6.U5 tri-snRNP-associated protein 2 , GN=USP39	3	7	3	565	65.34	8.91
P20020	Plasma membrane calcium-transporting ATPase 1 , GN=ATP2B1	4	7	2	1258	138.668	6.04
O00178	GTP-binding protein 1 , GN=GTPBP1	3	8	3	669	72.408	8.34
Q15059	Bromodomain-containing protein 3 ,	3	7	3	726	79.492	9.36
Q03001	Dystonin , GN=DST	3	7	2	7570	860.127	5.25
P22087	rRNA 2'-O-methyltransferase fibrillarin ,	2	7	2	321	33.763	10.18
Q9NYB9	Abl interactor 2 , GN=ABI2	2	6	2	513	55.629	6.16
P61956	Small ubiquitin-related modifier 2 ,	2	8	2	95	10.864	5.5
Q9NY27	Serine/threonine-protein phosphatase 4 regulatory subunit 2 , GN=PPP4R2	4	8	4	417	46.869	4.54
O96013-2	Isoform 2 of Serine/threonine-protein kinase PAK 4 , GN=PAK4	4	7	1	426	47.894	9.47
Q8WUM0	Nuclear pore complex protein Nup133 , GN=NUP133	3	7	3	1156	128.898	5.1
P78356	Phosphatidylinositol 5-phosphate 4-kinase type-2 beta , GN=PIP4K2B	3	6	3	416	47.348	7.33
Q96C19	EF-hand domain-containing protein D2 , GN=EFHD2	2	8	1	240	26.68	5.2
P62906	60S ribosomal protein L10a , GN=RPL10A	2	7	2	217	24.816	9.94
Q8NFD5-3	Isoform 3 of AT-rich interactive domain-containing protein 1B , GN=ARID1B	4	7	4	2289	241.31	6.67
Q8IYD1	Eukaryotic peptide chain release factor GTP-binding subunit ERF3B , GN=GSPT2	3	8	1	628	68.84	5.43
P16083	Ribosyldihydronicotinamide dehydrogenase [quinone] , GN=NQO2	3	7	3	231	25.902	6.29
Q9P2I0	Cleavage and polyadenylation specificity factor subunit 2 , GN=CPSF2	3	7	3	782	88.431	5.11
O95347	Structural maintenance of chromosomes protein 2 , GN=SMC2	3	7	3	1197	135.572	8.43
Q9BSY9	Desumoylating isopeptidase 2 , GN=DESI2	1	5	1	194	21.43	4.92

Q9Y2L1	Exosome complex exonuclease RRP44 ,	3	8	3	958	108.934	7.14	
Q9NR31	GTP-binding protein SAR1a , GN=SAR1A	3	7	2	198	22.353	6.68	
Q6ZSR9	Uncharacterized protein FLJ45252 , PE=2	6	7	6	355	37.953	5.26	
Q9P270	SLAIN motif-containing protein 2 , GN=SLAIN2	3	7	3	581	62.505	9.45	
O15230	Laminin subunit alpha-5 , GN=LAMA5 SV=8	4	7	4	3695	399.479	7.02	
P18615-3	Isoform 2 of Negative elongation factor E , GN=NELFE	3	6	3	387	43.898	9.39	
Q13442	28 kDa heat- and acid-stable phosphoprotein , GN=PDAP1	3	7	3	181	20.618	8.87	
Q9NSE4	Isoleucine--tRNA ligase, mitochondrial , GN=IARS2	4	7	4	1012	113.719	7.2	
A8K0Z3	WASH complex subunit 1 , GN=WASHC1	3	8	3	465	50.297	5.68	
O43670-4	motif-containing protein ZNF207 , GN=ZNF207	3	8	3	494	52.659	9.1	
Q9BSJ8-2	Isoform 2 of Extended synaptotagmin-1 , GN=ESYT1	4	8	4	1114	123.925	5.78	
Q9UKG9-3	Isoform 3 of Peroxisomal carnitine O-octanoyltransferase , GN=CROT	4	7	4	640	73.336	7.18	
Q93034	Cullin-5 , GN=CUL5	4	6	4	780	90.897	7.94	
Q6P1J9	Parafibromin , GN=CDC73	3	7	3	531	60.539	9.61	
Q9NXV6	CDKN2A-interacting protein , GN=CDKN2AIP	6	8	6	580	61.088	9.01	
Q96HQ2	CDKN2AIP N-terminal-like protein , GN=CDKN2AIPNL	2	6	2	116	13.187		5
P15927-3	Isoform 3 of Replication protein A 32 kDa subunit , GN=RPA2	2	7	2	358	38.786	8.94	
Q9NRY4	Rho GTPase-activating protein 35 , GN=ARHGAP35	3	6	3	1499	170.407	6.64	
O96013	Serine/threonine-protein kinase PAK 4 , GN=PAK4	4	7	1	591	64.032	9.73	
P08195-4	Isoform 4 of 4F2 cell-surface antigen heavy chain , GN=SLC3A2	4	7	4	661	71.079	4.97	
Q9BRP8	Partner of Y14 and mago , GN=PYM1	3	7	3	204	22.642	9.45	
Q9NWB7	Intraflagellar transport protein 57 homolog , GN=IFT57	3	6	3	429	49.078	4.98	
Q9GZT3	SRA stem-loop-interacting RNA-binding protein, mitochondrial , GN=SLIRP	3	8	3	109	12.341	10.24	
Q9UKF6	Cleavage and polyadenylation specificity factor subunit 3 , GN=CPSF3	4	6	4	684	77.436	5.6	
P15848	Arylsulfatase B , GN=ARSB	2	7	2	533	59.649	8.21	
Q5JPE7	Nodal modulator 2 , GN=NOMO2	5	7	5	1267	139.351	5.76	
O94760	N(G),N(G)-dimethylarginine dimethylaminohydrolase 1 , GN=DDAH1	6	7	6	285	31.102	5.81	
O15397	Importin-8 , GN=IPO8	4	7	4	1037	119.861	5.16	
P55058	Phospholipid transfer protein , GN=PLTP	5	8	5	493	54.705	7.01	
P11171	Protein 4.1 , GN=EPB41	4	8	4	864	96.957	5.58	
A6NCE7	Microtubule-associated proteins 1A/1B light chain 3 beta 2 , GN=MAP1LC3B2 PE=2	1	8	1	125	14.619	8.68	
O60641	Clathrin coat assembly protein AP180 , GN=SNAP91	3	7	2	907	92.444	4.86	
P54619-3	Isoform 3 of 5'-AMP-activated protein kinase subunit gamma-1 , GN=PRKAG1	1	6	1	340	38.508	7.09	
Q96G28-2	Isoform 2 of Cilia- and flagella-associated protein 36 , GN=CFAP36	2	6	2	367	41.902	5.01	
O94888	UBX domain-containing protein 7 ,	4	7	4	489	54.828	5.16	
Q5VIR6-4	Isoform 4 of Vacuolar protein sorting-associated protein 53 homolog , GN=VPS53	1	5	1	832	94.345	6.7	
P52306-5	Isoform 5 of Rap1 GTPase-GDP dissociation stimulator 1 , GN=RAP1GDS1	4	8	4	608	66.346	5.31	
P07203	Glutathione peroxidase 1 , GN=GPX1	2	7	2	203	22.075	6.55	
Q9BVG4	Protein PBDC1 , GN=PBDC1	2	8	2	233	26.041	4.79	
P51003	Poly(A) polymerase alpha , GN=PAPOLA	4	7	4	745	82.791	7.37	
P06493	Cyclin-dependent kinase 1 , GN=CDK1	6	7	6	297	34.074	8.4	
Q53FA7	Quinone oxidoreductase PIG3 , GN=TP53I3	4	8	4	332	35.514	7.17	
O94763	Unconventional prefoldin RPB5 interactor 1 , GN=URI1	2	7	2	535	59.796	5.05	
Q9NVM6	DnaJ homolog subfamily C member 17 , GN=DNAJC17	1	6	1	304	34.666	8.53	

P27816-3	Isoform 3 of Microtubule-associated protein 4 , GN=MAP4	3	7	3	539	58.52	6.04
O14828	Secretory carrier-associated membrane protein 3 , GN=SCAMP3	3	6	3	347	38.262	7.64
P54802	Alpha-N-acetylglucosaminidase , GN=NAGLU	5	7	5	743	82.214	6.65
Q13085-4	Isoform 4 of Acetyl-CoA carboxylase 1 , GN=ACACA	5	8	5	2383	269.827	6.52
Q9UBU9	Nuclear RNA export factor 1 , GN=NXF1	2	7	2	619	70.139	8.51
Q9BY44	Eukaryotic translation initiation factor 2A , GN=EIF2A	2	7	2	585	64.949	8.87
Q96EP5	DAZ-associated protein 1 , GN=DAZAP1	4	6	4	407	43.356	8.56
Q86VS8	Protein Hook homolog 3 , GN=HOOK3	4	7	4	718	83.074	5.17
P09110	3-ketoacyl-CoA thiolase, peroxisomal , GN=ACAA1	3	7	3	424	44.264	8.44
P43487	Ran-specific GTPase-activating protein , GN=RANBP1	3	8	3	201	23.296	5.29
Q6PKG0	La-related protein 1 , GN=LARP1	5	7	5	1096	123.434	8.82
O43633	Charged multivesicular body protein 2a , GN=CHMP2A	2	6	2	222	25.088	5.97
Q9UEE9	Craniofacial development protein 1 ,	3	8	3	299	33.573	4.81
P51688	N-sulphoglucosamine sulphohydrolase , GN=SGSH	2	7	2	502	56.659	6.95
Q9Y3C6	Peptidyl-prolyl cis-trans isomerase-like 1 , GN=PPIL1	2	6	2	166	18.225	7.99
P07741	Adenine phosphoribosyltransferase ,	3	8	3	180	19.595	6.02
P48431	Transcription factor SOX-2 , GN=SOX2	2	6	2	317	34.287	9.74
Q9H4G0-2	Isoform 2 of Band 4.1-like protein 1 , GN=EPB41L1	3	7	2	779	87.591	5.92
P26885	Peptidyl-prolyl cis-trans isomerase FKBP2 , GN=FKBP2	1	7	1	142	15.639	9.13
Q9NRG7-2	Isoform 2 of Epimerase family protein SDR39U1 , GN=SDR39U1	2	6	2	293	31.057	9.04
Q14151	Scaffold attachment factor B2 , GN=SAFB2	3	7	2	953	107.408	6.16
O00273	DNA fragmentation factor subunit alpha , GN=DFFA	3	7	3	331	36.5	4.79
Q7Z5L9	Interferon regulatory factor 2-binding protein 2 , GN=IRF2BP2	2	6	2	587	60.987	8.69
Q9Y657	Spindlin-1 , GN=SPIN1	3	8	3	262	29.582	6.96
O15294	UDP-N-acetylglucosamine--peptide N-acetylglucosaminyltransferase 110 kDa subunit , GN=OGT	4	6	4	1046	116.85	6.7
Q15018	BRISC complex subunit Abraxas 2 , GN=ABRAXAS2	3	7	3	415	46.872	6.21
O43678	NADH dehydrogenase [ubiquinone] 1 alpha subcomplex subunit 2 , GN=NDUFA2	1	6	1	99	10.915	9.57
Q7Z4V5	Hepatoma-derived growth factor-related protein 2 , GN=HDGFL2	2	6	1	671	74.272	7.49
P20933	N(4)-(beta-N-acetylglucosaminyl)-L-asparaginase , GN=AGA	1	8	1	346	37.184	6.28
Q9BUH6	Protein PAXX , GN=PAXX	3	7	3	204	21.626	5.48
P23378	Glycine dehydrogenase (decarboxylating), mitochondrial , GN=GLDC	3	6	3	1020	112.657	7.11
O00213	Amyloid-beta A4 precursor protein-binding family B member 1 , GN=APBB1	2	6	2	710	77.196	5.06
Q15424-3	Isoform 3 of Scaffold attachment factor B1 , GN=SAFB	3	6	2	917	102.793	5.49
Q96GD0	Pyridoxal phosphate phosphatase , GN=PDXP	2	7	2	296	31.678	6.55
Q7Z7K6-3	Isoform 3 of Centromere protein V ,	2	5	1	272	29.712	9.73
O14763	Tumor necrosis factor receptor superfamily member 10B , GN=TNFRSF10B	1	6	1	440	47.848	5.55
O94966-6	Isoform 6 of Ubiquitin carboxyl-terminal hydrolase 19 , GN=USP19	4	7	4	1384	151.83	6.42
Q9H3U1	Protein unc-45 homolog A , GN=UNC45A	5	7	5	944	103.011	6.07
Q96MW5	Conserved oligomeric Golgi complex subunit 8 , GN=COG8	2	6	2	612	68.38	5.2
Q8TCD5	5'(3')-deoxyribonucleotidase, cytosolic type , GN=NT5C	2	6	2	201	23.368	6.64

Q9H2J4	Phosducin-like protein 3 , GN=PDCL3	3	6	3	239		27.597	4.84
Q9Y625	Glypican-6 , GN=GPC6	2	6	1	555		62.695	5.41
P00813	Adenosine deaminase , GN=ADA	4	6	4	363		40.739	5.95
Q7Z6B7	SLIT-ROBO Rho GTPase-activating protein 1 , GN=SRGAP1	1	7	1	1085		124.186	6.83
Q14155-5	Isoform 5 of Rho guanine nucleotide exchange factor 7 , GN=ARHGEF7	3	5	3	731		82.519	6.84
Q14978-3	Isoform 3 of Nucleolar and coiled-body phosphoprotein 1 , GN=NOLC1	4	7	4	700		73.702	9.47
Q12972	Nuclear inhibitor of protein phosphatase 1 , GN=PPP1R8	3	6	3	351		38.455	7.37
P46100	Transcriptional regulator ATRX , GN=ATRX	6	7	6	2492		282.411	6.58
Q08J23	tRNA (cytosine(34)-C(5))-methyltransferase , GN=NSUN2	3	6	3	767		86.416	6.77
P18858	DNA ligase 1 , GN=LIG1	3	6	3	919		101.673	5.62
Q9UID3	Vacuolar protein sorting-associated protein 51 homolog , GN=VPS51	3	6	3	782		85.988	6.47
O60749	Sorting nexin-2 , GN=SNX2	5	7	4	519		58.435	5.12
O75970	Multiple PDZ domain protein , GN=MPDZ	4	6	4	2070		221.481	5.06
O00754	Lysosomal alpha-mannosidase , GN=MAN2B1	2	7	2	1011		113.672	7.28
O95425	Supervillin , GN=SVIL	3	6	3	2214		247.593	6.98
O14929	Histone acetyltransferase type B catalytic subunit , GN=HAT1	4	6	4	419		49.481	5.69
Q99622	Protein C10 , GN=C12orf57	3	6	3	126	13.17		5.14
Q9Y3P9	Rab GTPase-activating protein 1 ,	3	6	3	1069	121.66		5.25
Q14677-3	Isoform 3 of Clathrin interactor 1 , GN=CLINT1	4	7	4	643	70.25		6.58
P20936	Ras GTPase-activating protein 1 , GN=RASA1	5	6	5	1047		116.329	6.54
P43304	Glycerol-3-phosphate dehydrogenase, mitochondrial , GN=GPD2	5	6	5	727		80.802	7.69
Q9BY32	Inosine triphosphate pyrophosphatase , GN=ITPA	3	6	3	194		21.432	5.66
Q9Y314	Nitric oxide synthase-interacting protein , GN=NOSIP	4	6	4	301		33.151	8.82
Q9UBN7	Histone deacetylase 6 , GN=HDAC6	2	6	2	1215		131.336	5.3
Q9C0B0	RING finger protein unkempt homolog ,	3	6	3	810		88.029	6.86
Q9BRG1	Vacuolar protein-sorting-associated protein 25 , GN=VPS25	2	7	2	176		20.735	6.34
Q13332	Receptor-type tyrosine-protein phosphatase S , GN=PTPRS	5	6	5	1948		216.905	6.46
O95479	GDH/6PGL endoplasmic bifunctional protein , GN=H6PD	4	6	4	791		88.836	7.3
Q9Y333	U6 snRNA-associated Sm-like protein LSm2 , GN=LSM2	1	5	1	95		10.828	6.52
Q9UNP9-3	Isoform 3 of Peptidyl-prolyl cis-trans isomerase E , GN=PPIE	3	6	3	314		34.969	6.38
P49916	DNA ligase 3 , GN=LIG3	5	7	5	1009		112.835	9.01
Q14141	Septin-6 , GN=SEPT6	6	7	2	434		49.685	6.67
Q9H944	Mediator of RNA polymerase II transcription subunit 20 , GN=MED20	1	5	1	212		23.206	6.87
Q9BTC0	Death-inducer obliterator 1 , GN=DIDO1	5	7	5	2240		243.723	7.88
Q12768	WASH complex subunit 5 , GN=WASHC5	2	7	2	1159		134.201	6.98
Q96HN2	Adenosylhomocysteinase 3 , GN=AHCYL2	5	6	4	611		66.678	7.36
P11388-4	Isoform 4 of DNA topoisomerase 2-alpha , GN=TOP2A	5	7	2	1612		182.567	8.63
Q92615	La-related protein 4B , GN=LARP4B	2	6	2	738		80.503	6.92
P49841-2	Isoform 2 of Glycogen synthase kinase-3 beta , GN=GSK3B	2	6	2	433		48.004	8.78
Q9NXR1	Nuclear distribution protein nudE homolog 1 , GN=NDE1	3	7	3	346		38.785	5.27
P08648	Integrin alpha-5 , GN=ITGA5	3	6	3	1049		114.465	5.77
P40855	Peroxisomal biogenesis factor 19 , GN=PEX19	3	5	3	299		32.786	4.34
P51116	Fragile X mental retardation syndrome-related protein 2 , GN=FXR2	3	6	3	673		74.178	6.23
Q15124	Phosphoglucomutase-like protein 5 ,	3	7	3	567		62.186	7.21
P35813-3	Isoform 3 of Protein phosphatase 1A , GN=PPM1A	4	7	3	455		51.333	7.11

Q9NX46	Poly(ADP-ribose) glycohydrolase ARH3 , GN=ADPRHL2	5	7	5	363		38.922	5.07
Q9UIC8-2	Isoform 2 of Leucine carboxyl methyltransferase 1 , GN=LCMT1	3	7	3	357		41.079	7.01
Q8NEC7	Glutathione S-transferase C-terminal domain-containing protein , GN=GSTCD	2	7	2	633		71.033	7.78
Q9H3G5	Probable serine carboxypeptidase CPVL , GN=CPVL	1	6	1	476		54.129	5.62
P07947	Tyrosine-protein kinase Yes , GN=YES1	4	6	3	543		60.763	6.74
Q9BV79	Enoyl-[acyl-carrier-protein] reductase, mitochondrial , GN=MECR	1	6	1	373		40.436	8.76
P16615	Sarcoplasmic/endoplasmic reticulum calcium ATPase 2 , GN=ATP2A2	2	5	2	1042		114.683	5.34
Q8IVM0-2	Isoform 2 of Coiled-coil domain-containing protein 50 , GN=CCDC50	2	6	2	482		56.305	8.21
Q969G6	Riboflavin kinase , GN=RFK	2	6	2	155		17.612	8.13
O14936	Peripheral plasma membrane protein CASK , GN=CASK	2	5	2	926		105.056	6.43
P51571	Translocon-associated protein subunit delta , GN=SSR4	3	7	3	173		18.987	6.15
Q9NXG2	THUMP domain-containing protein 1 , GN=THUMPD1	2	7	2	353		39.291	7.88
O60762	Dolichol-phosphate mannosyltransferase subunit 1 , GN=DPM1	4	7	4	260		29.616	9.57
Q9H9H4	Vacuolar protein sorting-associated protein 37B , GN=VPS37B	3	6	3	285		31.287	7.34
Q9BUL8	Programmed cell death protein 10 ,	3	6	3	212		24.686	8.19
O76031	ATP-dependent Clp protease ATP-binding subunit clpX-like, mitochondrial , GN=CLPX	3	7	3	633		69.181	7.58
O00193	Small acidic protein , GN=SMAP	2	5	2	183	20.32		4.72
Q7Z3E5	LisH domain-containing protein ARMC9 , GN=ARMC9	2	5	2	817		91.704	6.2
O95989	Diphosphoinositol polyphosphate phosphohydrolase 1 , GN=NUDT3	2	7	2	172		19.459	6.34
Q9Y371-2	Isoform 2 of Endophilin-B1 , GN=SH3GLB1	4	6	4	386		43.169	5.68
O43432-3	Isoform 3 of Eukaryotic translation initiation factor 4 gamma 3 , GN=EIF4G3	4	6	3	1591		177.169	5.39
Q9Y467	Sal-like protein 2 , GN=SALL2	1	6	1	1007		105.244	6.3
Q9H832	Ubiquitin-conjugating enzyme E2 Z ,	1	5	1	354		38.186	5.62
Q9ULU8-4	Isoform 4 of Calcium-dependent secretion activator 1 , GN=CADPS	3	6	3	1358		153.353	5.74
Q7Z4W1	L-xylulose reductase , GN=DCXR	3	7	3	244		25.897	8.1
A6NHR9	Structural maintenance of chromosomes flexible hinge domain-containing protein 1 , GN=SMCHD1	5	7	5	2005		226.231	7.3
P54289	Voltage-dependent calcium channel subunit alpha-2/delta-1 , GN=CACNA2D1	4	6	4	1103	124.49		5.27
Q5VTL8	Pre-mRNA-splicing factor 38B , GN=PRPF38B	1	5	1	546		64.429	10.54
P62424	60S ribosomal protein L7a , GN=RPL7A	3	6	3	266		29.977	10.61
Q6WCQ1-2	Isoform 2 of Myosin phosphatase Rho-interacting protein , GN=MPRIP	3	5	3	1038	118.03		6.39
A0AV96	RNA-binding protein 47 , GN=RBM47	4	5	4	593		64.058	7.68
Q8WXA9-2	Isoform 2 of Splicing regulatory glutamine/lysine-rich protein 1 , GN=SREK1	3	7	3	624		71.606	10.2
Q9NT62	Ubiquitin-like-conjugating enzyme ATG3 , GN=ATG3	3	7	3	314		35.841	4.74
P24941	Cyclin-dependent kinase 2 , GN=CDK2	3	6	3	298		33.908	8.68
Q9NPA8	Transcription and mRNA export factor ENY2 , GN=ENY2	1	5	1	101		11.521	9.33
Q6FI81	Anamorsin , GN=CIAPIN1	3	6	3	312		33.561	5.62
Q6P3W7	SCY1-like protein 2 , GN=SCYL2	4	6	4	929		103.642	8.22
O15460	Prolyl 4-hydroxylase subunit alpha-2 , GN=P4HA2	2	5	2	535		60.864	5.71
Q96EQ0	Small glutamine-rich tetratricopeptide repeat-containing protein beta , GN=SGTB	1	5	1	304		33.408	4.92
Q3ZCW2	Galectin-related protein , GN=LGALSL	1	5	1	172		18.974	5.35

O15164	Transcription intermediary factor 1-alpha , GN=TRIM24	3	6	3	1050		116.757	7.11
P05556-3	Isoform 3 of Integrin beta-1 , GN=ITGB1	5	6	5	825	91.56		5.44
Q9NQP4	Prefoldin subunit 4 , GN=PFDN4	2	6	2	134		15.305	4.53
Q9UNE7	E3 ubiquitin-protein ligase CHIP , GN=STUB1	2	6	2	303		34.834	5.87
O15371	Eukaryotic translation initiation factor 3 subunit D , GN=EIF3D	3	7	3	548		63.932	6.05
Q9Y5A9	YTH domain-containing family protein 2 , GN=YTHDF2	2	6	2	579		62.296	8.79
Q69YQ0	Cytospin-A , GN=SPECC1L	3	6	3	1117		124.525	5.72
P11498	Pyruvate carboxylase, mitochondrial , GN=PC	4	6	4	1178		129.551	6.84
Q9UGV2	Protein NDRG3 , GN=NDRG3	2	6	2	375		41.382	5.31
Q15404	Ras suppressor protein 1 , GN=RSU1	1	4	1	277		31.521	8.65
Q86TG7	Retrotransposon-derived protein PEG10 , GN=PEG10	3	5	3	708		80.122	6.39
P30049	ATP synthase subunit delta, mitochondrial , GN=ATP5D	1	6	1	168		17.479	5.49
Q92609-2	Isoform 2 of TBC1 domain family member 5 , GN=TBC1D5	2	6	2	817		91.159	6.33
Q92879	CUGBP Elav-like family member 1 , GN=CELF1	3	5	3	486	52.03		8.46
O94906	Pre-mRNA-processing factor 6 , GN=PRPF6	3	6	3	941		106.858	8.25
Q9Y5K8	V-type proton ATPase subunit D ,	2	6	2	247		28.245	9.36
Q9BWW4	Single-stranded DNA-binding protein 3 , GN=SSBP3	3	6	3	388		40.395	6.9
Q9BZE1	39S ribosomal protein L37, mitochondrial , GN=MRPL37	2	5	2	423		48.087	8.59
O15217	Glutathione S-transferase A4 , GN=GSTA4	1	6	1	222		25.688	8.27
Q4KMG0	Cell adhesion molecule-related/down-regulated by oncogenes , GN=CDON	2	5	2	1287		139.059	6.51
Q9H2H8	Peptidyl-prolyl cis-trans isomerase-like 3 , GN=PPIL3	4	6	4	161		18.143	6.79
Q13492	Phosphatidylinositol-binding clathrin assembly protein , GN=PICALM	2	6	1	652	70.71		7.9
Q16527	Cysteine and glycine-rich protein 2 ,	1	5	1	193	20.94		8.62
Q96KR1	Zinc finger RNA-binding protein , GN=ZFR	3	6	3	1074		116.939	9.04
Q9UPY6	Wiskott-Aldrich syndrome protein family member 3 , GN=WASF3	3	6	3	502		55.259	6.43
Q13642	Four and a half LIM domains protein 1 ,	1	7	1	323		36.239	8.97
P57740	Nuclear pore complex protein Nup107 , GN=NUP107	4	6	4	925		106.307	5.43
Q92581-2	Isoform 2 of Sodium/hydrogen exchanger 6 , GN=SLC9A6	1	6	1	701		77.867	6.44
Q96JG6	Syndetin , GN=VPS50	4	6	4	964		111.104	6.2
P62857	40S ribosomal protein S28 , GN=RPS28	2	6	2	69		7.836	10.7
P20073	Annexin A7 , GN=ANXA7	2	5	2	488		52.706	5.68
Q06830	Peroxiredoxin-1 , GN=PRDX1	2	7	2	199		22.096	8.13
Q9BW83	Intraflagellar transport protein 27 homolog , GN=IFT27	2	6	2	186		20.467	5.41
P05026	Sodium/potassium-transporting ATPase subunit beta-1 , GN=ATP1B1	3	6	3	303		35.039	8.53
Q96C23	Aldose 1-epimerase , GN=GALM	3	6	3	342		37.742	6.65
O60826	Coiled-coil domain-containing protein 22 , GN=CCDC22	3	5	3	627		70.712	6.74
Q9NRN7	L-aminoadipate-semialdehyde dehydrogenase-phosphopantetheinyl transferase , GN=AASDHPPT	3	6	3	309		35.753	6.8
Q96T25	Zinc finger protein ZIC 5 , GN=ZIC5	1	5	1	663		68.404	8.76
Q9Y450	HBS1-like protein , GN=HBS1L	2	6	2	684		75.426	6.61
P29083	General transcription factor IIE subunit 1 , GN=GTF2E1	2	6	2	439		49.421	4.82
P98160	Basement membrane-specific heparan sulfate proteoglycan core protein , GN=HSPG2	4	6	4	4391		468.532	6.51
Q86U38	Nucleolar protein 9 , GN=NOP9	3	6	3	636		69.394	7.28
P61019	Ras-related protein Rab-2A , GN=RAB2A	3	6	1	212		23.531	6.54
Q6PGP7	Tetratricopeptide repeat protein 37 ,	5	5	5	1564		175.375	7.53

P08559-4	Isoform 4 of Pyruvate dehydrogenase E1 component subunit alpha, somatic form, mitochondrial , GN=PDHA1	3	7	3	428	47.549	8.54
Q9NQ88	Fructose-2,6-bisphosphatase TIGAR ,	3	6	3	270	30.043	7.69
A2A3N6	Putative PIP5K1A and PSMD4-like protein , GN=PIPSL PE=5	3	7	1	862	94.988	5.71
P84103	Serine/arginine-rich splicing factor 3 ,	4	7	3	164	19.318	11.65
Q14118	Dystroglycan , GN=DAG1	1	5	1	895	97.381	8.56
P40616	ADP-ribosylation factor-like protein 1 ,	2	6	2	181	20.404	5.72
Q6UXD5	Seizure 6-like protein 2 , GN=SEZ6L2	3	5	3	910	97.498	4.89
O75688	Protein phosphatase 1B , GN=PPM1B	3	6	2	479	52.609	5.05
Q96IJ6-2	Isoform 2 of Mannose-1-phosphate guanyltransferase alpha , GN=GMPPA	2	5	2	473	51.985	7.21
Q9H9E3	Conserved oligomeric Golgi complex subunit 4 , GN=COG4	3	6	3	785	89.026	5.19
Q00613	Heat shock factor protein 1 , GN=HSF1	2	5	2	529	57.225	5.19
P62753	40S ribosomal protein S6 , GN=RPS6	2	5	2	249	28.663	10.84
Q9H4A3-7	Isoform 6 of Serine/threonine-protein kinase WNK1 , GN=WNK1	3	6	3	2833	299.54	6.48
Q6PD62	RNA polymerase-associated protein CTR9 homolog , GN=CTR9	1	5	1	1173	133.42	6.77
P51654-3	Isoform 3 of Glypican-3 , GN=GPC3	2	5	2	603	68.369	6.43
P19404	NADH dehydrogenase [ubiquinone] flavoprotein 2, mitochondrial , GN=NDUFV2	3	6	3	249	27.374	8.06
Q15545	Transcription initiation factor TFIID subunit 7 , GN=TAF7	2	4	2	349	40.234	5.2
Q9NTX5	Ethylmalonyl-CoA decarboxylase ,	3	6	3	307	33.677	8.21
P14735	Insulin-degrading enzyme , GN=IDE	5	6	5	1019	117.893	6.61
P30154-2	Isoform 2 of Serine/threonine-protein phosphatase 2A 65 kDa regulatory subunit A beta isoform , GN=PPP2R1B	3	7	1	667	73.538	5.07
P49815	Tuberin , GN=TSC2	3	5	3	1807	200.481	7.31
Q13619	Cullin-4A , GN=CUL4A	3	6	2	759	87.624	8.13
O75155	Cullin-associated NEDD8-dissociated protein 2 , GN=CAND2	4	6	3	1236	135.171	5.68
Q8IY81	pre-rRNA processing protein FTSJ3 ,	2	5	2	847	96.499	8.4
O94813	Slit homolog 2 protein , GN=SLIT2	3	6	3	1529	169.759	7.06
P36959	GMP reductase 1 , GN=GMPR	2	5	2	345	37.395	7.06
P41225	Transcription factor SOX-3 , GN=SOX3	2	6	2	446	45.182	9.77
Q9NRF9	DNA polymerase epsilon subunit 3 ,	2	5	2	147	16.849	4.74
Q96G01	Protein bicaudal D homolog 1 , GN=BICD1	2	5	2	975	110.682	5.81
O43295	SLIT-ROBO Rho GTPase-activating protein 3 , GN=SRGAP3	3	6	3	1099	124.425	6.68
Q9UHY7	Enolase-phosphatase E1 , GN=ENOPH1	2	5	2	261	28.914	4.78
Q8IV38	Ankyrin repeat and MYND domain-containing protein 2 , GN=ANKMY2	3	6	3	441	49.267	6.25
O60879	Protein diaphanous homolog 2 , GN=DIAPH2	4	6	4	1101	125.49	6.58
Q14320	Protein FAM50A , GN=FAM50A	4	6	4	339	40.216	6.83
Q9Y3C8	Ubiquitin-fold modifier-conjugating enzyme 1 , GN=UFC1	1	5	1	167	19.446	7.4
Q99700	Ataxin-2 , GN=ATXN2	3	5	3	1313	140.196	9.57
Q9NRA8-3	Isoform 3 of Eukaryotic translation initiation factor 4E transporter , GN=EIF4ENIF1	2	5	2	986	108.262	8.32
O75475	PC4 and SFRS1-interacting protein , GN=PSIP1	4	5	4	530	60.067	9.13
Q7Z5K2-3	Isoform 3 of Wings apart-like protein homolog , GN=WAPL	2	5	2	1275	142.436	6.06
O60925	Prefoldin subunit 1 , GN=PFDN1	2	5	2	122	14.202	6.81
Q13613	Myotubularin-related protein 1 , GN=MTMR1	2	5	2	665	74.631	7.14
O95219	Sorting nexin-4 , GN=SNX4	2	5	2	450	51.876	5.99
Q05519	Serine/arginine-rich splicing factor 11 , GN=SRSF11	2	6	2	484	53.51	10.52
Q7L2E3-2	Isoform 2 of Putative ATP-dependent RNA helicase DHX30 , GN=DHX30	4	6	4	1222	136.03	8.6
P61086	Ubiquitin-conjugating enzyme E2 K ,	3	6	3	200	22.393	5.44
O14776	Transcription elongation regulator 1 , GN=TCERG1	3	6	3	1098	123.823	8.65
P09486	SPARC , GN=SPARC	2	5	2	303	34.61	4.84

O00217	NADH dehydrogenase [ubiquinone] iron-sulfur protein 8, mitochondrial , GN=NDUFS8	1	6	1	210	23.69	6.34
Q8N684-3	Isoform 3 of Cleavage and polyadenylation specificity factor subunit 7 , GN=CPSF7	3	5	3	514	56.341	8.87
Q13769	THO complex subunit 5 homolog , GN=THOC5	2	4	2	683	78.458	6.87
Q96P47-4	Isoform 4 of Arf-GAP with GTPase, ANK repeat and PH domain-containing protein 3 , GN=AGAP3	3	5	3	911	97.891	7.96
Q9BZV1	UBX domain-containing protein 6 ,	3	5	3	441	49.723	6.89
O60684	Importin subunit alpha-7 , GN=KPNA6	3	6	3	536	59.991	4.98
Q9UKN8	General transcription factor 3C polypeptide 4 , GN=GTF3C4	4	6	4	822	91.923	6.65
Q9UPT5	Exocyst complex component 7 , GN=EXOC7	3	6	3	735	83.33	6.79
Q06203	Amidophosphoribosyltransferase , GN=PPAT	2	6	2	517	57.362	6.76
Q9UPQ9	Trinucleotide repeat-containing gene 6B protein , GN=TNRC6B	2	5	2	1833	193.883	6.76
Q92506	Estradiol 17-beta-dehydrogenase 8 , GN=HSD17B8	3	6	3	261	26.957	6.54
Q96B54	Zinc finger protein 428 , GN=ZNF428	1	7	1	188	20.468	4.17
Q6GMV3	Putative peptidyl-tRNA hydrolase PTRHD1 , GN=PTRHD1	2	5	2	140	15.795	9.1
Q96A72	Protein mago nashi homolog 2 ,	3	5	3	148	17.265	6.39
Q86TB9	Protein PAT1 homolog 1 , GN=PATL1	2	5	2	770	86.796	6.67
Q969H8	Myeloid-derived growth factor , GN=MYDGF	3	6	3	173	18.783	6.68
P61006	Ras-related protein Rab-8A , GN=RAB8A	3	6	2	207	23.653	9.07
O15126	Secretory carrier-associated membrane protein 1 , GN=SCAMP1	3	5	3	338	37.896	7.42
Q13015	Protein AF1q , GN=MLLT11	3	6	3	90	10.055	4.54
O95248-4	Isoform 4 of Myotubularin-related protein 5 , GN=SBF1	2	5	2	1893	210.916	7.11
Q9Y2Q3	Glutathione S-transferase kappa 1 ,	1	5	1	226	25.48	8.41
O60841	Eukaryotic translation initiation factor 5B , GN=EIF5B	4	6	4	1220	138.742	5.49
Q8NBJ7	Sulfatase-modifying factor 2 , GN=SUMF2	4	6	4	301	33.822	8
Q9UI12	V-type proton ATPase subunit H ,	3	6	3	483	55.847	6.48
Q9Y4Z0	U6 snRNA-associated Sm-like protein LSm4 , GN=LSM4	3	6	3	139	15.34	9.99
Q9H444	Charged multivesicular body protein 4b , GN=CHMP4B	3	6	3	224	24.935	4.82
O43815	Striatin , GN=STRN	3	5	3	780	86.079	5.27
Q96EI5-2	Isoform 2 of Transcription elongation factor A protein-like 4 , GN=TCEAL4	2	5	2	358	40.553	7.36
Q5JRA6	Transport and Golgi organization protein 1 homolog , GN=MIA3	2	5	2	1907	213.57	4.84
P55039	Developmentally-regulated GTP-binding protein 2 , GN=DRG2	1	4	1	364	40.72	8.88
Q9BQE5	Apolipoprotein L2 , GN=APOL2	3	6	3	337	37.069	6.74
Q96CS2	HAUS augmin-like complex subunit 1 , GN=HAUS1	1	5	1	278	31.844	5.53
Q8WVM7	Cohesin subunit SA-1 , GN=STAG1	3	5	3	1258	144.336	5.59
Q7Z434	Mitochondrial antiviral-signaling protein , GN=MAVS	2	5	2	540	56.493	5.52
Q8NBT2	Kinetochore protein Spc24 , GN=SPC24	1	4	1	197	22.464	4.7
Q03701	CCAAT/enhancer-binding protein zeta , GN=CEBPZ	1	4	1	1054	120.898	5.94
Q9H307	Pinin , GN=PNN	4	6	4	717	81.565	7.14
Q9UNF0	Protein kinase C and casein kinase substrate in neurons protein 2 , GN=PACSIN2	1	4	1	486	55.704	5.2
Q8WYP5-2	Isoform 2 of Protein ELYS , GN=AHCTF1	2	5	2	2301	255.841	6.58
O96000	NADH dehydrogenase [ubiquinone] 1 beta subcomplex subunit 10 , GN=NDUFB10	3	6	3	172	20.763	8.48
P18621-3	Isoform 3 of 60S ribosomal protein L17 , GN=RPL17	2	5	2	228	26.356	10.1
P08621	U1 small nuclear ribonucleoprotein 70 kDa , GN=SNRNP70	3	6	3	437	51.526	9.94
Q9BQA1	Methylosome protein 50 , GN=WDR77	2	5	2	342	36.701	5.17

Q95817	BAG family molecular chaperone regulator 3 , GN=BAG3	4	5	4	575	61.557	6.95
P37840	Alpha-synuclein , GN=SNCA	2	5	2	140	14.451	4.7
Q04726	Transducin-like enhancer protein 3 , GN=TLE3	2	5	2	772	83.364	7.2
Q12802-2	Isoform 2 of A-kinase anchor protein 13 , GN=AKAP13	4	6	4	2817	307.654	5.2
P09132	Signal recognition particle 19 kDa protein , GN=SRP19	1	6	1	144	16.145	9.85
Q9NRY5	Protein FAM114A2 , GN=FAM114A2	2	5	2	505	55.434	4.88
Q8N6T3	ADP-ribosylation factor GTPase-activating protein 1 , GN=ARFGAP1	3	5	3	406	44.64	5.66
Q13557-11	Isoform Delta 11 of Calcium/calmodulin-dependent protein kinase type II subunit delta , GN=CAMK2D	2	5	2	524	59.114	7.56
Q06787	Synaptic functional regulator FMR1 ,	1	5	1	632	71.131	7.42
Q9HB07	UPF0160 protein MYG1, mitochondrial , GN=C12orf10	3	5	3	376	42.422	6.67
Q15126	Phosphomevalonate kinase , GN=PMVK	3	6	3	192	21.981	5.73
Q9NYB0	Telomeric repeat-binding factor 2-interacting protein 1 , GN=TERF2IP	3	5	3	399	44.233	4.73
Q5T440	Putative transferase CAF17, mitochondrial , GN=IBA57	1	4	1	356	38.131	9.83
Q96SI9	Spermatid perinuclear RNA-binding protein , GN=STRBP	3	6	1	672	73.606	8.72
Q12959-2	Isoform 2 of Disks large homolog 1 ,	3	5	3	926	103.258	5.92
Q8IZL8	Proline-, glutamic acid- and leucine-rich protein 1 , GN=PELP1	3	4	3	1130	119.624	4.34
Q9NWB1-2	Isoform 2 of RNA binding protein fox-1 homolog 1 , GN=RBFOX1	2	5	1	418	44.765	7.37
Q9UQ03	Coronin-2B , GN=CORO2B	1	5	1	480	54.918	8.27
Q6UWE0	E3 ubiquitin-protein ligase LRSAM1 , GN=LRSAM1	1	6	1	723	83.541	5.94
P50213	Isocitrate dehydrogenase [NAD] subunit alpha, mitochondrial , GN=IDH3A	2	5	2	366	39.566	6.92
P29372	DNA-3-methyladenine glycosylase , GN=MPG	2	6	2	298	32.848	9.57
Q9HB90	Ras-related GTP-binding protein C ,	3	5	3	399	44.196	5.1
O43491	Band 4.1-like protein 2 , GN=EPB41L2	4	6	3	1005	112.519	5.44
Q9UKV8	Protein argonaute-2 , GN=AGO2	4	6	3	859	97.146	9.19
Q14C86-6	VPS9 domain-containing protein 1 , GN=GAPVD1	3	5	3	1487	166.064	5.21
Q99447-3	Isoform 3 of Ethanolamine-phosphate cytidylyltransferase , GN=PCYT2	2	5	2	407	45.594	6.73
Q8NAV1	Pre-mRNA-splicing factor 38A , GN=PRPF38A	2	4	2	312	37.453	9.96
Q96CT7	Coiled-coil domain-containing protein 124 , GN=CCDC124	3	6	3	223	25.82	9.54
Q9UG63-2	Isoform 2 of ATP-binding cassette sub-family F member 2 , GN=ABCF2	2	6	2	634	72.397	7.49
Q8WU79	Stromal membrane-associated protein 2 , GN=SMAP2	2	5	2	429	46.755	8.87
Q96JQ0	Protocadherin-16 , GN=DCHS1	4	5	4	3298	345.971	4.94
Q6P996	Pyridoxal-dependent decarboxylase domain-containing protein 1 , GN=PDXDC1	3	6	3	788	86.652	5.38
P51648-2	Isoform 2 of Fatty aldehyde dehydrogenase , GN=ALDH3A2	1	5	1	508	57.633	8.92
Q6P9B6	TLD domain-containing protein 1 , GN=TLDC1	2	5	2	456	50.961	6.24
Q7Z3B4	Nucleoporin p54 , GN=NUP54	4	5	4	507	55.401	7.02
Q96J02	E3 ubiquitin-protein ligase Itchy homolog , GN=ITCH	2	5	2	903	102.738	6.3
Q9Y3D6	Mitochondrial fission 1 protein , GN=FIS1	1	6	1	152	16.927	8.79
Q15007	Pre-mRNA-splicing regulator WTAP ,	1	6	1	396	44.217	5.19
Q9P2R7	Succinate--CoA ligase [ADP-forming] subunit beta, mitochondrial , GN=SUCLA2	2	5	2	463	50.285	7.42
Q96GK7	Fumarylacetoacetate hydrolase domain-containing protein 2A , GN=FAHD2A	3	5	3	314	34.574	8.24
Q92541	RNA polymerase-associated protein RTF1 homolog , GN=RTF1	4	6	4	710	80.265	8.15

P83436	Conserved oligomeric Golgi complex subunit 7 , GN=COG7	3	5	3	770	86.289	5.47
Q9UI36	Dachshund homolog 1 , GN=DACH1	3	5	3	760	78.627	8.51
P04083	Annexin A1 , GN=ANXA1	4	5	4	346	38.69	7.02
O60220	Mitochondrial import inner membrane translocase subunit Tim8 A , GN=TIMM8A	2	6	2	97	10.991	5.16
Q9BZJ0	Crooked neck-like protein 1 , GN=CRNKL1	2	5	2	848	100.389	8
P30533	Alpha-2-macroglobulin receptor-associated protein , GN=LRPAP1	3	5	3	357	41.441	8.78
P46063	ATP-dependent DNA helicase Q1 , GN=RECQL	3	6	3	649	73.41	7.88
Q15345	Leucine-rich repeat-containing protein 41 , GN=LRRC41	3	5	3	812	88.594	8.38
Q6PD74	Alpha- and gamma-adaptin-binding protein p34 , GN=AAGAB	2	5	2	315	34.572	4.64
Q9BTL3	RNMT-activating mini protein ,	1	4	1	118	14.373	8.94
Q9P2N5	RNA-binding protein 27 , GN=RBM27	2	4	2	1060	118.645	9.19
Q15714-2	Isoform 2 of TSC22 domain family protein 1 , GN=TSC22D1	2	5	2	144	15.67	5.2
Q8WUD1	Ras-related protein Rab-2B , GN=RAB2B	3	5	1	216	24.199	7.83
Q9HCE1	Putative helicase MOV-10 , GN=MOV10	2	5	2	1003	113.599	8.82
Q9Y3D8	Adenylate kinase isoenzyme 6 , GN=AK6	1	5	1	172	20.049	4.58
O43639	Cytoplasmic protein NCK2 , GN=NCK2	2	5	2	380	42.889	6.95
A2RRP1	Neuroblastoma-amplified sequence ,	4	5	4	2371	268.401	5.96
O75448	Mediator of RNA polymerase II transcription subunit 24 , GN=MED24	2	5	2	989	110.234	6.95
Q96T51	RUN and FYVE domain-containing protein 1 , GN=RUFY1	3	5	3	708	79.767	5.74
Q9H845	Acyl-CoA dehydrogenase family member 9, mitochondrial , GN=ACAD9	3	5	3	621	68.717	7.96
Q8IUD2	ELKS/Rab6-interacting/CAST family member 1 , GN=ERC1	5	5	5	1116	128.008	5.97
Q6WKZ4	Rab11 family-interacting protein 1 , GN=RAB11FIP1	2	5	2	1283	137.083	5.43
Q8IU85	Calcium/calmodulin-dependent protein kinase type 1D , GN=CAMK1D	3	5	3	385	42.887	7.21
P30626	Sorcin , GN=SRI	2	5	2	198	21.662	5.59
P21912	Succinate dehydrogenase [ubiquinone] iron-sulfur subunit, mitochondrial , GN=SDHB	3	6	3	280	31.609	8.76
Q8WUZ0-2	Isoform 2 of B-cell CLL/lymphoma 7 protein family member C , GN=BCL7C	2	4	2	242	26.406	10.76
Q66K14	TBC1 domain family member 9B ,	2	5	2	1250	140.436	5.25
Q9NW08	DNA-directed RNA polymerase III subunit RPC2 , GN=POLR3B	1	6	1	1133	127.702	8.5
Q8NCN5	Pyruvate dehydrogenase phosphatase regulatory subunit, mitochondrial , GN=PDPR	1	6	1	879	99.301	6.35
O95155-4	Isoform 4 of Ubiquitin conjugation factor E4 B , GN=UBE4B	3	5	3	1353	151.465	5.62
Q15800	Methylsterol monooxygenase 1 , GN=MSMO1	1	4	1	293	35.193	7.23
P06737	Glycogen phosphorylase, liver form ,	4	5	4	847	97.087	7.17
O75340	Programmed cell death protein 6 ,	3	6	3	191	21.855	5.4
Q8IYI6	Exocyst complex component 8 , GN=EXOC8	3	4	3	725	81.747	5.49
O76094	Signal recognition particle subunit SRP72 , GN=SRP72	4	5	4	671	74.56	9.26
P22694-2	Isoform 2 of cAMP-dependent protein kinase catalytic subunit beta , GN=PRKACB	2	5	1	398	46.207	8.69
Q9Y5V3-2	Isoform 2 of Melanoma-associated antigen D1 , GN=MAGED1	3	4	3	834	91.901	5.82
Q86UV5-8	Isoform 8 of Ubiquitin carboxyl-terminal hydrolase 48 , GN=USP48	3	5	3	1047	120.449	6.1
Q16513	Serine/threonine-protein kinase N2 ,	3	5	3	984	111.964	6.3
P62854	40S ribosomal protein S26 , GN=RPS26	1	5	1	115	13.007	11
O75925-2	Isoform 2 of E3 SUMO-protein ligase PIAS1 , GN=PIAS1	2	4	1	653	72.157	7.4
P31321	cAMP-dependent protein kinase type I-beta regulatory subunit , GN=PRKAR1B	1	5	1	381	43.046	5.71
Q9BQG2	Peroxisomal NADH pyrophosphatase NUDT12 , GN=NUDT12	1	4	1	462	52.043	6.83

Q15120-2	Isoform 2 of [Pyruvate dehydrogenase (acetyl-transferring)] kinase isozyme 3, mitochondrial , GN=PDK3	3	5	3	415	48.013	8.69	
Q9Y6C2	EMILIN-1 , GN=EMILIN1	2	5	2	1016	106.629	5.17	
O43583	Density-regulated protein , GN=DENR	1	4	1	198	22.078	5.3	
Q96NT0-2	Isoform 2 of Coiled-coil domain-containing protein 115 , GN=CCDC115	1	4	1	188	20.372	8.34	
Q99538	Legumain , GN=LGMN	1	4	1	433	49.379	6.55	
Q06546	GA-binding protein alpha chain , GN=GABPA	2	4	2	454	51.263	4.97	
Q9Y2G5	GDP-fucose protein O-fucosyltransferase 2 , GN=POFUT2	2	5	2	429	49.944	6.6	
Q27J81	Inverted formin-2 , GN=INF2	3	5	3	1249	135.54	5.38	
P52434	DNA-directed RNA polymerases I, II, and III subunit RPABC3 , GN=POLR2H	2	5	2	150	17.132	4.68	
O14519	Cyclin-dependent kinase 2-associated protein 1 , GN=CDK2AP1	1	5	1	115	12.357	9.38	
Q9UH62	Armadillo repeat-containing X-linked protein 3 , GN=ARMCX3	2	4	2	379	42.474	8.37	
P10644	cAMP-dependent protein kinase type I-alpha regulatory subunit , GN=PRKAR1A	2	5	2	381	42.955	5.35	
O00506	Serine/threonine-protein kinase 25 ,	2	5	1	426	48.082	6.74	
Q9P0R6	GSK3B-interacting protein , GN=GSKIP	1	3	1	139	15.638	4.48	
Q9UHA4	Ragulator complex protein LAMTOR3 , GN=LAMTOR3	1	4	1	124	13.614	7.34	
Q9UNH7	Sorting nexin-6 , GN=SNX6	3	4	3	406	46.62	6.16	
Q9NWY4	Histone PARylation factor 1 , GN=HPF1	4	5	4	346	39.411	6.8	
P52594-4	Isoform 4 of Arf-GAP domain and FG repeat-containing protein 1 , GN=AGFG1	1	5	1	584	60.729	8.56	
Q8IWJ2	GRIP and coiled-coil domain-containing protein 2 , GN=GCC2	2	5	2	1684	195.789	5.14	
P98095-2	Isoform 2 of Fibulin-2 , GN=FBLN2	1	5	1	1231	131.775	4.86	
Q9NTJ4-4	Isoform 4 of Alpha-mannosidase 2C1 , GN=MAN2C1	2	3	2	1057	117.112	6.73	
O14787-2	Isoform 2 of Transportin-2 , GN=TNPO2	3	5	1	887	100.343	4.98	
Q9NXA8	NAD-dependent protein deacylase sirtuin-5, mitochondrial , GN=SIRT5	2	5	2	310	33.859	8.47	
P62244	40S ribosomal protein S15a , GN=RPS15A	1	4	1	130	14.83	10.13	
O75781	Paralemmin-1 , GN=PALM	2	4	2	387	42.05		5
O95163	Elongator complex protein 1 , GN=ELP1	4	5	4	1332	150.159	5.94	
O95639	Cleavage and polyadenylation specificity factor subunit 4 , GN=CPSF4	2	5	2	269	30.235	8.31	
Q5VW36	Focadhesin , GN=FOCAD	3	5	3	1801	199.944	6.62	
Q9UKG1	DCC-interacting protein 13-alpha , GN=APPL1	2	4	2	709	79.614	5.41	
Q9H7L9	Sin3 histone deacetylase corepressor complex component SDS3 , GN=SUDS3	1	4	1	328	38.112	5.66	
Q92785	Zinc finger protein ubi-d4 , GN=DPF2	1	4	1	391	44.127	6.33	
Q92614	Unconventional myosin-XVIIIa , GN=MYO18A	4	4	4	2054	232.971	6.3	
O00566	U3 small nucleolar ribonucleoprotein protein MPP10 , GN=MPHOSPH10	1	4	1	681	78.816	4.86	
Q96GA7	Serine dehydratase-like , GN=SDSL	3	5	3	329	34.652	6.89	
Q9Y6A4	Cilia- and flagella-associated protein 20 , GN=CFAP20	2	5	2	193	22.76	9.76	
Q13045	Protein flightless-1 homolog , GN=FLII	4	4	4	1269	144.659	6.05	
Q86YS7-5	Isoform 5 of C2 domain-containing protein 5 , GN=C2CD5	2	4	2	1054	116.394	6.24	
Q9H5X1	MIP18 family protein FAM96A , GN=FAM96A	1	5	1	160	18.343	4.88	
Q9BZL4	Protein phosphatase 1 regulatory subunit 12C , GN=PPP1R12C	3	5	3	782	84.83	5.57	
Q8WUA2	Peptidyl-prolyl cis-trans isomerase-like 4 , GN=PPIL4	2	5	2	492	57.189	5.92	
Q7L5N1	COP9 signalosome complex subunit 6 , GN=COPS6	3	5	3	327	36.14	5.73	
Q9UHG2	ProSAAS , GN=PCSK1N	1	4	1	260	27.356	6.62	
O75348	V-type proton ATPase subunit G 1 , GN=ATP6V1G1	1	5	1	118	13.749	8.79	
Q8WX93	Palladin , GN=PALLD	3	5	3	1383	150.47	7.09	

Q99627	COP9 signalosome complex subunit 8 , GN=COPS8	3	5	3	209	23.211	5.38
Q7L0Y3	Mitochondrial ribonuclease P protein 1 , GN=TRMT10C	2	4	2	403	47.317	9.36
P63173	60S ribosomal protein L38 , GN=RPL38	2	5	2	70	8.213	10.1
Q96FN4	Copine-2 , GN=CPNE2	2	5	2	548	61.151	6.07
Q9UKY7	Protein CDV3 homolog , GN=CDV3	2	4	2	258	27.318	6.4
P39023	60S ribosomal protein L3 , GN=RPL3	3	5	3	403	46.08	10.18
Q9Y4B6	DDB1- and CUL4-associated factor 1 ,	2	4	2	1507	168.9	5.06
Q9H488	GDP-fucose protein O-fucosyltransferase 1 , GN=POFUT1	2	4	2	388	43.927	8.53
O00471	Exocyst complex component 5 , GN=EXOC5	2	5	2	708	81.801	6.71
Q5SRD1	membrane translocase subunit Tim23B , GN=TIMM23B PE=5	2	4	2	257	28.03	9.39
Q8TB36	Ganglioside-induced differentiation-associated protein 1 , GN=GDAP1	3	4	3	358	41.32	8.34
P60510	Serine/threonine-protein phosphatase 4 catalytic subunit , GN=PPP4C	2	4	2	307	35.057	5.06
P08754	Guanine nucleotide-binding protein G(k) subunit alpha , GN=GNAI3	2	5	1	354	40.506	5.69
Q13057-2	Isoform 2 of Bifunctional coenzyme A synthase , GN=COASY	2	5	2	593	65.299	7.64
O75962	Triple functional domain protein , GN=TRIO	2	4	2	3097	346.683	6.37
Q96JH7	Deubiquitinating protein VCIP135 ,	2	4	2	1222	134.236	7.2
Q12824	SWI/SNF-related matrix-associated actin-dependent regulator of chromatin subfamily B member 1 , GN=SMARCB1	2	4	2	385	44.113	6.23
O00462	Beta-mannosidase , GN=MANBA PE=2	3	5	3	879	100.831	5.52
O95071	E3 ubiquitin-protein ligase UBR5 , GN=UBR5	3	4	3	2799	309.158	5.85
Q9H9Q4-2	Isoform 2 of Non-homologous end-joining factor 1 , GN=NHEJ1	1	4	1	316	35.269	5.26
Q92733	Proline-rich protein PRCC , GN=PRCC	3	4	3	491	52.386	5.1
Q9H977	WD repeat-containing protein 54 ,	1	4	1	334	35.868	6.2
Q9BVM4	Gamma-glutamylaminecyclotransferase , GN=GGACT	1	5	1	153	17.318	6.87
Q9UL15-2	Isoform 2 of BAG family molecular chaperone regulator 5 , GN=BAG5	2	4	2	488	55.992	6
Q9NP72-2	Isoform 2 of Ras-related protein Rab-18 , GN=RAB18	3	5	3	235	26.393	5.73
P52895	Aldo-keto reductase family 1 member C2 , GN=AKR1C2	1	4	1	323	36.712	7.49
P61925	cAMP-dependent protein kinase inhibitor alpha , GN=PKIA	2	4	2	76	7.984	4.54
Q13595	Transformer-2 protein homolog alpha , GN=TRA2A	2	5	2	282	32.669	11.27
P84098	60S ribosomal protein L19 , GN=RPL19	2	5	2	196	23.451	11.47
Q4VCS5	Angiomotin , GN=AMOT	1	4	1	1084	118.013	7.64
Q9Y2T2	AP-3 complex subunit mu-1 , GN=AP3M1	2	4	2	418	46.909	6.93
A1X283	SH3 and PX domain-containing protein 2B , GN=SH3PXD2B	3	4	3	911	101.516	8.69
Q9UBW7	Zinc finger MYM-type protein 2 , GN=ZMYM2	2	4	2	1377	154.81	6.34
P62241	40S ribosomal protein S8 , GN=RPS8	4	5	4	208	24.19	10.32
P50548	ETS domain-containing transcription factor ERF , GN=ERF	2	4	2	548	58.666	7.3
Q01433	AMP deaminase 2 , GN=AMPD2	3	5	3	879	100.624	6.93
P63167	Dynein light chain 1, cytoplasmic ,	3	5	2	89	10.359	7.4
Q9UJW0-3	Isoform 3 of Dynactin subunit 4 , GN=DCTN4	2	5	2	467	53.118	7.39
P24821	Tenascin , GN=TNC	2	4	2	2201	240.7	4.89
Q6PJT7	Zinc finger CCCH domain-containing protein 14 , GN=ZC3H14	3	5	3	736	82.823	7.31
P41227	N-alpha-acetyltransferase 10 , GN=NAA10	2	5	2	235	26.442	5.64
Q14694-2	Isoform 2 of Ubiquitin carboxyl-terminal hydrolase 10 , GN=USP10	3	4	3	846	92.54	5.69
P52815	39S ribosomal protein L12, mitochondrial , GN=MRPL12	2	4	2	198	21.335	8.87
Q92890-1	in ER-associated degradation protein 1 , GN=UFD1	2	5	2	343	38.7	6.34

Q60231	Putative pre-mRNA-splicing factor ATP-dependent RNA helicase DHX16 , GN=DHX16	3	5	3	1041		119.189	6.8
Q9Y3B7	39S ribosomal protein L11, mitochondrial , GN=MRPL11	2	4	2	192	20.67		9.91
Q4G176	Acyl-CoA synthetase family member 3, mitochondrial , GN=ACSF3	3	5	3	576		64.089	8.37
Q66PJ3	ADP-ribosylation factor-like protein 6-interacting protein 4 , GN=ARL6IP4	1	4	1	421		44.888	10.93
Q15833-3	Isoform 3 of Syntaxin-binding protein 2 , GN=STXBP2	1	5	1	604		67.652	6.68
Q96AY4	Tetratricopeptide repeat protein 28 ,	5	5	5	2481		270.715	6.89
Q14318-2	Isoform 2 of Peptidyl-prolyl cis-trans isomerase FKBP8 , GN=FKBP8	2	4	2	413		44.621	4.84
Q8NFF5	FAD synthase , GN=FLAD1	3	4	3	587		65.224	6.93
Q6ZN17	Protein lin-28 homolog B , GN=LIN28B	1	4	1	250		27.066	8.91
Q15165	Serum paraoxonase/arylesterase 2 ,	1	4	1	354		39.372	5.6
Q8N0X7	Spartin , GN=SPART	3	5	3	666		72.788	5.91
Q9NTI5	Sister chromatid cohesion protein PDS5 homolog B , GN=PDS5B	2	4	2	1447		164.563	8.47
Q9H9T3	Elongator complex protein 3 , GN=ELP3	4	5	4	547		62.219	8.88
Q14353	Guanidinoacetate N-methyltransferase , GN=GAMT	3	5	3	236		26.301	6.14
O75964	ATP synthase subunit g, mitochondrial , GN=ATP5L	3	5	3	103		11.421	9.64
Q9NR30	Nucleolar RNA helicase 2 , GN=DDX21	3	4	2	783	87.29		9.28
O43264	Centromere/kinetochore protein zw10 homolog , GN=ZW10	3	4	3	779		88.773	6.27
Q9BRJ2	39S ribosomal protein L45, mitochondrial , GN=MRPL45	2	4	2	306		35.328	9.03
Q9H270	Vacuolar protein sorting-associated protein 11 homolog , GN=VPS11	4	5	4	941		107.768	7.05
Q9HCN4-5	Isoform 5 of GPN-loop GTPase 1 , GN=GPN1	2	4	2	388		43.318	5.19
P43897-2	Isoform 2 of Elongation factor Ts, mitochondrial , GN=TSFM	1	4	1	346		37.632	7.72
O15047	Histone-lysine N-methyltransferase SETD1A , GN=SETD1A	1	4	1	1707	185.92		5.14
P02458	Collagen alpha-1(II) chain , GN=COL2A1	3	4	3	1487		141.699	6.92
Q8WXF0	Serine/arginine-rich splicing factor 12 , GN=SRSF12 PE=2	2	5	1	261		30.494	11.69
O00469-2	Isoform 2 of Procollagen-lysine,2-oxoglutarate 5-dioxygenase 2 , GN=PLOD2	4	5	4	758		87.043	6.71
Q96PY5-3	Isoform 2 of Formin-like protein 2 ,	3	4	3	1092		124.029	7.66
O95758-4	Isoform 4 of Polypyrimidine tract-binding protein 3 , GN=PTBP3	2	4	2	558		60.379	9.19
O95716	Ras-related protein Rab-3D , GN=RAB3D	3	4	2	219		24.252	4.93
Q8IXV7	Kelch domain-containing protein 8B , GN=KLHDC8B PE=2	1	4	1	354		37.652	8.27
Q86XZ4	Spermatogenesis-associated serine-rich protein 2 , GN=SPATS2	1	3	1	545		59.508	8.9
P13073	Cytochrome c oxidase subunit 4 isoform 1, mitochondrial , GN=COX4I1	1	5	1	169		19.564	9.51
Q9ULX6	A-kinase anchor protein 8-like , GN=AKAP8L	1	4	1	646		71.604	5.07
Q96TA1	Niban-like protein 1 , GN=FAM129B	2	4	2	746		84.085	6.19
P61247	40S ribosomal protein S3a , GN=RPS3A	2	5	2	264		29.926	9.73
O75582	Ribosomal protein S6 kinase alpha-5 , GN=RPS6KA5	2	4	2	802		89.809	7.11
P07585	Decorin , GN=DCN	1	3	1	359		39.722	8.54
Q5T8P6-3	Isoform 3 of RNA-binding protein 26 ,	3	4	3	980		110.598	9.07
Q9Y277-2	Isoform 2 of Voltage-dependent anion-selective channel protein 3 , GN=VDAC3	2	5	2	284	30.77		8.66
Q03426	Mevalonate kinase , GN=MVK	3	5	3	396		42.424	6.46
Q9NVV4-2	Isoform 2 of Poly(A) RNA polymerase, mitochondrial , GN=MTPAP	2	4	1	712		78.784	8.28
Q9UBG0	C-type mannose receptor 2 , GN=MRC2	1	5	1	1479		166.568	5.83
P67812-3	Isoform 3 of Signal peptidase complex catalytic subunit SEC11A , GN=SEC11A	1	5	1	185	21.44		9.42
Q9UIL1	Short coiled-coil protein , GN=SCOC	3	4	3	159		18.034	8.85

P26440	Isovaleryl-CoA dehydrogenase, mitochondrial , GN=IVD	2	5	2	423	46.29	8.19
Q96ST2	Protein IWS1 homolog , GN=IWS1	3	4	3	819	91.899	4.69
P50225	Sulfotransferase 1A1 , GN=SULT1A1	2	4	2	295	34.143	6.62
Q99807	5-demethoxyubiquinone hydroxylase, mitochondrial , GN=COQ7	1	5	1	217	24.262	8.59
Q15102	Platelet-activating factor acetylhydrolase IB subunit gamma , GN=PAFAH1B3	4	4	4	231	25.718	6.84
Q9GZR7	ATP-dependent RNA helicase DDX24 , GN=DDX24	1	4	1	859	96.271	9.06
Q9UN37	Vacuolar protein sorting-associated protein 4A , GN=VPS4A	3	4	3	437	48.867	7.8
Q99959	Plakophilin-2 , GN=PKP2	2	5	2	881	97.355	9.33
P16220-3	Isoform 3 of Cyclic AMP-responsive element-binding protein 1 , GN=CREB1	3	4	1	230	25.429	6.02
Q7Z4S6-4	Isoform 4 of Kinesin-like protein KIF21A , GN=KIF21A	2	4	2	1675	187.15	6.42
O95104	Splicing factor, arginine/serine-rich 15 , GN=SCAF4	3	5	2	1147	125.79	9.55
P36639	7,8-dihydro-8-oxoguanine triphosphatase , GN=NUDT1	2	5	2	197	22.505	5.27
Q9BVL4	Selenoprotein O , GN=SELENOO	1	4	1	669	73.444	5.97
Q9Y4P8	WD repeat domain phosphoinositide-interacting protein 2 , GN=WIPI2	2	4	2	454	49.377	6.46
Q9P000	COMM domain-containing protein 9 , GN=COMMD9	2	4	2	198	21.805	5.88
Q00577	Transcriptional activator protein Pur-alpha , GN=PURA	1	3	1	322	34.889	6.44
O14530	Thioredoxin domain-containing protein 9 , GN=TXNDC9	3	4	3	226	26.517	5.88
Q96C36	Pyrroline-5-carboxylate reductase 2 ,	2	4	2	320	33.616	7.77
Q8IUR7	Armadillo repeat-containing protein 8 , GN=ARMC8	2	5	2	673	75.46	6.73
P41223-2	Isoform 2 of Protein BUD31 homolog , GN=BUD31	1	4	1	145	17.32	8.87
P08134	Rho-related GTP-binding protein RhoC , GN=RHOC	2	4	1	193	21.992	6.58
P40937	Replication factor C subunit 5 , GN=RFC5	1	3	1	340	38.472	7.2
O60610	Protein diaphanous homolog 1 , GN=DIAPH1	3	5	3	1272	141.258	5.41
P52948	Nuclear pore complex protein Nup98-Nup96 , GN=NUP98	4	5	4	1817	197.457	6.4
Q96T60	Bifunctional polynucleotide phosphatase/kinase , GN=PNKP	2	4	2	521	57.04	8.46
Q9BYJ9	YTH domain-containing family protein 1 , GN=YTHDF1	3	4	2	559	60.836	8.79
P62913	60S ribosomal protein L11 , GN=RPL11	1	4	1	178	20.24	9.6
Q96P16	Regulation of nuclear pre-mRNA domain-containing protein 1A , GN=RPRD1A	2	4	1	312	35.698	7.55
P82909	28S ribosomal protein S36, mitochondrial , GN=MRPS36	1	4	1	103	11.459	9.99
O76054	SEC14-like protein 2 , GN=SEC14L2	2	4	2	403	46.116	7.84
P55957-2	Isoform 2 of BH3-interacting domain death agonist , GN=BID	2	4	2	241	26.819	7.08
Q5U5Q3	RNA-binding E3 ubiquitin-protein ligase MEX3C , GN=MEX3C	1	4	1	659	69.323	5
Q4G0F5	Vacuolar protein sorting-associated protein 26B , GN=VPS26B	1	4	1	336	39.13	7.36
Q9Y296	Trafficking protein particle complex subunit 4 , GN=TRAPPC4	2	4	2	219	24.325	6.21
Q9BUP0	EF-hand domain-containing protein D1 , GN=EFHD1	2	5	1	239	26.911	5.39
Q92466	DNA damage-binding protein 2 , GN=DDB2	2	5	2	427	47.833	9.47
P51687	Sulfite oxidase, mitochondrial , GN=SUOX	1	3	1	545	60.245	6.11
Q9Y5S9	RNA-binding protein 8A , GN=RBM8A	3	4	3	174	19.877	5.72
Q9BVL2	Nucleoporin p58/p45 , GN=NUP58	1	4	1	599	60.86	9.33
Q9NPJ6	Mediator of RNA polymerase II transcription subunit 4 , GN=MED4	2	5	2	270	29.727	5.1

Q9NXC5	GATOR complex protein MIOS , GN=MIOS	1	3	1	875	98.521	6.73	
Q7Z3J2	UPF0505 protein C16orf62 , GN=C16orf62	2	4	2	963	109.492	7.21	
Q99614	Tetratricopeptide repeat protein 1 , GN=TTC1	3	4	3	292	33.505	4.84	
Q53EL6	Programmed cell death protein 4 ,	2	4	2	469	51.703	5.21	
Q9UI26-2	Isoform 2 of Importin-11 , GN=IPO11	1	5	1	1015	116.932	5.41	
Q96BH1	E3 ubiquitin-protein ligase RNF25 ,	2	4	2	459	51.187	6.54	
P52735	Guanine nucleotide exchange factor VAV2 , GN=VAV2	2	4	2	878	101.224	7.08	
Q9HCU4	Cadherin EGF LAG seven-pass G-type receptor 2 , GN=CELSR2	2	4	2	2923	317.253	5.31	
Q9UL18	Protein argonaute-1 , GN=AGO1	2	4	1	857	97.152	9.16	
Q6NZY4	Zinc finger CCHC domain-containing protein 8 , GN=ZCCHC8	3	4	3	707	78.529	4.87	
O14976	Cyclin-G-associated kinase , GN=GAK	2	4	2	1311	143.1	5.73	
P39748	Flap endonuclease 1 , GN=FEN1	2	4	2	380	42.566	8.62	
Q92538	Golgi-specific brefeldin A-resistance guanine nucleotide exchange factor 1 , GN=GBF1	3	4	3	1859	206.315	5.73	
O75094-4	Isoform 4 of Slit homolog 3 protein ,	1	5	1	1530	168.452	7.65	
Q96JC1	Vam6/Vps39-like protein , GN=VPS39	1	4	1	886	101.744	6.99	
Q13330-3	Isoform 3 of Metastasis-associated protein MTA1 , GN=MTA1	3	4	3	698	79.143	9.41	
Q9NUW8	Tyrosyl-DNA phosphodiesterase 1 , GN=TDP1	2	4	2	608	68.377	7.65	
Q15031	Probable leucine--tRNA ligase, mitochondrial , GN=LARS2	2	4	2	903	101.911	8.22	
Q9UI09	NADH dehydrogenase [ubiquinone] 1 alpha subcomplex subunit 12 , GN=NDUFA12	1	4	1	145	17.104	9.63	
Q9P273	Teneurin-3 , GN=TENM3 PE=2	2	4	2	2699	300.76	6.42	
Q9BZE9-2	Isoform 2 of Tether containing UBX domain for GLUT4 , GN=ASPSCR1	2	4	2	647	69.947	8.09	
P61421	V-type proton ATPase subunit d 1 , GN=ATP6V0D1	1	3	1	351	40.303		5
Q8IZQ5	Selenoprotein H , GN=SELENOH	2	4	2	122	13.446	9.74	
Q13155	interacting multifunctional protein 2 , GN=AIMP2	3	4	3	320	35.326	8.22	
O43865	S-adenosylhomocysteine hydrolase-like protein 1 , GN=AHCYL1	3	4	2	530	58.913	6.89	
P46782	40S ribosomal protein S5 , GN=RPS5	1	5	1	204	22.862	9.72	
O75608	Acyl-protein thioesterase 1 , GN=LYPLA1	2	5	2	230	24.653	6.77	
Q8N3R9	MAGUK p55 subfamily member 5 , GN=MPP5	4	4	4	675	77.246	6.14	
Q96HE7	ERO1-like protein alpha , GN=ERO1A	1	4	1	468	54.358	5.68	
Q14165	Malectin , GN=MLEC	1	4	1	292	32.214	5.41	
Q99504	Eyes absent homolog 3 , GN=EYA3	1	4	1	573	62.624	5.21	
P07311	Acylphosphatase-1 , GN=ACYP1	2	5	2	99	11.254	9.31	
Q13825	Methylglutaconyl-CoA hydratase, mitochondrial , GN=AUH	3	4	3	339	35.586	9.48	
O43805	Sjoegren syndrome nuclear autoantigen 1 , GN=SSNA1	2	5	2	119	13.588	5.38	
Q9UBI1	COMM domain-containing protein 3 , GN=COMMD3	1	5	1	195	22.137	5.99	
Q4V328	GRIP1-associated protein 1 , GN=GRIPAP1	4	4	4	841	95.931	5.11	
Q96FZ7	Charged multivesicular body protein 6 , GN=CHMP6	1	4	1	201	23.47	5.31	
O00233	26S proteasome non-ATPase regulatory subunit 9 , GN=PSMD9	3	5	3	223	24.667	6.95	
O60831	PRA1 family protein 2 , GN=PRAF2	2	4	2	178	19.246	9.19	
P18887	DNA repair protein XRCC1 , GN=XRCC1	2	5	2	633	69.434	6.39	
Q9Y5B0	RNA polymerase II subunit A C-terminal domain phosphatase , GN=CTDP1	1	4	1	961	104.335	5.27	
O43424	Glutamate receptor ionotropic, delta-2 , GN=GRID2	1	5	1	1007	113.284	6.07	
Q13247	Serine/arginine-rich splicing factor 6 ,	2	5	2	344	39.563	11.43	
Q86VN1	Vacuolar protein-sorting-associated protein 36 , GN=VPS36	3	4	3	386	43.789	7.2	
Q9NW64	Pre-mRNA-splicing factor RBM22 ,	2	4	2	420	46.865	8.54	
Q14137	Ribosome biogenesis protein BOP1 ,	2	3	2	746	83.577	6.19	
Q5T5P2	Sickle tail protein homolog , GN=KIAA1217	3	5	3	1943	213.983	7.06	

Q13084	39S ribosomal protein L28, mitochondrial , GN=MRPL28	1	3	1	256		30.138	8.29	
Q8N158	Glypican-2 , GN=GPC2 PE=2	2	4	2	579	62.79			8
O00468	Agrin , GN=AGRN	3	4	3	2067		217.092	6.39	
Q9GZP4	PITH domain-containing protein 1 ,	2	4	2	211		24.163	5.74	
Q9NP79	Vacuolar protein sorting-associated protein VTA1 homolog , GN=VTA1	1	3	1	307		33.858	6.29	
P34896	Serine hydroxymethyltransferase, cytosolic , GN=SHMT1	2	4	1	483		53.049	7.71	
Q9NVD7	Alpha-parvin , GN=PARVA	2	4	2	372		42.217	5.95	
O15514	DNA-directed RNA polymerase II subunit RPB4 , GN=POLR2D	2	4	2	142		16.301	4.79	
P61970	Nuclear transport factor 2 , GN=NUTF2	1	4	1	127		14.469	5.38	
P19525	Interferon-induced, double-stranded RNA-activated protein kinase , GN=EIF2AK2	2	4	2	551		62.056	8.4	
A6NHG4	D-dopachrome decarboxylase-like protein , GN=DDTL PE=2	1	4	1	134		14.186	6.29	
Q9Y2S7	Polymerase delta-interacting protein 2 , GN=POLDIP2	3	4	3	368		42.007	8.63	
Q9Y5L0-1	Isoform 1 of Transportin-3 , GN=TNPO3	1	4	1	957		107.931	5.71	
Q96RR4	Calcium/calmodulin-dependent protein kinase kinase 2 , GN=CAMKK2	3	4	3	588		64.705	6.68	
Q96CW1	AP-2 complex subunit mu , GN=AP2M1	2	4	2	435		49.623	9.54	
Q53FT3	Protein Hikeshi , GN=HIKESHI	1	4	1	197		21.614	5.45	
P09382	Galectin-1 , GN=LGALS1	1	3	1	135		14.706	5.5	
Q8N668	COMM domain-containing protein 1 , GN=COMMD1	1	3	1	190		21.165	6.2	
P49761	Dual specificity protein kinase CLK3 ,	1	4	1	638		73.469	9.92	
Q9Y530	O-acetyl-ADP-ribose deacetylase 1 ,	2	4	2	152		17.014	8.31	
Q96HR3	Mediator of RNA polymerase II transcription subunit 30 , GN=MED30	1	4	1	178		20.264	8.27	
P11279	Lysosome-associated membrane glycoprotein 1 , GN=LAMP1	1	4	1	417		44.854	8.75	
Q9BYD3	39S ribosomal protein L4, mitochondrial , GN=MRPL4	2	4	2	311		34.897	9.72	
Q96T37	Putative RNA-binding protein 15 , GN=RBM15	2	4	2	977		107.124	10.08	
Q5R3I4	Tetratricopeptide repeat protein 38 ,	3	5	3	469		52.753	5.99	
Q9Y4F1-2	Isoform 2 of FERM, ARHGEF and pleckstrin domain-containing protein 1 , GN=FARP1	3	4	3	1076		122.037	8.48	
O76021	Ribosomal L1 domain-containing protein 1 , GN=RSL1D1	2	4	2	490		54.939	10.13	
Q96BD5-3	Isoform 3 of PHD finger protein 21A ,	3	3	3	681		74.935	9.45	
Q08170	Serine/arginine-rich splicing factor 4 ,	2	4	2	494		56.645	11.52	
Q9Y6Q2-2	Isoform 2 of Stonin-1 , GN=STON1	2	3	2	1158		129.146	5.27	
Q9Y5Q8-3	Isoform 3 of General transcription factor 3C polypeptide 5 , GN=GTF3C5	2	4	2	526		60.331	7.08	
Q14376	UDP-glucose 4-epimerase , GN=GALE	3	4	3	348		38.257	6.73	
Q9NZW5	MAGUK p55 subfamily member 6 , GN=MPP6	2	4	2	540		61.079	6.18	
O94776	Metastasis-associated protein MTA2 ,	3	4	3	668		74.976	9.66	
P11387	DNA topoisomerase 1 , GN=TOP1	1	4	1	765		90.669	9.31	
P16219	Short-chain specific acyl-CoA dehydrogenase, mitochondrial , GN=ACADS	2	4	2	412		44.269	7.99	
Q93050-3	Isoform 3 of V-type proton ATPase 116 kDa subunit a isoform 1 , GN=ATP6V0A1	1	3	1	838		96.537	6.64	
Q92804	TATA-binding protein-associated factor 2N , GN=TAF15	2	4	1	592		61.793	8.02	
Q96KP1	Exocyst complex component 2 , GN=EXOC2	3	4	3	924		104	6.9	
Q9UHL4	Dipeptidyl peptidase 2 , GN=DPP7	2	4	2	492		54.307	6.32	
Q9Y2V2	Calcium-regulated heat-stable protein 1 , GN=CARHSP1	3	4	3	147		15.882	8.21	
O95983	Methyl-CpG-binding domain protein 3 , GN=MBD3	3	4	3	291		32.823	5.34	
P17096-3	Isoform HMG-R of High mobility group protein HMG-I/HMG-Y , GN=HMGA1	1	4	1	179		19.682	11.75	
P55011	Solute carrier family 12 member 2 ,	1	4	1	1212		131.364	6.4	
O14548	Cytochrome c oxidase subunit 7A-related protein, mitochondrial , GN=COX7A2L	2	4	2	114		12.607	9.42	

P61353	60S ribosomal protein L27 , GN=RPL27	2	4	2	136		15.788	10.56	
Q5TF21	Protein SOGA3 , GN=SOGA3 PE=3	3	4	3	947		103.136	6.06	
P27144	Adenylate kinase 4, mitochondrial , GN=AK4	2	4	2	223		25.252	8.4	
Q8WVC0	RNA polymerase-associated protein LEO1 , GN=LEO1	2	3	2	666		75.359	4.51	
Q12962	Transcription initiation factor TFIID subunit 10 , GN=TAF10	1	3	1	218		21.698	6.57	
Q96E14	RecQ-mediated genome instability protein 2 , GN=RMI2	1	4	1	147		15.855	7.9	
P16333	Cytoplasmic protein NCK1 , GN=NCK1	3	4	3	377		42.837	6.47	
Q14344	Guanine nucleotide-binding protein subunit alpha-13 , GN=GNA13	2	4	2	377		44.022		8
P02649	Apolipoprotein E , GN=APOE	3	4	3	317		36.132	5.73	
Q9Y676	28S ribosomal protein S18b, mitochondrial , GN=MRPS18B	3	4	3	258		29.377	9.38	
O43747-2	Isoform 2 of AP-1 complex subunit gamma-1 , GN=AP1G1	2	4	2	825		91.664	6.8	
Q9BRJ7-2	Isoform 2 of Tudor-interacting repair regulator protein , GN=NUDT16L1	1	4	1	247		25.958	9.13	
P04179	Superoxide dismutase [Mn], mitochondrial , GN=SOD2	1	3	1	222		24.707	8.25	
Q9H3P7	Golgi resident protein GCP60 , GN=ACBD3	2	4	2	528		60.556	5.06	
O94855-2	Isoform 2 of Protein transport protein Sec24D , GN=SEC24D	3	4	3	1033		113.007	7.25	
P06241	Tyrosine-protein kinase Fyn , GN=FYN	2	4	1	537		60.723	6.67	
O60613	Selenoprotein F , GN=SELENOF	2	4	2	162	17.78		5.03	
Q5T7W0	Zinc finger protein 618 , GN=ZNF618	2	4	2	954		104.889	7.09	
Q96KQ7	Histone-lysine N-methyltransferase EHMT2 , GN=EHMT2	2	4	2	1210		132.287	5.45	
O75431	Metaxin-2 , GN=MTX2	2	4	2	263		29.744	6.29	
Q9NZ32	Actin-related protein 10 , GN=ACTR10	2	3	2	417		46.277	7.37	
Q9BQ39	ATP-dependent RNA helicase DDX50 , GN=DDX50	2	3	1	737		82.514	9.17	
Q9GZZ1	N-alpha-acetyltransferase 50 , GN=NAA50	2	4	2	169		19.386	8.81	
Q12830	Nucleosome-remodeling factor subunit BPTF , GN=BPTF	4	4	4	3046		338.054	6.54	
Q5SZK8	FRAS1-related extracellular matrix protein 2 , GN=FREM2	3	4	3	3169		350.937	5.03	
P67870	Casein kinase II subunit beta , GN=CSNK2B	1	3	1	215		24.926	5.55	
O60885	Bromodomain-containing protein 4 ,	4	4	4	1362		152.124	9.19	
Q9Y2Q5	Ragulator complex protein LAMTOR2 , GN=LAMTOR2	2	4	2	125		13.499	5.4	
Q9UK61	Protein TASOR , GN=FAM208A	3	4	3	1670		188.914	5.8	
Q9BW27	Nuclear pore complex protein Nup85 , GN=NUP85	2	4	2	656		74.971	5.55	
Q9Y3L5	Ras-related protein Rap-2c , GN=RAP2C	1	4	1	183		20.731	4.94	
C9JLW8	Mapk-regulated corepressor-interacting protein 1 , GN=MCRIP1	2	4	2	97		10.914	9.41	
P30047	GTP cyclohydrolase 1 feedback regulatory protein , GN=GCHFR	1	3	1	84		9.692	6.54	
P20396	Pro-thyrotropin-releasing hormone , GN=TRH	1	3	1	242		27.388	5.45	
Q4VC31	Coiled-coil domain-containing protein 58 , GN=CCDC58	1	3	1	144		16.609	7.81	
O75152	Zinc finger CCCH domain-containing protein 11A , GN=ZC3H11A	2	4	2	810		89.076	8.37	
Q9NR09	Baculoviral IAP repeat-containing protein 6 , GN=BIRC6	2	4	2	4857		529.919	6.05	
Q5RKV6	Exosome complex component MTR3 , GN=EXOSC6	2	4	2	272		28.218	6.28	
Q9Y5M8	Signal recognition particle receptor subunit beta , GN=SRPRB	2	4	2	271		29.684	9.04	
Q9BUQ8	Probable ATP-dependent RNA helicase DDX23 , GN=DDX23	4	4	4	820		95.524	9.55	
Q8WU76	Sec1 family domain-containing protein 2 , GN=SCFD2	3	4	3	684		75.079	6.68	
Q9Y248	DNA replication complex GINS protein PSF2 , GN=GINS2	2	4	2	185		21.414	5.44	

Q96CM8-2	Isoform 2 of Acyl-CoA synthetase family member 2, mitochondrial , GN=ACSF2	2	4	2	640		70.577	7.84
Q8NI27	THO complex subunit 2 , GN=THOC2	2	4	2	1593		182.659	8.44
O75439	Mitochondrial-processing peptidase subunit beta , GN=PMPCB	2	3	2	489		54.331	6.83
Q9Y608	Leucine-rich repeat flightless-interacting protein 2 , GN=LRRFIP2	2	4	1	721		82.121	6.95
Q13033	Striatin-3 , GN=STRN3	3	4	3	797		87.155	5.36
Q8N573	Oxidation resistance protein 1 , GN=OXR1	1	4	1	874		97.909	5.47
O95340-2	phosphoadenosine 5'-phosphosulfate synthase 2 , GN=PAPSS2	1	4	1	619		69.926	8.03
A1KXE4	Myelin-associated neurite-outgrowth inhibitor , GN=FAM168B	2	4	2	195		20.311	9.23
Q15843	NEDD8 , GN=NEDD8	2	4	2	81		9.066	8.43
P49770	Translation initiation factor eIF-2B subunit beta , GN=EIF2B2	3	4	3	351		38.965	6.16
P78318	Immunoglobulin-binding protein 1 ,	2	4	2	339		39.198	5.38
Q16762	Thiosulfate sulfurtransferase , GN=TST	4	4	4	297		33.408	7.25
Q8NFC6	Biorientation of chromosomes in cell division protein 1-like 1 , GN=BOD1L1	2	4	2	3051		330.266	5.08
Q96B26	Exosome complex component RRP43 , GN=EXOSC8	1	3	1	276	30.02		5.3
Q8NFV4	Protein ABHD11 , GN=ABHD11	2	4	2	315		34.668	9.48
Q8N766	ER membrane protein complex subunit 1 , GN=EMC1	3	4	3	993		111.689	7.66
Q8N3F8	MICAL-like protein 1 , GN=MICALL1	1	4	1	863		93.383	7.25
Q15311	RalA-binding protein 1 , GN=RALBP1	2	3	2	655		76.016	5.88
O43504	Ragulator complex protein LAMTOR5 , GN=LAMTOR5	1	3	1	91		9.608	4.87
Q8NEB9	Phosphatidylinositol 3-kinase catalytic subunit type 3 , GN=PIK3C3	2	3	2	887		101.485	6.81
P48729	Casein kinase I isoform alpha , GN=CSNK1A1	2	4	2	337	38.89		9.57
Q9NSA3	Beta-catenin-interacting protein 1 , GN=CTNNBIP1	1	3	1	81		9.165	5.41
P83731	60S ribosomal protein L24 , GN=RPL24	1	4	1	157		17.768	11.25
Q5VWJ9	Sorting nexin-30 , GN=SNX30	2	3	2	437		49.645	5.35
Q8TEA8	D-aminoacyl-tRNA deacylase 1 , GN=DTD1	2	3	2	209		23.409	8.24
P57076	UPF0769 protein C21orf59 , GN=C21orf59	2	4	2	290		33.203	7.44
Q15785	Mitochondrial import receptor subunit TOM34 , GN=TOMM34	3	4	3	309		34.538	8.98
P48539	Calmodulin regulator protein PCP4 , GN=PCP4	2	3	2	62		6.787	6.71
Q9BWS9	Chitinase domain-containing protein 1 , GN=CHID1	2	4	2	393		44.912	8.63
Q9UJY4	ADP-ribosylation factor-binding protein GGA2 , GN=GGA2	2	3	2	613		67.108	6.55
Q9ULR0-1	Isoform 2 of Pre-mRNA-splicing factor ISY1 homolog , GN=ISY1	1	3	1	331		37.543	5.78
P50995	Annexin A11 , GN=ANXA11	2	4	2	505		54.355	7.65
Q00796	Sorbitol dehydrogenase , GN=SORD	1	4	1	357	38.3		7.97
Q13303-3	Isoform 3 of Voltage-gated potassium channel subunit beta-2 , GN=KCNAB2	1	3	1	415		46.497	8.72
Q13610	Periodic tryptophan protein 1 homolog , GN=PWP1	1	3	1	501		55.793	4.77
Q9NR46-2	Isoform 2 of Endophilin-B2 , GN=SH3GLB2	3	4	3	404		44.738	5.57
P07093-3	Isoform 3 of Glia-derived nexin ,	2	4	2	409		45.238	9.22
O00151	PDZ and LIM domain protein 1 , GN=PDLIM1	1	4	1	329		36.049	7.02
Q8WWH5	Probable tRNA pseudouridine synthase 1 , GN=TRUB1	2	4	2	349		37.229	8.25
P82673	28S ribosomal protein S35, mitochondrial , GN=MRPS35	3	4	3	323		36.821	8.24
P42677	40S ribosomal protein S27 , GN=RPS27	1	4	1	84		9.455	9.45
Q9NZL4-3	Isoform 3 of Hsp70-binding protein 1 , GN=HSPBP1	1	3	1	405		44.445	6.47
P42574	Caspase-3 , GN=CASP3	2	4	2	277		31.587	6.54
Q9UP83-2	Isoform 2 of Conserved oligomeric Golgi complex subunit 5 , GN=COG5	1	4	1	860		94.842	6.6
O75312	Zinc finger protein ZPR1 , GN=ZPR1	3	4	3	459		50.893	4.73

Q9H3S7	Tyrosine-protein phosphatase non-receptor type 23 , GN=PTPN23	2	3	2	1636		178.861	6.92	
Q9UKL0	REST corepressor 1 , GN=RCOR1	2	3	2	485		53.295	7.03	
Q15738	Sterol-4-alpha-carboxylate 3-dehydrogenase, decarboxylating , GN=NSDHL	2	4	2	373		41.874	8.06	
P19784	Casein kinase II subunit alpha' , GN=CSNK2A2	3	4	3	350		41.187	8.56	
O95394-4	Isoform 3 of Phosphoacetylglucosamine mutase , GN=PGM3	1	4	1	570		62.902		6
Q8N3X1-2	Isoform 2 of Formin-binding protein 4 , GN=FNBP4	1	4	1	1019		110.383	4.75	
Q96HR9	Receptor expression-enhancing protein 6 , GN=REEP6	2	4	2	211		23.403	8.56	
Q6UXN9	WD repeat-containing protein 82 ,	2	3	2	313		35.056	7.69	
Q7L266	Isoaspartyl peptidase/L-asparaginase , GN=ASRGL1	2	3	2	308		32.034	6.24	
P53365	Arfaptin-2 , GN=ARFIP2	2	4	2	341		37.832	6.04	
O00268	Transcription initiation factor TFIID subunit 4 , GN=TAF4	1	4	1	1085		110.047	9.94	
P61020	Ras-related protein Rab-5B , GN=RAB5B	1	4	1	215		23.692	8.13	
Q9P1Y6	PHD and RING finger domain-containing protein 1 , GN=PHRF1	1	3	1	1649		178.557	8.95	
Q9BQ95	intermediate in Toll pathway, mitochondrial , GN=ECSIT	1	4	1	431		49.117	6.29	
Q8IXJ6	NAD-dependent protein deacetylase sirtuin-2 , GN=SIRT2	2	4	2	389		43.154	5.36	
Q9NQH7	Probable Xaa-Pro aminopeptidase 3 , GN=XPNPEP3	1	3	1	507		56.997	6.83	
P13521	Secretogranin-2 , GN=SCG2	1	3	1	617		70.897	4.75	
P55268	Laminin subunit beta-2 , GN=LAMB2	3	4	3	1798		195.854	6.52	
Q99417	c-Myc-binding protein , GN=MYCBP	2	4	2	103		11.959	5.91	
Q9H773	dCTP pyrophosphatase 1 , GN=DCTPP1	3	4	3	170		18.669	5.03	
O94992	Protein HEXIM1 , GN=HEXIM1	2	3	2	359		40.598	4.89	
Q12800	Alpha-globin transcription factor CP2 , GN=TFCP2	2	3	2	502	57.22		5.8	
Q5JTZ9	Alanine--tRNA ligase, mitochondrial ,	2	3	2	985		107.273	6.27	
Q9BT09	Protein canopy homolog 3 , GN=CNPY3	1	3	1	278		30.729	5.49	
P20290-2	Isoform 2 of Transcription factor BTF3 ,	3	4	3	162		17.688	7.5	
Q96TA2	ATP-dependent zinc metalloprotease YME1L1 , GN=YME1L1	1	3	1	773		86.401	8.76	
Q9Y244	Proteasome maturation protein , GN=POMP	2	3	2	141		15.779	5.11	
O15116	U6 snRNA-associated Sm-like protein LSm1 , GN=LSM1	1	4	1	133	15.17		5.22	
Q6NZ67	Mitotic-spindle organizing protein 2B , GN=MZT2B	3	4	3	158		16.216	10.15	
Q8WWB5	PIH1 domain-containing protein 2 ,	1	4	1	315		35.934	6.37	
Q9H0E9-2	Isoform 2 of Bromodomain-containing protein 8 , GN=BRD8	2	3	2	951		102.775	4.69	
P07108-5	Isoform 5 of Acyl-CoA-binding protein ,	1	3	1	148		16.484	9.38	
Q9UIA9	Exportin-7 , GN=XPO7	1	3	1	1087		123.828	6.32	
P62993	Growth factor receptor-bound protein 2 , GN=GRB2	3	4	3	217	25.19		6.32	
Q13952	Nuclear transcription factor Y subunit gamma , GN=NFYC	1	4	1	458		50.271	6.1	
Q96EY8	Cob(I)yrinic acid a,c-diamide adenosyltransferase, mitochondrial , GN=MMAB	2	4	2	250		27.371	8.6	
O15084-1	Isoform 3 of Serine/threonine-protein phosphatase 6 regulatory ankyrin repeat subunit A , GN=ANKRD28	1	4	1	1086		116.469	6.18	
O60645	Exocyst complex component 3 , GN=EXOC3	1	2	1	756	86.79		6.11	
Q8ND76	Cyclin-Y , GN=CCNY	1	3	1	341		39.312	7.2	
P61081	NEDD8-conjugating enzyme Ubc12 ,	1	4	1	183		20.887	7.69	
Q9P2Y4	Zinc finger protein 219 , GN=ZNF219	1	3	1	722	76.83		9.39	
Q9NPQ8-3	Isoform 3 of Synembryn-A , GN=RIC8A	3	4	3	537		60.334	5.33	
P62888	60S ribosomal protein L30 , GN=RPL30	1	3	1	115		12.776	9.63	
Q9BXW6	Oxysterol-binding protein-related protein 1 , GN=OSBPL1A	2	4	2	950		108.401	6.38	

Q9BW30	Tubulin polymerization-promoting protein family member 3 , GN=TPPP3	1	4	1	176	18.974	9.13
P49589-3	Isoform 3 of Cysteine--tRNA ligase, cytoplasmic , GN=CARS	3	3	3	831	94.578	6.83
Q9BRX2	Protein pelota homolog , GN=PELO	1	3	1	385	43.332	6.34
P10636-9	Isoform Tau-G of Microtubule-associated protein tau , GN=MAPT	2	3	2	776	80.892	7.08
O14972	Down syndrome critical region protein 3 , GN=DSCR3 PE=2	1	3	1	297	32.989	7.68
P37235	Hippocalcin-like protein 1 , GN=HPCAL1	3	4	2	193	22.299	5.35
Q9UFN0	Protein NipSnap homolog 3A ,	2	4	2	247	28.449	9.16
Q96T58	Msx2-interacting protein , GN=SPEN	3	4	3	3664	402.004	7.64
O95197	Reticulon-3 , GN=RTN3	2	4	2	1032	112.541	4.96
Q9BQW3-1	Isoform 1 of Transcription factor COE4 ,	1	3	1	642	68.189	9.83
Q14168-4	Isoform 4 of MAGUK p55 subfamily member 2 , GN=MPP2	2	3	2	597	66.76	6.65
Q01167	Forkhead box protein K2 , GN=FOXK2	2	3	2	660	69.02	9.54
Q9HBM1	Kinetochore protein Spc25 , GN=SPC25	2	3	2	224	26.137	8
P08397	Porphobilinogen deaminase , GN=HMBS	1	3	1	361	39.306	7.18
O95400	CD2 antigen cytoplasmic tail-binding protein 2 , GN=CD2BP2	3	4	3	341	37.623	4.61
Q92930	Ras-related protein Rab-8B , GN=RAB8B	2	4	1	207	23.569	9.07
P46736	Lys-63-specific deubiquitinase BRCC36 , GN=BRCC3	2	3	2	316	36.049	5.92
O94826	Mitochondrial import receptor subunit TOM70 , GN=TOMM70	2	4	2	608	67.412	7.12
P82664	28S ribosomal protein S10, mitochondrial , GN=MRPS10	1	3	1	201	22.985	8
O15031	Plexin-B2 , GN=PLXNB2	2	3	2	1838	204.997	6.24
Q6ZN55-2	Isoform 2 of Zinc finger protein 574 ,	3	3	3	985	108.504	8.41
Q14139-2	Isoform 2 of Ubiquitin conjugation factor E4 A , GN=UBE4A	2	4	2	1073	123.442	5.24
P17677-2	Isoform 2 of Neuromodulin , GN=GAP43	2	4	2	274	28.749	4.87
Q15020	Squamous cell carcinoma antigen recognized by T-cells 3 , GN=SART3	3	3	3	963	109.865	5.57
Q9P0S9	Transmembrane protein 14C , GN=TMEM14C	1	4	1	112	11.557	9.88
P06454	Prothymosin alpha , GN=PTMA	2	4	2	111	12.196	3.78
P21953	2-oxoisovalerate dehydrogenase subunit beta, mitochondrial , GN=BCKDHB	1	3	1	392	43.095	6.29
Q3ZCQ8-2	Isoform 2 of Mitochondrial import inner membrane translocase subunit TIM50 , GN=TIMM50	1	3	1	456	50.433	9.42
Q5JTD0	Tight junction-associated protein 1 ,	1	4	1	557	61.783	5.97
Q14690	Protein RRP5 homolog , GN=PDCD11	1	3	1	1871	208.57	8.87
Q2M2I8	AP2-associated protein kinase 1 , GN=AAK1	2	3	2	961	103.821	6.6
P54252	Ataxin-3 , GN=ATXN3	2	4	2	364	41.754	4.91
Q5VSL9	Striatin-interacting protein 1 , GN=STRIP1	2	3	2	837	95.515	6.29
P40121	Macrophage-capping protein , GN=CAPG	1	3	1	348	38.474	6.19
Q14696	LRP chaperone MESD , GN=MESD	2	3	2	234	26.06	7.78
Q5JTJ3-2	Isoform 2 of Cytochrome c oxidase assembly factor 6 homolog , GN=COA6	1	3	1	156	18.082	10.1
O43617	Trafficking protein particle complex subunit 3 , GN=TRAPPC3	1	3	1	180	20.261	4.96
Q9BRZ2	E3 ubiquitin-protein ligase TRIM56 ,	1	3	1	755	81.437	7.74
P00352	Retinal dehydrogenase 1 , GN=ALDH1A1	2	3	2	501	54.827	6.73
P84090	Enhancer of rudimentary homolog , GN=ERH	2	4	2	104	12.251	5.92
Q9P0V9-2	Isoform 2 of Septin-10 , GN=SEPT10	1	4	1	503	58.099	7.34
Q13405	39S ribosomal protein L49, mitochondrial , GN=MRPL49	1	4	1	166	19.186	9.45
Q9P2B2	Prostaglandin F2 receptor negative regulator , GN=PTGFRN	1	3	1	879	98.495	6.61
Q96LB3	Intraflagellar transport protein 74 homolog , GN=IFT74	3	4	3	600	69.196	6
O43768-4	Isoform 4 of Alpha-endosulfine , GN=ENSA	1	4	1	140	15.524	8.72
Q9BYT8	Neurolysin, mitochondrial , GN=NLN	1	4	1	704	80.6	6.64
O14907	Tax1-binding protein 3 , GN=TAX1BP3	1	3	1	124	13.726	8.48
P00966	Argininosuccinate synthase , GN=ASS1	3	3	3	412	46.501	8.02

O60333-4	Isoform 4 of Kinesin-like protein KIF1B , GN=KIF1B	3	4	2	1823		205.012	5.52
P54646	5'-AMP-activated protein kinase catalytic subunit alpha-2 , GN=PRKAA2	3	3	3	552	62.28		7.75
Q9Y6K9-2	Isoform 2 of NF-kappa-B essential modulator , GN=IKBKG	1	2	1	487		55.751	6.7
P11117	Lysosomal acid phosphatase , GN=ACP2	1	4	1	423		48.313	6.74
Q5T0N5	Formin-binding protein 1-like , GN=FNBP1L	2	4	2	605		70.021	6.64
Q12907	Vesicular integral-membrane protein VIP36 , GN=LMAN2	2	3	2	356		40.203	6.95
Q15437	Protein transport protein Sec23B ,	2	3	1	767		86.424	6.89
Q6P1N9	Putative deoxyribonuclease TATDN1 , GN=TATDN1	3	4	3	297	33.58		6.96
Q8WX92	Negative elongation factor B , GN=NELFB	2	4	2	580		65.655	6.13
Q6PJG6	BRCA1-associated ATM activator 1 ,	1	3	1	821		88.063	5.27
Q5JU69	Torsin-2A , GN=TOR2A PE=2	1	4	1	321		35.691	7.97
Q7Z2T5	TRMT1-like protein , GN=TRMT1L	2	3	2	733		81.695	7.88
Q9BTA9	WW domain-containing adapter protein with coiled-coil , GN=WAC	1	3	1	647		70.681	9.45
Q9BXR0	Queuine tRNA-ribosyltransferase catalytic subunit 1 , GN=QTRT1	2	3	2	403		44.019	7.23
P31146	Coronin-1A , GN=CORO1A	2	3	2	461		50.994	6.68
O95396	Adenylyltransferase and sulfurtransferase MOCS3 , GN=MOCS3	1	3	1	460		49.638	6.21
Q9BRX5-3	Isoform 3 of DNA replication complex GINS protein PSF3 , GN=GINS3	1	3	1	255		28.756	5.24
Q9Y376	Calcium-binding protein 39 , GN=CAB39	2	4	2	341		39.844	6.89
P62318	Small nuclear ribonucleoprotein Sm D3 , GN=SNRPD3	1	4	1	126		13.907	10.32
P62266	40S ribosomal protein S23 , GN=RPS23	1	4	1	143		15.798	10.49
O95456	Proteasome assembly chaperone 1 ,	2	4	2	288		32.833	7.17
P19388	DNA-directed RNA polymerases I, II, and III subunit RPABC1 , GN=POLR2E	3	3	3	210		24.536	5.95
O00560	Syntenin-1 , GN=SDCBP	2	3	2	298		32.424	7.53
Q6XQN6	Nicotinate phosphoribosyltransferase , GN=NAPRT	3	3	3	538		57.542	5.68
O95749	Geranylgeranyl pyrophosphate synthase , GN=GGPS1	1	3	1	300		34.849	6.14
Q13107	Ubiquitin carboxyl-terminal hydrolase 4 , GN=USP4	2	3	2	963		108.496	5.71
Q15654	Thyroid receptor-interacting protein 6 , GN=TRIP6	1	3	1	476		50.255	7.37
Q9Y2Q9	28S ribosomal protein S28, mitochondrial , GN=MRPS28	2	4	2	187	20.83		9.1
Q99081-3	Isoform 3 of Transcription factor 12 ,	2	3	2	706		75.799	6.9
Q15773	Myeloid leukemia factor 2 , GN=MLF2	1	3	1	248		28.129	6.9
Q86U44	N6-adenosine-methyltransferase catalytic subunit , GN=METTL3	2	3	2	580		64.433	6.42
P62917	60S ribosomal protein L8 , GN=RPL8	2	3	2	257		28.007	11.03
O75940	Survival of motor neuron-related-splicing factor 30 , GN=SMNDC1	1	2	1	238		26.694	7.24
O60941	Dystrobrevin beta , GN=DTNB	3	3	3	627	71.31		7.91
Q8IZP0	Abl interactor 1 , GN=ABI1	2	3	2	508		55.047	7.06
O14617-5	Isoform 5 of AP-3 complex subunit delta-1 , GN=AP3D1	3	3	3	1215		136.565	7.64
Q00169	Phosphatidylinositol transfer protein alpha isoform , GN=PITPNA	1	3	1	270		31.786	6.55
P45954	Short/branched chain specific acyl-CoA dehydrogenase, mitochondrial , GN=ACADSB	3	3	3	432		47.455	6.99
P50336	Protoporphyrinogen oxidase , GN=PPOX	2	3	2	477		50.734	8.16
Q9P0J1-2	Isoform 2 of [Pyruvate dehydrogenase [acetyl-transferring]]-phosphatase 1, mitochondrial , GN=PDP1	2	3	2	562		63.655	6.81
Q13232	Nucleoside diphosphate kinase 3 , GN=NME3	1	3	1	169		19.003	7.84
P14543	Nidogen-1 , GN=NID1	1	3	1	1247		136.291	5.29
Q15642-3	Isoform 3 of Cdc42-interacting protein 4 , GN=TRIP10	3	3	3	593		67.561	6.25

Q9Y653	Adhesion G-protein coupled receptor G1 , GN=ADGRG1	1	3	1	693		77.688	8.48
Q9UHR5	SAP30-binding protein , GN=SAP30BP	2	3	2	308	33.85		4.84
P51649-2	dehydrogenase, mitochondrial , GN=ALDH5A1	2	3	2	548		58.616	8.09
Q15024	Exosome complex component RRP42 , GN=EXOSC7	1	3	1	291		31.801	5.19
Q969Q5	Ras-related protein Rab-24 , GN=RAB24	1	3	1	203		23.109	6.23
P35612	Beta-adducin , GN=ADD2	3	3	3	726		80.803	5.92
Q99757	Thioredoxin, mitochondrial , GN=TXN2	2	3	2	166		18.372	8.29
Q9BSH5	Haloacid dehalogenase-like hydrolase domain-containing protein 3 , GN=HDHD3	2	3	2	251		27.982	6.71
P42694	Probable helicase with zinc finger domain , GN=HELZ	1	3	1	1942		218.833	7.42
P54709	Sodium/potassium-transporting ATPase subunit beta-3 , GN=ATP1B3	1	3	1	279		31.492	8.35
P03952	Plasma kallikrein , GN=KLKB1	1	3	1	638		71.323	8.22
Q9H993	Protein-glutamate O-methyltransferase , GN=ARMT1	3	3	3	441	51.14		5.76
Q9Y5X1	Sorting nexin-9 , GN=SNX9	2	3	2	595	66.55		5.58
Q15418-2	Isoform 2 of Ribosomal protein S6 kinase alpha-1 , GN=RPS6KA1	2	3	1	744	83.88		8.78
Q3SXM5	Inactive hydroxysteroid dehydrogenase-like protein 1 , GN=HSDL1	1	3	1	330		36.978	8.72
Q8N2F6	Armadillo repeat-containing protein 10 , GN=ARMC10	1	3	1	343		37.517	6.61
Q9BX40	Protein LSM14 homolog B , GN=LSM14B	1	2	1	385		42.045	9.69
P61962	DDB1- and CUL4-associated factor 7 ,	2	3	2	342		38.901	5.52
O60488	Long-chain-fatty-acid--CoA ligase 4 ,	2	3	2	711		79.137	8.38
O60568	Procollagen-lysine,2-oxoglutarate 5-dioxygenase 3 , GN=PLOD3	2	3	2	738		84.731	6.05
Q9UJA5	tRNA (adenine(58)-N(1))-methyltransferase non-catalytic subunit TRM6 , GN=TRMT6	2	3	2	497		55.764	7.55
Q9H446	RWD domain-containing protein 1 ,	1	3	1	243		27.922	4.2
P48739-3	Isoform 3 of Phosphatidylinositol transfer protein beta isoform , GN=PITPNB	3	3	3	273	31.68		6.65
Q8TBC4	NEDD8-activating enzyme E1 catalytic subunit , GN=UBA3	3	3	3	463		51.819	5.45
Q6IMI6	Sulfotransferase 1C3 , GN=SULT1C3	2	3	2	304		35.865	6.92
Q9UQR1	Zinc finger protein 148 , GN=ZNF148	2	3	2	794		88.921	6.48
Q96JB2	Conserved oligomeric Golgi complex subunit 3 , GN=COG3	1	3	1	828		94.036	5.57
Q14254	Flotillin-2 , GN=FLOT2	2	3	2	428		47.035	5.25
Q6VMQ6-4	Isoform 3 of Activating transcription factor 7-interacting protein 1 , GN=ATF7IP	2	3	2	1278		137.409	4.72
Q9P2N2	Rho GTPase-activating protein 28 , GN=ARHGAP28	1	3	1	729		82.008	7.75
Q9UGU0	Transcription factor 20 , GN=TCF20	2	3	2	1960		211.641	9.04
O43837	Isocitrate dehydrogenase [NAD] subunit beta, mitochondrial , GN=IDH3B	2	3	2	385		42.157	8.46
P26358-2	Isoform 2 of DNA (cytosine-5)-methyltransferase 1 , GN=DNMT1	2	3	2	1632		184.703	7.85
Q6YP21	Kynurenine--oxoglutarate transaminase 3 , GN=KYAT3	1	2	1	454		51.368	8.19
Q70UQ0-4	Isoform 4 of Inhibitor of nuclear factor kappa-B kinase-interacting protein , GN=IKBIP	2	3	2	377		43.057	4.92
Q08AM6	Protein VAC14 homolog , GN=VAC14	1	3	1	782		87.917	6.13
Q9BQ67	Glutamate-rich WD repeat-containing protein 1 , GN=GRWD1	1	3	1	446		49.388	4.92
Q8NFH3	Nucleoporin Nup43 , GN=NUP43	2	3	2	380		42.124	5.63
O95168	NADH dehydrogenase [ubiquinone] 1 beta subcomplex subunit 4 , GN=NDUFB4	1	3	1	129		15.199	9.85
Q09161	Nuclear cap-binding protein subunit 1 , GN=NCBP1	3	3	3	790		91.781	6.43
Q14515	SPARC-like protein 1 , GN=SPARCL1	1	3	1	664		75.161	4.81
Q8WXC6	COP9 signalosome complex subunit 9 , GN=COPS9	1	2	1	57		6.207	3.83

O75569	Interferon-inducible double-stranded RNA-dependent protein kinase activator A , GN=PRKRA	1	3	1	313	34.383	8.41
Q9H2D6	TRIO and F-actin-binding protein ,	2	3	2	2365	261.217	8.48
P17535	Transcription factor jun-D , GN=JUND	2	3	2	347	35.152	7.37
Q9NZD2	Glycolipid transfer protein , GN=GLTP	1	3	1	209	23.834	7.39
O15488	Glycogenin-2 , GN=GYG2	1	2	1	501	55.149	5.1
O75976	Carboxypeptidase D , GN=CPD	3	3	3	1380	152.835	6.05
P32321-2	Isoform 2 of Deoxycytidylate deaminase , GN=DCTD	2	3	2	189	21	7.53
Q92620	Pre-mRNA-splicing factor ATP-dependent RNA helicase PRP16 , GN=DHX38	1	3	1	1227	140.415	6.54
Q9NY61	Protein AATF , GN=AATF	2	3	2	560	63.094	4.94
P14209	CD99 antigen , GN=CD99	2	3	2	185	18.836	4.75
Q14966	Zinc finger protein 638 , GN=ZNF638	3	3	3	1978	220.488	6.38
Q8TB22-2	Isoform 2 of Spermatogenesis-associated protein 20 , GN=SPATA20	1	3	1	802	89.743	7.81
P50479	PDZ and LIM domain protein 4 , GN=PDLIM4	3	3	3	330	35.376	7.91
Q685J3	Mucin-17 , GN=MUC17	1	3	1	4493	451.465	4.11
Q9HCN8	Stromal cell-derived factor 2-like protein 1 , GN=SDF2L1	1	3	1	221	23.584	7.03
Q7L1Q6-3	Isoform 3 of Basic leucine zipper and W2 domain-containing protein 1 , GN=BZW1	2	3	2	451	51.249	6.62
Q9Y5L4	Mitochondrial import inner membrane translocase subunit Tim13 , GN=TIMM13	2	3	2	95	10.493	8.18
Q86UL8	Membrane-associated guanylate kinase, WW and PDZ domain-containing protein 2 , GN=MAGI2	2	3	2	1455	158.656	6.35
P45985-2	activated protein kinase kinase 4 , GN=MAP2K4	2	3	2	410	45.555	8.03
Q9P107	GEM-interacting protein , GN=GMIP	2	3	2	970	106.617	5.72
Q9NUQ3	Gamma-taxilin , GN=TXLNG	2	3	2	528	60.548	7.52
P63000-2	Isoform B of Ras-related C3 botulinum toxin substrate 1 , GN=RAC1	2	3	2	211	23.452	8.63
Q8NEZ2	Vacuolar protein sorting-associated protein 37A , GN=VPS37A	3	3	3	397	44.287	5.57
Q9Y6D6	Brefeldin A-inhibited guanine nucleotide-exchange protein 1 , GN=ARFGEF1	2	3	2	1849	208.634	5.85
Q6P1N0	Coiled-coil and C2 domain-containing protein 1A , GN=CC2D1A	1	3	1	951	103.998	8.09
Q15006	ER membrane protein complex subunit 2 , GN=EMC2	1	3	1	297	34.811	6.57
O95816	BAG family molecular chaperone regulator 2 , GN=BAG2	2	3	2	211	23.757	6.7
Q9H299	SH3 domain-binding glutamic acid-rich-like protein 3 , GN=SH3BGRL3	1	3	1	93	10.431	4.93
Q14258	E3 ubiquitin/ISG15 ligase TRIM25 ,	3	3	3	630	70.928	8.09
Q9H7P6	Multivesicular body subunit 12B ,	3	3	3	319	35.597	8.15
Q9Y237-2	Isoform 2 of Peptidyl-prolyl cis-trans isomerase NIMA-interacting 4 , GN=PIN4	3	3	3	156	16.598	9.96
Q96CW5	Gamma-tubulin complex component 3 , GN=TUBGCP3	2	3	2	907	103.506	8.12
Q4V339	COBW domain-containing protein 6 , GN=CBWD6 PE=3	2	3	2	395	43.937	4.87
Q13464	Rho-associated protein kinase 1 , GN=ROCK1	3	3	3	1354	158.076	5.9
Q9NWS0	PIH1 domain-containing protein 1 ,	1	3	1	290	32.342	5.14
P42858	Huntingtin , GN=HTT	1	2	1	3142	347.383	6.2
P49821	NADH dehydrogenase [ubiquinone] flavoprotein 1, mitochondrial , GN=NDUFV1	1	3	1	464	50.785	8.21
Q8NBN7	Retinol dehydrogenase 13 , GN=RDH13	2	3	2	331	35.91	8.1
P09234	U1 small nuclear ribonucleoprotein C , GN=SNRPC	1	3	1	159	17.381	9.67
Q8WUD4	Coiled-coil domain-containing protein 12 , GN=CCDC12	1	2	1	166	19.169	7.34
Q9ULL5-3	Isoform 3 of Proline-rich protein 12 ,	1	2	1	2036	210.914	7.87
Q8N108-12	Isoform 2 of Mesoderm induction early response protein 1 , GN=MIER1	2	3	2	565	63.387	4.46

Q9NUP1	Biogenesis of lysosome-related organelles complex 1 subunit 4 , GN=BLOC1S4	1	3	1	217	23.336	4.98
Q9BXK5	Bcl-2-like protein 13 , GN=BCL2L13	3	3	3	485	52.691	4.44
Q9H204	Mediator of RNA polymerase II transcription subunit 28 , GN=MED28	1	2	1	178	19.508	5.58
Q96RF0	Sorting nexin-18 , GN=SNX18	1	3	1	628	68.851	5.68
Q15021	Condensin complex subunit 1 , GN=NCAPD2	3	3	3	1401	157.082	6.61
Q5VTR2	E3 ubiquitin-protein ligase BRE1A ,	3	3	3	975	113.592	5.94
Q9Y2H1	Serine/threonine-protein kinase 38-like , GN=STK38L	2	3	2	464	53.968	6.81
Q8IWY9	Codanin-1 , GN=CDAN1	1	3	1	1227	134.035	6.77
P56181-2	[ubiquinone] flavoprotein 3, mitochondrial , GN=NDUFV3	3	3	3	473	50.953	9.17
Q8NF37	Lysophosphatidylcholine acyltransferase 1 , GN=LPCAT1	1	3	1	534	59.113	6.02
P27105	Erythrocyte band 7 integral membrane protein , GN=STOM	1	3	1	288	31.711	7.88
Q13163	Dual specificity mitogen-activated protein kinase kinase 5 , GN=MAP2K5	1	3	1	448	50.08	6.39
P33121	Long-chain-fatty-acid--CoA ligase 1 ,	1	3	1	698	77.893	7.15
P07148	Fatty acid-binding protein, liver , GN=FABP1	2	3	2	127	14.199	7.18
O95292	Vesicle-associated membrane protein-associated protein B/C , GN=VAPB	2	3	2	243	27.211	7.3
Q8N0Z6	Tetratricopeptide repeat protein 5 , GN=TTC5	2	3	2	440	48.897	6.48
Q96RP9-2	Isoform 2 of Elongation factor G, mitochondrial , GN=GFM1	2	3	2	770	85.815	7.18
Q15054	DNA polymerase delta subunit 3 , GN=POLD3	2	3	2	466	51.368	9.35
Q15633	RISC-loading complex subunit TARBP2 , GN=TARBP2	3	3	3	366	39.015	6.54
P06756	Integrin alpha-V , GN=ITGAV	2	3	2	1048	115.964	5.68
Q8TB72	Pumilio homolog 2 , GN=PUM2	2	3	1	1066	114.145	7.08
A6ZKI3	Retrotransposon Gag-like protein 8B ,	1	3	1	113	13.163	5.07
Q96S55	ATPase WRNIP1 , GN=WRNIP1	2	3	2	665	72.088	6.1
O00161	Synaptosomal-associated protein 23 , GN=SNAP23	1	2	1	211	23.34	5.01
O15212	Prefoldin subunit 6 , GN=PFDN6	1	3	1	129	14.574	8.88
Q8IUH3	RNA-binding protein 45 , GN=RBM45	1	3	1	476	53.469	7.17
Q9BPX5	Actin-related protein 2/3 complex subunit 5-like protein , GN=ARPC5L	1	3	1	153	16.931	6.6
Q53F19	Nuclear cap-binding protein subunit 3 , GN=NCBP3	2	3	2	620	70.549	5.73
Q9BXV9	EKC/KEOPS complex subunit GON7 ,	1	3	1	100	10.852	4.27
Q96IZ0	PRKC apoptosis WT1 regulator protein , GN=PAWR	2	3	2	340	36.545	5.41
O95571	Persulfide dioxygenase ETHE1, mitochondrial , GN=ETHE1	1	2	1	254	27.855	6.83
Q15768	Ephrin-B3 , GN=EFNB3	3	3	3	340	35.812	8.6
Q96IF1	LIM domain-containing protein ajuba , GN=AJUBA	2	3	2	538	56.897	7.14
Q99816	Tumor susceptibility gene 101 protein , GN=TSG101	2	3	2	390	43.916	6.46
Q86TP1	Exopolyphosphatase PRUNE1 , GN=PRUNE1	3	3	3	453	50.168	5.5
P18031	Tyrosine-protein phosphatase non-receptor type 1 , GN=PTPN1	2	3	2	435	49.935	6.27
Q58HT7	Genome polyprotein OS=Dengue virus type 4 (strain Philippines/H241/1956)	3	3	3	3387	378.164	8.43
Q9NUP9	Protein lin-7 homolog C , GN=LIN7C	1	3	1	197	21.82	8.43
Q658Y4	Protein FAM91A1 , GN=FAM91A1	3	3	3	838	93.85	6.39
Q9Y6R4	Mitogen-activated protein kinase kinase kinase 4 , GN=MAP3K4	1	3	1	1608	181.57	6.32
P36551	Oxygen-dependent coproporphyrinogen-III oxidase, mitochondrial , GN=CPOX	2	3	2	454	50.12	8.25
Q9ULP0-6	Isoform 6 of Protein NDRG4 , GN=NDRG4	1	2	1	391	42.859	5.74
Q9BRT9	DNA replication complex GINS protein SLD5 , GN=GINS4	2	3	2	223	26.03	4.98
P02794	Ferritin heavy chain , GN=FTH1	2	3	2	183	21.212	5.55

Q96EY7	Pentatricopeptide repeat domain-containing protein 3, mitochondrial , GN=PTCD3	3	3	3	689	78.5	6.42
P62072	Mitochondrial import inner membrane translocase subunit Tim10 , GN=TIMM10	1	2	1	90	10.326	6.29
Q9NQ29	Putative RNA-binding protein Luc7-like 1 , GN=LUC7L	3	3	2	371	43.701	9.92
O94811	Tubulin polymerization-promoting protein , GN=TPPP	2	2	2	219	23.679	9.44
Q8ND56	Protein LSM14 homolog A , GN=LSM14A	2	3	2	463	50.499	9.52
O94819	Kelch repeat and BTB domain-containing protein 11 , GN=KBTBD11	3	3	3	623	65.679	6.07
Q15506	Sperm surface protein Sp17 , GN=SPA17	2	3	2	151	17.396	4.78
O00186	Syntaxin-binding protein 3 , GN=STXBP3	2	3	2	592	67.721	7.8
Q9NPJ3	Acyl-coenzyme A thioesterase 13 ,	1	3	1	140	14.951	9.14
Q8IYQ7	Threonine synthase-like 1 , GN=THNSL1	2	3	2	743	83.017	7.12
P61966	AP-1 complex subunit sigma-1A , GN=AP1S1	1	2	1	158	18.721	5.73
O15525	Transcription factor MafG , GN=MAFG	1	3	1	162	17.839	10.04
Q12756-2	Isoform 2 of Kinesin-like protein KIF1A , GN=KIF1A	2	3	1	1799	202.774	5.54
Q9BQP7	Mitochondrial genome maintenance exonuclease 1 , GN=MGME1	1	3	1	344	39.396	7.68
Q8N6R0	Methyltransferase-like protein 13 , GN=METTL13	1	2	1	699	78.718	6.73
P17480	Nucleolar transcription factor 1 , GN=UBTF	2	3	2	764	89.35	5.81
Q7L8J4	SH3 domain-binding protein 5-like , GN=SH3BP5L	1	2	1	393	43.473	5.77
O00625	Pirin , GN=PIR	1	3	1	290	32.093	6.92
O60739	Eukaryotic translation initiation factor 1b , GN=EIF1B	2	3	2	113	12.816	7.37
Q9UBL3	Set1/Ash2 histone methyltransferase complex subunit ASH2 , GN=ASH2L	1	3	1	628	68.679	5.69
P23511	Nuclear transcription factor Y subunit alpha , GN=NFYA	1	2	1	347	36.854	8.94
Q15036	Sorting nexin-17 , GN=SNX17	2	3	2	470	52.868	7.46
P39059	Collagen alpha-1(XV) chain , GN=COL15A1	2	3	2	1388	141.632	5
P49366	Deoxyhypusine synthase , GN=DHPS	2	3	2	369	40.945	5.36
Q0VDF9	Heat shock 70 kDa protein 14 , GN=HSPA14	1	2	1	509	54.76	5.59
O00762	Ubiquitin-conjugating enzyme E2 C ,	2	3	2	179	19.64	7.37
Q96JM3	Chromosome alignment-maintaining phosphoprotein 1 , GN=CHAMP1	3	3	3	812	89.043	8.44
Q96JQ2	Calmin , GN=CLMN	1	2	1	1002	111.582	4.94
O14757	Serine/threonine-protein kinase Chk1 , GN=CHEK1	2	3	2	476	54.399	8.25
Q9BQ61	Telomerase RNA component interacting RNase , GN=TRIR	2	3	2	176	18.408	9.44
Q96FV2	Secernin-2 , GN=SCRN2	3	3	3	425	46.567	5.67
Q9ULT8	E3 ubiquitin-protein ligase HECTD1 ,	2	3	2	2610	289.203	5.35
P52435	DNA-directed RNA polymerase II subunit RPB11-a , GN=POLR2J	2	3	2	117	13.285	5.86
Q9H496	Torsin-1A-interacting protein 2, isoform IFRG15 , GN=TOR1AIP2	1	3	1	131	15.338	7.31
Q8IWA4	Mitofusin-1 , GN=MFN1	1	3	1	741	84.047	6.25
P14406	Cytochrome c oxidase subunit 7A2, mitochondrial , GN=COX7A2	1	3	1	83	9.39	9.76
Q96SL4	Glutathione peroxidase 7 , GN=GPX7	1	3	1	187	20.983	8.27
Q8WUA4	General transcription factor 3C polypeptide 2 , GN=GTF3C2	1	2	1	911	100.616	7.31
Q92572	AP-3 complex subunit sigma-1 , GN=AP3S1	2	3	2	193	21.718	5.39
P06730-2	Isoform 2 of Eukaryotic translation initiation factor 4E , GN=EIF4E	1	3	1	248	28.76	6.42
P17252	Protein kinase C alpha type , GN=PRKCA	2	3	2	672	76.7	7.05
P00167	Cytochrome b5 , GN=CYB5A	1	3	1	134	15.321	4.96
Q8TF01	Arginine/serine-rich protein PNISR ,	1	3	1	805	92.521	10.02
Q86VW0	SEC14 domain and spectrin repeat-containing protein 1 , GN=SESTD1	1	2	1	696	79.298	5.1
Q96MW1	Coiled-coil domain-containing protein 43 , GN=CCDC43	2	3	2	224	25.233	4.92

Q7Z406-2	Isoform 2 of Myosin-14 , GN=MYH14	3	3	1	2036	231.87	5.6
Q9NRG9	Aladin , GN=AAAS	3	3	3	546	59.536	7.5
Q9UJ04	Testis-specific Y-encoded-like protein 4 , GN=TSPYL4	3	3	3	414	45.098	8.57
P22033	Methylmalonyl-CoA mutase, mitochondrial , GN=MUT	2	3	2	750	83.082	6.93
Q00536-2	Isoform 2 of Cyclin-dependent kinase 16 , GN=CDK16	3	3	3	570	63.418	8.13
P10589	COUP transcription factor 1 , GN=NR2F1	1	3	1	423	46.126	8.25
Q8IWE2	Protein NOXP20 , GN=FAM114A1	2	2	2	563	60.704	4.68
Q9BWJ5	Splicing factor 3B subunit 5 , GN=SF3B5	3	3	3	86	10.129	6.35
Q96CB8	Integrator complex subunit 12 , GN=INTS12	1	3	1	462	48.777	9.69
Q9H9J2	39S ribosomal protein L44, mitochondrial , GN=MRPL44	1	2	1	332	37.512	8.4
Q6KC79	Nipped-B-like protein , GN=NIPBL	1	2	1	2804	315.854	7.91
Q96S59	Ran-binding protein 9 , GN=RANBP9	1	3	1	729	77.798	6.79
Q9BRK5	45 kDa calcium-binding protein , GN=SDF4	2	2	2	362	41.78	4.86
Q13418	Integrin-linked protein kinase , GN=ILK	2	3	2	452	51.386	8.07
P31350-2	Isoform 2 of Ribonucleoside-diphosphate reductase subunit M2 , GN=RRM2	2	3	1	449	51.06	6.57
O75928	E3 SUMO-protein ligase PIAS2 , GN=PIAS2	3	3	2	621	68.197	7.52
Q7Z3D6-2	Isoform 2 of D-glutamate cyclase, mitochondrial , GN=DGLUCY	2	3	2	621	66.884	6.87
Q8IXQ4	GPALPP motifs-containing protein 1 , GN=GPALPP1	1	3	1	340	38.119	5.41
Q9NV56	MRG/MORF4L-binding protein , GN=MRGBP	2	3	2	204	22.403	5.83
Q8WZ42-12	Isoform 12 of Titin , GN=TTN	2	3	2	35991	3.992.135	6.39
O76071	Probable cytosolic iron-sulfur protein assembly protein CIAO1 , GN=CIAO1	1	2	1	339	37.816	4.97
Q9H974-4	Isoform 4 of Queuine tRNA-ribosyltransferase accessory subunit 2 , GN=QTRT2	1	2	1	427	48.172	6.61
Q0VDG4	Secernin-3 , GN=SCRN3	1	2	1	424	48.513	5.55
Q13546	Receptor-interacting serine/threonine-protein kinase 1 , GN=RIPK1	1	2	1	671	75.883	6.33
Q6NTF9-2	Isoform 2 of Rhomboid domain-containing protein 2 , GN=RHBDD2	1	3	1	409	43.77	9.22
Q86Y37	CDK2-associated and cullin domain-containing protein 1 , GN=CACUL1	1	2	1	369	41.037	5.19
Q24JP5-2	Isoform 2 of Transmembrane protein 132A , GN=TMEM132A	1	2	1	1024	110.128	5.62
P08243	Asparagine synthetase [glutamine-hydrolyzing] , GN=ASNS	3	3	3	561	64.329	6.86
O00567	Nucleolar protein 56 , GN=NOP56	1	2	1	594	66.009	9.19
Q99615	DnaJ homolog subfamily C member 7 , GN=DNAJC7	2	3	2	494	56.405	6.96
Q96A35	39S ribosomal protein L24, mitochondrial , GN=MRPL24	1	3	1	216	24.899	9.29
Q9NZT2	Opioid growth factor receptor , GN=OGFR	1	2	1	677	73.28	4.84
Q9P265	Disco-interacting protein 2 homolog B , GN=DIP2B	2	2	2	1576	171.382	8.09
Q9BW91	ADP-ribose pyrophosphatase, mitochondrial , GN=NUDT9	1	3	1	350	39.101	8.22
P78560	Death domain-containing protein CRADD , GN=CRADD	2	3	2	199	22.731	6.8
Q96T88-2	Isoform 2 of E3 ubiquitin-protein ligase UHRF1 , GN=UHRF1	3	3	3	806	91.059	7.44
Q9UBI6	G(I)/G(S)/G(O) subunit gamma-12 , GN=GNG12	2	3	2	72	8.001	8.97
Q9Y315	Deoxyribose-phosphate aldolase , GN=DERA	2	3	2	318	35.208	8.94
Q8TB52	F-box only protein 30 , GN=FBXO30	1	3	1	745	82.251	5.4
Q9Y2I8-2	Isoform 2 of WD repeat-containing protein 37 , GN=WDR37	1	2	1	495	54.76	7.23
P61026	Ras-related protein Rab-10 , GN=RAB10	2	3	1	200	22.527	8.38
Q8NEU8	DCC-interacting protein 13-beta , GN=APPL2	2	2	2	664	74.446	4.94
Q9H425	Uncharacterized protein C1orf198 , GN=C1orf198	1	3	1	327	36.324	5.72

Q9NX24	H/ACA ribonucleoprotein complex subunit 2 , GN=NHP2	1	2	1	153	17.19		8.22
Q5TC12	ATP synthase mitochondrial F1 complex assembly factor 1 , GN=ATPAF1	1	2	1	328		36.414	7.96
Q7L9L4-2	Isoform 2 of MOB kinase activator 1B , GN=MOB1B	1	3	1	221		25.483	6.15
Q68CZ6	HAUS augmin-like complex subunit 3 , GN=HAUS3	2	2	2	603		69.607	5.55
Q9H267	Vacuolar protein sorting-associated protein 33B , GN=VPS33B	1	2	1	617	70.54		6.71
Q709C8	Vacuolar protein sorting-associated protein 13C , GN=VPS13C	3	3	3	3753		422.124	6.83
Q9UJX2	Cell division cycle protein 23 homolog , GN=CDC23	1	2	1	597	68.79		7.02
Q96RL7	Vacuolar protein sorting-associated protein 13A , GN=VPS13A	2	2	2	3174		360.047	6.33
Q9UJY5-6	Isoform 6 of ADP-ribosylation factor-binding protein GGA1 , GN=GGA1	1	2	1	656		72.232	5.35
Q86VM9	Zinc finger CCCH domain-containing protein 18 , GN=ZC3H18	3	3	3	953		106.315	8.32
Q6PL18	ATPase family AAA domain-containing protein 2 , GN=ATAD2	1	3	1	1390		158.456	6.32
P47914	60S ribosomal protein L29 , GN=RPL29	1	3	1	159		17.741	11.66
Q92896-2	Isoform 2 of Golgi apparatus protein 1 , GN=GLG1	2	3	2	1203		137.132	6.9
Q92600-2	Isoform 2 of CCR4-NOT transcription complex subunit 9 , GN=CNOT9	1	2	1	331		36.813	7.03
O95352	Ubiquitin-like modifier-activating enzyme ATG7 , GN=ATG7	2	3	2	703		77.909	6.24
Q9NRW7	Vacuolar protein sorting-associated protein 45 , GN=VPS45	1	3	1	570		65.036	8.24
Q9Y259	Choline/ethanolamine kinase , GN=CHKB	1	3	1	395		45.243	5.49
Q8N2U0	Transmembrane protein 256 , GN=TMEM256 PE=3	1	2	1	113		11.734	8.94
O75534-4	Isoform 4 of Cold shock domain-containing protein E1 , GN=CSDE1	2	3	2	844		93.683	6.52
P78537	Biogenesis of lysosome-related organelles complex 1 subunit 1 , GN=BLOC1S1	1	3	1	153		17.252	9.33
Q9BRF8	Serine/threonine-protein phosphatase CPPED1 , GN=CPPED1	1	3	1	314		35.526	6.2
P11441	Ubiquitin-like protein 4A , GN=UBL4A	1	3	1	157		17.766	8.66
O94842	TOX high mobility group box family member 4 , GN=TOX4	1	3	1	621		66.153	5.06
Q8IXH7	Negative elongation factor C/D , GN=NELFCD	2	3	2	590		66.204	5.1
P35914	Hydroxymethylglutaryl-CoA lyase, mitochondrial , GN=HMGCL	1	3	1	325		34.338	8.54
P07738	Bisphosphoglycerate mutase , GN=BPGM	1	3	1	259		29.987	6.54
P19387	DNA-directed RNA polymerase II subunit RPB3 , GN=POLR2C	1	3	1	275		31.422	4.92
O43813	LanC-like protein 1 , GN=LANCL1	1	3	1	399		45.254	7.75
Q9NVJ2	ADP-ribosylation factor-like protein 8B , GN=ARL8B	2	2	2	186		21.525	8.43
Q9GZT8	NIF3-like protein 1 , GN=NIF3L1	2	3	2	377		41.942	6.65
P53779	Mitogen-activated protein kinase 10 , GN=MAPK10	1	3	1	464		52.551	6.79
Q9BUR5	MICOS complex subunit MIC26 , GN=APOO	1	3	1	198		22.271	9.13
Q92522	Histone H1x , GN=H1FX	1	3	1	213		22.474	10.76
Q9UGL1-2	Isoform 2 of Lysine-specific demethylase 5B , GN=KDM5B	2	3	2	1580		179.294	6.76
Q9ULH7-5	Isoform 5 of MKL/myocardin-like protein 2 , GN=MKL2	1	2	1	1099		119.092	6.09
A4GCI8	Matrix protein 1 OS=Influenza A virus (strain A/Henry/1936 H1N1) GN=M PE=3	1	3	1	252		27.847	9.36
P09497	Clathrin light chain B , GN=CLTB	1	3	1	229		25.175	4.64
Q9H501	ESF1 homolog , GN=ESF1	3	3	3	851		98.735	5.11
Q9P0L0-2	Isoform 2 of Vesicle-associated membrane protein-associated protein A , GN=VAPA	2	3	2	294		32.593	8.91

Q9HC35	Echinoderm microtubule-associated protein-like 4 , GN=EML4	3	3	3	981	108.848	6.4
P36954	DNA-directed RNA polymerase II subunit RPB9 , GN=POLR2I	1	2	1	125	14.514	5.14
O95684	FGFR1 oncogene partner , GN=FGFR1OP	2	2	2	399	43.039	4.81
Q8NBS9	Thioredoxin domain-containing protein 5 , GN=TXNDC5	1	2	1	432	47.599	5.97
P10606	Cytochrome c oxidase subunit 5B, mitochondrial , GN=COX5B	1	2	1	129	13.687	8.81
Q8IX18	Probable ATP-dependent RNA helicase DHX40 , GN=DHX40	1	3	1	779	88.504	8.65
Q9BSL1	Ubiquitin-associated domain-containing protein 1 , GN=UBAC1	2	2	2	405	45.31	4.92
Q9Y3A6	Transmembrane emp24 domain-containing protein 5 , GN=TMED5	1	2	1	229	25.988	4.84
Q9Y312	Protein AAR2 homolog , GN=AAR2	2	2	2	384	43.444	5.96
Q8N806	Putative E3 ubiquitin-protein ligase UBR7 , GN=UBR7	2	2	2	425	47.968	4.81
O75165	DnaJ homolog subfamily C member 13 , GN=DNAJC13	2	2	2	2243	254.252	6.74
Q9UMY4	Sorting nexin-12 , GN=SNX12	2	2	2	172	19.718	7.87
Q5VW32	BRO1 domain-containing protein BROX , GN=BROX	2	2	2	411	46.447	7.65
Q9BTE1	Dynactin subunit 5 , GN=DCTN5	1	3	1	182	20.113	8.02
Q53GQ0	Very-long-chain 3-oxoacyl-CoA reductase , GN=HSD17B12	1	3	1	312	34.302	9.32
Q9NV96	Cell cycle control protein 50A , GN=TMEM30A	1	2	1	361	40.658	8.59
Q719H9	BTB/POZ domain-containing protein KCTD1 , GN=KCTD1	2	3	2	257	29.386	7.08
O00746	Nucleoside diphosphate kinase, mitochondrial , GN=NME4	2	3	2	187	20.646	10.29
Q86SX6	Glutaredoxin-related protein 5, mitochondrial , GN=GLRX5	1	2	1	157	16.618	6.79
O75909-4	Isoform 4 of Cyclin-K , GN=CCNK	1	2	1	600	66.095	8.63
Q9UKX3	Myosin-13 , GN=MYH13 PE=2	1	3	1	1938	223.465	5.68
Q5KU26	Collectin-12 , GN=COLEC12	1	2	1	742	81.465	5.69
P10619	Lysosomal protective protein , GN=CTSA	1	2	1	480	54.431	6.61
Q2M389	WASH complex subunit 4 , GN=WASHC4	2	3	2	1173	136.316	7.44
O43395	U4/U6 small nuclear ribonucleoprotein Prp3 , GN=PRPF3	2	2	2	683	77.481	9.5
P56937	3-keto-steroid reductase , GN=HSD17B7	1	2	1	341	38.182	8.1
Q96GG9	DCN1-like protein 1 , GN=DCUN1D1	1	2	1	259	30.105	5.34
Q99848	Probable rRNA-processing protein EBP2 , GN=EBNA1BP2	1	2	1	306	34.83	10.1
P05231	Interleukin-6 , GN=IL6	2	3	2	212	23.703	6.57
Q7Z478	ATP-dependent RNA helicase DHX29 , GN=DHX29	2	2	2	1369	155.139	8.09
P32004	Neural cell adhesion molecule L1 ,	1	2	1	1257	139.915	6.24
Q9Y4C8	Probable RNA-binding protein 19 ,	1	2	1	960	107.265	6.54
Q92530	Proteasome inhibitor PI31 subunit ,	2	2	2	271	29.798	5.74
Q96EK5	KIF1-binding protein , GN=KIF1BP	1	2	1	621	71.768	5.49
Q96DG6	Carboxymethylenebutenolidase homolog , GN=CMBL	1	2	1	245	28.03	7.18
Q01484	Ankyrin-2 , GN=ANK2	2	2	2	3957	433.448	5.14
Q00403	Transcription initiation factor IIB , GN=GTF2B	2	2	2	316	34.811	8.35
P56945-6	Isoform 6 of Breast cancer anti-estrogen resistance protein 1 , GN=BCAR1	2	2	2	916	97.817	5.63
O15498	Synaptobrevin homolog YKT6 , GN=YKT6	1	2	1	198	22.403	6.92
P12107-2	Isoform B of Collagen alpha-1(XI) chain , GN=COL11A1	1	2	1	1818	182.31	6.06
P52888	Thimet oligopeptidase , GN=THOP1	1	2	1	689	78.789	6.05
Q9Y4W2	Ribosomal biogenesis protein LAS1L ,	1	2	1	734	83.013	4.73
P49454	Centromere protein F , GN=CENPF	2	2	2	3210	367.537	5.07
P43121	Cell surface glycoprotein MUC18 , GN=MCAM	1	2	1	646	71.563	5.76
Q9NQR4	Omega-amidase NIT2 , GN=NIT2	2	2	2	276	30.589	7.21
Q12849	G-rich sequence factor 1 , GN=GRSF1	2	2	2	480	53.093	6.19

Q9P206-2	Isoform 2 of Uncharacterized protein KIAA1522 , GN=KIAA1522	1	2	1	1094	112.993	10.02
Q0JRZ9	F-BAR domain only protein 2 , GN=FCHO2	2	2	2	810	88.869	6.86
Q9NQ50	39S ribosomal protein L40, mitochondrial , GN=MRPL40	2	2	2	206	24.475	9.63
Q96EE3-1	Isoform B of Nucleoporin SEH1 , GN=SEH1L	1	2	1	421	46.549	7.9
Q9NXV2	BTB/POZ domain-containing protein KCTD5 , GN=KCTD5	1	2	1	234	26.076	6.24
O95674	Phosphatidate cytidylyltransferase 2 ,	1	2	1	445	51.384	7.09
Q01650	Large neutral amino acids transporter small subunit 1 , GN=SLC7A5	1	2	1	507	54.974	7.72
Q9Y4R8	Telomere length regulation protein TEL2 homolog , GN=TELO2	2	2	2	837	91.689	5.76
Q5T200	Zinc finger CCCH domain-containing protein 13 , GN=ZC3H13	1	2	1	1668	196.519	9.42
Q9NT22	EMILIN-3 , GN=EMILIN3	1	2	1	766	82.596	7.72
Q9HCM3	UPF0606 protein KIAA1549 , GN=KIAA1549	1	2	1	1950	210.625	6.11
Q8WX77	Insulin-like growth factor-binding protein-like 1 , GN=IGFBPL1 PE=2	1	2	1	278	28.987	7.93
Q8IY22	C-Maf-inducing protein , GN=CMIP	1	2	1	773	86.275	6.7
P78346-2	Isoform 2 of Ribonuclease P protein subunit p30 , GN=RPP30	1	2	1	322	35.542	9.61
Q8NB90	Spermatogenesis-associated protein 5 , GN=SPATA5	1	2	1	893	97.843	5.66
Q5T5Y3-3	Isoform 3 of Calmodulin-regulated spectrin-associated protein 1 , GN=CAMSAP1	1	2	1	1613	179.233	6.8
Q5XUX1	F-box/WD repeat-containing protein 9 , GN=FBXW9	1	2	1	488	54.081	6.48
Q9P2P1	Protein NYNRIN , GN=NYNRIN PE=2	2	2	2	1898	208.235	8.02
Q676U5	Autophagy-related protein 16-1 ,	1	2	1	607	68.223	6.64
Q14687	Genetic suppressor element 1 , GN=GSE1	1	2	1	1217	136.081	7.74
Q99567	Nuclear pore complex protein Nup88 , GN=NUP88	2	2	2	741	83.489	5.69
Q86U86	Protein polybromo-1 , GN=PBRM1	2	2	2	1689	192.825	6.89
Q8TCU4	Alstrom syndrome protein 1 , GN=ALMS1	1	2	1	4167	460.683	6.28
Q9Y520-7	Isoform 7 of Protein PRRC2C , GN=PRRC2C	2	2	2	2898	316.888	9.13
Q9GZU8	Protein FAM192A , GN=FAM192A	1	2	1	254	28.895	5.45
Q6L8Q7	2',5'-phosphodiesterase 12 , GN=PDE12	1	2	1	609	67.309	6.57
P60604	Ubiquitin-conjugating enzyme E2 G2 , GN=UBE2G2	1	2	1	165	18.554	4.7
Q13951-2	Isoform 2 of Core-binding factor subunit beta , GN=CBFB	1	2	1	187	21.978	5.58
P18846	Cyclic AMP-dependent transcription factor ATF-1 , GN=ATF1	1	2	1	271	29.215	8.37
A0JLT2	Mediator of RNA polymerase II transcription subunit 19 , GN=MED19	2	2	2	244	26.257	9.82
P01111	GTPase NRas , GN=NRAS	2	2	2	189	21.216	5.17
Q14653-4	Isoform 4 of Interferon regulatory factor 3 , GN=IRF3	1	2	1	452	49.104	6.52
P21283	V-type proton ATPase subunit C 1 , GN=ATP6V1C1	2	2	2	382	43.914	7.46
P43307	Translocon-associated protein subunit alpha , GN=SSR1	1	2	1	286	32.215	4.49
Q03154	Aminoacylase-1 , GN=ACY1	2	2	2	408	45.856	6.18
Q99747	Gamma-soluble NSF attachment protein , GN=NAPG	2	2	2	312	34.724	5.41
Q9Y2D4	Exocyst complex component 6B , GN=EXOC6B	2	2	2	811	94.141	6.46
O95861-2	Isoform 2 of 3'(2'),5'-bisphosphate nucleotidase 1 , GN=BPNT1	2	2	2	325	35.721	5.69
Q8NDI1	EH domain-binding protein 1 , GN=EHBP1	2	2	2	1231	139.931	5.35
Q9UQB3	Catenin delta-2 , GN=CTNND2	1	2	1	1225	132.574	7.74
Q01970	1-phosphatidylinositol 4,5-bisphosphate phosphodiesterase beta-3 , GN=PLCB3	1	2	1	1234	138.713	5.9
Q5SRE7	Phytanoyl-CoA dioxygenase domain-containing protein 1 , GN=PHYHD1	1	2	1	291	32.39	6.32
Q9P1Y5-2	Isoform 2 of Calmodulin-regulated spectrin-associated protein 3 , GN=CAMSAP3	1	2	1	1276	137.793	8.5

Q9BSH4	Translational activator of cytochrome c oxidase 1 , GN=TACO1	1	2	1	297	32.457	8.13
Q9BSJ2-4	Isoform 3 of Gamma-tubulin complex component 2 , GN=TUBGCP2	2	2	2	930	105.561	7.08
P27361	Mitogen-activated protein kinase 3 ,	2	2	1	379	43.108	6.74
A6NIH7	Protein unc-119 homolog B , GN=UNC119B	1	2	1	251	28.119	5.68
Q6ZMI0	Protein phosphatase 1 regulatory subunit 21 , GN=PPP1R21	1	2	1	780	88.26	6.84
Q8IY31-2	Isoform 2 of Intraflagellar transport protein 20 homolog , GN=IFT20	1	2	1	158	18.108	5.06
Q9UI08-2	Isoform 1 of Ena/VASP-like protein , GN=EVL	1	2	1	418	44.764	8.84
Q9Y3D2	Methionine-R-sulfoxide reductase B2, mitochondrial , GN=MSRB2	1	2	1	182	19.524	8.63
Q53T59	HCLS1-binding protein 3 , GN=HS1BP3	2	2	2	392	42.754	5.01
Q14135-4	Isoform 4 of Transcription cofactor vestigial-like protein 4 , GN=VGLL4	1	2	1	296	31.863	8.31
Q96KN1	Protein FAM84B , GN=FAM84B	2	2	2	310	34.453	5.54
Q8NFH4	Nucleoporin Nup37 , GN=NUP37	1	2	1	326	36.684	5.92
Q86U70	LIM domain-binding protein 1 , GN=LDB1	1	2	1	411	46.502	6.96
P47974	mRNA decay activator protein ZFP36L2 , GN=ZFP36L2	1	2	1	494	51.031	8.16
Q9C0E2	Exportin-4 , GN=XPO4	1	2	1	1151	130.056	5.05
Q96F85	CB1 cannabinoid receptor-interacting protein 1 , GN=CNRIP1	1	2	1	164	18.637	7.94
O00423-3	Isoform 3 of Echinoderm microtubule-associated protein-like 1 , GN=EML1	1	2	1	834	91.928	7.43
Q9NZN8	CCR4-NOT transcription complex subunit 2 , GN=CNOT2	2	2	2	540	59.7	7.66
P40938	Replication factor C subunit 3 , GN=RFC3	1	2	1	356	40.53	8.34
Q8WVV9	Heterogeneous nuclear ribonucleoprotein L-like , GN=HNRNPLL	1	2	1	542	60.045	7.72
O43708	Maleylacetoacetate isomerase , GN=GSTZ1	1	2	1	216	24.197	8.54
Q8NI22	Multiple coagulation factor deficiency protein 2 , GN=MCFD2	1	2	1	146	16.38	4.63
P50542-4	Isoform 4 of Peroxisomal targeting signal 1 receptor , GN=PEX5	1	2	1	654	72.246	4.51
Q13438	Protein OS-9 , GN=OS9	1	2	1	667	75.515	4.87
P48723	Heat shock 70 kDa protein 13 , GN=HSPA13	2	2	2	471	51.895	5.76
Q9UBP0	Spastin , GN=SPAST	1	2	1	616	67.155	9.64
P00374	Dihydrofolate reductase , GN=DHFR	2	2	2	187	21.439	7.42
P29084	Transcription initiation factor IIE subunit beta , GN=GTF2E2	2	2	2	291	33.023	9.66
P29317	Ephrin type-A receptor 2 , GN=EPHA2	1	2	1	976	108.197	6.23
Q9UH65	Switch-associated protein 70 , GN=SWAP70	2	2	2	585	68.954	5.87
Q9Y485	DmX-like protein 1 , GN=DMXL1	2	2	2	3027	337.625	6.34
O95453	Poly(A)-specific ribonuclease PARN ,	1	2	1	639	73.405	6.2
Q9ULE6	Paladin , GN=PALD1	2	2	2	856	96.693	6.54
Q9Y3Y2-3	Isoform 2 of Chromatin target of PRMT1 protein , GN=CHTOP	1	2	1	249	26.508	12.23
Q9H0E2	Toll-interacting protein , GN=TOLLIP	1	2	1	274	30.262	5.97
Q9ULJ8-3	Isoform 3 of Neurabin-1 , GN=PPP1R9A	1	2	1	1374	153.956	5.59
Q8TDJ6-3	Isoform 3 of DmX-like protein 2 , GN=DMXL2	1	2	1	3037	339.514	6.38
Q13144	Translation initiation factor eIF-2B subunit epsilon , GN=EIF2B5	2	2	2	721	80.329	5.08
P78362-2	Isoform 2 of SRSF protein kinase 2 ,	2	2	2	699	78.979	5.22
P20337	Ras-related protein Rab-3B , GN=RAB3B	2	2	1	219	24.742	5.02
Q6P1K2-5	Isoform 5 of Polyamine-modulated factor 1 , GN=PMF1	1	2	1	220	24.017	8.28
O14777	Kinetochore protein NDC80 homolog , GN=NDC80	2	2	2	642	73.867	5.6
Q9H6U6-8	Isoform 4 of Breast carcinoma-amplified sequence 3 , GN=BCAS3	2	2	2	950	103.596	6.71
O00512	B-cell CLL/lymphoma 9 protein , GN=BCL9	1	2	1	1426	149.194	8.91
P32189	Glycerol kinase , GN=GK	1	2	1	559	61.205	6.54
Q8TCX1	Cytoplasmic dynein 2 light intermediate chain 1 , GN=DYNC2LI1	1	2	1	351	39.6	7.53

Q12923-4	Isoform 4 of Tyrosine-protein phosphatase non-receptor type 13 , GN=PTPN13	2	2	2	2490		277.335	6.42
Q96B97	SH3 domain-containing kinase-binding protein 1 , GN=SH3KBP1	1	2	1	665		73.082	6.62
Q8WVY7	Ubiquitin-like domain-containing CTD phosphatase 1 , GN=UBLCP1	2	2	2	318		36.781	6.46
Q9Y4D8	Probable E3 ubiquitin-protein ligase HECTD4 , GN=HECTD4	2	2	2	3996		439.065	6.19
Q9NVH1	DnaJ homolog subfamily C member 11 , GN=DNAJC11	1	2	1	559		63.239	8.4
P61313	60S ribosomal protein L15 , GN=RPL15	1	2	1	204		24.131	11.62
Q8IYU8	Calcium uptake protein 2, mitochondrial , GN=MICU2	1	2	1	434		49.634	9.09
Q8WWL7	G2/mitotic-specific cyclin-B3 , GN=CCNB3	1	2	1	1395		157.816	6.68
A8MVW0	Protein FAM171A2 , GN=FAM171A2	1	2	1	826		87.381	8.05
O14545	TRAF-type zinc finger domain-containing protein 1 , GN=TRAFD1	1	2	1	582	64.8		5.29
P05412	Transcription factor AP-1 , GN=JUN	2	2	2	331		35.653	8.76
P63096	Guanine nucleotide-binding protein G(i) subunit alpha-1 , GN=GNAI1	2	2	1	354		40.335	5.97
P11166	Solute carrier family 2, facilitated glucose transporter member 1 , GN=SLC2A1	1	2	1	492		54.049	8.72
Q9UJ70-2	Isoform 2 of N-acetyl-D-glucosamine kinase , GN=NAGK	2	2	2	390		42.011	6.68
O95470	Sphingosine-1-phosphate lyase 1 , GN=SGPL1	1	2	1	568		63.483	9.16
P18859-2	Isoform 2 of ATP synthase-coupling factor 6, mitochondrial , GN=ATP5J	1	2	1	116	13.38		9.07
Q9Y6X4	Soluble lamin-associated protein of 75 kDa , GN=FAM169A	1	2	1	670		74.908	4.6
Q13243	Serine/arginine-rich splicing factor 5 ,	2	2	2	272		31.245	11.59
Q9UNW1	Multiple inositol polyphosphate phosphatase 1 , GN=MINPP1	1	2	1	487		55.016	7.81
O95302	Peptidyl-prolyl cis-trans isomerase FKBP9 , GN=FKBP9	1	2	1	570		63.044	5.08
Q9ULX7	Carbonic anhydrase 14 , GN=CA14	1	2	1	337		37.644	6.37
Q92823-5	Isoform 5 of Neuronal cell adhesion molecule , GN=NRCAM	2	2	2	1308		144.285	5.67
P26373	60S ribosomal protein L13 , GN=RPL13	1	2	1	211		24.247	11.65
Q9BVQ7	Spermatogenesis-associated protein 5-like protein 1 , GN=SPATA5L1	2	2	2	753	80.66		8.09
P82930	28S ribosomal protein S34, mitochondrial , GN=MRPS34	1	2	1	218		25.634	9.98
Q9H1A4	Anaphase-promoting complex subunit 1 , GN=ANAPC1	1	2	1	1944		216.361	6.3
Q14781	Chromobox protein homolog 2 , GN=CBX2	2	2	2	532		56.046	10.01
Q9Y2V7	Conserved oligomeric Golgi complex subunit 6 , GN=COG6	2	2	2	657		73.233	5.76
Q8IWZ8	SURP and G-patch domain-containing protein 1 , GN=SUGP1	1	2	1	645		72.425	7.61
Q5VU43-4	Isoform 4 of Myomegalin , GN=PDE4DIP	2	2	2	2362		267.014	5.48
O95251	Histone acetyltransferase KAT7 , GN=KAT7	2	2	2	611		70.598	8.85
Q9UBB6-3	Isoform 3 of Neurochondrin , GN=NCDN	1	2	1	731		79.107	5.55
Q12974	Protein tyrosine phosphatase type IVA 2 , GN=PTP4A2	2	2	2	167		19.115	8.37
Q8N0W4-2	Isoform 2 of Neuroligin-4, X-linked ,	2	2	2	836		93.988	6.05
Q9NP61	ADP-ribosylation factor GTPase-activating protein 3 , GN=ARFGAP3	2	2	2	516		56.893	7.36
O00562	Membrane-associated phosphatidylinositol transfer protein 1 , GN=PITPNM1	1	2	1	1244		134.763	5.95
Q8N4L2	Type 2 phosphatidylinositol 4,5-bisphosphate 4-phosphatase , GN=PIP4P2	1	2	1	257		28.062	8.68
Q5IJ48	Protein crumbs homolog 2 , GN=CRB2	1	2	1	1285		134.176	5.55
Q8NF91	Nesprin-1 , GN=SYNE1	2	2	2	8797		1.010.456	5.53
Q96RN5	Mediator of RNA polymerase II transcription subunit 15 , GN=MED15	2	2	2	788		86.699	9.42
Q8WVC6	Dephospho-CoA kinase domain-containing protein , GN=DCAKD	1	2	1	231		26.533	9.58

Q01780	Exosome component 10 , GN=EXOSC10	2	2	2	885	100.768	8.46
F5HDY6	Protein ORF27 OS=Human herpesvirus 8 type P (isolate GK18) GN=ORF27 PE=4	1	2	1	290	32.166	5.33
O75521	Enoyl-CoA delta isomerase 2, mitochondrial , GN=ECI2	1	2	1	394	43.557	9
Q86WI1	Fibrocystin-L , GN=PKHD1L1 PE=2	1	2	1	4243	465.439	6.11
O43660	Pleiotropic regulator 1 , GN=PLRG1	2	2	2	514	57.158	9.17
O60306	Intron-binding protein aquarius , GN=AQR	1	2	1	1485	171.186	6.37
Q9C0H6-2	Isoform 2 of Kelch-like protein 4 , GN=KLHL4	2	2	2	720	80.783	6.81
Q6ZVM7	TOM1-like protein 2 , GN=TOM1L2	2	2	2	507	55.522	4.79
O15231-6	Isoform 6 of Zinc finger protein 185 ,	1	2	1	721	76.81	7.05
P62256	Ubiquitin-conjugating enzyme E2 H ,	1	2	1	183	20.642	4.67
Q9UI10-2	Isoform 2 of Translation initiation factor eIF-2B subunit delta , GN=EIF2B4	1	2	1	543	59.578	9.42
Q49A26	Putative oxidoreductase GLYR1 , GN=GLYR1	2	2	2	553	60.518	9.17
Q96FZ2	Embryonic stem cell-specific 5-hydroxymethylcytosine-binding protein , GN=HMCES	2	2	2	354	40.549	8.15
Q0VF96	Cingulin-like protein 1 , GN=CGNL1	1	2	1	1302	148.989	5.67
Q9P2T1-2	Isoform 2 of GMP reductase 2 , GN=GMPR2	1	2	1	366	39.779	7.65
Q66577	Genome polyprotein OS=Echovirus 9 (strain Barty) PE=3	2	2	2	2203	246.122	6.79
Q5H9R7-5	Isoform 5 of Serine/threonine-protein phosphatase 6 regulatory subunit 3 , GN=PPP6R3	1	2	1	879	98.423	4.64
Q9H9C1	Spermatogenesis-defective protein 39 homolog , GN=VIPAS39	2	2	2	493	56.97	7.4
Q5TAT6	Collagen alpha-1(XIII) chain , GN=COL13A1	1	2	1	717	69.907	9.17
P38117-2	Isoform 2 of Electron transfer flavoprotein subunit beta , GN=ETFB	2	2	2	346	37.411	7.17
O14880	Microsomal glutathione S-transferase 3 , GN=MGST3	1	2	1	152	16.506	9.38
Q86VF2-5	Isoform 5 of Immunoglobulin-like and fibronectin type III domain-containing protein 1 , GN=IGFN1	2	2	2	3708	383.568	5.99
Q7Z589-7	Isoform 7 of BRCA2-interacting transcriptional repressor EMSY , GN=EMSY	2	2	2	1337	143.104	9.35
A5D8V6	Vacuolar protein sorting-associated protein 37C , GN=VPS37C	2	2	2	355	38.635	5.21
Q96LJ7	Dehydrogenase/reductase SDR family member 1 , GN=DHRS1	1	2	1	313	33.887	7.83
O94910	Adhesion G protein-coupled receptor L1 , GN=ADGRL1	1	2	1	1474	162.614	6.6
Q765P7	MTSS1-like protein , GN=MTSS1L	2	2	2	747	79.88	7.47
Q9UIG0	Tyrosine-protein kinase BAZ1B , GN=BAZ1B	2	2	2	1483	170.796	8.48
Q6IQ49	Protein SDE2 homolog , GN=SDE2	2	2	2	451	49.711	6.05
O75150	E3 ubiquitin-protein ligase BRE1B , GN=RNF40	2	2	2	1001	113.581	6.23
O00534	von Willebrand factor A domain-containing protein 5A , GN=VWA5A PE=2	2	2	2	786	86.434	6.58
Q96CS3	FAS-associated factor 2 , GN=FAF2	2	2	2	445	52.591	5.62
Q9NV88	Integrator complex subunit 9 , GN=INTS9	2	2	2	658	73.767	6.33
O14908	PDZ domain-containing protein GIPC1 , GN=GIPC1	1	2	1	333	36.027	6.28
Q9NVA1	Ubiquinol-cytochrome-c reductase complex assembly factor 1 , GN=UQCC1	1	2	1	299	34.578	8.91
Q8TD16-2	Isoform 2 of Protein bicaudal D homolog 2 , GN=BICD2	2	2	2	855	96.746	5.44
Q8NB49	Phospholipid-transporting ATPase IG , GN=ATP11C	2	2	2	1132	129.394	6.67
Q9NZ53	Podocalyxin-like protein 2 , GN=PODXL2	1	2	1	605	65.036	4.34
Q16512-2	Isoform 2 of Serine/threonine-protein kinase N1 , GN=PKN1	2	2	2	948	104.608	6.15
Q9P086	Mediator of RNA polymerase II transcription subunit 11 , GN=MED11	1	2	1	117	13.121	5.96
P05067	Amyloid-beta A4 protein , GN=APP	2	2	2	770	86.888	4.82
Q8WVK2	U4/U6.U5 small nuclear ribonucleoprotein 27 kDa protein , GN=SNRNP27	1	2	1	155	18.849	11.62

P62277	40S ribosomal protein S13 , GN=RPS13	1	2	1	151	17.212	10.54
Q9BQ52	Zinc phosphodiesterase ELAC protein 2 , GN=ELAC2	1	2	1	826	92.16	7.9
Q2NL82	Pre-rRNA-processing protein TSR1 homolog , GN=TSR1	1	2	1	804	91.752	7.42
P07711	Cathepsin L1 , GN=CTSL	1	2	1	333	37.54	5.45
Q9H2M9	Rab3 GTPase-activating protein non-catalytic subunit , GN=RAB3GAP2	2	2	2	1393	155.886	5.62
Q8IYB5	Stromal membrane-associated protein 1 , GN=SMAP1	1	2	1	467	50.354	8.75
P50583	Bis(5'-nucleosyl)-tetraphosphatase [asymmetrical] , GN=NUDT2	1	2	1	147	16.819	5.35
Q9NV35	Nucleotide triphosphate diphosphatase NUDT15 , GN=NUDT15	2	2	2	164	18.597	6.14
Q8N5N7	39S ribosomal protein L50, mitochondrial , GN=MRPL50	2	2	2	158	18.313	7.88
P46778	60S ribosomal protein L21 , GN=RPL21	1	2	1	160	18.553	10.49
Q8NFJ9-2	Isoform 3 of Bardet-Biedl syndrome 1 protein , GN=BBS1	2	2	2	630	69.614	8.5
Q99986	Serine/threonine-protein kinase VRK1 , GN=VRK1	2	2	2	396	45.447	8.91
Q9BYV8	Centrosomal protein of 41 kDa , GN=CEP41	1	2	1	373	41.343	8.32
P11274	Breakpoint cluster region protein , GN=BCR	1	2	1	1271	142.73	7.03
Q08AD1	Calmodulin-regulated spectrin-associated protein 2 , GN=CAMSAP2	2	2	2	1489	167.984	6.8
Q9NRS6	Sorting nexin-15 , GN=SNX15	1	2	1	342	38.268	5.19
Q96BP3	Peptidylprolyl isomerase domain and WD repeat-containing protein 1 , GN=PPWD1	2	2	2	646	73.528	7.15
O95376	E3 ubiquitin-protein ligase ARIH2 , GN=ARIH2	2	2	2	493	57.781	5.63
Q96JP5	E3 ubiquitin-protein ligase ZFP91 , GN=ZFP91	1	2	1	570	63.406	7.36
Q9NPF4	Probable tRNA N6-adenosine threonylcarbamoyltransferase , GN=OSGEP	2	2	2	335	36.403	6.35
Q15185	Prostaglandin E synthase 3 , GN=PTGES3	2	2	2	160	18.685	4.54
Q13523	Serine/threonine-protein kinase PRP4 homolog , GN=PRPF4B	2	2	2	1007	116.916	10.26
Q96PU5	E3 ubiquitin-protein ligase NEDD4-like , GN=NEDD4L	1	2	1	975	111.862	5.82
Q9BYD1	39S ribosomal protein L13, mitochondrial , GN=MRPL13	2	2	2	178	20.679	9.16
Q86V81	THO complex subunit 4 , GN=ALYREF	2	2	2	257	26.872	11.15
Q9Y2H0	Disks large-associated protein 4 , GN=DLGAP4	1	2	1	992	107.945	7.08
Q04721	Neurogenic locus notch homolog protein 2 , GN=NOTCH2	2	2	2	2471	265.226	5.14
P49720	Proteasome subunit beta type-3 , GN=PSMB3	1	2	1	205	22.933	6.55
Q13043	Serine/threonine-protein kinase 4 , GN=STK4	1	2	1	487	55.595	5.07
P56134	ATP synthase subunit f, mitochondrial , GN=ATP5J2	1	2	1	94	10.911	9.67
Q8WUB8	PHD finger protein 10 , GN=PHF10	2	2	2	498	56.015	6.62
Q9UBB9	Tuftelin-interacting protein 11 , GN=TFIP11	2	2	2	837	96.758	5.67
P55285	Cadherin-6 , GN=CDH6	2	2	2	790	88.254	4.93
Q00325	Phosphate carrier protein, mitochondrial , GN=SLC25A3	1	2	1	362	40.069	9.38
P42356	Phosphatidylinositol 4-kinase alpha ,	1	2	1	2102	236.678	7.06
Q9BX66-10	Isoform 10 of Sorbin and SH3 domain-containing protein 1 , GN=SORBS1	2	2	2	816	90.163	6.47
Q9NXN4	Ganglioside-induced differentiation-associated protein 2 , GN=GDAP2	1	2	1	497	56.189	5.74
Q96GQ7	Probable ATP-dependent RNA helicase DDX27 , GN=DDX27	2	2	2	796	89.779	9.28
Q8TDC3-2	Isoform 2 of Serine/threonine-protein kinase BRSK1 , GN=BRSK1	1	2	1	794	86.7	8.94
Q9BUR4	Telomerase Cajal body protein 1 ,	1	2	1	548	59.272	4.58
Q15645	Pachytene checkpoint protein 2 homolog , GN=TRIP13	1	2	1	432	48.52	6.09
Q02750	Dual specificity mitogen-activated protein kinase kinase 1 , GN=MAP2K1	2	2	1	393	43.411	6.62

O00330-3	Isoform 3 of Pyruvate dehydrogenase protein X component, mitochondrial , GN=PDHX	2	2	2	486	51.428	6.34
Q8N201	Integrator complex subunit 1 , GN=INTS1	2	2	2	2190	244.143	6.13
Q14031	Collagen alpha-6(IV) chain , GN=COL4A6	2	2	2	1691	163.704	9.2
Q6NXE6	Armadillo repeat-containing protein 6 , GN=ARMC6	2	2	2	501	54.107	6.24
Q14999	Cullin-7 , GN=CUL7	2	2	2	1698	191.04	5.87
O60245-2	Isoform B of Protocadherin-7 , GN=PCDH7	2	2	2	1072	116.358	5.2
O43572	A-kinase anchor protein 10, mitochondrial , GN=AKAP10	1	2	1	662	73.772	6.39
Q99797	Mitochondrial intermediate peptidase , GN=MIPEP	2	2	2	713	80.589	7.05
Q15363	Transmembrane emp24 domain-containing protein 2 , GN=TMED2	1	2	1	201	22.746	5.17
O15040	Tectonin beta-propeller repeat-containing protein 2 , GN=TECPR2	2	2	2	1411	153.751	5.55
Q86UU1	Pleckstrin homology-like domain family B member 1 , GN=PHLDB1	1	2	1	1377	151.068	8.63
Q6ZT12-4	Isoform 4 of E3 ubiquitin-protein ligase UBR3 , GN=UBR3	1	2	1	1917	215.831	6.2
Q8NCN4	E3 ubiquitin-protein ligase RNF169 ,	2	2	2	708	77.147	9.1
Q9NWV4	UPF0587 protein C1orf123 , GN=C1orf123	1	2	1	160	18.037	5.01
Q15003	Condensin complex subunit 2 , GN=NCAPH	2	2	2	741	82.511	5.06
Q9UPU5	Ubiquitin carboxyl-terminal hydrolase 24 , GN=USP24	2	2	2	2620	294.178	6.14
P24043	Laminin subunit alpha-2 , GN=LAMA2	2	2	2	3122	343.684	6.4
Q9UBQ0-2	Isoform 2 of Vacuolar protein sorting-associated protein 29 , GN=VPS29	2	2	2	186	20.914	7.05
Q4LE39	AT-rich interactive domain-containing protein 4B , GN=ARID4B	1	1	1	1312	147.719	5.12
P55212	Caspase-6 , GN=CASP6	2	2	2	293	33.288	6.93
Q8NBL1	Protein O-glucosyltransferase 1 ,	2	2	2	392	46.159	8.72
Q9BY41	Histone deacetylase 8 , GN=HDAC8	2	2	2	377	41.731	5.63
Q9NWV8	BRISC and BRCA1-A complex member 1 , GN=BABAM1	2	2	2	329	36.537	4.64
Q86UK7	E3 ubiquitin-protein ligase ZNF598 ,	1	2	1	904	98.575	8.4
Q9H0D6	5'-3' exoribonuclease 2 , GN=XRN2	1	2	1	950	108.513	7.47
Q8NBF6	Late secretory pathway protein AVL9 homolog , GN=AVL9	1	2	1	648	71.902	6.21
Q8IUI8	Cytokine receptor-like factor 3 , GN=CRLF3	1	2	1	442	49.735	5.14
Q13630	GDP-L-fucose synthase , GN=TSTA3	1	2	1	321	35.87	6.6
Q12841	Follistatin-related protein 1 , GN=FSTL1	1	2	1	308	34.963	5.52
Q8NFP7	Diphosphoinositol polyphosphate phosphohydrolase 3-alpha , GN=NUDT10	1	2	1	164	18.488	5.68
Q9P253	Vacuolar protein sorting-associated protein 18 homolog , GN=VPS18	2	2	2	973	110.116	6.07
Q8N448	Ligand of Numb protein X 2 , GN=LNX2	2	2	2	690	75.956	6.64
A7E2V4-2	Isoform 2 of Zinc finger SWIM domain-containing protein 8 , GN=ZSWIM8	1	2	1	1892	201.395	6.58
P13995	Bifunctional methylenetetrahydrofolate dehydrogenase/cyclohydrolase, mitochondrial , GN=MTHFD2	1	2	1	350	37.871	8.73
O95685	Protein phosphatase 1 regulatory subunit 3D , GN=PPP1R3D	1	2	1	299	32.538	8.07
P62750	60S ribosomal protein L23a , GN=RPL23A	2	2	2	156	17.684	10.45
Q9C040-2	Isoform 2 of Tripartite motif-containing protein 2 , GN=TRIM2	2	2	2	771	84.492	7.49
O95295	SNARE-associated protein Snapin ,	2	2	2	136	14.865	9.31
Q9Y5K5	Ubiquitin carboxyl-terminal hydrolase isozyme L5 , GN=UCHL5	1	2	1	329	37.583	5.33
Q96C57	Uncharacterized protein C12orf43 , GN=C12orf43	1	2	1	262	28.153	9.42
Q15154	Pericentriolar material 1 protein , GN=PCM1	2	2	2	2024	228.392	5.02
Q6UUV7	CREB-regulated transcription coactivator 3 , GN=CRTC3	2	2	2	619	66.918	6.84
Q53H47	Histone-lysine N-methyltransferase SETMAR , GN=SETMAR	1	2	1	684	77.984	7.14

Q5HYI7	Metaxin-3 , GN=MTX3	2	2	2	312		35.071	7.8
P21127	Cyclin-dependent kinase 11B , GN=CDK11B	1	2	1	795	92.65		5.57
P35052	Glypican-1 , GN=GPC1	1	2	1	558		61.641	7.3
P43115-12	Isoform 12 of Prostaglandin E2 receptor EP3 subtype , GN=PTGER3	1	2	1	429		47.705	9.98
Q9HA90	EF-hand and coiled-coil domain-containing protein 1 , GN=EFCC1 PE=2	2	2	2	598		65.661	5.6
O00461	Golgi integral membrane protein 4 ,	2	2	2	696		81.831	4.77
Q8TF05	Serine/threonine-protein phosphatase 4 regulatory subunit 1 , GN=PPP4R1	2	2	2	950		106.936	4.77
Q96CP2	FLYWCH family member 2 , GN=FLYWCH2	1	2	1	140		14.555	8.46
Q8NFZ8	Cell adhesion molecule 4 , GN=CADM4	2	2	2	388		42.759	6.3
Q96A83	Collagen alpha-1(XXVI) chain , GN=COL26A1	1	2	1	441		45.353	7.31
Q9Y5X2	Sorting nexin-8 , GN=SNX8	1	2	1	465		52.536	7.39
Q5T5U3	Rho GTPase-activating protein 21 , GN=ARHGAP21	2	2	2	1957		217.198	7.8
Q96EY5	Multivesicular body subunit 12A ,	1	2	1	273		28.765	8.91
P55769	NHP2-like protein 1 , GN=SNU13	1	2	1	128		14.165	8.46
P33527-9	Isoform 9 of Multidrug resistance-associated protein 1 , GN=ABCC1	2	2	2	1541	172.53		6.81
Q86W42	THO complex subunit 6 homolog , GN=THOC6	2	2	2	341		37.511	7.43
P53597	Succinate--CoA ligase [ADP/GDP-forming] subunit alpha, mitochondrial , GN=SUCLG1	1	2	1	346		36.227	8.79
P46976	Glycogenin-1 , GN=GYG1	1	2	1	350		39.359	5.53
Q6UB35	Monofunctional C1-tetrahydrofolate synthase, mitochondrial , GN=MTHFD1L	2	2	2	978		105.724	8.06
P11217	Glycogen phosphorylase, muscle form , GN=PYGM SV=6	2	2	2	842		97.031	7.03
Q9UGM6	Tryptophan--tRNA ligase, mitochondrial , GN=WARS2	1	2	1	360		40.121	9.28
P41229	Lysine-specific demethylase 5C , GN=KDM5C	2	2	2	1560		175.609	5.58
Q8TC07	TBC1 domain family member 15 ,	1	2	1	691	79.44		5.67
Q8N5D0	WD and tetratricopeptide repeats protein 1 , GN=WDTC1	2	2	2	677		75.872	7.27
P05204	Non-histone chromosomal protein HMG-17 , GN=HMGN2	1	2	1	90		9.387	9.99
Q9BU61	NADH dehydrogenase [ubiquinone] 1 alpha subcomplex assembly factor 3 , GN=NDUFAF3	2	2	2	184		20.337	8.22
Q147X3	N-alpha-acetyltransferase 30 , GN=NAA30	2	2	2	362		39.295	5.52
Q7L0J3	Synaptic vesicle glycoprotein 2A , GN=SV2A	1	2	1	742		82.642	5.57
Q9UBQ5	Eukaryotic translation initiation factor 3 subunit K , GN=EIF3K	1	2	1	218		25.043	4.93
P61964	WD repeat-containing protein 5 , GN=WDR5	2	2	2	334		36.565	8.27
Q8WUW1-2	Isoform 2 of Protein BRICK1 , GN=BRK1	1	2	1	106		12.038	6.76
Q92643	GPI-anchor transamidase , GN=PIGK	1	2	1	395		45.223	6.16
Q9H2W6	39S ribosomal protein L46, mitochondrial , GN=MRPL46	1	2	1	279		31.685	7.05
Q5VZL5	Zinc finger MYM-type protein 4 , GN=ZMYM4	2	2	2	1548		172.677	6.84
Q92791	Endoplasmic reticulum protein SC65 ,	2	2	2	437		50.349	4.77
Q5XXP4	Non-structural polyprotein OS=Chikungunya virus (strain 37997) PE=2	1	2	1	2474		275.756	7.2
Q14684	Ribosomal RNA processing protein 1 homolog B , GN=RRP1B	2	2	2	758		84.375	9.76
Q8NBU5	ATPase family AAA domain-containing protein 1 , GN=ATAD1	1	2	1	361		40.718	6.9
Q96I25	Splicing factor 45 , GN=RBM17	2	2	2	401		44.934	5.97
Q15274	Nicotinate-nucleotide pyrophosphorylase [carboxylating] , GN=QPRT	2	2	2	297		30.826	6.21
Q13439-5	Isoform 5 of Golgin subfamily A member 4 , GN=GOLGA4	1	2	1	2243		262.167	5.4
Q96QZ7	Membrane-associated guanylate kinase, WW and PDZ domain-containing protein 1 , GN=MAGI1	1	2	1	1491		164.481	7.58
O75175	CCR4-NOT transcription complex subunit 3 , GN=CNOT3	1	2	1	753		81.822	6.2
O00255	Menin , GN=MEN1	1	2	1	615		67.981	6.55
Q69YN4	Protein virilizer homolog , GN=VIRMA	1	2	1	1812		201.898	5.01

O75164	Lysine-specific demethylase 4A , GN=KDM4A	2	2	2	1064		120.585	5.85	
Q9ULH0	Kinase D-interacting substrate of 220 kDa , GN=KIDINS220	1	2	1	1771		196.419	6.62	
Q6PML9	Zinc transporter 9 , GN=SLC30A9	1	2	1	568		63.475	8.32	
Q9ULU4-19	Isoform 19 of Protein kinase C-binding protein 1 , GN=ZMYND8	2	2	2	1241		137.943	6.93	
Q9NUD5-2	Isoform 2 of Zinc finger CCHC domain-containing protein 3 , GN=ZCCHC3	2	2	2	239		26.637	8.57	
P41162	ETS translocation variant 3 , GN=ETV3	1	2	1	512		56.966	8.57	
P81133	Single-minded homolog 1 , GN=SIM1 PE=2	1	2	1	766		85.461	7.43	
P18754-2	Isoform 2 of Regulator of chromosome condensation , GN=RCC1	1	2	1	452		48.115	8.16	
Q9BQT9-2	Isoform 2 of Calsyntenin-3 , GN=CLSTN3	1	1	1	968		106.966	5.38	
P35556	Fibrillin-2 , GN=FBN2	1	2	1	2912		314.558	4.86	
Q9NVX0	HAUS augmin-like complex subunit 2 , GN=HAUS2	1	2	1	235		26.916	7.88	
Q8TEM1	Nuclear pore membrane glycoprotein 210 , GN=NUP210	1	2	1	1887		204.983	6.81	
Q96DB5	Regulator of microtubule dynamics protein 1 , GN=RMDN1	1	2	1	314		35.785	8.5	
Q8NCM8-2	Isoform 2 of Cytoplasmic dynein 2 heavy chain 1 , GN=DYNC2H1	1	2	1	4314		493.109	6.57	
P78330	Phosphoserine phosphatase , GN=PSPH	1	2	1	225		24.992	5.69	
Q8WVF1	Protein OSCP1 , GN=OSCP1	1	2	1	389		44.558	5.83	
Q9UPN9-2	Isoform Beta of E3 ubiquitin-protein ligase TRIM33 , GN=TRIM33	2	2	2	1110		120.465	6.67	
O15511-2	Isoform 2 of Actin-related protein 2/3 complex subunit 5 , GN=ARPC5	1	2	1	154	16.62		6.02	
Q9HCE5	N6-adenosine-methyltransferase non-catalytic subunit , GN=METTL14	1	2	1	456		52.118	6.21	
Q9Y2S2	Lambda-crystallin homolog , GN=CRYL1	1	2	1	319		35.396	6.18	
Q9H0E9-4	Isoform 4 of Bromodomain-containing protein 8 , GN=BRD8	1	2	1	866		94.238	4.7	
Q9NXF7	DDB1- and CUL4-associated factor 16 , GN=DCAF16	1	2	1	216		24.178	6.04	
Q92552-2	Isoform 2 of 28S ribosomal protein S27, mitochondrial , GN=MRPS27	1	2	1	428		49.119	6.33	
Q9Y279	V-set and immunoglobulin domain-containing protein 4 , GN=VSIG4	1	2	1	399		43.959	6.35	
Q9HAS0	Protein Njmu-R1 , GN=C17orf75	1	2	1	396		44.593	5.03	
Q86U28	Iron-sulfur cluster assembly 2 homolog, mitochondrial , GN=ISCA2	1	2	1	154		16.466	5.25	
Q99933	BAG family molecular chaperone regulator 1 , GN=BAG1	1	2	1	345		38.755	7.81	
O00203	AP-3 complex subunit beta-1 , GN=AP3B1	2	2	2	1094		121.244	6.04	
F5HEN7	Small capsomere-interacting protein OS=Human cytomegalovirus (strain Merlin) GN=SCP PE=3	1	2	1	75		8.489	9.99	
Q587I9	Vesicle transport protein SFT2C , GN=SFT2D3 PE=2	1	2	1	215		21.776	9.89	
Q8WUH2	Transforming growth factor-beta receptor-associated protein 1 , GN=TGFBRAP1	1	2	1	860		97.096	6.55	
Q6UW63	KDEL motif-containing protein 1 , GN=KDELC1	1	2	1	502		58.005	7.71	
Q9UQ84	Exonuclease 1 , GN=EXO1	1	2	1	846		94.045	8.31	
Q01995	Transgelin , GN=TAGLN	1	2	1	201		22.596	8.84	
A0FGR8-6	Isoform 6 of Extended synaptotagmin-2 , GN=ESYT2	1	2	1	942		104.643	9.33	
Q8IWT0	Protein archease , GN=ZBTB8OS	1	2	1	167		19.478	4.49	
Q02413	Desmoglein-1 , GN=DSG1	1	2	1	1049		113.676	5.03	
P08574	Cytochrome c1, heme protein, mitochondrial , GN=CYC1	1	2	1	325		35.399		9
Q9HA64	Ketosamine-3-kinase , GN=FN3KRP	1	2	1	309	34.39		7.33	
P53007	Tricarboxylate transport protein, mitochondrial , GN=SLC25A1	1	2	1	311		33.991	9.89	
O00115	Deoxyribonuclease-2-alpha , GN=DNASE2	1	2	1	360		39.556	8.05	
Q16595	Frataxin, mitochondrial , GN=FXN	2	2	2	210		23.121	8.69	

Q94875-11	Isoform 11 of Sorbin and SH3 domain-containing protein 2 , GN=SORBS2	1	2	1	1200	134.51	8.53
P06400	Retinoblastoma-associated protein , GN=RB1	1	2	1	928	106.092	7.94
Q9BQK8-2	Isoform 2 of Phosphatidate phosphatase LPIN3 , GN=LPIN3	1	2	1	852	93.643	5.52
P55735-3	Isoform 3 of Protein SEC13 homolog ,	1	2	1	368	40.721	5.85
P16383	GC-rich sequence DNA-binding factor 2 , GN=GCFC2	1	2	1	781	89.329	5.99
Q9UQ88-8	Isoform SV12 of Cyclin-dependent kinase 11A , GN=CDK11A	1	2	1	167	18.099	9.31
Q96CM3	Mitochondrial RNA pseudouridine synthase RPUSD4 , GN=RPUSD4	2	2	2	377	42.178	9.88
O94761	ATP-dependent DNA helicase Q4 ,	2	2	2	1208	132.993	8.09
Q13217	DnaJ homolog subfamily C member 3 , GN=DNAJC3	1	1	1	504	57.544	6.15
Q99856	AT-rich interactive domain-containing protein 3A , GN=ARID3A	2	2	2	593	62.85	4.91
Q86VQ1	Glucocorticoid-induced transcript 1 protein , GN=GLCCI1	1	2	1	547	57.988	9.44
Q9C0H2-4	Isoform 4 of Protein tweety homolog 3 , GN=TTYH3	1	2	1	526	57.945	5.31
Q96C01	Protein FAM136A , GN=FAM136A	1	2	1	138	15.631	7.61
Q8IU81	Interferon regulatory factor 2-binding protein 1 , GN=IRF2BP1	2	2	2	584	61.649	8.18
O14686-3	Isoform 3 of Histone-lysine N-methyltransferase 2D , GN=KMT2D	2	2	2	5540	593.304	5.58
Q7Z6K5-2	Isoform C15orf38-AP3S2 of Arpin , GN=ARPIN	1	2	1	394	43.854	5.3
P46020	Phosphorylase b kinase regulatory subunit alpha, skeletal muscle isoform , GN=PHKA1	2	2	2	1223	137.225	6.19
Q99569	Plakophilin-4 , GN=PKP4	1	2	1	1192	131.787	8.94
P82933	28S ribosomal protein S9, mitochondrial , GN=MRPS9	1	2	1	396	45.806	9.51
O15056	Synaptojanin-2 , GN=SYNJ2	2	2	2	1496	165.435	7.34
P36969	Phospholipid hydroperoxide glutathione peroxidase, mitochondrial , GN=GPX4	2	2	2	197	22.161	8.37
O60469	Down syndrome cell adhesion molecule , GN=DSCAM	1	2	1	2012	222.121	7.68
Q3V6T2	Girdin , GN=CCDC88A	2	2	2	1871	215.909	6.21
P35520-2	Isoform 2 of Cystathionine beta-synthase , GN=CBS	1	2	1	565	61.824	6.44
P53814-6	Isoform B3 of Smoothelin , GN=SMTN	1	2	1	940	101.748	8.78
A6NDU8	UPF0600 protein C5orf51 , GN=C5orf51	1	1	1	294	33.599	5.26
Q9Y4C2	TRPM8 channel-associated factor 1 ,	2	2	2	921	102.061	6.54
P22681	E3 ubiquitin-protein ligase CBL , GN=CBL	2	2	2	906	99.569	6.54
O75414	Nucleoside diphosphate kinase 6 , GN=NME6	1	2	1	186	21.129	8.32
Q04206	Transcription factor p65 , GN=RELA	1	2	1	551	60.181	5.68
Q10471	acetylgalactosaminyltransferase 2 , GN=GALNT2	1	2	1	571	64.691	8.35
Q9NXR7-1	Isoform 1 of BRISC and BRCA1-A complex member 2 , GN=BABAM2	1	2	1	415	46.944	6.14
Q9HCE9	Anoctamin-8 , GN=ANO8	1	2	1	1232	135.949	5.82
A6NEF3	Golgin subfamily A member 6-like protein 4 , GN=GOLGA6L4 PE=3	1	2	1	574	67.522	4.79
Q9HCP6	Protein-cysteine N-palmitoyltransferase HHAT-like protein , GN=HHATL PE=2	1	2	1	504	56.666	7.12
Q5T1M5	FK506-binding protein 15 , GN=FKBP15	1	2	1	1219	133.547	5.2
P36915	Guanine nucleotide-binding protein-like 1 , GN=GNL1	2	2	2	607	68.619	5.8
Q9BXB4	Oxysterol-binding protein-related protein 11 , GN=OSBPL11	1	2	1	747	83.591	7.06
Q8N3C7	CAP-Gly domain-containing linker protein 4 , GN=CLIP4	1	2	1	705	76.269	8.7
Q9P260-2	Isoform 2 of LisH domain and HEAT repeat-containing protein KIAA1468 , GN=KIAA1468	2	2	2	1250	138.657	5.53
P01185	Vasopressin-neurophysin 2-copeptin ,	1	2	1	164	17.313	5.29
Q6AI08	HEAT repeat-containing protein 6 ,	1	2	1	1181	128.699	7.03

Q9HD42	Charged multivesicular body protein 1a , GN=CHMP1A	1	2	1	196		21.689	8.06
Q13325	Interferon-induced protein with tetratricopeptide repeats 5 , GN=IFIT5	1	2	1	482		55.812	7.4
Q96A99	Pentraxin-4 , GN=PTX4 PE=2	1	2	1	478		52.307	9.55
Q9NWB6	Arginine and glutamate-rich protein 1 , GN=ARGLU1	1	2	1	273		33.197	10.35
Q9NY47-5	Isoform 5 of Voltage-dependent calcium channel subunit alpha-2/delta-2 , GN=CACNA2D2	2	2	2	1152		129.919	5.8
Q99584	Protein S100-A13 , GN=S100A13	1	2	1	98		11.464	6.16
Q8IVL0	Neuron navigator 3 , GN=NAV3	2	2	2	2385		255.491	8.76
Q15269	Periodic tryptophan protein 2 homolog , GN=PWP2 PE=2	1	2	1	919		102.387	6.15
Q92542	Nicastrin , GN=NCSTN	1	2	1	709		78.362	5.99
P35268	60S ribosomal protein L22 , GN=RPL22	1	2	1	128		14.778	9.19
Q9H0R8-2	receptor-associated protein-like 1 , GN=GABARAPL1	1	2	1	146		16.997	9.5
Q86X83	COMM domain-containing protein 2 , GN=COMMD2	1	1	1	199		22.731	6.73
Q86W50	U6 small nuclear RNA (adenine-(43)-N(6))-methyltransferase , GN=METTL16	1	1	1	562		63.581	7.85
Q9BT25	HAUS augmin-like complex subunit 8 , GN=HAUS8	1	1	1	410	44.83		7.06
Q9NZR1	Tropomodulin-2 , GN=TMOD2	1	1	1	351		39.571	5.27
Q9BQ69	O-acetyl-ADP-ribose deacetylase MACROD1 , GN=MACROD1	1	1	1	325		35.483	9.51
Q86TS9	39S ribosomal protein L52, mitochondrial , GN=MRPL52	1	1	1	123		13.655	10.26
Q9UIS9-12	Isoform 12 of Methyl-CpG-binding domain protein 1 , GN=MBD1	1	1	1	655		72.079	8.78
Q86TU7	Histone-lysine N-methyltransferase setd3 , GN=SETD3	1	1	1	594		67.215	5.96
A6NED2	RCC1 domain-containing protein 1 ,	1	1	1	376		40.054	5.27
Q9UBQ6	Exostosin-like 2 , GN=EXTL2	1	1	1	330		37.441	8.95
Q8N3C0	Activating signal cointegrator 1 complex subunit 3 , GN=ASCC3	1	1	1	2202		251.301	7.09
O94952	F-box only protein 21 , GN=FBXO21 PE=2	1	1	1	628		72.224	6.09
Q5T653	39S ribosomal protein L2, mitochondrial , GN=MRPL2	1	1	1	305	33.28		11.3
Q9NZU0	Leucine-rich repeat transmembrane protein FLRT3 , GN=FLRT3	1	1	1	649		72.957	7.64
Q6N022	Teneurin-4 , GN=TENM4	1	1	1	2769		307.761	6.55
Q96G46	tRNA-dihydrouridine(47) synthase [NAD(P)(+)]-like , GN=DUS3L	1	1	1	650		72.548	8.05
Q9NVC6	Mediator of RNA polymerase II transcription subunit 17 , GN=MED17	1	1	1	651		72.845	7.44
Q6GMV2	SET and MYND domain-containing protein 5 , GN=SMYD5	1	1	1	418	47.31		5.05
Q96ED9	Protein Hook homolog 2 , GN=HOOK2	1	1	1	719		83.155	5.47
Q9Y276	Mitochondrial chaperone BCS1 , GN=BCS1L	1	1	1	419		47.504	8.5
Q63HN8-4	Isoform 2 of E3 ubiquitin-protein ligase RNF213 , GN=RNF213	1	1	1	5256		596.106	6.43
Q15042-3	Isoform 2 of Rab3 GTPase-activating protein catalytic subunit , GN=RAB3GAP1	1	1	1	988		111.215	5.45
Q96PZ0	Pseudouridylate synthase 7 homolog ,	1	1	1	661		74.988	6.37
Q5TGY3	AT-hook DNA-binding motif-containing protein 1 , GN=AHDC1	1	1	1	1603		168.245	9.04
Q92845	Kinesin-associated protein 3 , GN=KIFAP3	1	1	1	792		91.146	5.08
Q8IZ40	REST corepressor 2 , GN=RCOR2	1	1	1	523		57.976	9.01
Q9UL63	Muskelin , GN=MKLN1	1	1	1	735		84.713	6.34
P27707	Deoxycytidine kinase , GN=DCK	1	1	1	260		30.499	5.21
Q96EP0	E3 ubiquitin-protein ligase RNF31 ,	1	1	1	1072		119.575	6.57
Q96BJ3	Axin interactor, dorsalization-associated protein , GN=AIDA	1	1	1	306		35.001	6.55
Q9UBT7	Alpha-catulin , GN=CTNNAL1	1	1	1	734		81.845	6.64

Q9Y478	5'-AMP-activated protein kinase subunit beta-1 , GN=PRKAB1	1	1	1	270	30.363	6.4
Q9Y5B6	PAX3- and PAX7-binding protein 1 ,	1	1	1	917	104.739	5.68
Q9NZJ4	Sacsin , GN=SACS	1	1	1	4579	520.795	7.05
Q9BY67-3	Isoform 3 of Cell adhesion molecule 1 , GN=CADM1	1	1	1	471	51.501	4.87
Q6NYC8	Phostensin , GN=PPP1R18	1	1	1	613	67.902	5.4
Q9Y3S1	Serine/threonine-protein kinase WNK2 , GN=WNK2	1	1	1	2297	242.525	6.13
Q96K17	Transcription factor BTF3 homolog 4 , GN=BTF3L4	1	1	1	158	17.26	6.35
O94766	Galactosylgalactosylxylosylprotein 3-beta-glucuronosyltransferase 3 , GN=B3GAT3	1	1	1	335	37.099	8.27
A1L0T0	Acetolactate synthase-like protein , GN=ILVBL	1	1	1	632	67.825	8.15
Q16222	UDP-N-acetylhexosamine pyrophosphorylase , GN=UAP1	1	1	1	522	58.732	6.33
Q8NHH9	Atlastin-2 , GN=ATL2	1	1	1	583	66.187	5.48
P32929	Cystathionine gamma-lyase , GN=CTH	1	1	1	405	44.479	6.7
Q99570	Phosphoinositide 3-kinase regulatory subunit 4 , GN=PIK3R4	1	1	1	1358	153.007	7.17
Q9UMX5	Neudesin , GN=NENF	1	1	1	172	18.845	5.69
Q9UPT9	Ubiquitin carboxyl-terminal hydrolase 22 , GN=USP22	1	1	1	525	59.921	8.05
Q96J84-2	Isoform 2 of Kin of IRRE-like protein 1 , GN=KIRREL1	1	1	1	773	84.997	5.73
Q9BPY3	Protein FAM118B , GN=FAM118B	1	1	1	351	39.474	5.99
Q8N5F7	NF-kappa-B-activating protein , GN=NKAP	1	1	1	415	47.11	10.11
P29590	Protein PML , GN=PML	1	1	1	882	97.489	6.21
O14683	Tumor protein p53-inducible protein 11 , GN=TP53I11	1	1	1	189	21.04	9.55
Q16650	T-box brain protein 1 , GN=TBR1	1	1	1	682	74.007	7.33
Q9BVP2	Guanine nucleotide-binding protein-like 3 , GN=GNL3	1	1	1	549	61.954	9.16
O75600-2	Isoform 2 of 2-amino-3-ketobutyrate coenzyme A ligase, mitochondrial , GN=GCAT	1	1	1	445	47.944	7.97
Q9NQT5	Exosome complex component RRP40 , GN=EXOSC3	1	1	1	275	29.553	8.1
Q9BUA3	Uncharacterized protein C11orf84 , GN=C11orf84	1	1	1	381	41.011	5.01
Q9BT23	LIM domain-containing protein 2 , GN=LIMD2	1	1	1	127	14.061	9.03
O95391	Pre-mRNA-splicing factor SLU7 , GN=SLU7	1	1	1	586	68.344	7.14
P62875	DNA-directed RNA polymerases I, II, and III subunit RPABC5 , GN=POLR2L	1	1	1	67	7.64	7.77
O75800	Zinc finger MYND domain-containing protein 10 , GN=ZMYND10	1	1	1	440	50.312	6.19
Q00534	Cyclin-dependent kinase 6 , GN=CDK6	1	1	1	326	36.915	6.46
Q96S82	Ubiquitin-like protein 7 , GN=UBL7	1	1	1	380	40.485	5.07
Q96HR8	H/ACA ribonucleoprotein complex non-core subunit NAF1 , GN=NAF1	1	1	1	494	53.683	4.87
P60903	Protein S100-A10 , GN=S100A10	1	1	1	97	11.196	7.37
Q15047	Histone-lysine N-methyltransferase SETDB1 , GN=SETDB1	1	1	1	1291	143.066	6.02
Q9P0J7	E3 ubiquitin-protein ligase KCMF1 ,	1	1	1	381	41.919	5.66
Q96JJ3	Engulfment and cell motility protein 2 , GN=ELMO2	1	1	1	720	82.562	5.9
Q9C0H9-5	Isoform 4 of SRC kinase signaling inhibitor 1 , GN=SRCIN1	1	1	1	1183	127.027	9.38
P20645	Cation-dependent mannose-6-phosphate receptor , GN=M6PR	1	1	1	277	30.973	5.83
P0CG08	Golgi pH regulator B , GN=GPR89B	1	1	1	455	52.882	9.28
Q7Z7H5	Transmembrane emp24 domain-containing protein 4 , GN=TMED4	1	1	1	227	25.926	8.28
Q9UPQ0	LIM and calponin homology domains-containing protein 1 , GN=LIMCH1	1	1	1	1083	121.792	6.47
Q66K64	DDB1- and CUL4-associated factor 15 , GN=DCAF15	1	1	1	600	66.421	6.58

Q9P2Q2	FERM domain-containing protein 4A , GN=FRMD4A	1	1	1	1039	115.387	8.87
Q15061	WD repeat-containing protein 43 ,	1	1	1	677	74.843	5.57
Q9Y3C4	EKC/KEOPS complex subunit TPRKB ,	1	1	1	175	19.648	6.79
Q92576	PHD finger protein 3 , GN=PHF3	1	1	1	2039	229.339	6.96
Q5VUA4	Zinc finger protein 318 , GN=ZNF318	1	1	1	2279	250.958	7.2
P39880-3	Isoform 3 of Homeobox protein cut-like 1 , GN=CUX1	1	1	1	1516	165.587	5.86
Q8N5I2	Arrestin domain-containing protein 1 , GN=ARRDC1	1	1	1	433	45.953	7.02
Q96SU4	Oxysterol-binding protein-related protein 9 , GN=OSBPL9	1	1	1	736	83.132	6.18
Q99496	E3 ubiquitin-protein ligase RING2 , GN=RNF2	1	1	1	336	37.632	6.84
O14662	Syntaxin-16 , GN=STX16	1	1	1	325	37.008	6.11
Q9HAZ2	PR domain zinc finger protein 16 ,	1	1	1	1276	140.162	6.21
Q9ULM3	YEATS domain-containing protein 2 ,	1	1	1	1422	150.689	8.98
Q15906-2	Isoform 2 of Vacuolar protein sorting-associated protein 72 homolog , GN=VPS72	1	1	1	375	41.822	6.67
Q13416	Origin recognition complex subunit 2 ,	1	1	1	577	65.931	6.51
Q96NT3-3	Isoform 3 of Protein GUCD1 , GN=GUCD1	1	1	1	295	33.455	8.72
Q9Y243	RAC-gamma serine/threonine-protein kinase , GN=AKT3	1	1	1	479	55.739	6.02
Q9NU22	Midasin , GN=MDN1	1	1	1	5596	632.42	5.68
O95359-3	Isoform 3 of Transforming acidic coiled-coil-containing protein 2 , GN=TACC2	1	1	1	2871	300.873	4.78
Q9NP92	39S ribosomal protein S30, mitochondrial , GN=MRPS30	1	1	1	439	50.333	7.97
O43281	Embryonal Fyn-associated substrate , GN=EFS	1	1	1	561	58.779	5.11
O00570	Transcription factor SOX-1 , GN=SOX1	1	1	1	391	38.998	9.7
Q03060	cAMP-responsive element modulator , GN=CREM	1	1	1	361	38.916	6.65
Q14678	KN motif and ankyrin repeat domain-containing protein 1 , GN=KANK1	1	1	1	1352	147.197	5.3
Q53FV1	ORM1-like protein 2 , GN=ORMDL2	1	1	1	153	17.352	9.64
O14795-2	Isoform 2 of Protein unc-13 homolog B , GN=UNC13B	1	1	1	1610	182.727	6.02
P98174	FYVE, RhoGEF and PH domain-containing protein 1 , GN=FGD1	1	1	1	961	106.494	6.58
Q8NEY1	Neuron navigator 1 , GN=NAV1	1	1	1	1877	202.347	8.07
O75146	Huntingtin-interacting protein 1-related protein , GN=HIP1R	1	1	1	1068	119.315	6.67
Q9NYJ1-2	Isoform 2 of Cytochrome c oxidase assembly factor 4 homolog, mitochondrial , GN=COA4	1	1	1	96	11.301	6.67
P55327-3	Isoform 3 of Tumor protein D52 , GN=TPD52	1	1	1	248	26.367	6.34
Q16773	Kynurenine--oxoglutarate transaminase 1 , GN=KYAT1	1	1	1	422	47.844	6.47
P55211	Caspase-9 , GN=CASP9	1	1	1	416	46.251	6.05
Q9BX40-2	Isoform 2 of Protein LSM14 homolog B , GN=LSM14B	1	1	1	385	41.995	9.39
Q9H3H9	Transcription elongation factor A protein-like 2 , GN=TCEAL2 PE=2	1	1	1	227	25.834	5.94
Q9UL12	Sarcosine dehydrogenase, mitochondrial , GN=SARDH	1	1	1	918	100.973	7.25
Q9H490	Phosphatidylinositol glycan anchor biosynthesis class U protein , GN=PIGU	1	1	1	435	50.019	7.72
Q9UNH6-3	Isoform 3 of Sorting nexin-7 , GN=SNX7	1	1	1	451	51.586	4.87
Q13480-2	Isoform 2 of GRB2-associated-binding protein 1 , GN=GAB1	1	1	1	724	79.955	6.38
Q9NZ52	ADP-ribosylation factor-binding protein GGA3 , GN=GGA3	1	1	1	723	78.267	5.58
Q8N9N7	Leucine-rich repeat-containing protein 57 , GN=LRRC57	1	1	1	239	26.737	8.43
P48380	Transcription factor RFX3 , GN=RFX3	1	1	1	749	83.477	6
Q9H6V9-2	Isoform 2 of Lipid droplet-associated hydrolase , GN=LDAH	1	1	1	331	37.965	6.68
Q9H8M2	Bromodomain-containing protein 9 ,	1	1	1	597	66.958	6.19
Q7Z5J4	Retinoic acid-induced protein 1 , GN=RAI1	1	1	1	1906	203.225	8.79

Q6EMK4	Vasorin , GN=VASN	1	1	1	673	71.668	7.39
O95999	B-cell lymphoma/leukemia 10 , GN=BCL10	1	1	1	233	26.235	5.74
Q70E73	Ras-associated and pleckstrin homology domains-containing protein 1 , GN=RAPH1	1	1	1	1250	135.171	8.85
Q9GZT4	Serine racemase , GN=SRR	1	1	1	340	36.543	6.54
Q8WXD2	Secretogranin-3 , GN=SCG3	1	1	1	468	52.973	5.03
Q9BSB4	Autophagy-related protein 101 , GN=ATG101	1	1	1	218	24.987	6.15
Q9GZY8	Mitochondrial fission factor , GN=MFF	1	1	1	342	38.441	8.95
Q9NSC5	Homer protein homolog 3 , GN=HOMER3	1	1	1	361	39.812	5.45
Q9H6S3-3	receptor kinase substrate 8-like protein 2 , GN=EPS8L2	1	1	1	731	82.271	7.03
Q9NVM4	Protein arginine N-methyltransferase 7 , GN=PRMT7	1	1	1	692	78.409	5.57
Q8IUF8	Ribosomal oxygenase 2 , GN=RIOX2	1	1	1	465	52.767	6.7
Q9HCS7	Pre-mRNA-splicing factor SYF1 , GN=XAB2	1	1	1	855	99.946	6.23
Q9UNL4	Inhibitor of growth protein 4 , GN=ING4	1	1	1	249	28.512	7.62
Q8WWX9	Selenoprotein M , GN=SELENOM	1	1	1	145	16.222	5.54
O94832	Unconventional myosin-Id , GN=MYO1D	1	1	1	1006	116.129	9.39
Q96Q42	Alsin , GN=ALS2	1	1	1	1657	183.518	6.27
O95980	Reversion-inducing cysteine-rich protein with Kazal motifs , GN=RECK	1	1	1	971	106.386	6.74
Q9BTE3	Mini-chromosome maintenance complex-binding protein , GN=MCMBP	1	1	1	642	72.934	5.87
O00743-3	Isoform 3 of Serine/threonine-protein phosphatase 6 catalytic subunit , GN=PPP6C	1	1	1	342	38.921	6.18
Q96IU4	Protein ABHD14B , GN=ABHD14B	1	1	1	210	22.332	6.4
Q96DV4	39S ribosomal protein L38, mitochondrial , GN=MRPL38	1	1	1	380	44.568	7.53
P22415	Upstream stimulatory factor 1 , GN=USF1	1	1	1	310	33.518	5.54
Q9NP74	Palmdelphin , GN=PALMD	1	1	1	551	62.72	5.44
Q14241	Elongin-A , GN=ELOA	1	1	1	798	89.853	9.57
Q9Y5P6-2	Isoform 2 of Mannose-1-phosphate guanyltransferase beta , GN=GMPPB	1	1	1	387	42.594	6.29
Q969X5	Endoplasmic reticulum-Golgi intermediate compartment protein 1 , GN=ERGIC1	1	1	1	290	32.571	7.06
Q9UKS6	Protein kinase C and casein kinase substrate in neurons protein 3 , GN=PACSIN3	1	1	1	424	48.457	6.18
Q9H6R3	Acyl-CoA synthetase short-chain family member 3, mitochondrial , GN=ACSS3	1	1	1	686	74.73	8.63
Q9P0J0-2	[ubiquinone] 1 alpha subcomplex subunit 13 , GN=NDUFA13	1	1	1	222	24.866	9.7
Q674X7	Kazrin , GN=KAZN	1	1	1	775	86.298	7.03
Q5ZPR3	CD276 antigen , GN=CD276	1	1	1	534	57.199	4.91
Q15911	Zinc finger homeobox protein 3 , GN=ZFHX3	1	1	1	3703	404.165	6.2
Q13449	Limbic system-associated membrane protein , GN=LSAMP	1	1	1	338	37.37	6.98
Q13526	Peptidyl-prolyl cis-trans isomerase NIMA-interacting 1 , GN=PIN1	1	1	1	163	18.232	8.82
Q9HAF1-3	Isoform 3 of Chromatin modification-related protein MEAF6 , GN=MEAF6	1	1	1	201	22.741	8.28
O75446	Histone deacetylase complex subunit SAP30 , GN=SAP30	1	1	1	220	23.292	9.17
Q8WXE9-3	Isoform 2 of Stonin-2 , GN=STON2	1	1	1	920	102.77	5.5
Q9H6E4	Coiled-coil domain-containing protein 134 , GN=CCDC134	1	1	1	229	26.544	8.85
Q96JG8-4	Isoform 4 of Melanoma-associated antigen D4 , GN=MAGED4	1	1	1	757	83.165	6.57
Q9ULT0-4	Isoform 3 of Tetratricopeptide repeat protein 7A , GN=TTC7A	1	1	1	882	98.875	6.46
Q92544	Transmembrane 9 superfamily member 4 , GN=TM9SF4	1	1	1	642	74.47	6.54
Q86V88	Magnesium-dependent phosphatase 1 , GN=MDP1	1	1	1	176	20.096	6.39
Q9UBP4	Dickkopf-related protein 3 , GN=DKK3	1	1	1	350	38.365	4.65
Q8N3K9	Cardiomyopathy-associated protein 5 , GN=CMYA5	1	1	1	4069	448.935	4.78

Q96DX7	Tripartite motif-containing protein 44 , GN=TRIM44	1	1	1	344		38.448	4.21	
Q6MZM0	Hephaestin-like protein 1 , GN=HEPHL1 PE=2	1	1	1	1159		131.519	6.74	
P10746	Uroporphyrinogen-III synthase , GN=UROS	1	1	1	265	28.61		5.48	
Q9BXU1	Serine/threonine-protein kinase 31 , GN=STK31 PE=2	1	1	1	1019		115.621	5.14	
Q14161	ARF GTPase-activating protein GIT2 ,	1	1	1	759	84.49		7.23	
P49406	39S ribosomal protein L19, mitochondrial , GN=MRPL19	1	1	1	292		33.514	9.5	
Q5PRF9	Protein Smaug homolog 2 , GN=SAMD4B	1	1	1	694		75.436	6.83	
P25686	DnaJ homolog subfamily B member 2 , GN=DNAJB2	1	1	1	324		35.558	5.95	
O75093	Slit homolog 1 protein , GN=SLIT1 PE=2	1	1	1	1534		167.816	6.57	
Q96GS4	BLOC-1-related complex subunit 6 ,	1	1	1	357		37.204	5.76	
Q969S9	Ribosome-releasing factor 2, mitochondrial , GN=GFM2	1	1	1	779		86.546	6.51	
P49789	Bis(5'-adenosyl)-triphosphatase , GN=FHIT	1	1	1	147		16.848	7.08	
O15504	Nucleoporin-like protein 2 , GN=NUPL2	1	1	1	423		44.844	9.19	
P17706	Tyrosine-protein phosphatase non-receptor type 2 , GN=PTPN2	1	1	1	415		48.442	8.29	
O95260	Arginyl-tRNA--protein transferase 1 ,	1	1	1	518		59.052	7.93	
Q08AE8	Protein spire homolog 1 , GN=SPIRE1	1	1	1	756		85.489	8.62	
Q9Y3D0	Mitotic spindle-associated MMXD complex subunit MIP18 , GN=FAM96B	1	1	1	163		17.652	5.19	
P34741	Syndecan-2 , GN=SDC2	1	1	1	201		22.146	4.86	
A6NIE6	Putative RRN3-like protein RRN3P2 , GN=RRN3P2 PE=5	1	1	1	340	38.01		5.17	
Q14517	Protocadherin Fat 1 , GN=FAT1	1	1	1	4588		505.958		5
P16401	Histone H1.5 , GN=HIST1H1B	1	1	1	226		22.566	10.92	
Q9UHQ9	NADH-cytochrome b5 reductase 1 ,	1	1	1	305		34.073	9.38	
Q9Y232	Chromodomain Y-like protein , GN=CDYL	1	1	1	598	66.44		9.45	
Q6PRD1	Probable G-protein coupled receptor 179 , GN=GPR179	1	1	1	2367		257.202	5.71	
Q5SQN1	Synaptosomal-associated protein 47 , GN=SNAP47	1	1	1	464		52.529	8.69	
Q96RE7	Nucleus accumbens-associated protein 1 , GN=NACC1	1	1	1	527		57.222	5.74	
Q9P1Z2	Calcium-binding and coiled-coil domain-containing protein 1 , GN=CALCOCO1	1	1	1	691		77.289	4.82	
Q9Y6M7-7	Isoform 7 of Sodium bicarbonate cotransporter 3 , GN=SLC4A7	1	1	1	1259		140.677	6.62	
Q969X6	U3 small nucleolar RNA-associated protein 4 homolog , GN=UTP4	1	1	1	686		76.841	8.85	
O43314	Inositol hexakisphosphate and diphosphoinositol-pentakisphosphate kinase 2 , GN=PPIP5K2	1	1	1	1243		140.318	8.22	
Q9NS73	MAP3K12-binding inhibitory protein 1 , GN=MBIP	1	1	1	344		39.257	7.24	
Q4KMQ2-2	Isoform 2 of Anoctamin-6 , GN=ANO6	1	1	1	931		108.362	7.87	
Q9UGR2	Zinc finger CCCH domain-containing protein 7B , GN=ZC3H7B	1	1	1	993		111.506	7.17	
Q8IW45-2	Isoform 2 of ATP-dependent (S)-NAD(P)H-hydrate dehydratase , GN=NAXD	1	1	1	390		41.335	9.1	
O75575	DNA-directed RNA polymerase III subunit RPC9 , GN=CRCP	1	1	1	148		16.861	5.39	
Q9C0E8-4	Isoform 4 of Endoplasmic reticulum junction formation protein lunapark , GN=LNPK	1	1	1	459		50.817	5.08	
Q96GX9	Methylthioribulose-1-phosphate dehydratase , GN=APIP	1	1	1	242		27.107	7.12	
Q5TIE3	von Willebrand factor A domain-containing protein 5B1 , GN=VWA5B1	1	1	1	1220		133.718	8.15	
Q9BVJ6	U3 small nucleolar RNA-associated protein 14 homolog A , GN=UTP14A	1	1	1	771		87.924	7.87	
Q8N122	Regulatory-associated protein of mTOR , GN=RPTOR	1	1	1	1335		148.942	6.89	
Q9H9S4	Calcium-binding protein 39-like , GN=CAB39L	1	1	1	337		39.063	8.4	
Q99418	Cytohesin-2 , GN=CYTH2	1	1	1	400		46.517	5.54	

Q92990	Glomulin , GN=GLMN	1	1	1	594	68.165	5.33
Q6IQ23-2	Isoform 2 of Pleckstrin homology domain-containing family A member 7 , GN=PLEKHA7	1	1	1	1122	127.184	9.35
P19838-2	Isoform 2 of Nuclear factor NF-kappa-B p105 subunit , GN=NFKB1	1	1	1	969	105.361	5.4
Q99541	Perilipin-2 , GN=PLIN2	1	1	1	437	48.045	6.8
Q63ZY3-2	Isoform 2 of KN motif and ankyrin repeat domain-containing protein 2 , GN=KANK2	1	1	1	859	91.864	5.63
P09228	Cystatin-SA , GN=CST2	1	1	1	141	16.434	4.93
Q99704	Docking protein 1 , GN=DOK1	1	1	1	481	52.359	6.47
Q8NEN9	PDZ domain-containing protein 8 , GN=PDZD8	1	1	1	1154	128.483	6.09
Q8IX15-3	Isoform 2 of Homeobox and leucine zipper protein Homez , GN=HOMEZ	1	1	1	552	61.657	5.21
Q8TDY8	Immunoglobulin superfamily DCC subclass member 4 , GN=IGDCC4	1	1	1	1250	134.126	6.2
Q96RS6	NudC domain-containing protein 1 , GN=NUDCD1	1	1	1	583	66.713	5.11
Q5HYK3	2-methoxy-6-polyprenyl-1,4-benzoquinol methylase, mitochondrial , GN=COQ5	1	1	1	327	37.117	6.95
Q5JS37	NHL repeat-containing protein 3 , GN=NHLRC3 PE=2	1	1	1	347	38.258	6.43
Q0GBX7	Matrix protein OS=Rabies virus (strain China/DRV) GN=M PE=3	1	1	1	202	23.315	6.55
Q9HC44	Vasculin-like protein 1 , GN=GPBP1L1	1	1	1	474	52.269	6.84
Q9H098-2	Isoform 2 of Protein FAM107B ,	1	1	1	306	34.738	7.01
P10415	Apoptosis regulator Bcl-2 , GN=BCL2	1	1	1	239	26.249	7.28
Q6Y288	Beta-1,3-glucosyltransferase , GN=B3GLCT	1	1	1	498	56.528	7.5
Q86VP3-2	Isoform 2 of Phosphofurin acidic cluster sorting protein 2 , GN=PACS2	1	1	1	904	99.566	6.8
Q9Y4I1	Unconventional myosin-Va , GN=MYO5A	1	1	1	1855	215.269	8.48
Q9UQ26	Regulating synaptic membrane exocytosis protein 2 , GN=RIMS2	1	1	1	1411	160.303	9.07
O95169	NADH dehydrogenase [ubiquinone] 1 beta subcomplex subunit 8, mitochondrial , GN=NDUFB8	1	1	1	186	21.751	6.8
Q969Z0	Protein TBRG4 , GN=TBRG4	1	1	1	631	70.693	7.42
Q9NWA0	Mediator of RNA polymerase II transcription subunit 9 , GN=MED9	1	1	1	146	16.392	7.39
Q9NW13	RNA-binding protein 28 , GN=RBM28	1	1	1	759	85.685	9.22
Q12788	Transducin beta-like protein 3 , GN=TBL3	1	1	1	808	88.978	6.9
Q9H4L7-2	Isoform 2 of SWI/SNF-related matrix-associated actin-dependent regulator of chromatin subfamily A containing DEAD/H box 1 , GN=SMARCAD1	1	1	1	1028	117.528	5.55
Q8IWD4	Coiled-coil domain-containing protein 117 , GN=CCDC117	1	1	1	279	30.522	5.07
A8CG34	Nuclear envelope pore membrane protein POM 121C , GN=POM121C	1	1	1	1229	124.982	10.37
Q8ND04-2	Isoform 2 of Protein SMG8 , GN=SMG8	1	1	1	1023	113.259	7.68
Q9UQ13	Leucine-rich repeat protein SHOC-2 ,	1	1	1	582	64.847	8.46
O60936-3	Isoform 3 of Nucleolar protein 3 , GN=NOL3	1	1	1	270	29.337	4.3
Q9NR50	Translation initiation factor eIF-2B subunit gamma , GN=EIF2B3	1	1	1	452	50.208	6.47
Q8IYW4	ENTH domain-containing protein 1 , GN=ENTHD1 PE=2	1	1	1	607	67.496	6.62
Q92520	Protein FAM3C , GN=FAM3C	1	1	1	227	24.665	8.29
Q9H0Q3	FXYD domain-containing ion transport regulator 6 , GN=FXYD6	1	1	1	95	10.535	5.1
O75787	Renin receptor , GN=ATP6AP2	1	1	1	350	38.983	6.1
Q17R31-3	Isoform 3 of Putative deoxyribonuclease TATDN3 , GN=TATDN3	1	1	1	281	31.103	7.5
Q96BW9	Phosphatidate cytidylyltransferase, mitochondrial , GN=TAMM41	1	1	1	452	51.034	7.94
Q01459	Di-N-acetylchitobiase , GN=CTBS	1	1	1	385	43.732	6.64
Q0PDM0	Non-structural protein 1 OS=Influenza A virus (strain A/X-31 H3N2) GN=NS PE=3	1	1	1	230	25.832	7.97
O15269	Serine palmitoyltransferase 1 , GN=SPTLC1	1	1	1	473	52.71	6.01

P27909	Genome polyprotein OS=Dengue virus type 1 (strain Brazil/97-11/1997)	1	1	1	3392		378.661	8.31
P04424	Argininosuccinate lyase , GN=ASL	1	1	1	464		51.625	6.48
P29400-2	Isoform 2 of Collagen alpha-5(IV) chain , GN=COL4A5	1	1	1	1691	161.53		7.62
Q86VR2	Reticulophagy regulator 3 , GN=RETREG3	1	1	1	466		51.364	4.97
P16749	OS=Human cytomegalovirus (strain AD169) GN=UL69	1	1	1	744		82.626	6.76
Q96RG2-2	Isoform 2 of PAS domain-containing serine/threonine-protein kinase , GN=PASK	1	1	1	1330		143.593	4.88
Q5SSJ5	Heterochromatin protein 1-binding protein 3 , GN=HP1BP3	1	1	1	553		61.169	9.67
P08253	72 kDa type IV collagenase , GN=MMP2	1	1	1	660		73.835	5.47
Q6PII5-2	Isoform 2 of Hydroxyacylglutathione hydrolase-like protein , GN=HAGHL	1	1	1	282		31.214	6.14
Q86YH6	Decaprenyl-diphosphate synthase subunit 2 , GN=PDSS2	1	1	1	399		44.101	8.24
Q05048	Cleavage stimulation factor subunit 1 , GN=CSTF1	1	1	1	431		48.327	6.58
Q08378	Golgin subfamily A member 3 , GN=GOLGA3	1	1	1	1498		167.252	5.44
O14578-4	Isoform 4 of Citron Rho-interacting kinase , GN=CIT	1	1	1	2069		236.459	6.67
O75844	CAAX prenyl protease 1 homolog ,	1	1	1	475		54.778	7.49
Q9NS86	LanC-like protein 2 , GN=LANCL2	1	1	1	450		50.821	7.43
Q8N543	Prolyl 3-hydroxylase OGFOD1 , GN=OGFOD1	1	1	1	542		63.206	5.11
Q4J6C6	Prolyl endopeptidase-like , GN=PREPL	1	1	1	727		83.873	6.38
P16885	1-phosphatidylinositol 4,5-bisphosphate phosphodiesterase gamma-2 , GN=PLCG2	1	1	1	1265		147.776	6.64
P23919	Thymidylate kinase , GN=DTYMK	1	1	1	212		23.804	8.27
P56524	Histone deacetylase 4 , GN=HDAC4	1	1	1	1084		118.966	6.96
Q08752	Peptidyl-prolyl cis-trans isomerase D ,	1	1	1	370		40.738	7.21
Q9NW68-3	Isoform 3 of BSD domain-containing protein 1 , GN=BSDC1	1	1	1	474		51.135	4.55
Q9Y294	Histone chaperone ASF1A , GN=ASF1A	1	1	1	204		22.954	4.41
Q9Y6X8	Zinc fingers and homeoboxes protein 2 , GN=ZHX2	1	1	1	837	92.25		6.86
Q6ZNH5	Zinc finger protein 497 , GN=ZNF497	1	1	1	498		54.686	8.94
Q13188-2	Isoform 2 of Serine/threonine-protein kinase 3 , GN=STK3	1	1	1	519		59.424	5.25
Q8N1B4	Vacuolar protein sorting-associated protein 52 homolog , GN=VPS52	1	1	1	723	82.17		5.99
P23468	Receptor-type tyrosine-protein phosphatase delta , GN=PTPRD	1	1	1	1912		214.625	6.57
Q96I51	RCC1-like G exchanging factor-like protein , GN=RCC1L	1	1	1	464		49.965	8.4
P48200	Iron-responsive element-binding protein 2 , GN=IREB2	1	1	1	963		104.978	7.05
Q5T160	Probable arginine--tRNA ligase, mitochondrial , GN=RARS2	1	1	1	578		65.464	8.21
Q86WR0	Coiled-coil domain-containing protein 25 , GN=CCDC25	1	1	1	208		24.463	6.8
Q9UEW8	STE20/SPS1-related proline-alanine-rich protein kinase , GN=STK39	1	1	1	545		59.436	6.29
O94829	Importin-13 , GN=IPO13	1	1	1	963		108.126	5.3
Q9Y385	Ubiquitin-conjugating enzyme E2 J1 ,	1	1	1	318		35.177	6.74
Q5T447	E3 ubiquitin-protein ligase HECTD3 ,	1	1	1	861		97.051	5.64
P0CE71	Putative oncomodulin-2 , GN=OCM2 PE=5	1	1	1	109		12.122	4.27
P15559	NAD(P)H dehydrogenase [quinone] 1 ,	1	1	1	274		30.848	8.88
Q9Y639	Neuroplastin , GN=NPTN	1	1	1	398	44.36		7.99
P21359	Neurofibromin , GN=NF1	1	1	1	2839		319.168	7.39
Q9HD47	Ran guanine nucleotide release factor , GN=RANGRF	1	1	1	186		20.435	4.94
Q9P241	Probable phospholipid-transporting ATPase VD , GN=ATP10D PE=2	1	1	1	1426		160.171	7.15
P86397	Hydroxyacyl-thioester dehydratase type 2, mitochondrial , GN=HTD2	1	1	1	168		18.547	8.92
P09525	Annexin A4 , GN=ANXA4	1	1	1	319	35.86		6.13

Q5VWZ2	Lysophospholipase-like protein 1 ,	1	1	1	237	26.299	7.84
P10109	Adrenodoxin, mitochondrial , GN=FDX1	1	1	1	184	19.381	5.83
Q9UIF8	Bromodomain adjacent to zinc finger domain protein 2B , GN=BAZ2B	1	1	1	2168	240.311	6.54
Q9H4Z3	Phosphorylated CTD-interacting factor 1 , GN=PCIF1	1	1	1	704	80.618	7.42
P30499	HLA class I histocompatibility antigen, Cw-1 alpha chain , GN=HLA-C	1	1	1	366	40.939	5.71
Q14CX7	N-alpha-acetyltransferase 25, NatB auxiliary subunit , GN=NAA25	1	1	1	972	112.221	6.64
Q16760	Diacylglycerol kinase delta , GN=DGKD	1	1	1	1214	134.44	7.56
Q9NYZ3	G2 and S phase-expressed protein 1 ,	1	1	1	720	76.598	9.39
O00584	Ribonuclease T2 , GN=RNASET2	1	1	1	256	29.462	7.08
A3FMB2	Genome polyprotein OS=Human hepatitis A virus genotype IA (isolate H2) PE=3	1	2	1	2227	251.237	6.52
O43312-5	Isoform 4 of Metastasis suppressor protein 1 , GN=MTSS1	1	1	1	759	82.628	6.81
Q8IW50-6	Isoform 6 of Protein FAM219A ,	1	1	1	168	18.545	4.64
P62487	DNA-directed RNA polymerase II subunit RPB7 , GN=POLR2G	1	1	1	172	19.282	5.54
Q9BRR8	G patch domain-containing protein 1 , GN=GPATCH1	1	1	1	931	103.282	7.05
Q8IWU5	Extracellular sulfatase Sulf-2 , GN=SULF2	1	1	1	870	100.39	9.17
Q96FX7	tRNA (adenine(58)-N(1))-methyltransferase catalytic subunit TRMT61A , GN=TRMT61A	1	1	1	289	31.362	7.36
O14933	Ubiquitin/ISG15-conjugating enzyme E2 L6 , GN=UBE2L6	1	1	1	153	17.757	7.88
Q9UMS0	NFU1 iron-sulfur cluster scaffold homolog, mitochondrial , GN=NFU1	1	1	1	254	28.444	5.07
Q6P6C2-1	Isoform 1 of RNA demethylase ALKBH5 , GN=ALKBH5	1	1	1	467	52.222	8.51
Q96L46	Calpain small subunit 2 , GN=CAPNS2 PE=2	1	1	1	248	27.643	5.73
Q9Y3U8	60S ribosomal protein L36 , GN=RPL36	1	1	1	105	12.246	11.59
A6NM28	Zinc finger protein 92 homolog , GN=ZFP92	1	1	1	416	45.762	10.2
Q13591	Semaphorin-5A , GN=SEMA5A	1	1	1	1074	120.537	7.21
Q9UKU7	Isobutyryl-CoA dehydrogenase, mitochondrial , GN=ACAD8	1	1	1	415	45.04	7.85
P22304	Iduronate 2-sulfatase , GN=IDS	1	1	1	550	61.834	5.45
Q86T03-2	Isoform 2 of Type 1 phosphatidylinositol 4,5-bisphosphate 4-phosphatase , GN=PIP4P1	1	1	1	284	30.165	8.98
Q6ZP68	Putative protein ATP11AUN , GN=ATP11AUN PE=2	1	1	1	121	13.408	8.35
Q0VAK6	Leiomodin-3 , GN=LMOD3	1	1	1	560	64.874	5.54
Q9ULJ3	Zinc finger and BTB domain-containing protein 21 , GN=ZBTB21	1	1	1	1066	118.796	8.29
P29353-6	Isoform 6 of SHC-transforming protein 1 , GN=SHC1	1	1	1	584	62.854	6.44
Q969V6	MKL/myocardin-like protein 1 , GN=MKL1	1	1	1	931	98.858	5.86
Q9Y2G4-1	Isoform 2 of Ankyrin repeat domain-containing protein 6 , GN=ANKRD6	1	1	1	692	75.69	8.97
Q13164	Mitogen-activated protein kinase 7 ,	1	1	1	816	88.331	5.88
Q9HCE6	Rho guanine nucleotide exchange factor 10-like protein , GN=ARHGEF10L	1	1	1	1279	140.321	5.88
Q9NTJ5	Phosphatidylinositide phosphatase SAC1 , GN=SACM1L	1	1	1	587	66.924	7.12
Q96S52	GPI transamidase component PIG-S ,	1	1	1	555	61.617	6.49
Q9Y2D5-4	Isoform 3 of A-kinase anchor protein 2 , GN=AKAP2	1	1	1	1103	121.997	5.06
Q9Y6X9	MORC family CW-type zinc finger protein 2 , GN=MORC2	1	1	1	1032	117.75	8.38
Q9H2X9	Solute carrier family 12 member 5 ,	1	1	1	1139	126.102	6.73
P36404	ADP-ribosylation factor-like protein 2 ,	1	1	1	184	20.865	6.34
Q9UK58	Cyclin-L1 , GN=CCNL1	1	1	1	526	59.597	10.7
Q9Y6W6	Dual specificity protein phosphatase 10 , GN=DUSP10	1	1	1	482	52.609	7.74
Q9UN71	Protocadherin gamma-B4 , GN=PCDHGB4	1	1	1	923	99.865	4.93

Q9H094	Neuroblastoma breakpoint family member 3 , GN=NBPF3 PE=2	1	1	1	633	72.906	4.5
Q8NI77	Kinesin-like protein KIF18A , GN=KIF18A	1	1	1	898	102.216	8.91
P55157	Microsomal triglyceride transfer protein large subunit , GN=MTTP	1	1	1	894	99.289	8.41
Q14244-7	Isoform 7 of Ensconsin , GN=MAP7	1	1	1	771	86.9	9.77
Q9BXF3	Cat eye syndrome critical region protein 2 , GN=CECR2	1	1	1	1484	164.109	6.98
P27153	RNA-directed RNA polymerase catalytic subunit OS=Dhori virus (strain Indian/1313/61) GN=P2 PE=3	1	1	1	716	81.24	6.27
Q96FC7	Phytanoyl-CoA hydroxylase-interacting protein-like , GN=PHYHIPL	1	1	1	376	42.459	6.42
Q99767	Amyloid-beta A4 precursor protein-binding family A member 2 , GN=APBA2	1	1	1	749	82.46	4.86
O15013	Rho guanine nucleotide exchange factor 10 , GN=ARHGEF10	1	1	1	1369	151.516	5.68
P10301	Ras-related protein R-Ras , GN=RRAS	1	1	1	218	23.466	6.93
Q9ULK5	Vang-like protein 2 , GN=VANGL2	1	1	1	521	59.677	9.22
Q8TCA0	Leucine-rich repeat-containing protein 20 , GN=LRRC20	1	1	1	184	20.496	6.55
Q9Y2P7	Zinc finger protein 256 , GN=ZNF256	1	1	1	627	71.825	8.81
Q9H8H3	Methyltransferase-like protein 7A , GN=METTL7A	1	1	1	244	28.301	8.38
Q06587	E3 ubiquitin-protein ligase RING1 , GN=RING1	1	1	1	406	42.403	5.62
Q13491-4	Isoform 4 of Neuronal membrane glycoprotein M6-b , GN=GPM6B	1	1	1	328	36.196	6.09
O75343	Guanylate cyclase soluble subunit beta-2 , GN=GUCY1B2 PE=5	1	1	1	617	70.322	8.63
Q68G74	LIM/homeobox protein Lhx8 , GN=LHX8	1	1	1	356	39.275	8.22
Q9NR11	Zinc finger protein 302 , GN=ZNF302	1	1	1	478	54.778	8.91
Q5BJF6-7	Isoform 7 of Outer dense fiber protein 2 , GN=ODF2	1	1	1	701	80.835	8.66
Q9UKN7	Unconventional myosin-XV , GN=MYO15A	1	1	1	3530	395.044	9.17
Q3KSU8	Large tegument protein deneddylase OS=Epstein-Barr virus (strain GD1) GN=BPLF1 PE=3	1	1	1	3176	340.395	5.68
Q8NDH6	Islet cell autoantigen 1-like protein ,	1	1	1	482	54.373	5.33
P18099	Protein Vpx OS=Human immunodeficiency virus type 2 subtype A (isolate BEN) GN=vpx	1	1	1	113	13.199	5.49
Q9NP77	RNA polymerase II subunit A C-terminal domain phosphatase SSU72 , GN=SSU72	1	1	1	194	22.56	5.33
O95229	ZW10 interactor , GN=ZWINT	1	1	1	277	31.274	5.15
Q9H7R0	Zinc finger protein 442 , GN=ZNF442 PE=2	1	1	1	627	72.816	8.7
Q7Z7F0	Protein BLOM7 , GN=KIAA0907	1	1	1	614	64.805	8.73
Q8IX06	Putative exonuclease GOR , GN=REXO1L1P	1	1	1	675	73.808	9.14
P07307	Asialoglycoprotein receptor 2 , GN=ASGR2	1	1	1	311	35.07	6.25
O60566-3	Isoform 3 of Mitotic checkpoint serine/threonine-protein kinase BUB1 beta , GN=BUB1B	1	1	1	1064	121.31	5.36
O60493	Sorting nexin-3 , GN=SNX3	1	1	1	162	18.751	8.66
P38570	Integrin alpha-E , GN=ITGAE	1	1	1	1179	130.077	5.74
A6NMZ7	Collagen alpha-6(VI) chain , GN=COL6A6	1	1	1	2263	247.019	6.89
Q9UK45	U6 snRNA-associated Sm-like protein LSm7 , GN=LSM7	1	1	1	103	11.595	5.27
Q9Y5Q9	General transcription factor 3C polypeptide 3 , GN=GTF3C3	1	1	1	886	101.208	5.07
P15918	V(D)J recombination-activating protein 1 , GN=RAG1	1	1	1	1043	119.02	8.68
Q9H7B4	Histone-lysine N-methyltransferase SMYD3 , GN=SMYD3	1	1	1	428	49.065	7.25
Q96PQ0	VPS10 domain-containing receptor SorCS2 , GN=SORCS2	1	1	1	1159	128.071	7.08
Q3MHD2-2	Isoform 2 of Protein LSM12 homolog ,	1	1	1	198	21.985	6.9
Q9NPC7	Myoneurin , GN=MYNN	1	1	1	610	68.638	8.25
A6NDG6	Glycerol-3-phosphate phosphatase , GN=PGP	1	1	1	321	33.985	6.14

P0C2W1	F-box/SPRY domain-containing protein 1 , GN=FBXO45	1	1	1	286	30.613	7.83
Q86WB0	Nuclear-interacting partner of ALK ,	1	1	1	502	55.226	5.62
Q9P016	Thymocyte nuclear protein 1 , GN=THYN1	1	1	1	225	25.681	9.25
P52362	Large tegument protein deneddylase OS=Human herpesvirus 7 (strain JI) GN=U31 PE=3	1	1	1	2059	239.327	6.93
Q4G0T1	Scavenger receptor cysteine-rich domain-containing protein SCART1 ,	1	1	1	1027	108.541	6.15
Q99592-2	Isoform 2 of Zinc finger and BTB domain-containing protein 18 , GN=ZBTB18	1	1	1	531	59.328	5.63
Q9Y6G5	COMM domain-containing protein 10 , GN=COMMD10	1	1	1	202	22.952	6.54
O75691	Small subunit processome component 20 homolog , GN=UTP20	1	1	1	2785	318.182	7.39
Q9NYJ8	TGF-beta-activated kinase 1 and MAP3K7-binding protein 2 , GN=TAB2	1	1	1	693	76.447	8.54
Q14232	Translation initiation factor eIF-2B subunit alpha , GN=EIF2B1	1	1	1	305	33.691	7.33
Q8NFW8	N-acylneuraminate cytidylyltransferase , GN=CMAS	1	1	1	434	48.349	7.93
Q8NAP8-2	Isoform 2 of Zinc finger and BTB domain-containing protein 8B , GN=ZBTB8B	1	1	1	512	55.527	5.41
P49639	Homeobox protein Hox-A1 , GN=HOXA1	1	1	1	335	36.618	8.05
Q9Y3P4	Rhomboid domain-containing protein 3 , GN=RHBDD3 PE=2	1	1	1	386	40.458	6.58
Q9ULC3	Ras-related protein Rab-23 , GN=RAB23	1	1	1	237	26.643	6.6
ENSEMBL:EN	(Bos taurus) 63 kDa protein	1	1	1	606	63.127	8.46
Q9BRV8	Suppressor of IKBKE 1 , GN=SIKE1	1	1	1	207	23.706	5.21
O95409	Zinc finger protein ZIC 2 , GN=ZIC2	1	1	1	532	54.971	8.41
Q15303	Receptor tyrosine-protein kinase erbB-4 , GN=ERBB4	1	1	1	1308	146.713	6.39
Q9H492-2	Isoform 2 of Microtubule-associated proteins 1A/1B light chain 3A , GN=MAP1LC3A	1	1	1	125	14.483	7.99
Q9BYP7	Serine/threonine-protein kinase WNK3 , GN=WNK3	1	1	1	1800	198.292	6.07
Q9C0G0	Zinc finger protein 407 , GN=ZNF407	1	1	1	2248	247.211	6.49
Q75N90	Fibrillin-3 , GN=FBN3 PE=2	1	1	1	2809	300.149	5.07
A4D1P6	WD repeat-containing protein 91 ,	1	1	1	747	83.292	6.58
Q9P0P0	E3 ubiquitin-protein ligase RNF181 ,	1	1	1	153	17.897	5.06
Q17RR3	Pancreatic lipase-related protein 3 , GN=PNLIPRP3 PE=2	1	1	1	467	52.22	8.27
Q9NSV4	Protein diaphanous homolog 3 , GN=DIAPH3	1	1	1	1193	136.839	7.03
Q79666	Gag-Pol polyprotein OS=Human immunodeficiency virus type 1 group O (isolate MVP5180) GN=gag-pol PE=3	1	1	1	1446	162.668	8.12
Q9H269	Vacuolar protein sorting-associated protein 16 homolog , GN=VPS16	1	1	1	839	94.634	6.77
Q03053	Genome polyprotein OS=Coxsackievirus B5 (strain Peterborough / 1954/UK/85) PE=3	1	1	1	2185	243.143	6.8
Q7Z7B0	Filamin-A-interacting protein 1 , GN=FILIP1	1	1	1	1213	138.024	8.32
Q96RT7	Gamma-tubulin complex component 6 , GN=TUBGCP6	1	1	1	1819	200.372	6.32
Q8IZP6	RING finger protein 113B , GN=RNF113B	1	1	1	322	36.236	7.59
O15085-2	Isoform 2 of Rho guanine nucleotide exchange factor 11 , GN=ARHGEF11	1	1	1	1562	172.137	5.54
P19320	Vascular cell adhesion protein 1 , GN=VCAM1	1	1	1	739	81.224	5.22
O14893	Gem-associated protein 2 , GN=GEMIN2	1	1	1	280	31.565	5.58
Q9UPN7	Serine/threonine-protein phosphatase 6 regulatory subunit 1 , GN=PPP6R1	1	1	1	881	96.664	4.55
Q9H9B1	Histone-lysine N-methyltransferase EHMT1 , GN=EHMT1	1	1	1	1298	141.377	5.76
Q9BQQ3	Golgi reassembly-stacking protein 1 , GN=GORASP1	1	1	1	440	46.454	4.5
Q7Z570	Zinc finger protein 804A , GN=ZNF804A PE=2	1	1	1	1209	136.802	7.94
Q99720	Sigma non-opioid intracellular receptor 1 , GN=SIGMAR1	1	1	1	223	25.112	5.96

Q9ULE3	DENN domain-containing protein 2A , GN=DENND2A PE=2	1	1	1	1009		113.782	8.95
P57679	Ellis-van Creveld syndrome protein , GN=EVC	1	1	1	992	111.92		6.73
Q96EY1	DnaJ homolog subfamily A member 3, mitochondrial , GN=DNAJA3	1	1	1	480		52.456	9.26
Q96SN8	CDK5 regulatory subunit-associated protein 2 , GN=CDK5RAP2	1	1	1	1893		214.905	5.58
P36738	Major capsid protein L1 OS=Human papillomavirus type 34 GN=L1 PE=3	1	1	1	528	59.01		8.27
Q14527	Helicase-like transcription factor , GN=HLTF	1	1	1	1009		113.857	8.6
Q13905-3	Isoform 3 of Rap guanine nucleotide exchange factor 1 , GN=RAPGEF1	1	1	1	1095		122.659	6.25
Q9Y2K6	Ubiquitin carboxyl-terminal hydrolase 20 , GN=USP20	1	1	1	914		101.938	6.11
Q9NPG4	Protocadherin-12 , GN=PCDH12	1	1	1	1184		128.915	5.29
O75420	GRB10-interacting GYF protein 1 , GN=GIGYF1	1	1	1	1035		114.531	5.39
Q86YL5-2	Isoform 2 of Testis development-related protein , GN=TDRP	1	1	1	198		22.271	8.24
Q9ULS6	Potassium voltage-gated channel subfamily S member 2 , GN=KCNS2	1	1	1	477		54.203	5.86
Q8NCV1-2	Isoform 2 of Adenosine deaminase domain-containing protein 2 , GN=ADAD2	1	1	1	665		70.944	8.69
O60508	Pre-mRNA-processing factor 17 , GN=CDC40	1	1	1	579		65.479	7.06
P50750-2	Isoform 2 of Cyclin-dependent kinase 9 , GN=CDK9	1	1	1	489		53.331	9.48
Q9P2K9	Protein dispatched homolog 3 , GN=DISP3	1	1	1	1392	152.95		7.55
Q9P0W2	SWI/SNF-related matrix-associated actin-dependent regulator of chromatin subfamily E member 1-related , GN=HMG20B	1	1	1	317		35.791	9.35
Q8NDZ9	Putative uncharacterized protein LOC439951 , PE=5	1	1	1	215		22.292	12.41
Q96GA3	Protein LTV1 homolog , GN=LTV1	1	1	1	475		54.821	4.91
P23677	Inositol-trisphosphate 3-kinase A , GN=ITPKA	1	1	1	461		50.977	7.65
Q14181	DNA polymerase alpha subunit B , GN=POLA2	1	1	1	598		65.906	5.24
P53701	Cytochrome c-type heme lyase , GN=HCCS	1	1	1	268		30.582	6.68
O75147	Obscurin-like protein 1 , GN=OBSL1	1	1	1	1896		206.817	5.63
Q8N9V7	Testis- and ovary-specific PAZ domain-containing protein 1 , GN=TOPAZ1 PE=2	1	1	1	1692		190.806	7.87
Q03393	6-pyruvoyl tetrahydrobiopterin synthase , GN=PTS	1	1	1	145		16.375	6.68
Q14997	Proteasome activator complex subunit 4 , GN=PSME4	1	1	1	1843		211.199	6.9
Q7Z5Q1-9	Isoform 8 of Cytoplasmic polyadenylation element-binding protein 2 , GN=CPEB2	1	1	1	1034		109.735	8.12
Q0ZME4	Membrane protein OS=Human coronavirus HKU1 (isolate N5) GN=M PE=3	1	1	1	223		25.641	9.39
Q99490	Arf-GAP with GTPase, ANK repeat and PH domain-containing protein 2 , GN=AGAP2	1	1	1	1192		124.597	9.89
Q9BSQ5-2	Isoform 2 of Cerebral cavernous malformations 2 protein , GN=CCM2	1	1	1	465		51.381	5.6
P36762	Minor capsid protein L2 OS=Human papillomavirus type 49 GN=L2 PE=3	1	1	1	521	57.02		5.14
O14910	Protein lin-7 homolog A , GN=LIN7A	1	1	1	233		25.981	8.72
O94830	Phospholipase DDHD2 , GN=DDHD2	1	1	1	711	80.98		5.39
O00159	Unconventional myosin-Ic , GN=MYO1C	1	1	1	1063		121.606	9.41
O94913	Pre-mRNA cleavage complex 2 protein Pcf11 , GN=PCF11	1	1	1	1555		172.944	8.48
Q8NEZ5	F-box only protein 22 , GN=FBXO22	1	1	1	403	44.48		7.03
Q6UXV4	MICOS complex subunit MIC27 , GN=APOOL	1	1	1	268	29.14		9.52
Q9ULX9	Transcription factor MafF , GN=MAFF	1	1	1	164		17.749	9.8
Q9BTZ2	Dehydrogenase/reductase SDR family member 4 , GN=DHRS4	1	1	1	278		29.518	8.56
Q86WR6	Uncharacterized protein C17orf64 , GN=C17orf64 PE=2	1	1	1	236		27.165	9.61
Q9BYB4	Guanine nucleotide-binding protein subunit beta-like protein 1 , GN=GNB1L	1	1	1	327		35.595	7.97

Q9H9A5-6	Isoform 6 of CCR4-NOT transcription complex subunit 10 , GN=CNOT10	1	1	1	804		89.084	8.21
Q12834	Cell division cycle protein 20 homolog , GN=CDC20	1	1	1	499		54.689	9.23
Q9BWH6	RNA polymerase II-associated protein 1 , GN=RPAP1	1	1	1	1393		152.659	6.38
O95379-4	Isoform 4 of Tumor necrosis factor alpha-induced protein 8 , GN=TNFAIP8	1	1	1	210	24.37		8.78
Q14894	Ketimine reductase mu-crystallin , GN=CRYM	1	1	1	314		33.754	5.14
Q8NCX0	Coiled-coil domain-containing protein 150 , GN=CCDC150	1	1	1	1101		128.681	7.02
Q71SY5-3	Isoform 3 of Mediator of RNA polymerase II transcription subunit 25 , GN=MED25	1	1	1	796	82.92		8.84
O14562	Ubiquitin domain-containing protein UBFD1 , GN=UBFD1	1	1	1	309		33.361	5.77
Q9UMF0	Intercellular adhesion molecule 5 ,	1	1	1	924		97.056	5.95
P17405	Sphingomyelin phosphodiesterase ,	1	1	1	629		69.707	7.28
Q86Y82	Syntaxin-12 , GN=STX12	1	1	1	276		31.622	5.59
Q9BR61	Acyl-CoA-binding domain-containing protein 6 , GN=ACBD6	1	1	1	282		31.131	5.11
Q7Z7H8-2	Isoform 2 of 39S ribosomal protein L10, mitochondrial , GN=MRPL10	1	1	1	271		30.288	9.25
Q6V0I7	Protocadherin Fat 4 , GN=FAT4	1	1	1	4981		542.351	4.94
Q9Y2W6	Tudor and KH domain-containing protein , GN=TDRKH	1	1	1	561		62.007	5.02
Q9HBL0	Tensin-1 , GN=TNS1	1	1	1	1735		185.586	7.75
Q9BWD3	Retrotransposon Gag-like protein 8A ,	1	1	1	113	13.18		5.07
Q8N5G0-2	Isoform 2 of Small integral membrane protein 20 , GN=SMIM20	1	1	1	168		18.391	10.99
Q9BXF6	Rab11 family-interacting protein 5 , GN=RAB11FIP5	1	1	1	653		70.372	9.23
Q9Y2E4	Disco-interacting protein 2 homolog C , GN=DIP2C	1	1	1	1556		170.658	7.39
Q96JM7	Lethal(3)malignant brain tumor-like protein 3 , GN=L3MBTL3	1	1	1	780	88.28		6.46
A6NJ78	Probable methyltransferase-like protein 15 , GN=METTL15	1	1	1	407		46.092	8.62
Q8TEA1	Putative methyltransferase NSUN6 ,	1	1	1	469		51.737	7.96
O15381	Nuclear valosin-containing protein-like , GN=NVL	1	1	1	856		94.991	6.48
Q15554	Telomeric repeat-binding factor 2 , GN=TERF2	1	1	1	542		59.557	9.35
Q8TD55	Pleckstrin homology domain-containing family O member 2 , GN=PLEKHO2	1	1	1	490		53.317	5.43
Q9NUY8	TBC1 domain family member 23 ,	1	1	1	699		78.272	5.41
Q9BUI4	DNA-directed RNA polymerase III subunit RPC3 , GN=POLR3C	1	1	1	534		60.573	7.31
Q92574	Hamartin , GN=TSC1	1	1	1	1164		129.685	6.47
Q96PM9	Zinc finger protein 385A , GN=ZNF385A	1	1	1	386		40.428	9.86
P02647	Apolipoprotein A-I , GN=APOA1	1	1	1	267		30.759	5.76
Q9BRQ6	MICOS complex subunit MIC25 , GN=CHCHD6	1	1	1	235		26.441	8.85
Q7L8L6	FAST kinase domain-containing protein 5, mitochondrial , GN=FASTKD5	1	1	1	764		86.519	8.13
Q9Y680	Peptidyl-prolyl cis-trans isomerase FKBP7 , GN=FKBP7	1	1	1	259	29.99		6.54
Q9NYK5-2	Isoform 2 of 39S ribosomal protein L39, mitochondrial , GN=MRPL39	1	1	1	353		40.432	7.91
Q13426	DNA repair protein XRCC4 , GN=XRCC4	1	1	1	336		38.263	4.98
Q9Y3A3	MOB-like protein phocein , GN=MOB4	1	1	1	225		26.016	5.78
O95619	YEATS domain-containing protein 4 ,	1	1	1	227		26.483	8.41
Q9ULE0	Protein WWC3 , GN=WWC3	1	1	1	1092	122.6		6.37
Q96FV9	THO complex subunit 1 , GN=THOC1	1	1	1	657		75.619	4.98
P82675	28S ribosomal protein S5, mitochondrial , GN=MRPS5	1	1	1	430		47.976	9.92
Q5JTV8	Torsin-1A-interacting protein 1 ,	1	1	1	583		66.208	8.18
P51178-2	Isoform 2 of 1-phosphatidylinositol 4,5-bisphosphate phosphodiesterase delta-1 , GN=PLCD1	1	1	1	777		88.079	7.23

Q8N5I9	Uncharacterized protein C12orf45 , GN=C12orf45	1	1	1	185		20.111	5.22
Q99523	Sortilin , GN=SORT1	1	1	1	831		92.009	5.74
Q13884	Beta-1-syntrophin , GN=SNTB1	1	1	1	538		58.025	8.63
Q9BZL6	Serine/threonine-protein kinase D2 ,	1	1	1	878		96.689	6.84
Q15291	Retinoblastoma-binding protein 5 ,	1	1	1	538		59.116	5.1
Q9C0K0	B-cell lymphoma/leukemia 11B , GN=BCL11B	1	1	1	894		95.459	6.55
O95905-3	Isoform 3 of Protein ecdysoneless homolog , GN=ECD	1	1	1	677	76.46		4.86
Q16849	Receptor-type tyrosine-protein phosphatase-like N , GN=PTPRN	1	1	1	979		105.781	7.11
P34949	Mannose-6-phosphate isomerase , GN=MPI	1	1	1	423		46.626	5.95
Q8WU90	Zinc finger CCCH domain-containing protein 15 , GN=ZC3H15	1	1	1	426		48.573	5.31
P32856	Syntaxin-2 , GN=STX2	1	1	1	288	33.32		6.28
P48751-3	Isoform 3 of Anion exchange protein 3 , GN=SLC4A3	1	1	1	1259		138.575	6.4
A4FU28	cTAGE family member 9 , GN=CTAGE9 PE=2	1	1	1	777		87.898	5.31
P25398	40S ribosomal protein S12 , GN=RPS12	1	1	1	132		14.505	7.21
Q92599	Septin-8 , GN=SEPT8	1	1	1	483		55.721	6.28
Q6PEV8	Protein FAM199X , GN=FAM199X	1	1	1	388		42.775	5.08
Q86WA6	Valacyclovir hydrolase , GN=BPHL	1	1	1	291		32.522	9.14
Q8TBF4	Zinc finger CCHC-type and RNA-binding motif-containing protein 1 , GN=ZCRB1	1	1	1	217		24.576	8.53
Q9NT99	Leucine-rich repeat-containing protein 4B , GN=LRRC4B PE=2	1	1	1	713		76.386	7.23
P59827	BPI fold-containing family B member 4 , GN=BPIFB4 PE=2	1	1	1	614		65.013	5.11
P28284	E3 ubiquitin-protein ligase ICP0 OS=Human herpesvirus 2 (strain HG52) GN=RL2	1	1	1	825		81.936	8.13
Q96K80	Zinc finger CCCH domain-containing protein 10 , GN=ZC3H10	1	1	1	434		46.023	7.64
P61009	Signal peptidase complex subunit 3 ,	1	1	1	180		20.301	8.62
P0DI81-3	Isoform 3 of Trafficking protein particle complex subunit 2 , GN=TRAPPC2	1	1	1	174		20.361	7.02
Q13136	Liprin-alpha-1 , GN=PPFIA1	1	1	1	1202		135.695	6.29
Q6UUV9-2	Isoform 2 of CREB-regulated transcription coactivator 1 , GN=CRTC1	1	1	1	650		68.739	5.95
Q9UFC0	Leucine-rich repeat and WD repeat-containing protein 1 , GN=LRWD1	1	1	1	647		70.816	7.21
P45983	Mitogen-activated protein kinase 8 ,	1	1	1	427		48.264	6.89
Q8TF46	DIS3-like exonuclease 1 , GN=DIS3L	1	1	1	1054		120.711	6.54
Q96T83	Sodium/hydrogen exchanger 7 , GN=SLC9A7	1	1	1	725	80.08		6.42
Q9UG01	Intraflagellar transport protein 172 homolog , GN=IFT172	1	1	1	1749		197.451	6.13
P09619	Platelet-derived growth factor receptor beta , GN=PDGFRB	1	1	1	1106		123.889	4.98
Q9BVC4	Target of rapamycin complex subunit LST8 , GN=MLST8	1	1	1	326		35.853	5.86
Q96QE3	ATPase family AAA domain-containing protein 5 , GN=ATAD5	1	1	1	1844		207.441	9.19
P41743	Protein kinase C iota type , GN=PRKCI	1	1	1	596		68.218	5.85
Q9H1D9	DNA-directed RNA polymerase III subunit RPC6 , GN=POLR3F	1	1	1	316		35.661	6.11
Q495W5	Alpha-(1,3)-fucosyltransferase 11 , GN=FUT11	1	1	1	492		55.781	5.94
Q9BYN8	28S ribosomal protein S26, mitochondrial , GN=MRPS26	1	1	1	205		24.197	10.39
Q9H2L5-2	Isoform 2 of Ras association domain-containing protein 4 , GN=RASSF4	1	1	1	330		37.231	7.47
Q8N5A5	Zinc finger CCCH-type with G patch domain-containing protein , GN=ZGPAT	1	1	1	531		57.324	5.43
P55081	Microfibrillar-associated protein 1 ,	1	1	1	439		51.927	4.98
Q8NDA8	Maestro heat-like repeat-containing protein family member 1 , GN=MROH1 PE=2	1	1	1	1641		181.134	6.89
Q12851	Mitogen-activated protein kinase kinase kinase kinase 2 , GN=MAP4K2	1	1	1	820		91.498	6.34

Q8NEF9	Serum response factor-binding protein 1 , GN=SRFBP1	1	1	1	429	48.604	9.58
Q9UKZ1	CCR4-NOT transcription complex subunit 11 , GN=CNOT11	1	1	1	510	55.18	6.4
Q8N653	Leucine-zipper-like transcriptional regulator 1 , GN=LZTR1	1	1	1	840	94.658	6.57
P20930	Filaggrin , GN=FLG	1	1	1	4061	434.922	9.25
Q5JSL3	Dedicator of cytokinesis protein 11 , GN=DOCK11	1	1	1	2073	237.519	7.74
Q14106	Protein Tob2 , GN=TOB2	1	1	1	344	36.609	6.93
Q2VWA4	SKI family transcriptional corepressor 2 , GN=SKOR2	1	1	1	1001	104.17	6.48
Q9HCM1	Uncharacterized protein KIAA1551 , GN=KIAA1551	1	1	1	1747	194.736	8.78
Q13740	CD166 antigen , GN=ALCAM	1	1	1	583	65.061	6.25
Q96EV2	RNA-binding protein 33 , GN=RBM33	1	1	1	1170	129.906	6.93
Q96CV9	Optineurin , GN=OPTN	1	1	1	577	65.88	5.24
Q9H8H0	Nucleolar protein 11 , GN=NOL11	1	1	1	719	81.072	6.07
D5LX59	Large tegument protein deneddylase OS=Human cytomegalovirus (strain Towne) GN=UL48	1	1	1	2242	253.207	6.87
Q8NCB2	CaM kinase-like vesicle-associated protein , GN=CAMKV PE=2	1	1	1	501	54.32	5.55
Q9BXJ3	Complement C1q tumor necrosis factor-related protein 4 , GN=C1QTNF4	1	1	1	329	35.234	8.27
Q2TAA2	Isoamyl acetate-hydrolyzing esterase 1 homolog , GN=IAH1	1	1	1	248	27.581	5.3
Q9Y2R9	28S ribosomal protein S7, mitochondrial , GN=MRPS7	1	1	1	242	28.116	9.99
O75718	Cartilage-associated protein , GN=CRTAP	1	1	1	401	46.532	5.73
Q05707	Collagen alpha-1(XIV) chain , GN=COL14A1	1	1	1	1796	193.394	5.3
O95825	Quinone oxidoreductase-like protein 1 , GN=CRYZL1	1	1	1	349	38.672	5.78
Q92934	Bcl2-associated agonist of cell death ,	1	1	1	168	18.381	7.15
P27482	Calmodulin-like protein 3 , GN=CALML3	1	1	1	149	16.88	4.42
Q5BJH7	Protein YIF1B , GN=YIF1B	1	1	1	314	34.413	9.16
Q8N1G1	RNA exonuclease 1 homolog , GN=REXO1	1	1	1	1221	131.429	8.95
O15297	Protein phosphatase 1D , GN=PPM1D	1	1	1	605	66.633	8.94
Q96LP6	Uncharacterized protein C12orf42 , GN=C12orf42 PE=2	1	1	1	360	39.713	9.6
O43427	Acidic fibroblast growth factor intracellular-binding protein , GN=FIBP	1	1	1	364	41.851	6.48
Q9Y618	Nuclear receptor corepressor 2 , GN=NCOR2	1	1	1	2525	274.635	7.59
P0CG34	Thymosin beta-15A , GN=TMSB15A	1	1	1	45	5.226	5.36
P03265	DNA-binding protein OS=Human adenovirus C serotype 5 GN=DBP	1	1	1	529	59.102	7.93
P28288	ATP-binding cassette sub-family D member 3 , GN=ABCD3	1	1	1	659	75.428	9.36
O94967-4	Isoform 4 of WD repeat-containing protein 47 , GN=WDR47	1	1	1	927	102.875	6.04
A0AVF1	Intraflagellar transport protein 56 , GN=TTC26 PE=2	1	1	1	554	64.136	6.93
P35243	Recoverin , GN=RCVRN	1	1	1	200	23.116	5.16
Q9BV19	Uncharacterized protein C1orf50 ,	1	1	1	199	21.863	5.88
Q7Z7A4	PX domain-containing protein kinase-like protein , GN=PXK	1	1	1	578	64.909	9.35
Q9Y5X3	Sorting nexin-5 , GN=SNX5	1	1	1	404	46.787	6.76
Q9Y664	KICSTOR complex protein kaptin , GN=KPTN	1	1	1	436	48.05	5.26
P0CG12	Chromosome transmission fidelity protein 8 homolog isoform 2 , GN=CHTF8	1	1	1	524	51.359	12.41
Q9UKL3	CASP8-associated protein 2 , GN=CASP8AP2	1	1	1	1982	222.52	6.58
Q9Y2B9	cAMP-dependent protein kinase inhibitor gamma , GN=PKIG PE=2	1	1	1	76	7.906	4.25
Q9BW62	Katanin p60 ATPase-containing subunit A-like 1 , GN=KATNAL1	1	1	1	490	55.357	6.74
P45877	Peptidyl-prolyl cis-trans isomerase C ,	1	1	1	212	22.749	8.4
Q05469	Hormone-sensitive lipase , GN=LIPE	1	1	1	1076	116.525	6.7

Q9Y3B8	Oligoribonuclease, mitochondrial , GN=REXO2	1	1	1	237		26.816	6.87
Q9HAR2-4	Isoform 4 of Adhesion G protein-coupled receptor L3 , GN=ADGRL3	1	1	1	1469		164.476	6.51
Q5SY16	Polynucleotide 5'-hydroxyl-kinase NOL9 , GN=NOL9	1	1	1	702		79.272	9.13
Q8NEB7	Acrosin-binding protein , GN=ACRBP PE=2	1	1	1	543	61.32		5.16
P53367	Arfaptin-1 , GN=ARFIP1	1	1	1	373		41.713	6.7
Q96DT7	Zinc finger and BTB domain-containing protein 10 , GN=ZBTB10	1	1	1	871		94.835	5.16
Q8N0Z3	Spindle and centriole-associated protein 1 , GN=SPICE1	1	1	1	855		96.205	7.44
O43920	NADH dehydrogenase [ubiquinone] iron-sulfur protein 5 , GN=NDUFS5	1	1	1	106		12.509	9.14
O75886	Signal transducing adapter molecule 2 , GN=STAM2	1	1	1	525		58.128	5.07
Q9NQ11	Cation-transporting ATPase 13A2 ,	1	1	1	1180		128.711	8.12
Q96T21	Selenocysteine insertion sequence-binding protein 2 , GN=SECISBP2	1	1	1	854		95.402	8.12
Q9HCK4-3	Isoform 3 of Roundabout homolog 2 ,	1	1	1	1394		153.025	6.67
Q8WTS1	1-acylglycerol-3-phosphate O-acyltransferase ABHD5 , GN=ABHD5	1	1	1	349		39.071	6.61
Q96RL6	Sialic acid-binding Ig-like lectin 11 , GN=SIGLEC11	1	1	1	698		75.747	7.5
P19878	Neutrophil cytosol factor 2 , GN=NCF2	1	1	1	526		59.723	6.16
Q14692	Ribosome biogenesis protein BMS1 homolog , GN=BMS1	1	1	1	1282		145.716	6.44
Q0P651	Protein ABHD18 , GN=ABHD18 PE=2	1	1	1	414		46.924	9.28
Q9UPW5-3	Isoform 3 of Cytosolic carboxypeptidase 1 , GN=AGTPBP1	1	1	1	1238	138.56		6.21
Q96AQ6	Pre-B-cell leukemia transcription factor-interacting protein 1 , GN=PBXIP1	1	1	1	731		80.594	5.33
Q9P2M1	LRP2-binding protein , GN=LRP2BP	1	1	1	347		39.755	7.9
P07305	Histone H1.0 , GN=H1F0	1	1	1	194	20.85		10.84
A7KAX9	Rho GTPase-activating protein 32 , GN=ARHGAP32	1	1	1	2087		230.385	6.74
Q96CU9	FAD-dependent oxidoreductase domain-containing protein 1 , GN=FOXRED1	1	1	1	486		53.778	7.78
Q6RW13	Type-1 angiotensin II receptor-associated protein , GN=AGTRAP	1	1	1	159		17.408	6.14
Q86V15	Zinc finger protein castor homolog 1 ,	1	1	1	1759		189.948	7.03
O75695	Protein XRP2 , GN=RP2	1	1	1	350		39.615	5.12
Q8N4P3	Guanosine-3',5'-bis(diphosphate) 3'-pyrophosphohydrolase MESH1 , GN=HDDC3	1	1	1	179		20.317	6.74
Q9Y2Q0	Phospholipid-transporting ATPase IA , GN=ATP8A1	1	1	1	1164		131.285	6.84
Q8NEE8	Tetratricopeptide repeat protein 16 , GN=TTC16 PE=2	1	1	1	873		98.248	9.01
Q15751	Probable E3 ubiquitin-protein ligase HERC1 , GN=HERC1	1	1	1	4861		531.891	6.04
Q8TF44	C2 calcium-dependent domain-containing protein 4C , GN=C2CD4C	1	1	1	421		44.549	9.73
O60504	Vinexin , GN=SORBS3	1	1	1	671		75.295	9.45
Q14149	MORC family CW-type zinc finger protein 3 , GN=MORC3	1	1	1	939		107.045	5.6
P50914	60S ribosomal protein L14 , GN=RPL14	1	1	1	215		23.417	10.93
O15228	Dihydroxyacetone phosphate acyltransferase , GN=GNPAT	1	1	1	680		77.138	6.57
P82921	28S ribosomal protein S21, mitochondrial , GN=MRPS21	1	1	1	87		10.734	10.21
Q86UA1	Pre-mRNA-processing factor 39 , GN=PRPF39	1	1	1	669		78.381	5.4
Q16623	Syntaxin-1A , GN=STX1A	1	1	1	288		33.003	5.24
Q5JSZ5	Protein PRRC2B , GN=PRRC2B	1	1	1	2229		242.817	8.34
P10398	Serine/threonine-protein kinase A-Raf , GN=ARAF	1	1	1	606		67.542	9.01
O60674	Tyrosine-protein kinase JAK2 , GN=JAK2	1	1	1	1132	130.59		7.21
Q5K2K4	Glycoprotein OS=Isfahan virus GN=G PE=3	1	1	1	523		58.305	7.11
Q15526	Surfeit locus protein 1 , GN=SURF1	1	1	1	300	33.31		9.6

Q9NXG6-3	Isoform 3 of Transmembrane prolyl 4-hydroxylase , GN=P4HTM	1	1	1	563	63.072	6.1
O60258	Fibroblast growth factor 17 , GN=FGF17	1	1	1	216	24.876	10.42
Q9NP66	High mobility group protein 20A ,	1	1	1	347	40.119	6.49
P17900	Ganglioside GM2 activator , GN=GM2A	1	1	1	193	20.825	5.31
Q9Y3Z3	Deoxynucleoside triphosphate triphosphohydrolase SAMHD1 , GN=SAMHD1	1	1	1	626	72.155	7.14
Q6IAN0	Dehydrogenase/reductase SDR family member 7B , GN=DHRS7B	1	1	1	325	35.097	9.55
Q9H7P9	Pleckstrin homology domain-containing family G member 2 , GN=PLEKHG2	1	1	1	1386	147.877	5.86
Q96B23	Uncharacterized protein C18orf25 , GN=C18orf25	1	1	1	403	43.298	4.88
Q3MIS6	Zinc finger protein 528 , GN=ZNF528 PE=2	1	1	1	628	72.092	9.19
Q8N103	T-cell activation Rho GTPase-activating protein , GN=TAGAP	1	1	1	731	80.653	6.47
Q15648	Mediator of RNA polymerase II transcription subunit 1 , GN=MED1	1	1	1	1581	168.373	8.73
Q8NI36	WD repeat-containing protein 36 ,	1	1	1	951	105.255	7.53
Q96J01	THO complex subunit 3 , GN=THOC3	1	1	1	351	38.747	6.09
Q8IYB7	DIS3-like exonuclease 2 , GN=DIS3L2	1	1	1	885	99.216	6.1
P04156-2	Isoform 2 of Major prion protein , GN=PRNP	1	1	1	246	26.868	9.11
P30281	G1/S-specific cyclin-D3 , GN=CCND3	1	1	1	292	32.499	7.06
O76041	Nebulette , GN=NEBL	1	1	1	1014	116.38	7.99
Q02040	A-kinase anchor protein 17A , GN=AKAP17A	1	1	1	695	80.686	9.73
Q9NSY1	BMP-2-inducible protein kinase , GN=BMP2K	1	1	1	1161	129.091	6.51
Q6P2P2	Protein arginine N-methyltransferase 9 , GN=PRMT9	1	1	1	845	94.441	5.25
Q96M91	Cilia- and flagella-associated protein 53 , GN=CFAP53	1	1	1	514	61.796	8.9
P53985	Monocarboxylate transporter 1 ,	1	1	1	500	53.909	8.66
P21980	Protein-glutamine gamma-glutamyltransferase 2 , GN=TGM2	1	1	1	687	77.28	5.22
B3SRQ8	Non-structural glycoprotein 4 OS=Rotavirus A (strain RVA/Human/Indonesia/69M/1980/G8P4[10]) PE=3	1	1	1	175	20.336	8.32
Q9Y6N8	Cadherin-10 , GN=CDH10	1	1	1	788	88.397	4.98
P61254	60S ribosomal protein L26 , GN=RPL26	1	1	1	145	17.248	10.55
Q80943	Replication protein E1 OS=Human papillomavirus type 60 GN=E1 PE=3	1	1	1	610	69.812	5.4
Q86W92	Liprin-beta-1 , GN=PPFIBP1	1	1	1	1011	113.952	5.55
Q5HYI8	Rab-like protein 3 , GN=RABL3	1	1	1	236	26.407	7.11
Q5T6C5	Ataxin-7-like protein 2 , GN=ATXN7L2	1	1	1	722	77.132	9.23
P0C6A0	GATA-type zinc finger protein 1 , GN=ZGLP1	1	1	1	271	29.609	9.03
P50579	Methionine aminopeptidase 2 , GN=METAP2	1	1	1	478	52.858	5.82
O96008	Mitochondrial import receptor subunit TOM40 homolog , GN=TOMM40	1	1	1	361	37.869	7.25
Q8IXK0-5	Isoform 5 of Polyhomeotic-like protein 2 , GN=PHC2	1	1	1	859	90.756	8.69
P41247	Patatin-like phospholipase domain-containing protein 4 , GN=PNPLA4 PE=2	1	1	1	253	27.963	9.11
Q8TB37	Iron-sulfur protein NUBPL , GN=NUBPL	1	1	1	319	34.061	9.04
Q7Z569	BRCA1-associated protein , GN=BRAP	1	1	1	592	67.261	5.92
Q8WWC4	m-AAA protease-interacting protein 1, mitochondrial , GN=MAIP1	1	1	1	291	32.524	9.17
Q6P1L8	39S ribosomal protein L14, mitochondrial , GN=MRPL14	1	1	1	145	15.937	10.24
Q5VX52	Spermatogenesis-associated protein 1 , GN=SPATA1 PE=2	1	1	1	437	50.276	8.43
Q13586	Stromal interaction molecule 1 , GN=STIM1	1	1	1	685	77.375	6.67
Q9BQ70	Transcription factor 25 , GN=TCF25	1	1	1	676	76.619	6.35
Q9H173	Nucleotide exchange factor SIL1 , GN=SIL1	1	1	1	461	52.052	5.36
Q9Y221	60S ribosome subunit biogenesis protein NIP7 homolog , GN=NIP7	1	1	1	180	20.449	8.51
P49643	DNA primase large subunit , GN=PRIM2	1	1	1	509	58.769	7.91
Q6ZNJ1	Neurobeachin-like protein 2 , GN=NBEAL2	1	1	1	2754	302.326	6.38

Q9Y5K1	Meiotic recombination protein SPO11 , GN=SPO11 PE=2	1	1	1	396	44.508	8.87
Q8NB25	Protein FAM184A , GN=FAM184A PE=2	1	1	1	1140	132.883	5.83
P48728	Aminomethyltransferase, mitochondrial , GN=AMT	1	1	1	403	43.918	8.57
P49247	Ribose-5-phosphate isomerase , GN=RPIA	1	1	1	311	33.248	8.54
Q9ULQ1-3	Isoform 3 of Two pore calcium channel protein 1 , GN=TPCN1	1	1	1	888	101.73	7.15
P48549	G protein-activated inward rectifier potassium channel 1 , GN=KCNJ3	1	1	1	501	56.567	8.22
O15068-3	Isoform 3 of Guanine nucleotide exchange factor DBS , GN=MCF2L	1	1	1	1067	120.542	6.77
O75054-2	Isoform 2 of Immunoglobulin superfamily member 3 , GN=IGSF3	1	1	1	1214	137.43	6.09
Q86YM7	Homer protein homolog 1 , GN=HOMER1	1	1	1	354	40.252	5.44
P15391-2	Isoform 2 of B-lymphocyte antigen CD19 , GN=CD19	1	1	1	557	61.161	4.98
Q6PDB4	Zinc finger protein 880 , GN=ZNF880 PE=2	1	1	1	577	66.719	9.29
P04053	DNA nucleotidylexotransferase , GN=DNTT	1	1	1	509	58.499	8.43
P82970	High mobility group nucleosome-binding domain-containing protein 5 , GN=HMGN5	1	1	1	282	31.506	4.55
Q9UIF9	Bromodomain adjacent to zinc finger domain protein 2A , GN=BAZ2A	1	1	1	1905	211.065	6.64
Q96SB4-3	Isoform 1 of SRSF protein kinase 1 ,	1	1	1	826	92.354	7.71
O43307-3	Isoform 3 of Rho guanine nucleotide exchange factor 9 , GN=ARHGEF9	1	1	1	414	49.426	7.08
Q9NZ01	Very-long-chain enoyl-CoA reductase ,	1	1	1	308	36.011	9.45
Q5TF58	Intermediate filament family orphan 2 , GN=IFFO2 PE=2	1	1	1	517	57.292	4.97
P78509	Reelin , GN=RELN	1	1	1	3460	388.137	5.88
P01023	Alpha-2-macroglobulin , GN=A2M	1	1	1	1474	163.188	6.46
Q6P1X6	UPF0598 protein C8orf82 , GN=C8orf82	1	1	1	216	23.874	9.14
Q02413-2	Isoform 2 of Desmoglein-1 , GN=DSG1	1	1	1	408	42.592	5.24
Q9BSM1	Polycomb group RING finger protein 1 , GN=PCGF1	1	1	1	259	30.327	8.91
Q6ZR52-2	Isoform 2 of Zinc finger protein 493 ,	1	1	1	774	89.765	9.16
P12036	Neurofilament heavy polypeptide , GN=NEFH	1	1	1	1026	112.411	6.18
P62910	60S ribosomal protein L32 , GN=RPL32	1	1	1	135	15.85	11.33
Q6ZN03	Putative uncharacterized protein encoded by LINC00322 , GN=LINC00322 PE=5	1	1	1	302	32.353	8.68
Q9P2H3-3	Isoform IFT80-L of Intraflagellar transport protein 80 homolog , GN=IFT80	1	1	1	1080	123.242	7.08
B3SRR9	Outer capsid protein VP4 OS=Rotavirus A (strain RVA/Human/United States/D/1974/G1P1A[8]) PE=3	1	1	1	775	87.344	6.9
Q9Y6N7-2	Isoform 2 of Roundabout homolog 1 ,	1	1	1	1655	181.216	6.07
Q5T619	Zinc finger protein 648 , GN=ZNF648	1	1	1	568	62.301	8.62
Q8IVW6	AT-rich interactive domain-containing protein 3B , GN=ARID3B	1	1	1	561	60.6	7.52
Q8N2G4-2	Isoform 2 of Ly6/PLAUR domain-containing protein 1 , GN=LYPD1	1	1	1	164	17.75	8.38
Q9BUK6	Protein misato homolog 1 , GN=MSTO1	1	1	1	570	61.796	6.11
P48169	Gamma-aminobutyric acid receptor subunit alpha-4 , GN=GABRA4 PE=2	1	1	1	554	61.583	9.55
P62249	40S ribosomal protein S16 , GN=RPS16	1	1	1	146	16.435	10.21
Q9H5J0	Zinc finger and BTB domain-containing protein 3 , GN=ZBTB3	1	1	1	574	61.789	5.62
Q9P2D1	Chromodomain-helicase-DNA-binding protein 7 , GN=CHD7	1	1	1	2997	335.717	6.34
O60673	DNA polymerase zeta catalytic subunit , GN=REV3L	1	1	1	3130	352.554	8.47
Q9Y3S1-3	Isoform 3 of Serine/threonine-protein kinase WNK2 , GN=WNK2	1	1	1	779	84.815	5.44
Q9P219	Protein Daple , GN=CCDC88C	1	1	1	2028	228.091	6.23
Q9NS15	Latent-transforming growth factor beta-binding protein 3 , GN=LTBP3	1	1	1	1303	139.267	6.07

Q9UPX8-3	Isoform 3 of SH3 and multiple ankyrin repeat domains protein 2 , GN=SHANK2	1	1	1	1849		201.136	6.7
Q6ZMS4	Zinc finger protein 852 , GN=ZNF852	1	1	1	543		62.044	8.12
Q8N3A8-2	Isoform 2 of Poly [ADP-ribose] polymerase 8 , GN=PARP8	1	1	1	812		91.101	8.37
P54687-5	Isoform 5 of Branched-chain-amino-acid aminotransferase, cytosolic , GN=BCAT1	1	1	1	398		44.103	5.39
P17027	Zinc finger protein 23 , GN=ZNF23	1	1	1	643		73.012	8.12
O60825	6-phosphofructo-2-kinase/fructose-2,6-bisphosphatase 2 , GN=PFKFB2	1	1	1	505	58.44		8.38
P25391	Laminin subunit alpha-1 , GN=LAMA1	1	1	1	3075		336.867	6.35
Q96QC0	Serine/threonine-protein phosphatase 1 regulatory subunit 10 , GN=PPP1R10	1	1	1	940		98.996	9.17
Q13572	Inositol-tetrakisphosphate 1-kinase ,	1	1	1	414		45.592	6.16
P04035-3	Isoform 3 of 3-hydroxy-3-methylglutaryl-coenzyme A reductase , GN=HMGCR	1	1	1	908		99.687	6.77
Q8TDF5	Neuropilin and tolloid-like protein 1 , GN=NETO1 PE=2	1	1	1	533		60.153	7.02
Q9H4S2	GS homeobox 1 , GN=GSX1 PE=2	1	1	1	264		27.865	8.92
Q96JN2-4	Isoform 4 of Coiled-coil domain-containing protein 136 , GN=CCDC136	1	1	1	1157		134.583	4.67
P32242	Homeobox protein OTX1 , GN=OTX1	1	1	1	354		37.304	9.33
Q9H251-8	Isoform 8 of Cadherin-23 , GN=CDH23	1	1	1	3357		369.424	4.67
Q9H2U1	ATP-dependent RNA helicase DHX36 , GN=DHX36	1	1	1	1008		114.688	7.68
Q9UGJ0	5'-AMP-activated protein kinase subunit gamma-2 , GN=PRKAG2	1	1	1	569		63.027	9.35
Q96GE6-2	Isoform 2 of Calmodulin-like protein 4 , GN=CALML4	1	1	1	120		13.652	6.38
Q86YW9	Mediator of RNA polymerase II transcription subunit 12-like protein , GN=MED12L	1	1	1	2145		239.967	7.77
Q14993	Collagen alpha-1(XIX) chain , GN=COL19A1	1	1	1	1142		115.149	8.32
Q9NPD3	Exosome complex component RRP41 , GN=EXOSC4	1	1	1	245		26.366	6.52
Q14919-2	Isoform 2 of Dr1-associated corepressor , GN=DRAP1	1	1	1	211		23.133	5.27
Q5RHP9	Glutamate-rich protein 3 , GN=ERICH3 PE=2	1	1	1	1530		168.363	4.88
Q01968	Inositol polyphosphate 5-phosphatase OCRL-1 , GN=OCRL	1	1	1	901		104.138	6.55
Q9QJ37	Large tegument protein deneddylase OS=Human herpesvirus 6B (strain Z29) GN=U31 PE=3	1	1	1	2077		240.134	6.64
O60870	DNA/RNA-binding protein KIN17 , GN=KIN	1	1	1	393		45.345	8.95
A6BM72-3	Isoform 3 of Multiple epidermal growth factor-like domains protein 11 , GN=MEGF11	1	1	1	875		91.369	6.04
Q8TB03	Uncharacterized protein CXorf38 ,	1	1	1	319		36.646	6.3
Q9Y2J4-3	Isoform 3 of Angiomotin-like protein 2 , GN=AMOTL2	1	1	1	777		85.428	7.18
Q32M88	Protein-glucosylgalactosylhydroxylysine glucosidase , GN=PGGHG	1	1	1	737		80.604	5.25
Q8N5J2	Ubiquitin carboxyl-terminal hydrolase MINDY-1 , GN=MINDY1	1	1	1	469		51.746	4.86
Q9NZU5	LIM and cysteine-rich domains protein 1 , GN=LMCD1	1	1	1	365		40.806	7.93
Q03692	Collagen alpha-1(X) chain , GN=COL10A1	1	1	1	680		66.117	9.67
Q9H479	Fructosamine-3-kinase , GN=FN3K	1	1	1	309		35.149	7.55
Q9JFB7	Thymidine kinase OS=Vaccinia virus (strain Tian Tan) GN=TK PE=3	1	1	1	177		20.105	6.02
Q56UN5	Mitogen-activated protein kinase kinase kinase 19 , GN=MAP3K19 PE=2	1	1	1	1328		150.442	7.05
Q5W0Q7	SUMO-specific isopeptidase USPL1 ,	1	1	1	1092		120.364	6.19
Q8N729	Neuropeptide W , GN=NPW	1	1	1	165		18.037	11.62
P82094-2	Isoform 2 of TATA element modulatory factor , GN=TMF1	1	1	1	1096	123.08		4.92
Q9H694	Protein bicaudal C homolog 1 , GN=BICC1	1	1	1	974		104.778	8.54
Q6P1W5	Uncharacterized protein C1orf94 ,	1	1	1	598		65.312	8.28
Q9BTE7	DCN1-like protein 5 , GN=DCUN1D5	1	1	1	237		27.491	5.58

Q66474	Genome polyprotein OS=Echovirus 6 (strain Charles) PE=3	1	1	1	2191	245.119	6.81
O14645	Axonemal dynein light intermediate polypeptide 1 , GN=DNALI1	1	1	1	258	29.644	8.5
Q15050	Ribosome biogenesis regulatory protein homolog , GN=RRS1	1	1	1	365	41.168	10.7
P49427	Ubiquitin-conjugating enzyme E2 R1 ,	1	1	1	236	26.72	4.54
P09854	DNA polymerase catalytic subunit OS=Human herpesvirus 1 (strain SC16) GN=UL30 PE=3	1	1	1	1235	136.381	7.37
Q6P158	Putative ATP-dependent RNA helicase DHX57 , GN=DHX57	1	1	1	1386	155.507	7.71
P51572-2	Isoform 2 of B-cell receptor-associated protein 31 , GN=BCAP31	1	1	1	313	34.73	7.84
P36780	Regulatory protein E2 OS=Human papillomavirus type 9 GN=E2 PE=3	1	1	1	461	52.109	10.05
Q8TAF3	WD repeat-containing protein 48 ,	1	1	1	677	76.162	7.03
P98082	Disabled homolog 2 , GN=DAB2	1	1	1	770	82.397	5.53
Q4KWH8	1-phosphatidylinositol 4,5-bisphosphate phosphodiesterase eta-1 , GN=PLCH1	1	1	1	1693	189.104	7.74
Q9H1J7	Protein Wnt-5b , GN=WNT5B PE=2	1	1	1	359	40.297	8.4
Q13895	Bystin , GN=BYSL	1	1	1	437	49.57	8.12
Q9NRE2	Teashirt homolog 2 , GN=TSHZ2	1	1	1	1034	114.933	7.83
Q6S545	POTE ankyrin domain family member H , GN=POTEH PE=2	1	1	1	545	60.926	7.3
Q96A57-2	Isoform 1 of Transmembrane protein 230 , GN=TMEM230	1	1	1	183	19.898	9.22
Q76I76	Protein phosphatase Slingshot homolog 2 , GN=SSH2	1	1	1	1423	158.116	5.41
P32314	Forkhead box protein N2 , GN=FOXN2	1	1	1	431	47.131	6.42
O75151	Lysine-specific demethylase PHF2 , GN=PHF2	1	1	1	1096	120.7	9.17
O75077	Disintegrin and metalloproteinase domain-containing protein 23 , GN=ADAM23	1	1	1	832	91.867	7.64
Q9UIQ6	Leucyl-cystinyl aminopeptidase , GN=LNPEP	1	1	1	1025	117.274	5.73
Q9UH03	Neuronal-specific septin-3 , GN=SEPT3	1	1	1	358	40.678	7.2
O94822-3	Isoform 3 of E3 ubiquitin-protein ligase listerin , GN=LTN1	1	1	1	1812	205.046	6.52
Q9NZQ3	NCK-interacting protein with SH3 domain , GN=NCKIPSD	1	1	1	722	78.91	6.38
B1AK76	Putative SNURF-like protein , GN=SNURFL	1	1	1	121	14.236	8.62
Q6NT76-5	Isoform 5 of Homeobox-containing protein 1 , GN=HMBOX1	1	1	1	443	49.836	5.76
O94850	Dendrin , GN=DDN	1	1	1	711	75.95	10.17
P20138	Myeloid cell surface antigen CD33 , GN=CD33	1	1	1	364	39.8	8.38
Q9UMX1	Suppressor of fused homolog , GN=SUFU	1	1	1	484	53.913	5.33
Q86UE4	Protein LYRIC , GN=MTDH	1	1	1	582	63.799	9.32
Q9UDR5	Alpha-aminoadipic semialdehyde synthase, mitochondrial , GN=AASS	1	1	1	926	102.066	6.64
Q96AG4	Leucine-rich repeat-containing protein 59 , GN=LRRC59	1	1	1	307	34.909	9.57
Q13574	Diacylglycerol kinase zeta , GN=DGKZ	1	1	1	1117	124.05	9.04
Q96SB8-2	Isoform 2 of Structural maintenance of chromosomes protein 6 , GN=SMC6	1	1	1	1117	129.073	7.15
Q8N695	Sodium-coupled monocarboxylate transporter 1 , GN=SLC5A8	1	1	1	610	66.534	7.75
Q8IUZ5	5-phosphohydroxy-L-lysine phospho-lyase , GN=PHYKPL	1	1	1	450	49.679	6.76
P02462	Collagen alpha-1(IV) chain , GN=COL4A1	1	1	1	1669	160.514	8.28
Q9UNA4	DNA polymerase iota , GN=POLI	1	1	1	740	82.954	6.07
Q8N5M1	ATP synthase mitochondrial F1 complex assembly factor 2 , GN=ATPAF2	1	1	1	289	32.752	7.09
O94851	[F-actin]-monooxygenase MICAL2 ,	1	1	1	1124	126.609	8.65
O60437	Periplakin , GN=PPL	1	1	1	1756	204.623	5.6
Q83887	Envelopment polyprotein OS=New York virus GN=GP	1	1	1	1140	125.536	7.31
Q9HAU5	Regulator of nonsense transcripts 2 ,	1	1	1	1272	147.717	5.69
P23610	Factor VIII intron 22 protein , GN=F8A1	1	1	1	371	39.079	6.84

Q96CD2	Phosphopantothenoylcysteine decarboxylase , GN=PPCDC	1	1	1	204	22.381	6.1
Q96A33	Coiled-coil domain-containing protein 47 , GN=CCDC47	1	1	1	483	55.838	4.87
Q8IY17-4	Isoform 4 of Neuropathy target esterase , GN=PNPLA6	1	1	1	1375	150.859	7.74
Q9UDY2-7	Isoform 7 of Tight junction protein ZO-2 , GN=TJP2	1	1	1	1221	137.258	8.05
O15198	Mothers against decapentaplegic homolog 9 , GN=SMAD9	1	1	1	467	52.46	7.77
A0JNW5	UHRF1-binding protein 1-like ,	1	1	1	1464	164.095	6.32
Q9H4L4	Sentrin-specific protease 3 , GN=SENP3	1	1	1	574	64.969	8.56
O43676	NADH dehydrogenase [ubiquinone] 1 beta subcomplex subunit 3 , GN=NDUFB3	1	1	1	98	11.395	9.2
A6NFK2	Glutaredoxin domain-containing cysteine-rich protein 2 , GN=GRXCR2 PE=3	1	1	1	248	28.266	6.68
O95218	Zinc finger Ran-binding domain-containing protein 2 , GN=ZRANB2	1	1	1	330	37.382	10.01
P11234-2	Isoform 2 of Ras-related protein Ral-B ,	1	1	1	228	25.95	7.81
P00450	Ceruloplasmin , GN=CP	1	1	1	1065	122.128	5.72
P54750-5	Isoform 5 of Calcium/calmodulin-dependent 3',5'-cyclic nucleotide phosphodiesterase 1A , GN=PDE1A	1	1	1	552	63	6.13
Q8TAM6-2	Isoform 2 of Ermin , GN=ERMN	1	1	1	297	34.28	4.92
O15079-2	Isoform 2 of Syntaphilin , GN=SNPH	1	1	1	538	57.954	5.94
Q01804	OTU domain-containing protein 4 ,	1	1	1	1114	123.968	6.71
Q7Z5L9-3	Isoform 3 of Interferon regulatory factor 2-binding protein 2 , GN=IRF2BP2	1	1	1	163	17.128	7.66
P05186-2	Isoform 2 of Alkaline phosphatase, tissue-nonspecific isozyme , GN=ALPL	1	1	1	447	48.878	6.6
P49674	Casein kinase I isoform epsilon , GN=CSNK1E	1	1	1	416	47.285	9.66
Q9Y3L3	SH3 domain-binding protein 1 , GN=SH3BP1	1	1	1	701	75.666	6.77
Q6F5E8	Capping protein, Arp2/3 and myosin-I linker protein 2 , GN=CARMIL2	1	1	1	1435	154.593	6.76
Q9P109	Beta-1,3-galactosyl-O-glycosyl-glycoprotein beta-1,6-N-acetylglucosaminyltransferase 4 , GN=GCNT4 PE=2	1	1	1	453	53.018	8.25
Q80903	Regulatory protein E2 OS=Human papillomavirus type 37 GN=E2 PE=3	1	1	1	454	51.254	10.15
P59817	Zinc finger protein 280A , GN=ZNF280A PE=2	1	1	1	542	60.777	8.76
Q8N142-2	Isoform 2 of Adenylosuccinate synthetase isozyme 1 , GN=ADSSL1	1	1	1	500	54.528	9.03
P41440	Folate transporter 1 , GN=SLC19A1	1	1	1	591	64.827	8.95
O43310-2	Isoform 2 of CBP80/20-dependent translation initiation factor , GN=CTIF	1	1	1	600	67.741	6.54
P10114	Ras-related protein Rap-2a , GN=RAP2A	1	1	1	183	20.602	4.82
Q5H9K5	Zinc finger matrin-type protein 1 , GN=ZMAT1 PE=2	1	1	1	638	74.708	8.38
Q6SW82	Protein UL49 OS=Human cytomegalovirus (strain Merlin) GN=UL49 PE=3	1	1	1	570	63.792	9.03
P15155	Outer capsid protein VP4 OS=Rotavirus B (isolate RVB/Rat/United States/IDIR/1984/G1P[X]) PE=3	1	1	1	751	85.503	6.39
Q9H0W8	Protein SMG9 , GN=SMG9	1	1	1	520	57.614	7.01
Q9NX47	E3 ubiquitin-protein ligase MARCH5 , GN=MARCH5	1	1	1	278	31.211	8.7
Q96B49	Mitochondrial import receptor subunit TOM6 homolog , GN=TOMM6	1	1	1	74	7.997	4.89
Q5TC84	Opioid growth factor receptor-like protein 1 , GN=OGFRL1 PE=2	1	1	1	451	51.22	6.13
Q9Y5E7	Protocadherin beta-2 , GN=PCDHB2	1	1	1	798	87.2	4.89
Q8IXS2-2	Isoform 2 of Coiled-coil domain-containing protein 65 , GN=CCDC65	1	1	1	497	58.704	7.94
P42892	Endothelin-converting enzyme 1 , GN=ECE1	1	1	1	770	87.108	5.88
O43683	Mitotic checkpoint serine/threonine-protein kinase BUB1 , GN=BUB1	1	1	1	1085	122.297	6.47
P46781	40S ribosomal protein S9 , GN=RPS9	1	1	1	194	22.578	10.65

Q9BQI0-2	Isoform 2 of Allograft inflammatory factor 1-like , GN=AIF1L	1	1	1	176		20.002	7.77
Q7Z7L7	Protein zer-1 homolog , GN=ZER1	1	1	1	766		88.113	5.62
Q5VTT5	Myomesin-3 , GN=MYOM3	1	1	1	1437		162.088	6.23
Q5T7V8	RAB6-interacting golgin , GN=GORAB	1	1	1	394		44.965	7.08
Q9Y3Q0	N-acetylated-alpha-linked acidic dipeptidase 2 , GN=NAALAD2	1	1	1	740		83.539	8.5
Q9BZH6	WD repeat-containing protein 11 ,	1	1	1	1224		136.598	6.92
Q6E0U4	Dermokine , GN=DMKN	1	1	1	476		47.054	7.28
Q5XKP0	MICOS complex subunit MIC13 , GN=MIC13	1	1	1	118		13.079	9.42
Q00059	Transcription factor A, mitochondrial ,	1	1	1	246		29.078	9.72
Q6EMB2-2	Isoform 2 of Tubulin polyglutamylase TTLL5 , GN=TTLL5	1	1	1	817		91.465	9.26
P58004	Sestrin-2 , GN=SESN2	1	1	1	480		54.459	5.9
P03521	Nucleoprotein OS=Vesicular stomatitis Indiana virus (strain San Juan) GN=N	1	1	1	422		47.379	6.71
O15194	CTD small phosphatase-like protein , GN=CTDSPL	1	1	1	276		31.109	5.54
P42768	Wiskott-Aldrich syndrome protein , GN=WAS	1	1	1	502	52.88		6.64
Q9NTG7	NAD-dependent protein deacetylase sirtuin-3, mitochondrial , GN=SIRT3	1	1	1	399		43.546	8.7
Q14055	Collagen alpha-2(IX) chain , GN=COL9A2	1	1	1	689		65.091	9.17
Q8TBE0	Bromo adjacent homology domain-containing 1 protein , GN=BAHD1	1	1	1	780		84.599	9.07
Q8N8X9	Protein mab-21-like 3 , GN=MAB21L3	1	1	1	362	42.33		8.54
P10200	OS=Human herpesvirus 1 (strain 17) GN=UL16	1	1	1	373		40.417	7.74
Q96D71	RalBP1-associated Eps domain-containing protein 1 , GN=REPS1	1	1	1	796		86.609	5.69
Q9NUZ1-4	Isoform 4 of Acyl-coenzyme A oxidase-like protein , GN=ACOXL	1	1	1	580		65.415	9.22
Q9UII2	ATPase inhibitor, mitochondrial , GN=ATPIF1	1	1	1	106		12.241	9.35
Q8N5M4	Tetratricopeptide repeat protein 9C ,	1	1	1	171		20	8.92
Q92796	Disks large homolog 3 , GN=DLG3	1	1	1	817		90.258	7.03
P49675	Steroidogenic acute regulatory protein, mitochondrial , GN=STAR	1	1	1	285		31.893	9.03
P54315	Inactive pancreatic lipase-related protein 1 , GN=PNLIPRP1	1	1	1	467		51.815	5.73
Q56VL3	OCIA domain-containing protein 2 ,	1	1	1	154		16.943	9.03
Q6ZQQ6-2	Isoform 2 of WD repeat-containing protein 87 , GN=WDR87	1	1	1	2912		337.564	7.28
Q9Y4F4	TOG array regulator of axonemal microtubules protein 1 , GN=TOGARAM1	1	1	1	1720		189.242	8.5
O43861	Probable phospholipid-transporting ATPase IIB , GN=ATP9B PE=2	1	1	1	1147	129.22		7.61
P27987	Inositol-trisphosphate 3-kinase B , GN=ITPKB	1	1	1	946		102.312	8.43
Q5TCQ9	Membrane-associated guanylate kinase, WW and PDZ domain-containing protein 3 , GN=MAGI3	1	1	1	1506		165.506	8.02
Q81258	Genome polyprotein OS=Hepatitis C virus genotype 3a (isolate NZL1)	1	1	1	3021		329.366	8.07
Q9NZH5	Securin-2 , GN=PTTG2 PE=2	1	1	1	202		22.288	6.25
Q4KMZ1-3	Isoform 3 of IQ domain-containing protein C , GN=IQCC	1	1	1	546		61.928	8.43
Q9P0N9	TBC1 domain family member 7 , GN=TBC1D7	1	1	1	293	33.95		7.94
Q68D20	Protein PMS2CL , GN=PMS2CL PE=2	1	1	1	193		20.896	5.34
Q9H967	WD repeat-containing protein 76 ,	1	1	1	626		69.725	9.25
A2RU30-3	Isoform 3 of Protein TESPA1 , GN=TESPA1	1	1	1	394		45.202	8.12
Q9Y4D8-5	Isoform 5 of Probable E3 ubiquitin-protein ligase HECTD4 , GN=HECTD4	1	1	1	1321		144.984	5.78
Q53QZ3	Rho GTPase-activating protein 15 , GN=ARHGAP15	1	1	1	475	54.51		9.39
Q9BV73	Centrosome-associated protein CEP250 , GN=CEP250	1	1	1	2442		280.967	5.02
Q12778	Forkhead box protein O1 , GN=FOXO1	1	1	1	655		69.617	6.77
Q8WWA0	Intelectin-1 , GN=ITLN1	1	1	1	313		34.939	6.01

Q8NHK3	Killer cell immunoglobulin-like receptor 2DL5B , GN=KIR2DL5B PE=3	1	1	1	375		40.581	8.1
P55042	GTP-binding protein RAD , GN=RRAD	1	1	1	308		33.225	8.88
O15014	Zinc finger protein 609 , GN=ZNF609	1	1	1	1411		151.098	8.03
Q9H583	HEAT repeat-containing protein 1 ,	1	1	1	2144		242.215	6.54
Q96KG9	N-terminal kinase-like protein , GN=SCYL1	1	1	1	808		89.575	6.3
Q9NR19-2	Isoform 2 of Acetyl-coenzyme A synthetase, cytoplasmic , GN=ACSS2	1	1	1	714		80.036	6.74
O00116	Alkyldihydroxyacetonephosphate synthase, peroxisomal , GN=AGPS	1	1	1	658		72.866	7.34
P78332	RNA-binding protein 6 , GN=RBM6	1	1	1	1123		128.565	6.32
P55287	Cadherin-11 , GN=CDH11 PE=2	1	1	1	796		87.911	4.91
Q9HC57	WAP four-disulfide core domain protein 1 , GN=WFDC1 PE=2	1	1	1	220		23.961	8.38
Q15759	Mitogen-activated protein kinase 11 , GN=MAPK11	1	1	1	364		41.331	5.83
P09613	Envelopment polyprotein OS=Uukuniemi virus (strain S23) GN=GP	1	1	1	1008		113.515	7.84
Q8N5C6	S1 RNA-binding domain-containing protein 1 , GN=SRBD1	1	1	1	995		111.705	8.72
Q6JQN1-5	Isoform 5 of Acyl-CoA dehydrogenase family member 10 , GN=ACAD10	1	1	1	1090		122.262	8.32
Q9Y5B8	Nucleoside diphosphate kinase 7 , GN=NME7	1	1	1	376		42.464	6.47
A6NCF6	Putative MAGE domain-containing protein MAGEA13P , GN=MAGEA13P PE=5	1	1	1	341		37.875	6.37
Q16619	Cardiotrophin-1 , GN=CTF1	1	1	1	201		21.214	9.01
O60547	GDP-mannose 4,6 dehydratase , GN=GMDS	1	1	1	372		41.923	7.31
Q9BTL4	Immediate early response gene 2 protein , GN=IER2	1	1	1	223	24.18		6.92
P23515	Oligodendrocyte-myelin glycoprotein ,	1	1	1	440		49.576	7.94
Q14197	Peptidyl-tRNA hydrolase ICT1, mitochondrial , GN=MRPL58	1	1	1	206		23.615	10.07
P32243-2	Isoform 2 of Homeobox protein OTX2 ,	1	1	1	297		32.386	9.23
Q96N16	Janus kinase and microtubule-interacting protein 1 , GN=JAKMIP1	1	1	1	626		73.164	6.1
Q07343	cAMP-specific 3',5'-cyclic phosphodiesterase 4B , GN=PDE4B	1	1	1	736		83.291	5.25
Q9NRJ5	Poly(A) polymerase beta , GN=PAPOLB PE=2	1	1	1	636		71.636	6.44
Q9Y2L5	Trafficking protein particle complex subunit 8 , GN=TRAPPC8	1	1	1	1435		160.896	6.87
Q8NB50	Zinc finger protein 62 homolog , GN=ZFP62	1	1	1	900		102.445	9.04
Q13495-3	Isoform 2 of Mastermind-like domain-containing protein 1 , GN=MAMLD1	1	1	1	998	106.43		7.4
Q9BRA2	Thioredoxin domain-containing protein 17 , GN=TXNDC17	1	1	1	123		13.932	5.52
O12158	Gag-Pol polyprotein OS=Human immunodeficiency virus type 1 group M subtype C (isolate 92BR025) GN=gag-pol	1	1	1	1431		161.569	8.75
Q96P15	Serpin B11 , GN=SERPINB11 PE=2	1	1	1	392	44.07		8.25
Q68DQ2	Very large A-kinase anchor protein ,	1	1	1	2970		330.427	5.2
P12821	Angiotensin-converting enzyme , GN=ACE	1	1	1	1306	149.62		6.39
Q96JE7	Protein transport protein Sec16B ,	1	1	1	1060		116.532	6.09
P51530	DNA replication ATP-dependent helicase/nuclease DNA2 , GN=DNA2	1	1	1	1060		120.337	7.74
A6NNA2	Serine/arginine repetitive matrix protein 3 , GN=SRRM3 PE=2	1	1	1	597	65.21		11.68
P78417	Glutathione S-transferase omega-1 ,	1	1	1	241		27.548	6.6
P30542	Adenosine receptor A1 , GN=ADORA1	1	1	1	326		36.488	8.62
Q9H900	Protein zwilch homolog , GN=ZWILCH	1	1	1	591		67.172	6.27
Q6PID8	Kelch domain-containing protein 10 , GN=KLHDC10	1	1	1	442		49.067	9.38
Q8WXC6-1	Isoform 2 of COP9 signalosome complex subunit 9 , GN=COPS9	1	1	1	252		27.648	6.33
Q6DN72	Fc receptor-like protein 6 , GN=FCRL6	1	1	1	434		47.717	7.58
P12596	Non-structural protein 1 OS=Influenza B virus (strain B/ID/1986) GN=NS PE=3	1	1	1	281		31.938	6.34
P41236	Protein phosphatase inhibitor 2 , GN=PPP1R2	1	1	1	205		23.001	4.74

P36268-3	Isoform 3 of Inactive glutathione hydrolase 2 , GN=GGT2	1	1	1	574		62.082	7.33	
P62873	Guanine nucleotide-binding protein G(I)/G(S)/G(T) subunit beta-1 , GN=GNB1	1	1	1	340		37.353		6
Q9BVC6	Transmembrane protein 109 , GN=TMEM109	1	1	1	243		26.194	10.48	
O00592	Podocalyxin , GN=PODXL	1	1	1	558		58.599	5.49	
Q6DD87	Zinc finger protein 787 , GN=ZNF787	1	1	1	383		40.518	7.84	
P57057	Glucose-6-phosphate exchanger SLC37A1 , GN=SLC37A1 PE=2	1	1	1	533		57.611	8.31	
Q13112	Chromatin assembly factor 1 subunit B , GN=CHAF1B	1	1	1	559		61.454	7.5	
O94927	HAUS augmin-like complex subunit 5 , GN=HAUS5	1	1	1	633		71.638	8.51	
A6NHX0	Cytosolic arginine sensor for mTORC1 subunit 2 , GN=CASTOR2	1	1	1	329		36.034	5.16	
Q6UXK5	Leucine-rich repeat neuronal protein 1 , GN=LRRN1	1	1	1	716		80.665	6.15	
Q9UDY8	lymphoma translocation protein 1 , GN=MALT1	1	1	1	824		92.213	5.73	
P19623	Spermidine synthase , GN=SRM	1	1	1	302		33.803	5.49	
Q9BZ72	Membrane-associated phosphatidylinositol transfer protein 2 , GN=PITPNM2	1	1	1	1349	148.84		7.17	
Q70J99-3	Isoform 3 of Protein unc-13 homolog D , GN=UNC13D	1	1	1	1142		128.739	6.62	
P05882	Envelope glycoprotein gp160 OS=Human immunodeficiency virus type 1 group M subtype D (isolate Z84) GN=env PE=3	1	1	1	863		97.681	8.46	
Q5VZ89-7	Isoform 2 of DENN domain-containing protein 4C , GN=DENND4C	1	1	1	1958		217.681	6.64	
O15417	Trinucleotide repeat-containing gene 18 protein , GN=TNRC18	1	1	1	2968		314.326	8.7	
Q86UC2	Radial spoke head protein 3 homolog , GN=RSPH3	1	1	1	560		63.647	5.69	
F5HF35	Protein UL136 OS=Human cytomegalovirus (strain Merlin) GN=UL136	1	1	1	240		27.042	8.06	
O15119	T-box transcription factor TBX3 , GN=TBX3	1	1	1	743		79.339	8.16	
Q5GH72	XK-related protein 7 , GN=XKR7 PE=2	1	1	1	579		63.785	9.01	
Q2M3V2	Ankyrin repeat domain-containing protein SOWAHA , GN=SOWAHA	1	1	1	549		57.407	10.18	
Q9UMN6	Histone-lysine N-methyltransferase 2B , GN=KMT2B	1	1	1	2715	293.33		8.22	
P59768	Guanine nucleotide-binding protein G(I)/G(S)/G(O) subunit gamma-2 , GN=GNG2	1	1	1	71		7.845	7.99	
P12757	Ski-like protein , GN=SKIL	1	1	1	684		76.927	7.11	
P29597	Non-receptor tyrosine-protein kinase TYK2 , GN=TYK2	1	1	1	1187		133.565	7.15	
Q8IV45	UNC5C-like protein , GN=UNC5CL	1	1	1	518		57.781	6.67	
Q9NZM3	Intersectin-2 , GN=ITSN2	1	1	1	1697	193.34		8.12	
Q96DE5	Anaphase-promoting complex subunit 16 , GN=ANAPC16	1	1	1	110	11.66		4.97	
Q9BZF9	Uveal autoantigen with coiled-coil domains and ankyrin repeats , GN=UACA	1	1	1	1416		162.404	7.03	
P21404	Genome polyprotein OS=Coxsackievirus A9 (strain Griggs)	1	1	1	2201		246.376	7.09	
Q7Z5P9	Mucin-19 , GN=MUC19	1	1	1	8384		804.768	5.01	
Q6ZNL6	FYVE, RhoGEF and PH domain-containing protein 5 , GN=FGD5	1	1	1	1462		159.791	5.01	
P16849	G-protein coupled receptor homolog UL33 OS=Human cytomegalovirus (strain AD169) GN=UL33	1	1	1	412		46.266	9.8	
Q93097	Protein Wnt-2b , GN=WNT2B	1	1	1	391		43.742		9
O14647	Chromodomain-helicase-DNA-binding protein 2 , GN=CHD2	1	1	1	1828		211.214	8.1	
P31271	Homeobox protein Hox-A13 , GN=HOXA13	1	1	1	388		39.702	9.13	
Q9Y3D9	28S ribosomal protein S23, mitochondrial , GN=MRPS23	1	1	1	190		21.757	8.9	

Q6UB99	Ankyrin repeat domain-containing protein 11 , GN=ANKRD11	1	1	1	2663	297.731	7.11
Q6P1J6-2	Isoform 2 of Phospholipase B1, membrane-associated , GN=PLB1	1	1	1	488	54.307	7.27
Q8IV35	WD repeat-containing protein 49 , GN=WDR49 PE=2	1	1	1	697	79.244	8.47
P22674	Cyclin-O , GN=CCNO	1	1	1	350	38.071	8.06
Q9UBL0-3	Isoform 3 of cAMP-regulated phosphoprotein 21 , GN=ARPP21	1	1	1	813	88.515	6.95
P0DOY2	Immunoglobulin lambda constant 2 ,	1	1	1	106	11.287	7.24
Q5K4E3	Polyserase-2 , GN=PRSS36	1	1	1	855	91.896	5.66
P87691	Hemagglutinin-esterase-fusion glycoprotein OS=Influenza C virus (strain C/Yamagata/4/1988) GN=HE PE=3	1	1	1	648	71.097	5.86
Q13625-3	Isoform 3 of Apoptosis-stimulating of p53 protein 2 , GN=TP53BP2	1	1	1	1134	126.245	6.21
P51511	Matrix metalloproteinase-15 , GN=MMP15	1	1	1	669	75.759	7.46
Q9NR81-2	Isoform 2 of Rho guanine nucleotide exchange factor 3 , GN=ARHGEF3	1	1	1	558	63.715	6.92
